# Mechanical macrocyclic effects: stimuli-responsive dual emission by self-inclusion and mutual exclusion

**DOI:** 10.1039/d6sc06062e

**Published:** 2026-10-02

**Authors:** Seungyeon Hyun, Doui Kim, Dongjin Choi, Eunkyeong Choi, Jongcheol Seo, Dongwhan Lee

**Affiliations:** a Department of Chemistry, Seoul National University 1 Gwanak-ro, Gwanak-gu Seoul 08826 Korea dongwhan@snu.ac.kr; b Department of Chemistry, Pohang University of Science and Technology (POSTECH) 77 Cheongam-ro Pohang Gyeongsangbuk-do 37673 Korea; c School of Interdisciplinary Bioscience and Bioengineering (I-BIO), Pohang University of Science and Technology (POSTECH) 77 Cheongam-ro Pohang Gyeongsangbuk-do 37673 Korea

## Abstract

A macrocycle divides space into an inside and an outside. This physical boundary allows a guest to reside either within the cavity or outside. When two guests are tethered to one macrocycle, they compete for the same cavity and mutually exclude each other. We exploit this simple principle to design a macrocyclic molecular device with two identical fluorophores. By design, when one fluorophore pendant is sequestered by the nonpolar cavity (“*in*”), the other is automatically exposed to the polar external environment (“*out*”), producing two distinct emission bands. This positional asymmetry arises from self-inclusion, mutual exclusion, and rigid-body tilting, all working cooperatively. The resulting dual emission reports the contrasting environments experienced by the two identical fluorophores within the same molecule, with the relative populations of the *in*- and *out*-states controlled by conformational switching driven by solvent polarity, temperature, or host–guest interactions.

## Abstract 2

By self-inclusion and mutual exclusion, a macrocycle renders two identical fluorophores functionally nonequivalent. The resulting dual emission is controlled by solvent polarity, temperature, and guest binding.

## Graphical abstract

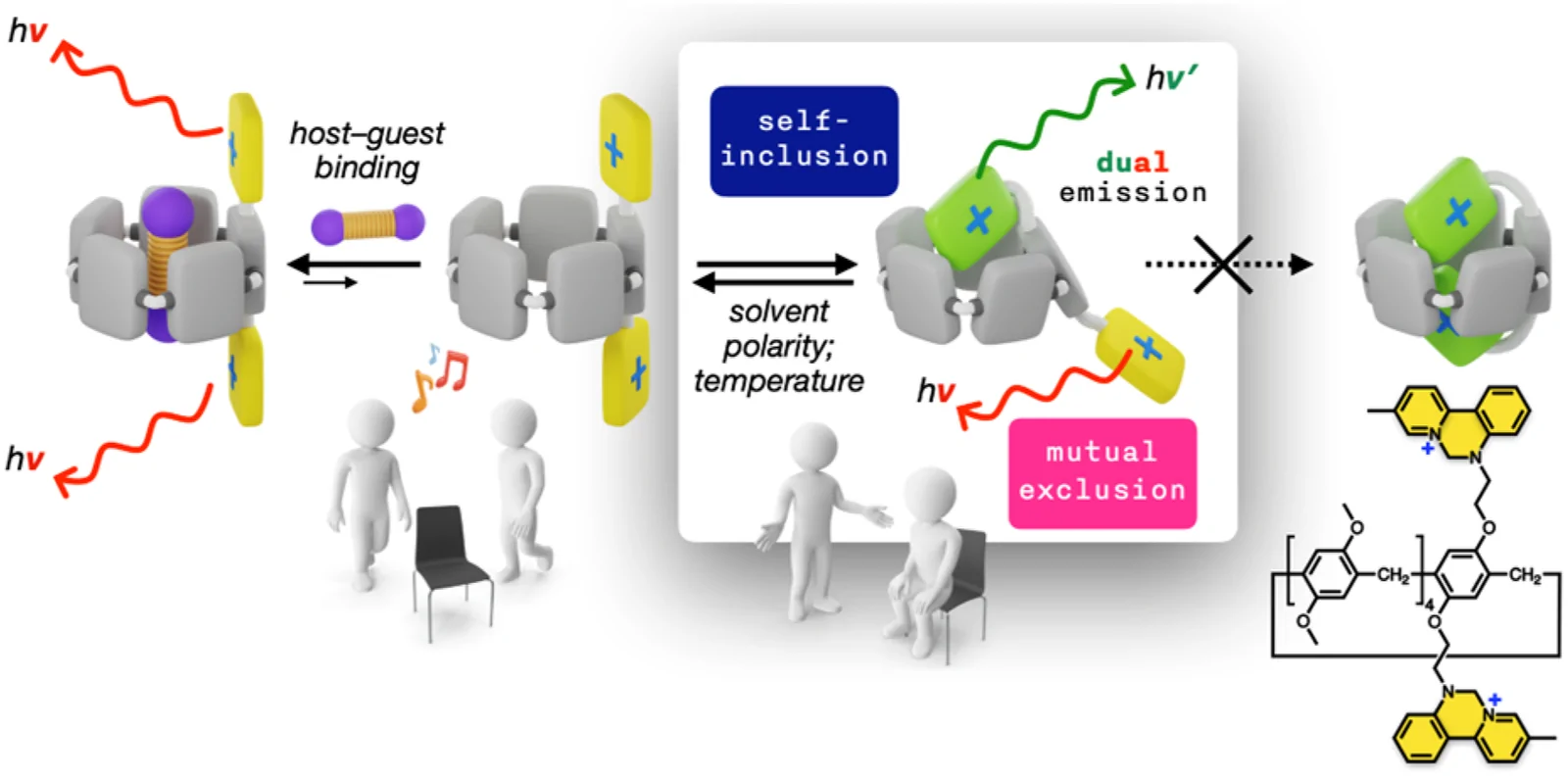

## Introduction

A macrocycle is formed by bending a linear molecule and joining its ends to define a cavity. As a result, space is divided into an inside and an outside. This geometric transformation from an open chain to a cyclic boundary is a cornerstone of supramolecular chemistry, as exemplified by crown ethers,^1,2^ which recognize guests to form complexes.^3,4^ The macrocyclic cavity creates an interior phase that can differ from the surrounding bulk environment (Fig. 1a).^5^ Because this confined space constrains guest motion and provides a defined reaction microenvironment, intra-cavity inclusion has become a central paradigm in molecular recognition,^6,7^ transport,^8–10^ and catalysis.^11,12^

**Fig. 1:**
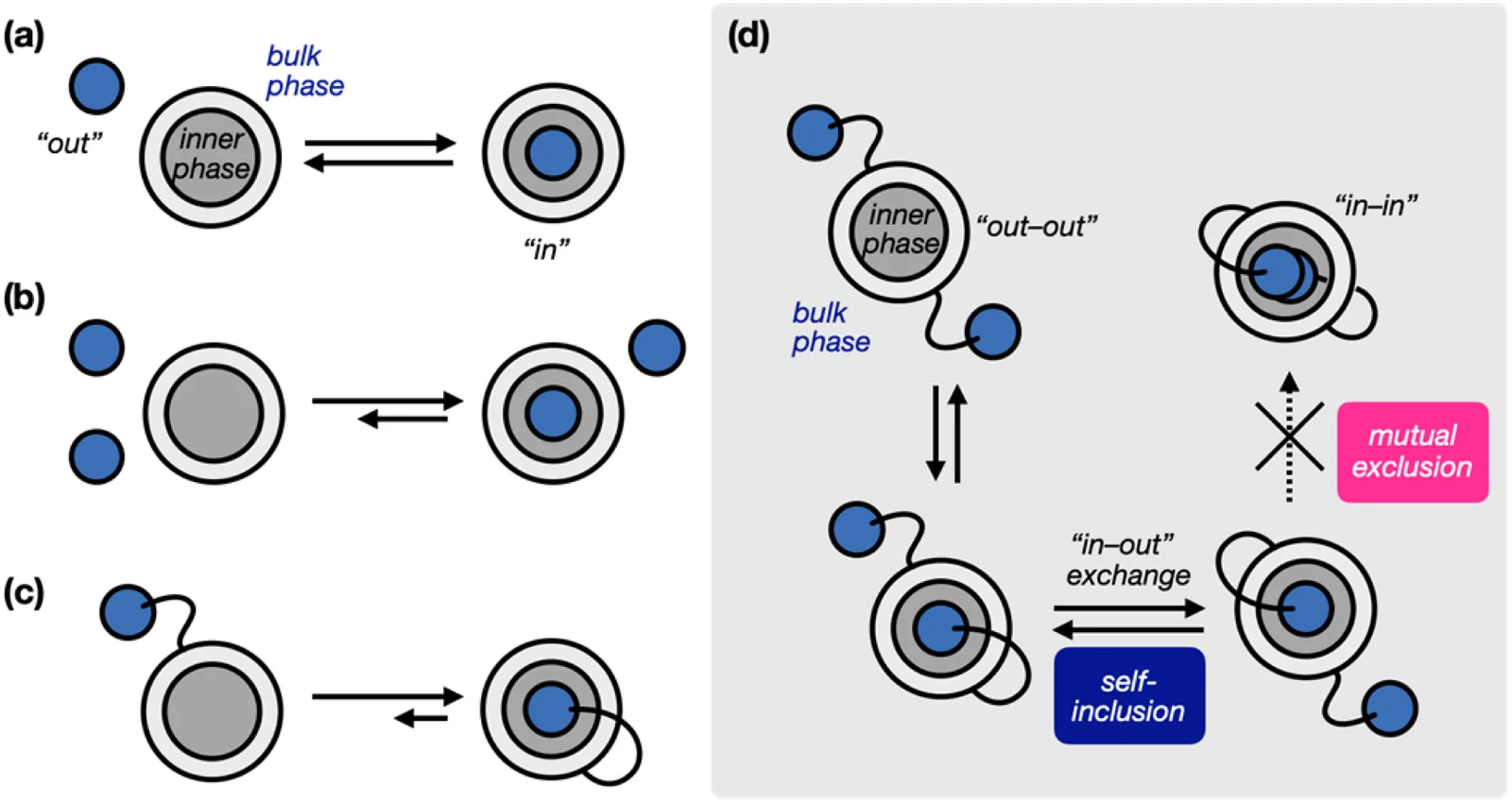
Schematic representations of macrocycle-based host–guest systems. (a) A macrocycle divides space into an interior phase and the surrounding bulk environment. (b) Intermolecular host–guest complexation as a concentration-dependent stochastic process. (c) Self-inclusion of a single covalently tethered pendant. (d) Two identical pendants compete for the same cavity, leading to mutual exclusion and a structurally enforced asymmetric state.

In intermolecular host–guest systems, an externally added guest experiences the boundary between the interior phase and the bulk environment. Inclusion is therefore a stochastic process governed by guest concentration: increasing the guest concentration raises the frequency of productive encounters and drives the equilibrium toward complexation (Fig. 1b). Covalently tethering the guest to the host changes this situation in two ways. First, it raises the effective concentration of the guest near the cavity and shifts the equilibrium toward inclusion (Fig. 1c). More importantly, once host and guest are linked as parts of a single molecule, the once-intermolecular process becomes intramolecular. As in molecular machines such as catenanes^13,14^ and rotaxanes,^15,16^ this self-contained assembly operates independently of bulk concentration. Self-inclusion complexation has therefore been exploited in various supramolecular devices, including pseudo[1]rotaxanes^17–19^ and molecular lassos.^20–22^ In a single-tethered pendant system, however, the host-linked guest can occupy only one environment at any given moment, either inside or outside.

Having attached one pendant to a macrocyclic rim, one can logically extend the design by attaching another identical pendant to the opposite side, producing a more symmetric construct. If a single pendant shuttles *in* and *out* of the cavity and alternately experiences two environments, one might expect that adding a second pendant would simply double the effect, much like increasing the concentration in an intermolecular process. However, the situation unfolds differently. The two pendants now compete for the same cavity. Because they cannot both reside inside at the same time, this competition gives rise to a phenomenon beyond simple amplification. Restriction reduces the degrees of freedom, but in doing so, it creates a new order. When two chemically identical units compete to occupy a single cavity (Fig. 1d, bottom), an asymmetric state is structurally enforced: one unit occupies the interior phase, while the other remains in the bulk environment. In other words, the two units mutually exclude each other (Fig. 1d, right).

This situation changes the meaning of shuttling. In a single-tethered system, one pendant alternates between the inside and the outside over time. In the double-tethered system, however, two identical pendants divide these environments at the same moment. An intuitive analogy is a two-person game of musical chairs, in which only one person can sit at a time. The two may trade places, but one is always seated and the other standing. In our molecular system, this site exchange occurs intramolecularly, with neither tethered unit escaping the continuous cycle. As a result, positional asymmetry emerges despite the symmetric design of the molecule. The key question is how to tell which pendant occupies which environment. One solution is to introduce a fluorophore that exhibits environment-dependent photophysical behavior, so that the occupancy states of the nonpolar interior and the polar exterior can be read out as distinct emission signals (Fig. 2a). When tethered to the cavity, the two chemically identical fluorophores partition between the confined space and the surrounding bulk environment to produce dual emission (Fig. 2b), which can be controlled by polarity, temperature, and host–guest interactions, as detailed in the following sections.

**Fig. 2:**
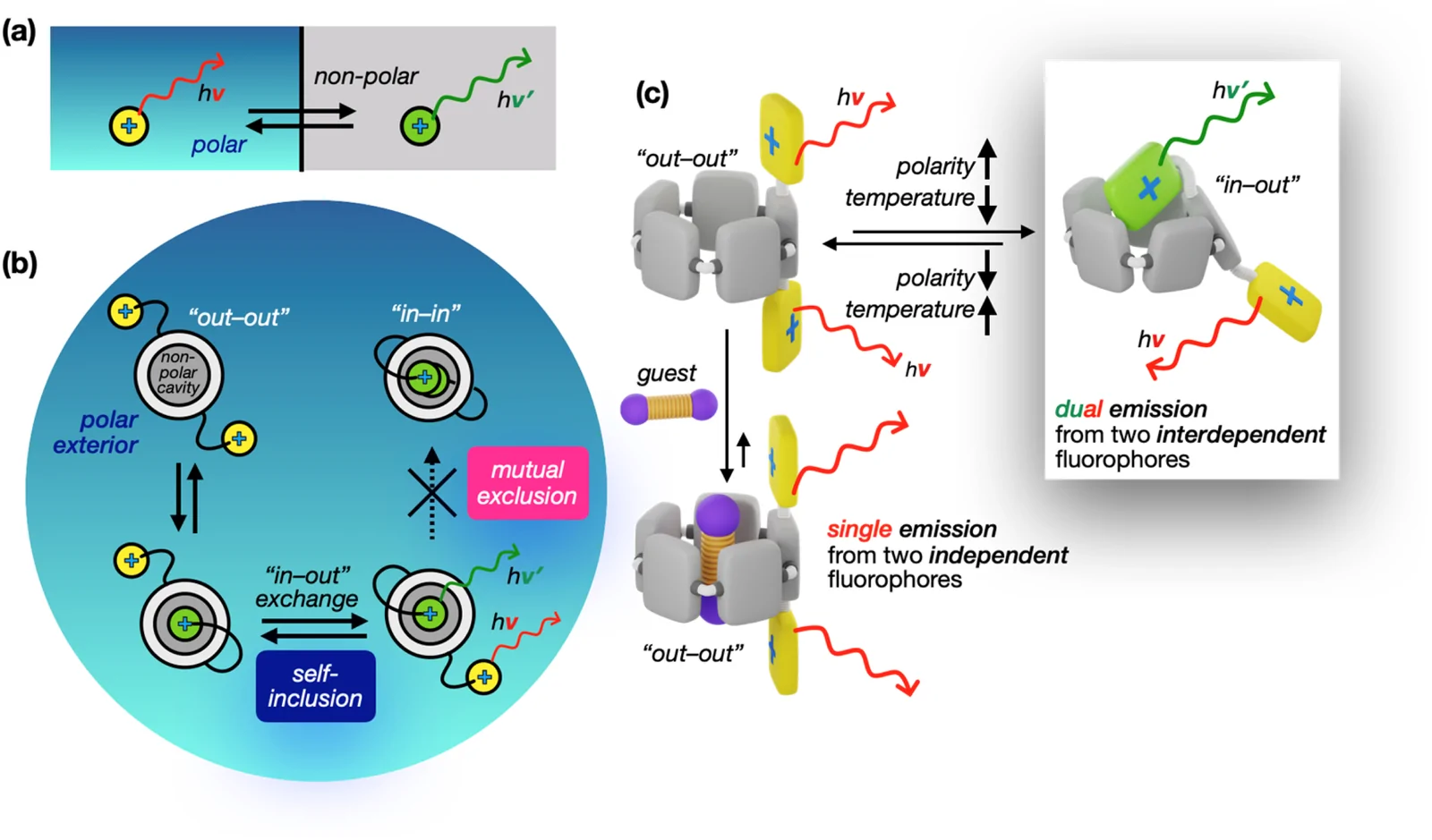
Fluorescence readout of positional asymmetry. (a) A fluorophore exhibits distinct photophysical behavior in polar and nonpolar environments. (b) With two fluorophores tethered to a common host, the *out*–*out* and *in*–*out* conformers equilibrate as a function of environmental polarity, producing dual emission from the *in*-position (green) and the *out*-position (red). (c) Three-dimensional representation of conformational switching between the *out*–*out* single-emitter state and the *in*–*out* dual-emitter state, driven by changes in solvent polarity, temperature, or guest inclusion.

## Design principles

Within a single molecule, it is difficult to establish both nonpolar and polar environments simultaneously, since these are bulk properties that emerge from intermolecular interactions. To compartmentalize nonpolar and polar regions across a molecular interface, we employed the macrocyclic pillar[5]arene scaffold, P5 (Fig. 3).^23^ In addition to providing an aromatic-rich nonpolar cavity, the phenylene panels of P5 can swivel and tilt around the methylene (–CH_2_–) linkers while still maintaining the rigid pentagonal molecular skeleton.^24,25^ With these structural features, P5 has been widely employed in supramolecular chemistry, particularly because its rim positions can be readily modified to install various functional groups.^26–28^

**Fig. 3:**
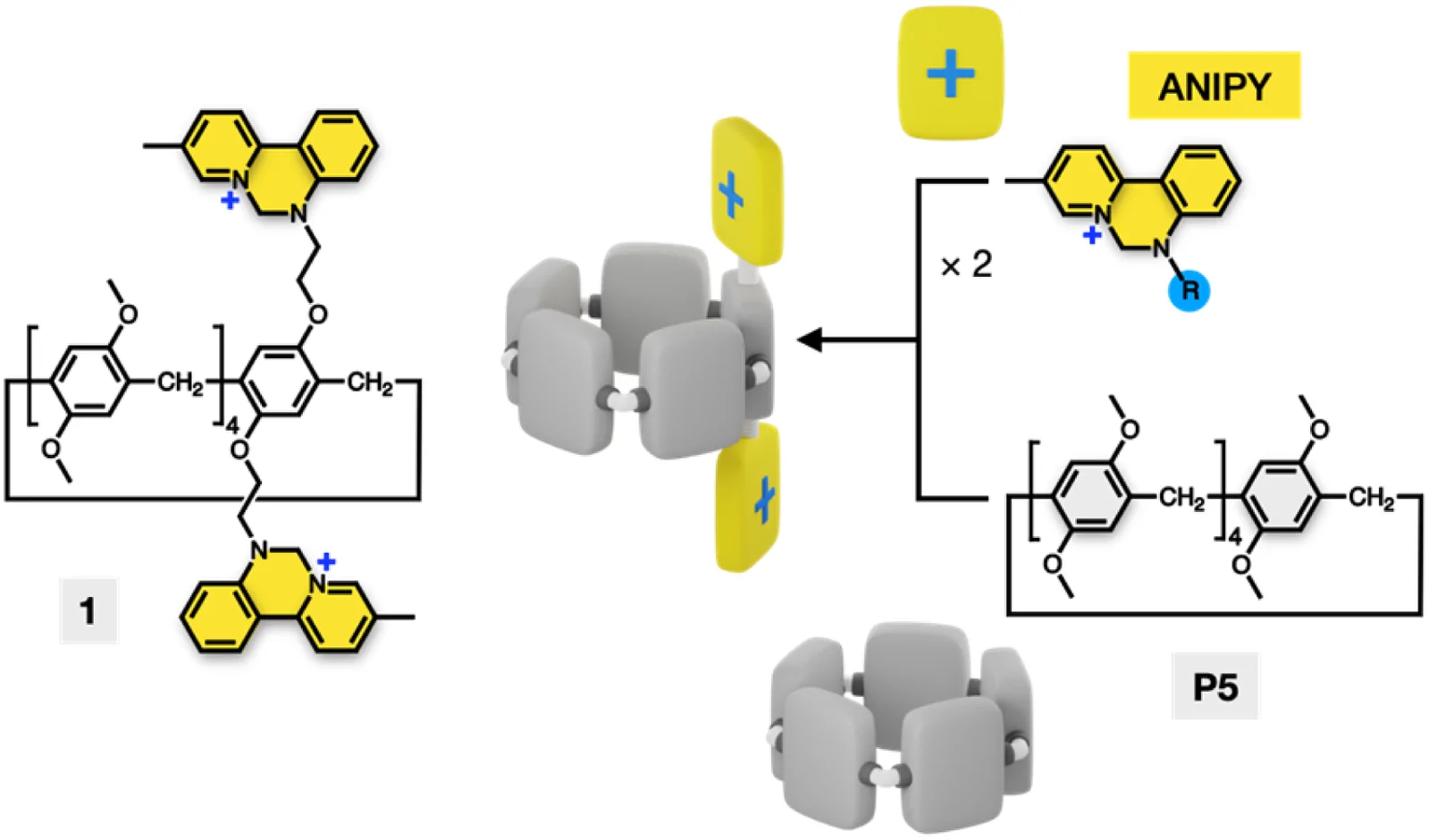
Chemical structure and schematic representation of dicationic 1, composed of two fluorogenic ANIPY units tethered to the pillar[5]arene macrocycle P5.

As the fluorescent polar component to be appended to P5, we chose the anilidopyridyl-derived polyheterocycle ANIPY (Fig. 3), which we recently reported.^29^ As a cationic fluorophore, ANIPY is readily soluble in polar solvents. With their ring-fused large π-surfaces and positive charges, two ANIPY units cannot simultaneously interact with the inner cavity of P5. This mutual exclusion is further reinforced by the seesaw-like mechanical tilting motion of the rim (Fig. 2c). Although electrostatic repulsion, steric constraints, and mechanical tilting operate by independent mechanisms, they work cooperatively to enforce mutual exclusion during site exchange. This mode of action is realized in the bis-functionalized macrocycle 1 (Fig. 3). With its toroidal shape, the P5 host unit cannot provide a fully enclosing three-dimensional cavity. As an operational definition, when the ANIPY unit remains fully solvated, we designate this as the “*out*” state. When folding of the tethered arm toward the P5 fragment begins to displace the solvation shell and initiate direct host–guest contact, we designate this as the “*in*” state. The following sections describe how this molecule responds to contrasting environments.

## Results and discussion

### Synthesis of bis-functionalized fluorescent macrocycle

The fluorophore-appended macrocycle 1 (Fig. 3) was synthesized in a straightforward manner by connecting individual functional components in a modular fashion (Scheme 1). A double substitution reaction between bis-bromoethoxy pillar[5]arene 5 and anilidopyridine 6 afforded intermediate 7, which was subjected to acid-catalyzed condensation with formaldehyde to afford dicationic 1 as a chloride salt.^30,31^ Using 6 and mono-bromoethoxy pillar[5]arene (Scheme S1), we also prepared the mono-functionalized pillar[5]arene cation 2 as a control (Scheme 1b).

**Scheme 1:**
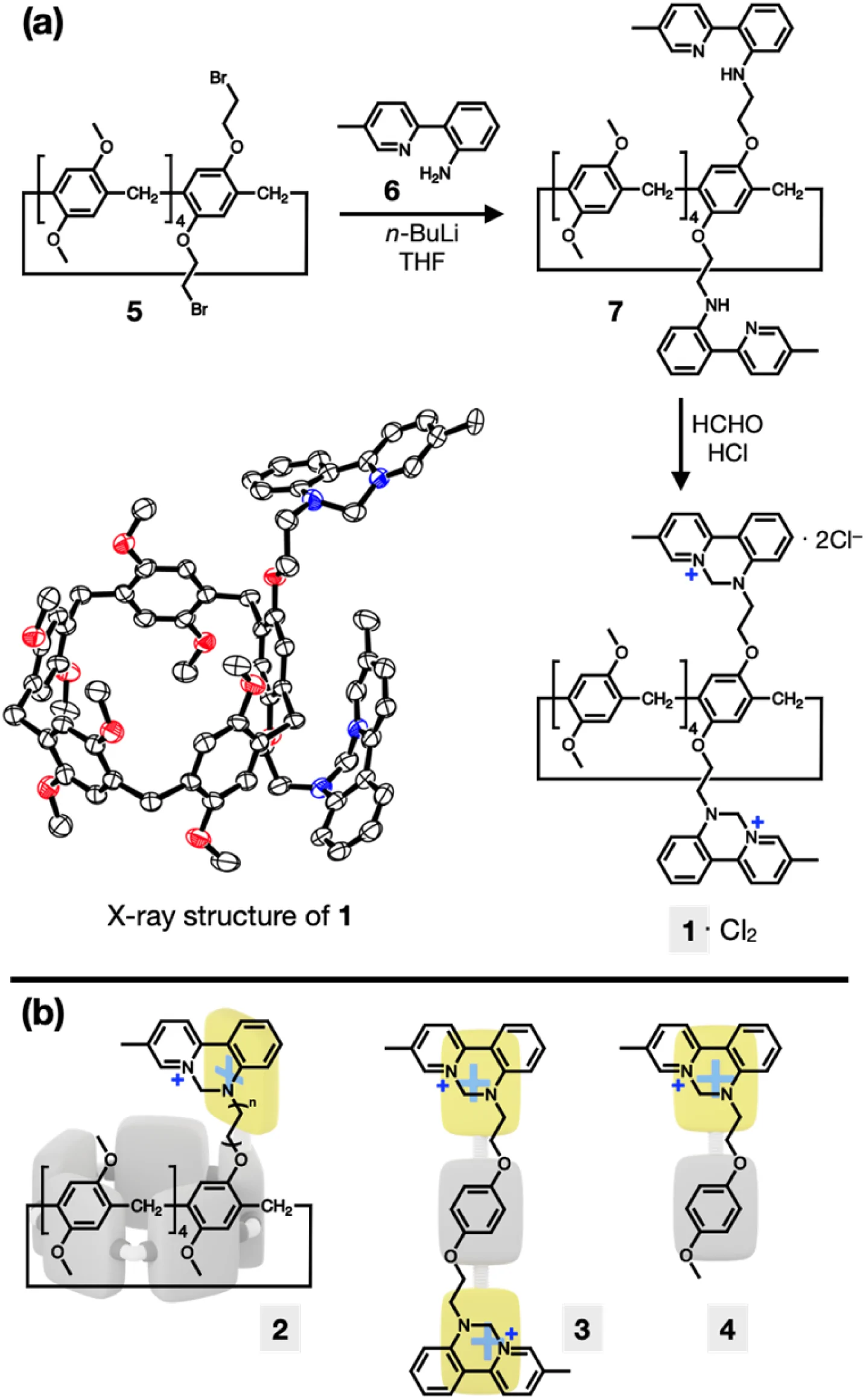
(a) Synthetic route to 1, and (b) chemical structures of model compounds 2–4, overlaid with semitransparent schematic representations.

The three-dimensional structures of both compounds were confirmed by X-ray crystallography (Fig. S1 and Table S1). As macrocycle-free reference systems, 3 and 4 were also prepared as their chloride salts. Comparative studies on these four compounds, with variations in the number of fluorophores and the presence or absence of the macrocyclic host, helped identify the structural requirements needed to realize the design principles outlined in Fig. 2c.

### Different emission from identical fluorophores implicating local asymmetry

With compounds 1–4 in hand, we proceeded to study their photophysical properties (Fig. 4 and Table S2). As shown in Fig. 4a, the fluorescence spectrum of 1 in MeOH features a broad emission band centered at *λ*_max,em_ = 580 nm, accompanied by a blue-shifted shoulder at 495 nm. However, this dual emission was not observed for 3 having two ANIPY groups appended to a phenylene ring as a site model of P5 (Fig. 4c). Compound 3 reproduced the longer-wavelength emission of 1, but not the blue-shifted band.

**Fig. 4:**
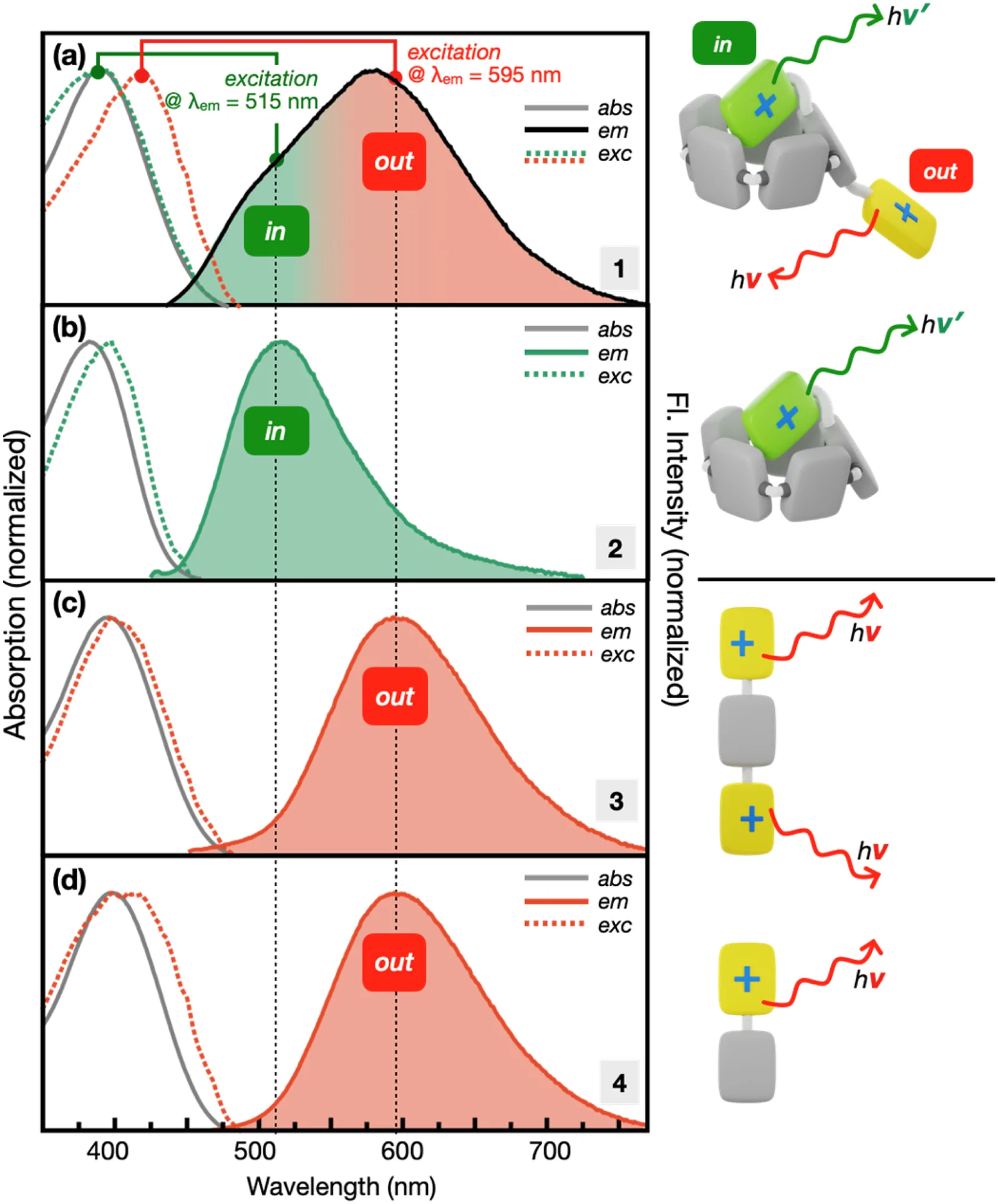
Normalized absorption, excitation, and emission spectra of 1–4 in MeOH at *T* = 293 K. Schematic representations of the corresponding structures are shown next to each spectrum.

To probe the origin of the peculiar dual emission of 1, excitation spectra were recorded at two different emission wavelengths, *λ*_em_ = 515 and 595 nm. These measurements revealed two distinct excitation profiles (Fig. 4a, dotted lines), ruling out typical dual emission scenarios involving either a single emitter with two emissive states or two electronically correlated fluorophores.^32,33^ Time-resolved fluorescence decay further supported the presence of two independent emissive species. When 1 was excited at *λ* = 375 nm, distinct decay components were observed for the emissions at *λ* = 485 and 620 nm (Table S3 and Fig. S2). These findings indicate that although the two ANIPY fluorophores in 1 are structurally identical, they behave differently.

To further define the role of the macrocyclic scaffold, we examined the emission properties of the other model compounds. Compound 2, having a single ANIPY group tethered to P5, showed a pronounced blue shift (*λ*_max,em_ = 515 nm) relative to macrocycle-free 4 (*λ*_max,em_ = 595 nm). As observed for compound 1, an intriguing “macrocyclic effect” emerges for 2 as well. The pillararene scaffold changes the photophysical behavior of the appended fluorophore. In contrast, the macrocycle-free models 3 and 4, having two and one ANIPY groups, respectively, exhibited a single emission band at 595 nm (Fig. 4c and d). The close match between these spectra and the long-wavelength emission component of 1 reinforces the assignment of this band to an ANIPY unit in a macrocycle-free-like environment. Taken together, these studies support that the dual emission of 1 arises from two nonequivalent ANIPY units residing in distinct environments (*vide infra*). This interpretation is further substantiated by deconvolution of the emission profile of 1 into two spectral components, each matching one of the two distinct emission profiles (Fig. S3 and Table S4).

### Molecular origin of dual emission

When comparing the spectral behavior of 2 and 4 (Fig. 4b and d), a question immediately arises: why does ANIPY emission undergo a dramatic blue shift when placed in proximity to the macrocycle? To answer this question, we carried out DFT computational studies on a model fluorophore, ANIPY-Me, with R = CH_3_ (Fig. 3 and Fig. S4; Tables S5 and S6). Since photoexcitation of ANIPY-Me primarily involves the HOMO-to-LUMO transition (Fig. S5a), we quantified the atomic coefficients of the frontier molecular orbitals (FMOs) and found that the HOMO and LUMO are localized on the aniline nitrogen atom and the pyridinium ring, respectively (Fig. S5b). During excitation from *S*_0,min_ to *S*_1,FC_, the computed dipole moment increases from 5.28 to 5.93 D, indicating charge separation in the excited state. The dipole moment further increases during relaxation to *S*_1,min_, consistent with stabilization of the separated charges in the excited state (Fig. S6).^34,35^

As shown in Fig. S7a, the emission spectra of ANIPY-Me undergo a bathochromic shift with increasing solvent polarity. This result underscores the solvent-polarity dependence characteristic of an intramolecular charge transfer (ICT) process, as supported by the DFT studies.^36^ In a polar environment, relaxation of the surrounding solvent stabilizes the charge-separated excited state, leading to long-wavelength emission. Therefore, when the ANIPY fluorophore is not tethered to the macrocycle (Fig. 4c and d) or resides away from it (Fig. 4a, *out*-position), long-wavelength emission is observed. In contrast, when ANIPY caps the nonpolar cavity of the macrocycle, polar solvent molecules cannot effectively stabilize the charge-separated excited state, and short-wavelength emission is produced (Fig. 4b).^37^ With one ANIPY unit located away from the macrocycle and the other approaching it, 1 displays both short- and long-wavelength emission (Fig. 4a). A mixture of macrocycle-free ANIPY-Me and unfunctionalized P5 in either CDCl_3_ or DMF, solvents in which both are soluble, provided no spectroscopic evidence of interaction (Fig. S8 and S9). Covalent tethering is thus central to the self-inclusion behavior observed for 1, although caution should be exercised when comparing results obtained in different solvent systems.

### Conformational control of dual emission by solvent polarity and temperature

The fluorescence spectra of the four compounds 1–4 (Fig. 4) demonstrate that mutual exclusion enables dual emission from two chemically identical fluorophores. Expanding on this idea, we investigated how the external stimuli can drive conformational switching of 1, as read out by dual emission (Fig. 5). As shown in the normalized absorption and emission spectra (Fig. 5a), the absorption profile of 1 is largely independent of solvent polarity, whereas its emission profile changes substantially. A new short-wavelength emission band emerges in polar solvents such as MeOH, in addition to the single emission band observed predominantly in nonpolar solvents such as CHCl_3_.

**Fig. 5:**
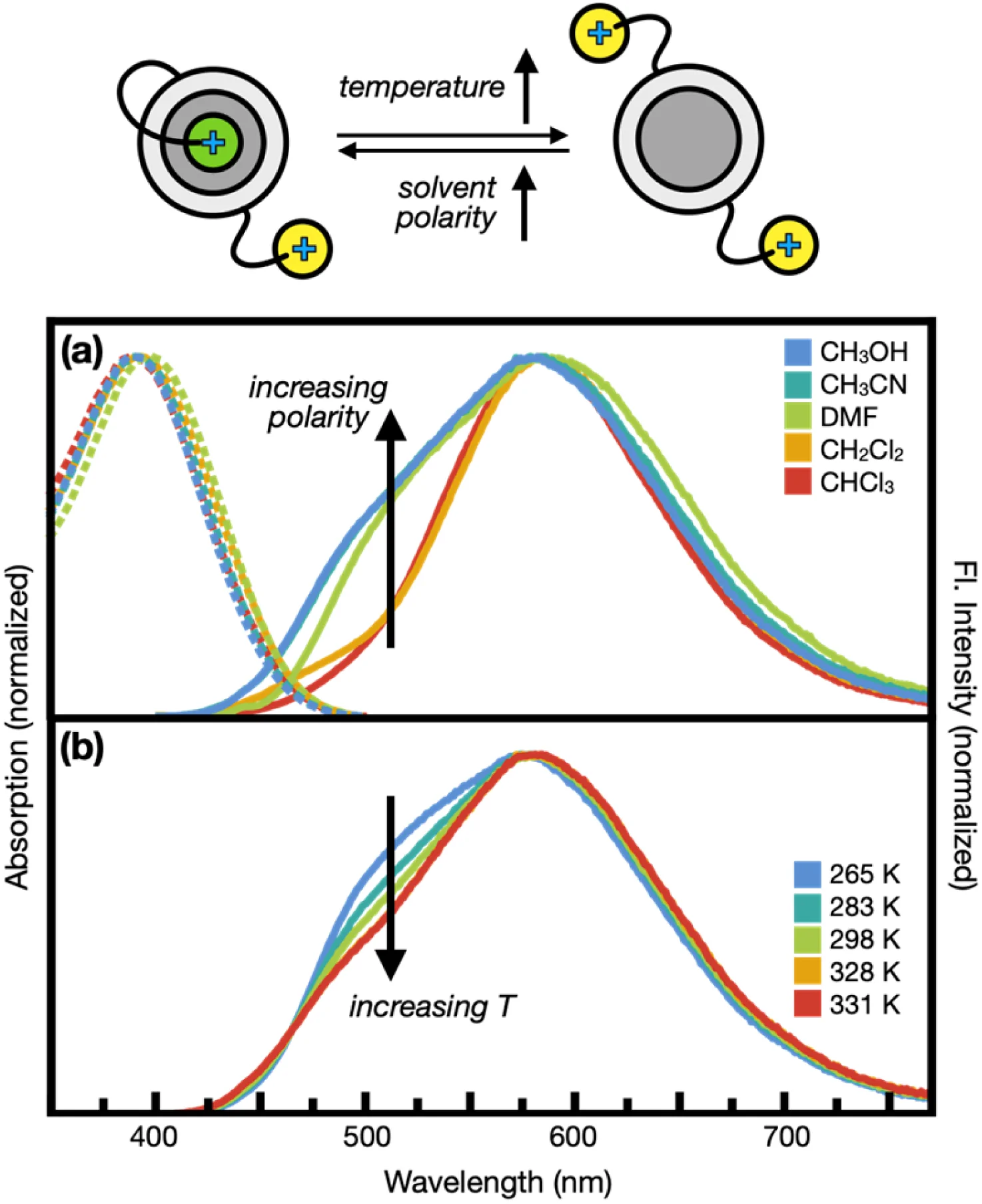
(a) Solvent-dependent (*T* = 293 K) and (b) temperature-dependent (in MeOH) absorption (dashed lines) and emission (solid lines) spectra of 1·Cl_2_ (0.1 mM), recorded with *λ*_exc_ = 390 nm. A schematic diagram of the two-state model for dual emission is shown above the spectra.

Control experiments established that the macrocycle-free model compounds 3 and 4 consistently produce a single emission band, with only a polarity-dependent spectral shift upon solvent variation (Fig. S7), but without the emergence of a new feature as observed in 1 (Fig. 5a). These results suggest the emergence of a new emissive species arising from a distinct conformer of 1 in polar solvents, rather than simply a shift in the emission energy of ANIPY. We postulate that as solvent polarity increases, the aromatic-rich ANIPY fragment slides toward the macrocyclic rim to minimize its solvent-exposed surface area, enhancing the contribution of the short-wavelength *in*-emission (Fig. 5). The mono-functionalized compound 2 exhibited a similar polarity-dependent response, with emission shifting toward shorter wavelengths as solvent polarity increased from DMF to MeOH (Fig. S10a).^38^

Temperature provides a second handle for conformational control of the dual emission in 1. While the overall emission intensity diminishes at higher temperatures (Fig. S11), the normalized spectra clearly reveal a disproportionately rapid decrease in the short-wavelength *in*-emission relative to the long-wavelength *out*-emission (Fig. 5b). This observation is consistent with a reduced fraction of intramolecular association at elevated temperatures. Increased thermal motion promotes bond rotation and vibration, favoring less restricted conformations in which the ANIPY unit is dissociated from the macrocycle. Upon lowering the temperature, the relative emission intensities changed reversibly (Fig. S12). Consistent with this interpretation, mono-functionalized compound 2 showed a red shift in the *in*-emission at higher temperature, indicating an increased contribution from the *out*-conformation, as observed for 1 (Fig. S10b).

### Nonpolar clustering by self-inclusion and mutual exclusion: energetic preferences

At this point, a general chemistry question arises. As a charged fluorophore, ANIPY in MeOH is expected to favor the polar exterior environment over the nonpolar P5 cavity. What, then, drives the self-inclusion manifested in Fig. 5? The answer is not simply a preference of ANIPY for a nonpolar cavity, but a balance of competing energetic terms. ANIPY has a cationic polycyclic π-skeleton, and P5 is a large macrocycle comprised of aromatic units. In a polar solvent such as MeOH, 1 would thus tend to reduce the solvent-exposed area of the extended π-surfaces while retaining favorable stabilization of its charged components. Self-inclusion provides one way to achieve this balance by clustering the nonpolar π-surfaces of ANIPY and P5 in a compact conformation.

To test this model, Connolly surface analysis was carried out using different probe radii (0.8–2.5 Å) for the DFT computational models of 1 (Fig. 6). As shown in Fig. 6b and S13, the solvent-accessible surface area of compact 1_*in*–*out*_ is smaller than that of extended 1_*out*–*out*_ (Fig. 6a). Molecular electrostatic potential (MEP) map analysis (Fig. S14) also confirmed that 1_*in*–*out*_ benefits from electrostatic interactions between the electron-rich pillararene base and the electron-deficient ANIPY pendants. The structural basis for ANIPY self-inclusion was investigated further by DFT computational studies. To simulate the seesaw motion (Fig. 2c), the *τ*_Ph–Ph_ value, defined as the dihedral angle between the rim skeleton of P5 and the *para*-substituted phenylene panel, was systematically varied to map the conformational energy landscape associated with swiveling of the methylene-linked panels. All other internal coordinates were allowed to relax, and the optimized *τ* values were measured and plotted (Fig. 6c). The 1_*in*–*out*_ conformer converges to an energy minimum at *τ* = 67° in MeOH, with the relative energy increasing as *τ* deviates in either direction. This result suggests that the flexible methylene bridges of P5 allow the phenylene panels to tilt, pushing one ANIPY unit outward by a seesaw-like motion and leaving it fully exposed to the bulk solvent environment.

**Fig. 6:**
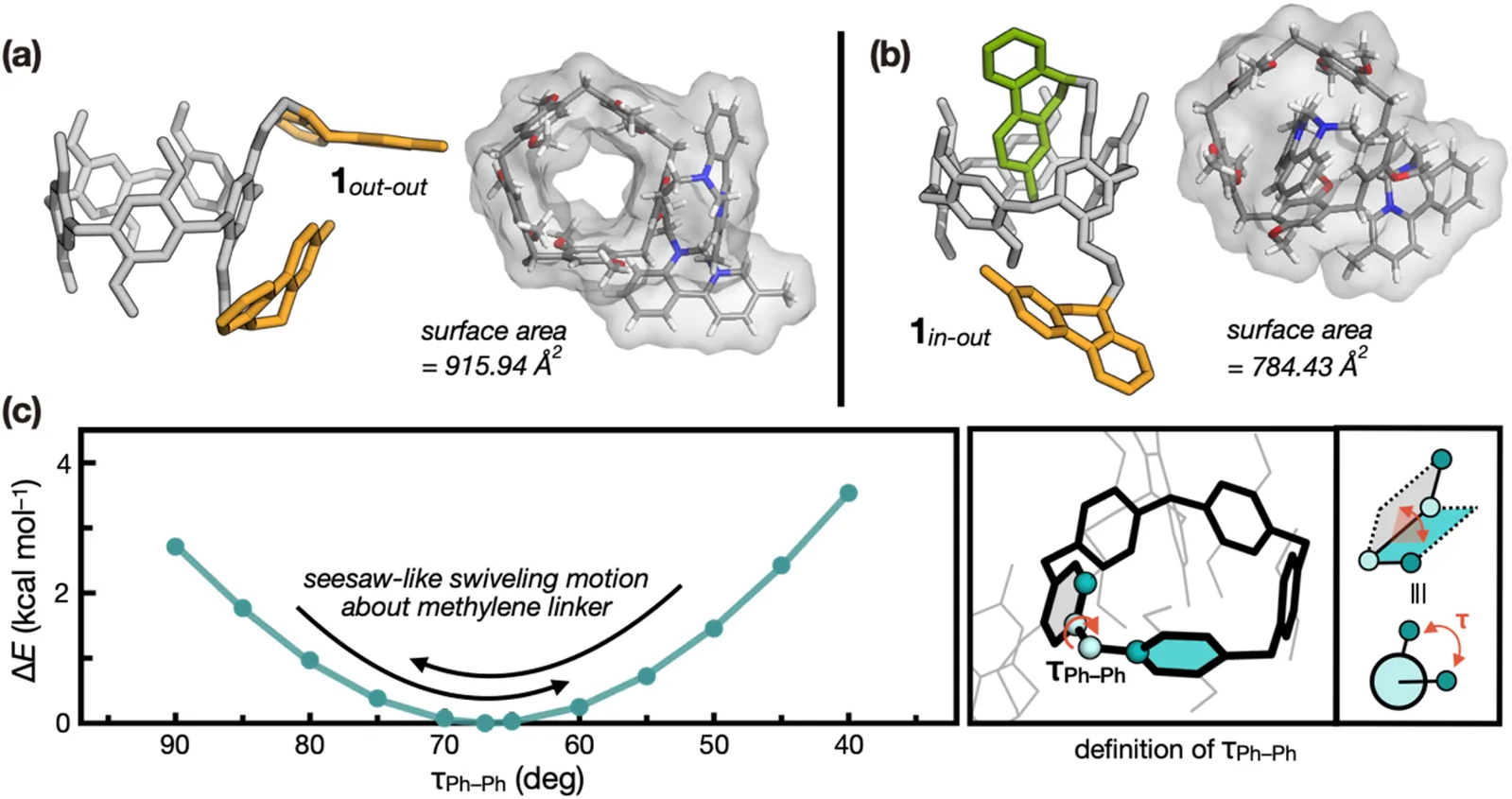
Energy-minimized DFT computational models of (a) 1_*out*–*out*_ and (b) 1_*in*–*out*_ conformations, calculated at the B3LYP-D3/6-31G** level with implicit solvation in MeOH, along with Connolly surface analysis. (c) Relative energy (Δ*E*) as a function of *τ*, defined in the right panel, calculated for the DFT model of 1.

To confirm the coupled nature of this motion, the dihedral angle on the opposite side of the same phenylene panel (*τ*_opposite_) was measured from each geometry-optimized structure along the scan. As *τ*_Ph–Ph_ decreases from 90° to 40°, *τ*_opposite_ increases monotonically from 117° to 130° (Fig. S15). This correlated movement arises from the structural rigidity of the phenylene panel: as a planar rigid body, tilting about one methylene linker necessarily opens the angle on the opposite side. The methylene bridges therefore function as mechanical pivots in a seesaw-like motion. The energy penalty upon deviation from *τ* = 67° reflects a restoring force that guides the system toward a well-defined docked geometry while preserving conformational flexibility.

### Conformational snapshots by ion mobility mass spectrometry

With the *in-vs.-out* conformational duality of 1 suggested by solution spectroscopy (Fig. 5) and DFT calculations (Fig. 6), we employed a structure-sensitive analytical technique to assess consistency with the proposed conformational model in the gas phase. Ion mobility spectrometry–mass spectrometry (IMS–MS) is well suited for this purpose because it separates gas-phase ions based on size and shape, allowing conformational isomers to be detected as distinct structural snapshots.^39–42^ As shown in Fig. 7a, IMS analysis of a MeOH solution sample of 1·Cl_2_ produced an ion signal at *m*/*z* = 584, assigned to dicationic 1. Two well-resolved peaks appeared in the arrival time distribution (ATD) at *t* = 44.0 and 57.5 ms, with collision cross-section (CCS) values of 360 and 470 Å^2^, respectively. In IMS, ions with smaller CCS values undergo fewer collisions with the buffer gas and thus drift more quickly, arriving at the detector earlier.^43,44^ The major signal at the shorter arrival time (*t* = 44.0 ms) is thus assigned to the more compact 1_*in*–*out*_ conformer, which is supported by the agreement between the experimentally determined CCS value of 360 Å^2^ and the computed value of 375 Å^2^ (Fig. S16).

**Fig. 7:**
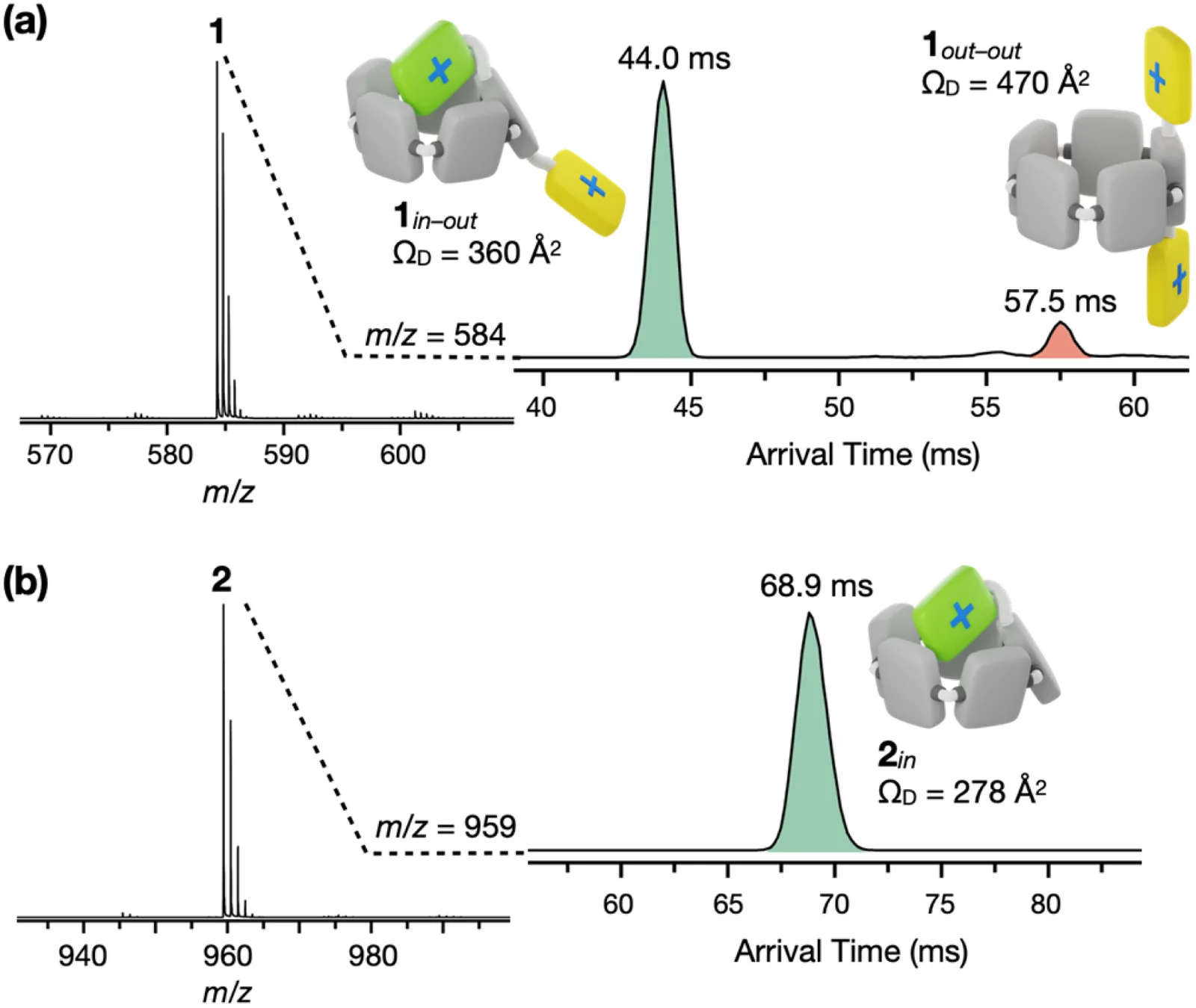
ESI-IMS–MS spectra of (a) 1 and (b) 2, showing mass-to-charge analysis (left) and ion mobility separation as arrival time distributions (right). Experimental collision cross-section values (*Ω*_D_) are provided for each resolved mobility peak.

The minor signal at the longer arrival time (*t* = 57.5 ms), accompanied by broad satellite peaks, is assigned to the more extended 1_*out*–*out*_ conformer (Fig. 6a).^45^ The discrepancy between the experimental (470 Å^2^) and computed (417 Å^2^) CCS values of 1_*out*–*out*_ may reflect conformational heterogeneity of the extended shape, which can sample a wider range of structures.^46^ After electrospray and desolvation, ions lose their counterions and must self-solvate to stabilize the charge.^47,48^ This process may lead to the coexistence of multiple 1_*out*–*out*_ geometries, observed as minor peaks at *t* = 52–57 ms adjacent to the main signal (*t* = 57.5 ms), rather than annealing into a single stable conformation.

Consistent with the single emission band in MeOH (Fig. 4b), compound 2, having one ANIPY pendant, produced a single peak in the ATD of the ion signal at *m*/*z* = 959, corresponding to the monocation (Fig. 7b). A good fit to a Gaussian function points to one predominant conformation (Fig. S17), and the experimentally determined CCS value (278 Å^2^) is consistent with the computed value (285 Å^2^) for the 2_*in*_ geometry, in which the ANIPY pendant is self-included toward the P5 rim (Fig. S18). On the 1–100 ms time scale of ion separation in drift-tube IMS–MS experiments, distinct conformers can be kinetically trapped and retain structural memory from the solution phase.^49^ The observed gas-phase geometries are thus consistent with solution-derived conformational preferences: 1 coexists as 1_*out*–*out*_ and 1_*in*–*out*_ conformers (Fig. 6), whereas 2 predominantly adopts a compact folded conformation (Fig. S18).

### Guest binding controls dual emission by conformational switching

As a rigid, pillar-shaped macrocyclic molecule, P5 is widely used as a supramolecular host for linear guest molecules such as dicyanoalkanes.^50–52^ Exploiting this host–guest chemistry, we used 1,2-dicyanoethane (1,2-DCE) to control the conformational equilibrium between the *in*–*out* and *out*–*out* forms of 1 (Fig. 2c). As schematically shown in Fig. 8a, binding of the guest should displace the ANIPY pendant, shifting the conformational equilibrium and changing the ratio of the dual emission intensities.

**Fig. 8:**
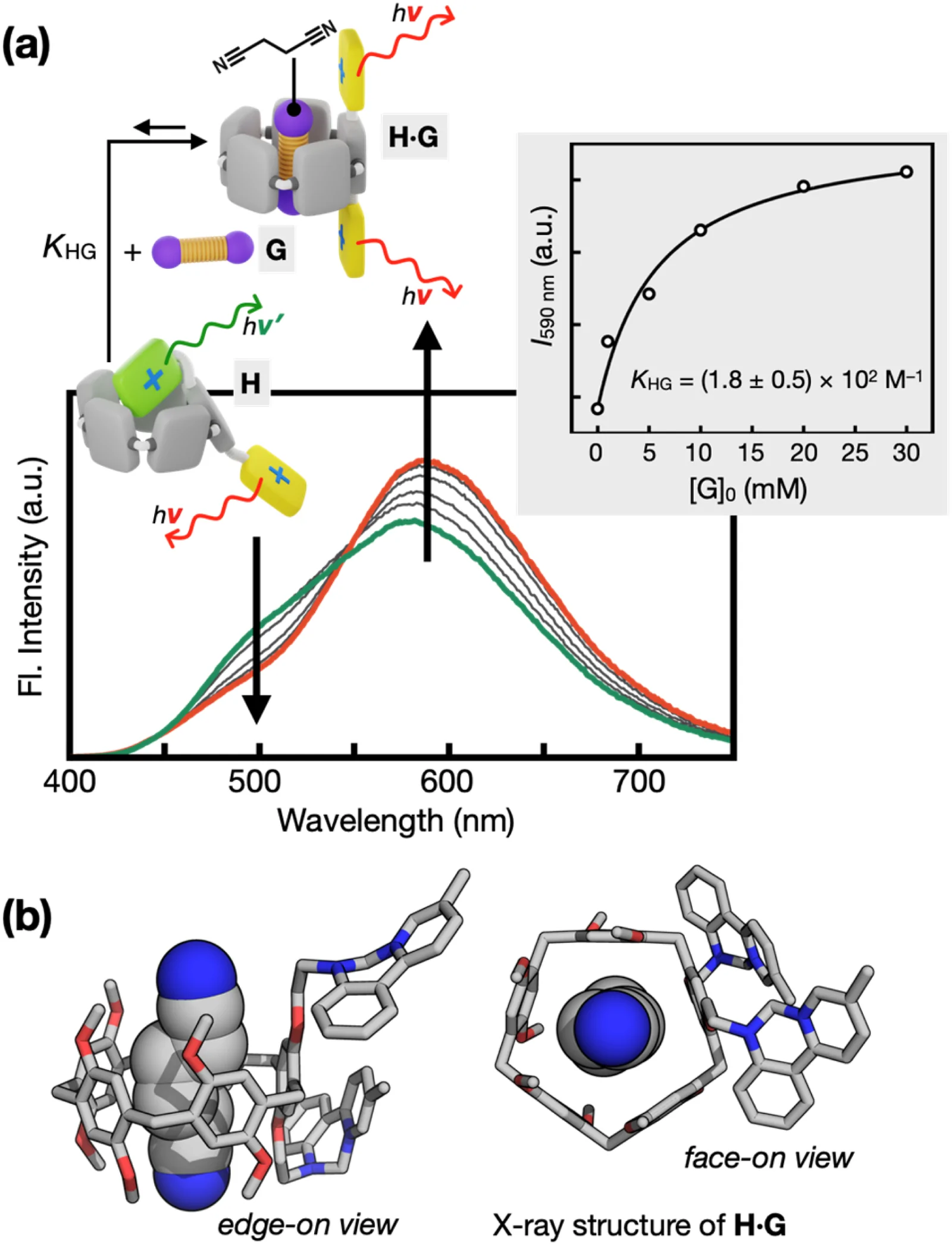
(a) Fluorescence titration (*λ*_exc_ = 390 nm) of host (H) 1 (0.1 mM in MeOH; *T* = 293 K) with the guest (G) 1,2-dicyanoethane, fitted to a 1 : 1 binding isotherm using changes in the fluorescence intensity at *λ* = 590 nm (*I*_590 nm_). (b) X-ray crystal structure of the host–guest complex represented by a capped-stick model for the host (1) and space-filling model for the guest (1,4-dicyanobutane).

To test this guest-induced switching, the fluorescence spectral changes of 1 were monitored in MeOH by titration with 1,2-DCE. As shown in Fig. 8a, guest addition decreased the emission intensity at 515 nm, accompanied by a concomitant increase in the emission at 595 nm. The sharply defined isoemissive point at *λ* = 545 nm suggests the presence of only two species, free host and host–guest complex, in equilibrium. Using the emission intensity at 590 nm, the titration data were fitted to a 1 : 1 binding isotherm with *K* = (1.8 ± 0.5) × 10^2^ M^−1^ (SI Note 1). Under these conditions, the UV-vis absorption spectra showed no appreciable shift upon guest addition (Fig. S19a). The broader applicability of guest-induced conformational switching was examined with an extended analogue, 1,4-dicyanobutane (1,4-DCB). Fluorescence titration of 1 with 1,4-DCB produced a similar spectral response with a binding constant of *K* = (2.0 ± 0.2) × 10^2^ M^−1^ (Fig. S20); the UV-vis absorption spectra showed no appreciable shift (Fig. S19b).

Structural evidence for guest encapsulation was obtained by X-ray crystallographic analysis of the 1⊃1,4-DCB inclusion complex. The crystal structure confirmed that the guest threads through the P5 cavity of 1 (Fig. 8b and Fig. S21), consistent with previously reported dicyanoalkane complexes of pillar[5]arene-based hosts.^53–55^ Computational studies using the independent gradient model based on Hirshfeld partition (IGMH) identified extensive van der Waals (vdW) contacts between the guest and the inner surface of the cavity (Fig. S22).

For mono-functionalized 2, both guests showed similar binding behavior. The fluorescence spectra underwent gradual red shifts upon guest binding (Fig. S23 and S24), while the UV-vis absorption spectra showed only modest changes (Fig. S25). The binding constants were determined to be *K* = (4.6 ± 0.3) × 10^2^ M^−1^ and *K* = (2.9 ± 0.4) × 10^2^ M^−1^ for 1,2-DCE and 1,4-DCB, respectively (Table S7). The higher binding affinity of 2 relative to 1, observed consistently for both guests, is in line with the mechanism outlined in Fig. 8a. In the bis-functionalized system 1, one ANIPY pendant occupies or partially blocks access to the P5 cavity in polar solvent. In contrast, guest binding to mono-functionalized 2 does not face such internal competition, thus allowing more efficient and tighter association.

Under similar conditions, the local site model 3, which lacks the macrocyclic moiety, showed no fluorescence response to 1,2-DCE (Fig. S26). Together, these findings are consistent with the important functional role of P5, which serves not only to bind the guest but also to partition the nonpolar “*in*” and polar “*out*” environments that give rise to dual fluorescence emission. Typical fluorescent chemical sensors rely on covalent or noncovalent modification of the fluorophore's electronic structure to induce spectral changes.^56,57^ In contrast, our system employs the analyte as a structural modulator that shifts the conformational equilibrium of the fluorogenic self-inclusion complex, thereby producing a quantifiable optical response.

### Linker-length-dependent mutual exclusion and dual emission

To test the working principles outlined in Fig. 2c, we systematically varied the alkyl linker, a key structural component of our system. In addition to the short ethylene-linked systems 1 and 2, analogues with longer butylene (1b) and hexylene (1h) linkers were prepared (Fig. 9; Scheme S1). In contrast to the behavior of 1 (Fig. 4a), both 1b and 1h exhibit a single emission band, while their absorption spectra remain largely unchanged across the series (Fig. 9a and b). This trend indicates that linker length does not significantly affect the ground-state electronic structure, but strongly biases the emissive conformation.

**Fig. 9:**
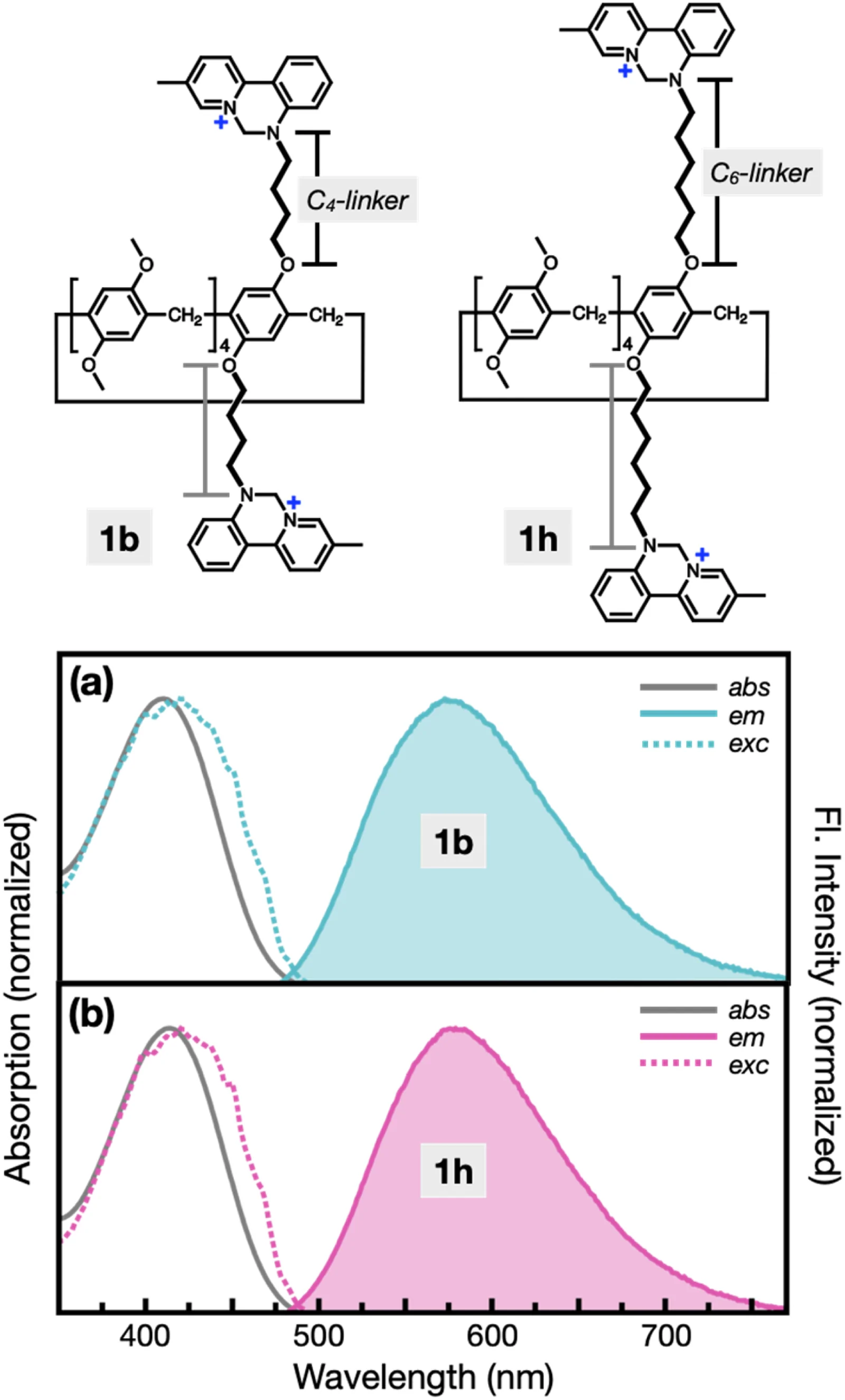
Chemical structures of the longer-linker derivatives (top), and normalized absorption, excitation, and emission spectra of (a) 1b and (b) 1h in MeOH at *T* = 293 K.

Excitation spectra recorded at different emission wavelengths, 515 nm and 595 nm, as well as the emission maxima of 1b (*λ* = 575 nm) and 1h (*λ* = 580 nm), are essentially superimposable (Fig. S27). This observation indicates that the two fluorophores in the longer-linker systems are spectroscopically indistinguishable and behave as one dominant emissive species (Fig. S28). Consistent with this assignment, the emission maxima of the bis-functionalized systems closely match those of their mono-functionalized analogues, 2b and 2h (Scheme S1 and Fig. S29).

The same single-state behavior persists under external perturbation. Across the longer-linker series (*i.e.*, butylene and hexylene derivatives 1b, 1h, 2b, and 2h), changes in solvent polarity induce only gradual spectral shifts, without the emergence of new emission features (Fig. S30). This behavior is consistent with a conventional solvatochromic response from a single emissive state, as observed for the model fluorophore ANIPY-Me (Fig. S7a). Likewise, temperature-dependent measurements showed no apparent changes in spectral shape (Fig. S31). This behavior stands in sharp contrast to 1, where temperature changes shift the relative populations of two distinct emissive states (Fig. 5b). Guest titration studies pointed toward the same conclusion. While correlated intensity changes were observed for the distinct emission bands of 1, the longer-linker systems exhibited only gradual fluorescence quenching and bathochromic shifts upon guest addition (Fig. S32–S35), without appreciable shifts in the UV-vis absorption spectra (Fig. S36–S39). This behavior cannot be described by the two-state model of 1 (Fig. 2c).

Taken together, these results suggest that dual emission in 1 depends critically on the short ethylene linker, which enforces the positional asymmetry needed for mutual exclusion. When the linker is extended, this geometric constraint is relaxed, and the two ANIPY pendants no longer occupy distinct emissive environments. The short linker of 1 also promotes self-inclusion by maintaining effective proximity between the appended fluorophore and the P5 cavity, supported by favorable enthalpic stabilization. In contrast, linker elongation increases conformational flexibility and attenuates this proximity effect, increasing not only the entropic penalty but also the energetic cost and conformational requirements for adopting the folded state, which becomes increasingly strained relative to the ideal staggered conformation as the linker is extended (Fig. S40 and S41). As a consequence, access to dual *in*-and-*out* emissive states is suppressed in the butylene- and hexylene-linked systems.

To probe the structural characteristics of the longer-linker analogues, IMS–MS analysis was also conducted on 1b and 1h (Fig. S42 and S43). 1h displayed a major peak (*t* = 43.1 ms) accompanied by a minor satellite peak (*t* = 41.9 ms). The CCS value of the major peak (399 Å^2^) closely matched the value calculated for a structure with both ANIPY pendants positioned outside the cavity (394 Å^2^), consistent with its spectroscopically indistinguishable behavior (Fig. S44). The minor peak likely reflects structural heterogeneity arising from extended conformations accessed by the long hexylene linker. By contrast, 1b exhibited a single peak (*t* = 46.3 ms), with a CCS value (377 Å^2^) in agreement with that calculated for a structure with one ANIPY pendant positioned near the cavity (364 Å^2^; Fig. S45). This result suggests a weak tendency toward partial folding in the butylene-linked system. However, the single emission band observed in solution indicates that this conformational bias is not sufficient to generate distinct *in*-and-*out* emissive environments. Thus, 1b represents an intermediate case between the well-defined mutual exclusion of 1 and the more flexible hexylene-linked system. Overall, the IMS–MS analysis highlights the critical role of linker length in governing conformational dynamics and spectroscopic output, establishing a structural basis for mutual exclusion and stimuli-responsive dual emission.

## Conclusions

Molecules can gain function by integrating distinct states into a single covalent framework. In this study, we translated this idea into a macrocyclic platform featuring two identical fluorophores constrained to occupy different molecular spaces: one capping the nonpolar cavity and the other exposed to the polar medium. This built-in asymmetry arises from self-inclusion and mutual exclusion, which transform chemically identical units into functionally nonequivalent reporters and produce dual fluorescence for spatially resolved microenvironment readout. The emission profile of this molecular device responds to solvent polarity, temperature, and guest binding, which demonstrates that spatial heterogeneity can be modulated by intramolecular conformational reorganization. Linker variation further showed that this behavior depends critically on tether length, which enforces the proximity and geometric constraint required for mutual exclusion. These findings suggest a simple design principle: molecular architecture can make identical functional units behave differently by controlling their placement within macrocycle-defined environments.

## Author contributions

Conceptualization: S. H. and D. L.; data curation: S. H., D. K., D. C., and E. C.; formal analysis: S. H., D. K., D. C., and E. C.; investigation: S. H., D. K., D. C., and E. C.; methodology: S. H. and D. L.; visualization: S. H. and D. L.; supervision: J. S. and D. L.; validation: J. S.; funding acquisition: D. L.; writing – original draft: S. H. and D. L.; writing – review & editing: S. H., D. K., D. C., E. C., J. S., and D. L.

## Conflicts of interest

There are no conflicts to declare.

## Supplementary Material

SC-OLF-D6SC06062E-s001

## Data Availability

CCDC 2432639 (1·Cl_2_), 2432640 (2·Cl), and 2552442 ([1⊃1,4-DCB]·Cl_2_) contain the supplementary crystallographic data for this paper.^58*a*–*c*^ The data supporting this article have been included as part of the supplementary information (SI). Ref. 59–89 are cited in the SI. Supplementary information: experimental procedures, NMR spectra, supplementary figures, supplementary note, and supplementary tables. See DOI: https://doi.org/10.1039/d6sc06062e.
