# Supplementary material for: Mechanical macrocyclic effects: stimuli-responsive dual emission by self-inclusion and mutual exclusion
Source: Chem Sci. 2026 Oct 2. Online ahead of print. doi: 10.1039/d6sc06062e (PMC13656219; doi:10.1039/d6sc06062e)
Supplement: Supplementary file 1 [file SC-OLF-D6SC06062E-s001.pdf]

Supplementary Information (SI) for Chemical Science.
This journal is © The Royal Society of Chemistry

Supplementary Information

Mechanical macrocyclic effects: stimuli-responsive dual emission by self-inclusion and mutual exclusion

Seungyeon Hyun,[a] Doui Kim,[b] Dongjin Choi,[b] Eunkyeong Choi,[a] Jongcheol Seo,[b,c] and Dongwhan Lee[a,*]

[a] *Department of Chemistry, Seoul National University, 1 Gwanak-ro, Gwanak-gu, Seoul 08826, Korea*
[b] *Department of Chemistry, Pohang University of Science and Technology (POSTECH), 77 Cheongam-ro, Pohang, Gyeongsangbuk-do 37673, Korea*
[c] *School of Interdisciplinary Bioscience and Bioengineering (I-BIO), Pohang University of Science and Technology (POSTECH), 77 Cheongam-ro, Pohang, Gyeongsangbuk-do 37673, Korea*

Table of Contents

1. Experimental Section S-3

2. Synthetic Procedures S-7

3. SC-XRD Experiments S-15

4. Validation Reply Forms S-17

5. Supplementary Tables S-18

6. Supplementary Note S-23

7. Supplementary Figures S-25

8. Cartesian Coordinates of DFT Computational Models S-87

1. Experimental Section

General Considerations. All reagents were obtained from commercial suppliers and used as received unless otherwise noted. The solvents THF was saturated with argon and purified by passage through activated Al_2O_3 columns under argon (Innovative Technology SPS PureSolv MD4). All air-sensitive manipulations were carried out under argon atmosphere by standard Schlenk-line techniques. The compounds 2-(5-methylpyridin-2-yl)aniline (**6**),[1] *N*-Methyl-2-(5-methylpyridin-2-yl)aniline (**ANIPY-Me**·Cl),[2] 1^2,1^5-bis(2-bromoethoxy)-3^2,3^5,5^2,5^5,7^2,7^5,9^2,9^5-octamethoxy-1,3,5,7,9(1,4)-pentabenzenacyclodecaphane (**5**),[3] 1^2,1^5-bis(4-bromobutoxy)-3^2,3^5,5^2,5^5,7^2,7^5,9^2,9^5-octamethoxy-1,3,5,7,9(1,4)-pentabenzenacyclodecaphane (**5b**),[4] and 1^2,1^5-bis((6-bromohexyl)oxy)-3^2,3^5,5^2,5^5,7^2,7^5,9^2,9^5-octamethoxy-1,3,5,7,9(1,4)-pentabenzenacyclodecaphane (**5h**),[5] 1^2-(2-bromoethoxy)-1^5,3^2,3^5,5^2,5^5,7^2,7^5,9^2,9^5-nonamethoxy-1,3,5,7,9(1,4)-pentabenzenacyclodecaphane (**8**),[6] 1^2-(4-bromobutoxy)-1^5,3^2,3^5,5^2,5^5,7^2,7^5,9^2,9^5-nonamethoxy-1,3,5,7,9(1,4)-pentabenzenacyclodecaphane (**8b**),[5] 1^2-((6-bromohexyl)oxy)-1^5,3^2,3^5,5^2,5^5,7^2,7^5,9^2,9^5-nonamethoxy-1,3,5,7,9(1,4)-pentabenzenacyclodecaphane (**8h**),[7] 1,4-bis(2-bromoethoxy)benzene (**10**)[8] and 1-(2-bromoethoxy)-4-methoxybenzene (**12**),[9] were synthesized according to reported procedures.

[1] H. Park, H. M. Ahn, H. Y. Jeong, C. Kim and D. Lee, *Chem. – Eur. J.*, 2018, **24**, 8632–8638.

[2] S. Kim, J. Kim and D. Lee, *Angew. Chem. Int. Ed.*, 2021, **60**, 10858–10864.

[3] X.-Y. Hu, P. Zhang, X. Wu, W. Xia, T. Xiao, J. Jiang, C. Lin and L. Wang, *Polym. Chem.*, 2012, **3**, 3060–3063.

[4] Q. Lin, Y.-Q. Fan, P.-P. Mao, L. Liu, J. Liu, Y.-M. Zhang, H. Yao and T.-B. Wei, *Chem. – Eur. J.*, 2018, **24**, 777–783.

[5] J. Li, J. Dong, H. Wang, X. Yang, J. Chen, J. Gu and S. Lin, *Adv. Mater. Interfaces*, 2021, **8**, 2101627.

[6] S. Sun, X.-Y. Hu, D. Chen, J. Shi, Y. Dong, C. Lin, Y. Pan and L. Wang, *Polym. Chem.*, 2013, **4**, 2224–2229.

[7] Y.-M. Zhang, Q.-Y. Yang, X.-Q. Ma, H.-Q. Dong, Y.-F. Zhang, W.-L. Guan, H. Yao, T.-B. Wei and Q. Lin, *J. Phys. Chem. A*, 2020, **124**, 9811–9817.

[8] E. H. Wanderlind, D. G. Liz, A. P. Gerola, R. F. Affeldt, V. Nascimento, L. C. Bretanha, R. Montecinos, L. Garcia-Rio, H. D. Fiedler and F. Nome, *ACS Catal.*, 2018, **8**, 3343–3347.

[9] P. Guo, Q. Chen, T. Liu, L. Xu, Q. Yang and X. Qian, *ACS Med. Chem. Lett.*, 2013, **4**, 527–531.

Physical Measurements. ^{1}H NMR and ^{13}C NMR spectra were recorded on a 400 MHz Agilent 400-MR DD2 Magnetic Resonance System, a 500 MHz Varian/Oxford As-500 spectrometer, a 500 MHz Bruker Ascend™ 500 spectrometer, or a 500 MHz Bruker Ascend Evo 500 spectrometer. Chemical shifts were referenced to the residual solvent peaks.latex [10] High-resolution electrospray ionization (ESI) mass spectra were obtained on a Thermo Scientific Orbitrap Exploris 120 mass spectrometer. FT-IR spectra were recorded on a PerkinElmer Spectrum Two FT-IR spectrometer. UV–vis spectra were recorded on an Agilent 8453 UV–vis spectrophotometer with ChemStation software. Fluorescence spectra were recorded on a Photon Technology International Quanta-Master 400 Spectrofluorometer with FelixGX software. Absolute quantum yields were determined by using an integrating sphere attached to the fluorimeter. Time-resolved photoluminescence (PL) decay measurements were carried out with an Edinburgh FS5-TCSPC equipped with a 375 nm diode laser (EPL-375).

[10] G. R. Fulmer, A. J. M. Miller, N. H. Sherden, H. E. Gottlieb, A. Nudelman, B. M. Stoltz, J. E. Bercaw and K. I. Goldberg, *Organometallics*, 2010, **29**, 2176–2179.

Ion Mobility Mass Spectrometry. Experiments were conducted using a hybrid drift tube quadrupole time-of-flight instrument (6560 IM-QTOF, Agilent Technologies Inc., Santa Clara, CA) equipped with an electrospray ionization source. Sample solutions were prepared by dissolving each compound in MeOH (100 μM). The sample solution was delivered to the standard ESI emitter (tip opening i.d. ~150 μm) at a flow rate of 15 μL min^{-1} and electrosprayed under a 2–3 kV voltage offset using N_2 nebulizing gas for the positive ion modes. The ions were collected in the entrance ion funnel and pulsed into the 80 cm-long drift tube filled with a nitrogen buffer gas (~3.945 Torr). The ions were separated by their CCSs and charges while they traveled through the drift tube under the influence of a constant electric field (9–15 V·cm^{-1}). The separated ions were subsequently focused in an exit ion funnel and transferred into the high-vacuum region, where the time-of-flight (TOF) mass spectra were recorded for IM-separated ions every 250 μs.

Determination of Collision Cross-Section Value. The experimental CCS value of a specific m/z ion was determined using the stepped-field method,[11] in which the arrival time (t_D) was measured at varying drift voltages (V_d) across the drift tube. The arrival time t_D was calculated as the sum of the ion mobility-derived drift time (t_d) and the time spent after the ion mobility spectrometer (t_0), as shown in eqn (1) and (2).

$$t_d = \frac{L}{v_d} = \frac{L^2}{KV_d} \tag{1}$$

$$t_D = t_d + t_0 = \frac{L^2}{K} \cdot \frac{1}{V_d} + t_0 \tag{2}$$

Plotting t_D as a function of $1/V_d$, followed by linear regression using eqn (2), provides the mobility (K) of the analyte ion. This mobility can then be converted into the CCS value using the Mason–Schamp equation (eqn (3)), which incorporates parameters such as standard pressure (P_0), temperature (T_0), number density of buffer gas molecules (N_0), ion charge state (z), elementary charge (e, 1.602 × 10^{-19} C), reduced mass (μ), and the Boltzmann constant (k_B).

$$\Omega = \frac{3ze}{16N_0}\left(\frac{2\pi}{\mu k_B T}\right)^{\frac{1}{2}} \frac{P_0}{P} \cdot \frac{T}{T_0} \cdot \frac{1}{K} \tag{3}$$

[11] S. M. Stow, T. J. Causon, X. Zheng, R. T. Kurulugama, T. Mairinger, J. C. May, E. E. Rennie, E. S. Baker, R. D. Smith, J. A. McLean, S. Hann and J. C. Fjeldsted, *Anal. Chem.*, 2017, **89**, 9048–9055.

Computational Details. Global optimizations were performed using the Global Optimization Algorithm (GOAT)[12] algorithm with ORCA 6.1.1 program[13] at the semiempirical level (GFN2-xTB)[14] of theory. Solvation was considered using the analytical linearized Poisson–Boltzmann (ALPB)[15] implicit solvation model. The global minimum structure identified from this conformer search was selected as the initial structure for subsequent DFT calculations. All density functional theory (DFT) and time-dependent density functional theory (TD-DFT) calculations were performed with Gaussian 16 program suite.[16] Ground-state and excited-state geometry optimizations were carried out with DFT and TD-DFT calculations using B3LYP hybrid functional with Grimme's D3 dispersion correction (B3LYP-D3)[17] with 6-31G** and 6-311++G** basis sets. Vibrational frequency calculations were performed to confirm that all optimized geometries converged to minima or saddle points for transition states. Zero-point and thermal corrections were made to the electronic energies to obtain Gibbs free energies of the optimized geometries. Solvation was considered by self-consistent reaction field (SCRF) calculations with conductor-like polarizable continuum model (CPCM) of MeOH ($\varepsilon = 32.6$).[18] Independent gradient model analysis[19] based on Hirshfeld partition of molecular density (IGMH)[20] were performed using Multiwfn 3.8 software.[21] Theoretical CCS values under nitrogen buffer gas were predicted using the trajectory method implemented in the iMoS program.[22] The trajectory method used was TMLJ (Trajectory Method with Lennard-Jones 4–6–12 potential), including a quadrupole potential term to account for ion–neutral interactions.

[12] B. de Souza, *Angew. Chem. Int. Ed.*, 2025, **64**, e202500393.

[13] F. Neese, *Wiley Interdiscip. Rev.: Comput. Mol. Sci.*, 2025, **15**, e70019.

[14] C. Bannwarth, S. Ehlert and S. Grimme, *J. Chem. Theory Comput.*, 2019, **15**, 1652–1671.

[15] S. Ehlert, M. Stahn, S. Spicher and S. Grimme, *J. Chem. Theory Comput.*, 2021, **17**, 4250–4261.

[16] M. J. Frisch, G. W. Trucks, H. B. Schlegel, G. E. Scuseria, M. A. Robb, J. R. Cheeseman, G. Scalmani, V. Barone, G. A. Petersson, H. Nakatsuji, X. Li, M. Caricato, A. V. Marenich, J. Bloino, B. G. Janesko, R. Gomperts, B. Mennucci, H. P. Hratchian, J. V. Ortiz, A. F. Izmaylov, J. L. Sonnenberg, D. Williams-Young, F. Ding, F. Lipparini, F. Egidi, J. Goings, B. Peng, A. Petrone, T. Henderson, D. Ranasinghe, V. G. Zakrzewski, J. Gao, N. Rega, G. Zheng, W. Liang, M. Hada, M. Ehara, K. Toyota, R. Fukuda, J. Hasegawa, M. Ishida, T. Nakajima, Y. Honda, O. Kitao, H. Nakai, T. Vreven, K. Throssell, J. A. Montgomery, Jr., J. E. Peralta, F. Ogliaro, M. J. Bearpark, J. J. Heyd, E. N. Brothers, K. N. Kudin, V. N. Staroverov, T. A. Keith, R. Kobayashi, J. Normand, K. Raghavachari, A. P. Rendell, J. C. Burant, S. S. Iyengar, J. Tomasi, M. Cossi, J. M. Millam, M. Klene, C. Adamo, R. Cammi, J. W. Ochterski, R. L. Martin, K. Morokuma, O. Farkas, J. B. Foresman and D. J. Fox, *Gaussian 16* (Revision C.01), Gaussian, Inc., Wallingford, CT, 2016.

[17] S. Grimme, J. Antony, S. Ehrlich and H. Krieg, *J. Chem. Phys.*, 2010, **132**, 154104.

[18] J. Tomasi, B. Mennucci and R. Cammi, *Chem. Rev.*, 2005, **105**, 2999–3094.

[19] C. Lefebvre, H. Khartabil, J.-C. Boisson, J. Contreras-García, J.-P. Piquemal and E. Hénon, *ChemPhysChem*, 2018, **19**, 724–735.

[20] T. Lu and Q. Chen, *J. Comput. Chem.*, 2022, **43**, 539–555.

[21] T. Lu and F. Chen, *J. Comput. Chem.*, 2012, **33**, 580–592.

[22] V. Shrivastav, M. Nahin, C. J. Hogan and C. Larriba-Andaluz, *J. Am. Soc. Mass. Spectrom.*, 2017, **28**, 1540–1551.

2. Synthetic Procedures

Scheme S1. Synthetic routes.

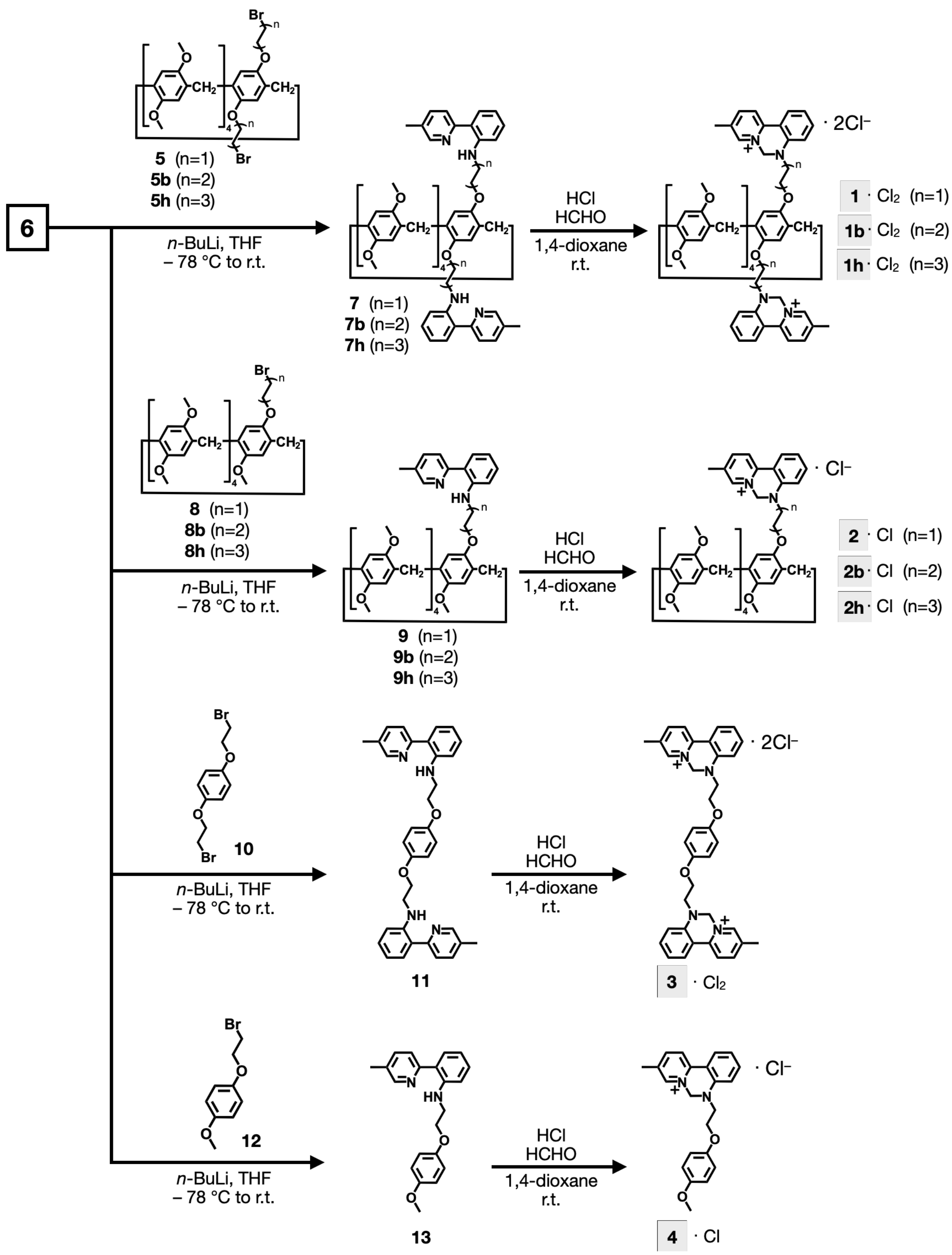

***N*,*N*′-(((3^{2},3^{5},5^{2},5^{5},7^{2},7^{5},9^{2},9^{5}-Octamethoxy-1,3,5,7,9(1,4)-pentabenzenacyclodecaphane-1^{2},1^{5}-diyl)bis(oxy))bis(ethane-2,1-diyl))bis(2-(5-methylpyridin-2-yl)aniline) (7).** An oven-dried 50-mL Schlenk flask was charged with **6** (328 mg, 1.78 mmol) and dry THF (6 mL) under an Ar atmosphere. The solution was cooled to –78 ℃, and *n*-butyllithium (1.1 mL, 1.6 M solution in hexanes, 1.8 mmol) was added dropwise via syringe over a period of 1 min. After 20 min, a solution of **5** (708 mg, 0.756 mmol) in dry THF (4 mL) was added dropwise over a period of 5 min. The reaction mixture was warmed to r.t., stirred for 2 d, quenched by adding water (15 mL), and extracted into CH_2Cl_2 (70 mL × 3). The combined extracts were dried over anhyd Na_2SO_4, filtered, and concentrated under reduced pressure. Flash column chromatography on SiO_2 (hexane:CH_2Cl_2:EtOAc = 2:1:0 to 4:2:1, v/v) furnished **7** as a yellow solid (350 mg, 0.306 mmol, yield = 34%). ^{1}H NMR (500 MHz, CD_2Cl_2, 298 K): δ 8.64 (t, J = 4.9 Hz, 2H), 8.36 (s, 2H), 7.58 (s, 3H), 7.54 (d, J = 7.8 Hz, 2H), 7.25 (t, J = 7.7 Hz, 2H), 6.94 (s, 2H), 6.89–6.81 (m, 11H), 6.74 (t, J = 7.4 Hz, 2H), 4.20–4.15 (m, 2H) 4.11 (q, J = 9.2, 7.8 Hz, 2H), 3.86 (s, 1H), 3.84 (s, 1H) 3.77–3.68 (m, 26H), 3.65 (q, J = 5.3 Hz, 4H), 3.58 (s, 6H), 2.29 (s, 6H). ^{13}C NMR (126 MHz, CD_2Cl_2, 298 K): δ 157.3, 150.9, 150.85, 150.79, 150.1, 148.3, 148.2, 138.12, 131.08, 130.4, 129.8, 129.0, 128.74, 128.67, 128.6, 122.5, 122.3, 116.5, 113.9, 113.8, 113.7, 111.8, 67.4, 56.1, 55.98, 55.97, 55.94, 55.86, 43.8, 29.62, 29.57, 29.55, 18.4. FT-IR (ATR, cm^{-1}): 2934, 2827, 1605, 1581, 1498, 1465, 1446, 1399, 1327, 1307, 1266, 1211, 1092, 1046, 994, 953, 882, 854, 830, 772, 747, 709, 677, 649, 606, 592, 563, 538, 520, 498, 486, 473, 461, 450, 441, 431, 418, 406. HRMS (ESI) calcd for $C_{71}H_{74}N_4O_{10}$ $[M + H]^+$ 1143.5491, found 1143.5478.

5,5′-(((3^{2},3^{5},5^{2},5^{5},7^{2},7^{5},9^{2},9^{5}-Octamethoxy-1,3,5,7,9(1,4)-pentabenzenacyclodecaphane-1^{2},1^{5}-diyl)bis(oxy))bis(ethane-2,1-diyl))bis(9-methyl-5,6-dihydropyrido[1,2-*c*]quinazolin-7-ium) chloride (1·Cl_2). To a dry 1,4-dioxane solution (14 mL) of **7** (117 mg, 0.102 mmol) was added formaldehyde (0.09 mL, 37 wt% solution in H_2O, 1 mmol) under an Ar atmosphere. After 5 min, HCl (0.05 mL, 4 M solution in dioxane, 0.2 mmol) was added dropwise. The reaction mixture was stirred at r.t. for 2 h to furnish a yellow solid material, which was isolated by filtration, and washed with 1,4-dioxane and Et_2O to afford **1**·Cl_2 as a yellow solid (114 mg, 0.0917 mmol, yield = 92%). ^{1}H NMR (500 MHz, CD_2Cl_2, 298 K): δ 8.87 (s, 2H), 7.91 (d, J = 7.5 Hz, 2H), 7.77 (d, J = 7.5 Hz, 2H), 7.61 (t, J = 7.4 Hz, 2H), 7.41 (d, J = 8.3 Hz, 4H), 7.21 (t, J = 7.6 Hz, 2H), 6.89 (s, 2H), 6.78 (s, 2H), 6.72 (s, 2H), 6.62 (s, 1H), 6.47 (s, 2H), 6.29 (s, 2H), 5.79 (d, J = 4.6 Hz, 5H) 3.69 (m, 15H), 3.63 (s, 16H), 3.28–3.13 (m, 4H). ^{13}C NMR (126 MHz, CD_2Cl_2, 298 K): δ 151.0, 150.9, 149.5, 146.8, 145.9, 143.5, 141.8, 136.7, 135.1, 129.3, 129.1, 128.7, 126.7, 123.2, 122.3, 119.2, 118.8, 115.5, 114.5, 114.3, 114.1, 69.1, 68.0, 67.5, 56.5, 56.4, 56.2, 29.9, 29.5, 29.3, 17.6. FT-IR (ATR, cm^{-1}): 2934, 1630, 1608, 1524, 1498, 1464, 1398, 1275, 1210, 1119, 1042, 951, 872, 761, 704, 672, 650, 616, 605, 580, 563, 538, 519, 498, 486, 473, 461, 451, 441, 431, 418, 406. HRMS (ESI) calcd for $C_{73}H_{76}N_4O_{10}Cl$ $[M – 2Cl]^{2+}$ 584.2775, found 584.2770.

***N*,*N*′-(((3^{2},3^{5},5^{2},5^{5},7^{2},7^{5},9^{2},9^{5}-Octamethoxy-1,3,5,7,9(1,4)-pentabenzenacyclodecaphane-1^{2},1^{5}-diyl)bis(oxy))bis(butane-4,1-diyl))bis(2-(5-methylpyridin-2-yl)aniline) (7b).** An oven-dried 25-mL Schlenk flask was charged with **6** (138 mg, 0.748 mmol) and dry THF (3.2 mL) under an Ar atmosphere. The solution was cooled to –78 ℃, and *n*-butyllithium (0.47 mL, 1.6 M solution in hexanes, 0.75 mmol) was added dropwise via syringe over a period of 1 min. After 20 min, a solution of **5b** (372 mg, 0.374 mmol) in dry THF (1 mL) was added dropwise over a period of 5 min. The reaction mixture was warmed to r.t., stirred for 1 d, quenched by adding water (10 mL), and extracted into CH_2Cl_2 (50 mL × 3). The combined extracts were dried over anhyd Na_2SO_4, filtered, and concentrated under reduced pressure. Flash column chromatography on SiO_2

(hexane:CH_2Cl_2:EtOAc = 2:1:0 to 10:2:1, v/v) furnished **7b** as a white solid (233 mg, 0.194 mmol, yield = 52%). ^{1}H NMR (500 MHz, CD_2Cl_2, 298 K): δ 8.38–8.36 (m, 4H), 7.62–7.57 (m, 4H), 7.55 (dd, J = 7.8, 1.6 Hz, 2H), 7.24 (ddd, J = 8.6, 7.2, 1.7 Hz, 2H), 6.90 (s, 2H), 6.87–6.85 (m, 8H), 6.75 (d, J = 8.4 Hz, 2H), 6.71–6.68 (m, 2H), 4.00–3.96 (m, 2H), 3.95–3.91 (m, 2H), 3.75–3.70 (m, 28H), 3.64 (s, 6H), 3.31 (q, J = 5.9 Hz, 4H), 2.31 (s, 6H), 2.02–1.95 (m, 8H). ^{13}C NMR (126 MHz, CD_2Cl_2, 298 K): δ 157.5, 151.01, 150.98, 150.9, 150.3, 148.5, 148.2, 138.1, 131.0, 130.5, 129.6, 128.92, 128.88, 128.86, 128.7, 122.1, 121.7, 115.9, 114.7, 114.0, 113.9, 113.8, 111.6, 68.6, 56.05, 55.98, 43.8, 29.8, 29.7, 29.5, 28.4, 27.0, 18.4. FT-IR (ATR, cm^{-1}): 3276, 2934, 2853, 2827, 1604, 1582, 1497, 1465, 1446, 1398, 1328, 1309, 1288, 1268, 1208, 1150, 1100, 1046, 952, 929, 879, 854, 831, 775, 744, 727, 708, 674, 650, 562, 446, 409. HRMS (ESI) calcd for $C_{75}H_{82}N_4O_{10}$ [M + H]$^+$ 1199.6104, found 1199.6140.

5,5′-(((3^2,3^5,5^2,5^5,7^2,7^5,9^2,9^5-Octamethoxy-1,3,5,7,9(1,4)-pentabenzenacyclodecaphane-1^2,1^5-diyl)bis(oxy))bis(butane-4,1-diyl))bis(9-methyl-5,6-dihydropyrido[1,2-*c*]quinazolin-7-ium) chloride (1b·Cl$_2$). To a dry 1,4-dioxane solution (25 mL) of **7b** (233 mg, 0.194 mmol) was added formaldehyde (0.19 mL, 37 wt% solution in H_2O, 2.5 mmol) under an Ar atmosphere. After 5 min, HCl (0.1 mL, 4 M solution in dioxane, 0.4 mmol) was added dropwise. The reaction mixture was stirred at r.t. for 2 h to furnish a yellow solid material, which was isolated by filtration, and washed with 1,4-dioxane and Et_2O to afford **BisC4**·Cl_2 as a yellow solid (164 mg, 0.127 mmol, yield = 67%). ^{1}H NMR (500 MHz, CD_2Cl_2, 298 K): δ 9.85 (s, 2H), 7.99–7.95 (m, 4H), 7.93–7.90 (m, 2H), 7.56 (td, J = 7.7, 1.4 Hz, 2H), 7.15–7.11 (m, 4H), 6.83 (d, J = 1.8 Hz, 6H), 6.69 (d, J = 5.4 Hz, 4H), 6.39 (d, J = 11.5 Hz, 2H), 6.13 (d, J = 11.5 Hz, 2H), 3.92–3.86 (m, 4H), 3.83–3.79 (m, 4H), 3.72–3.67 (m, 26H), 3.59 (s, 5H), 3.52 (s, 3H), 2.48 (s, 6H), 2.06 (p, J = 7.2 Hz, 4H), 1.94–1.89 (m, 4H). ^{13}C NMR (126 MHz, CD_2Cl_2, 298 K): δ 150.94, 150.85, 149.9, 146.8, 145.9, 144.4, 142.4, 136.6, 135.4, 128.9, 128.8, 128.6, 129.4, 122.3, 121.8, 117.0, 116.3, 114.6, 114.0, 113.8, 113.7, 68.9, 68.0, 67.6, 56.13, 56.09, 56.06, 50.9, 29.8, 29.4, 27.8, 25.2, 18.5. FT-IR (ATR, cm^{-1}): 3384, 2936, 2853, 2829, 1632, 1609, 1584, 1559, 1527, 1497, 1465, 1399, 1310, 1277, 1209, 1120, 1044, 993, 952, 929, 874, 855, 774, 758, 704, 673, 650, 613, 582, 558, 458. HRMS (ESI) calcd for $C_{77}H_{84}N_4O_{10}Cl_2$ [M - 2Cl]$^{2+}$ 612.3088, found 612.3097.

***N,N′*-(((3^2,3^5,5^2,5^5,7^2,7^5,9^2,9^5-Octamethoxy-1,3,5,7,9(1,4)-pentabenzenacyclodecaphane-1^2,1^5-diyl)bis(oxy))bis(hexane-6,1-diyl))bis(2-(5-methylpyridin-2-yl)aniline) (7h).** An oven-dried 25-mL Schlenk flask was charged with **6** (77.5 mg, 0.420 mmol) and dry THF (3 mL) under an Ar atmosphere. The solution was cooled to –78 °C, and n-butyllithium (0.27 mL, 1.6 M solution in hexanes, 0.43 mmol) was added dropwise via syringe over a period of 1 min. After 20 min, a solution of **5h** (200 mg, 0.191 mmol) in dry THF (3 mL) was added dropwise over a period of 5 min. The reaction mixture was warmed to r.t., stirred for 1 d, quenched by adding water (10 mL), and extracted into CH_2Cl_2 (50 mL × 3). The combined extracts were dried over anhyd Na_2SO_4, filtered, and concentrated under reduced pressure. Flash column chromatography on SiO_2 (hexane:EtOAc = 6:1, v/v) furnished **7h** as a yellow solid (85.7 mg, 0.0683 mmol, yield = 36%). ^{1}H NMR (500 MHz, CD_2Cl_2, 298 K): δ 8.41 (s, 2H), 8.29 (s, 2H), 7.62–7.57 (m, 4H), 7.55–7.52 (m, 2H), 7.26–7.21 (m, 2H), 6.89–6.86 (m, 10H), 6.74 (d, J = 8.3 Hz, 2H), 6.68 (t, J = 7.5 Hz, 2H), 3.95–3.90 (m, 2H), 3.88–3.84 (m, 2H), 3.75–3.70 (m, 34H), 3.22 (t, J = 7.0 Hz, 4H), 2.33 (s, 6H), 1.95–1.89 (m, 2H), 1.88–1.82 (m, 2H), 1.75 (q, J = 7.2 Hz, 4H), 1.68–1.61 (m, 4H), 1.59–1.54 (m, 4H). ^{13}C NMR (126 MHz, CD_2Cl_2, 298 K): δ 157.5, 150.93, 150.89, 150.2, 148.5, 148.1, 138.1, 130.9, 130.5, 129.6, 128.9, 128.8, 128.7, 122.1, 115.8, 114.6, 113.85, 113.79, 111.6, 68.7, 56.01, 55.99, 43.8, 30.5, 29.9, 29.7, 29.6, 29.5, 27.9, 26.8, 18.4. FT-IR (ATR, cm^{-1}): 3281, 2932, 2854, 2827, 1605,

1581, 1497, 1464, 1446, 1398, 1328, 1309, 1288, 1267, 1208, 1183, 1150, 1100, 1046, 952, 929, 880, 854, 775, 744, 727, 708, 675, 650, 562, 445, 426, 415, 405. HRMS (ESI) calcd for $C_{79}H_{90}N_4O_{10}$ $[M + H]^+$ 1255.6730, found 1255.6744.

5,5′-(((3^2,3^5,5^2,5^5,7^2,7^5,9^2,9^5-Octamethoxy-1,3,5,7,9(1,4)-pentabenzenacyclodecaphane-1^2,1^5-diyl)bis(oxy))bis(hexane-6,1-diyl))bis(9-methyl-5,6-dihydropyrido[1,2-*c*]quinazolin-7-ium) chloride (1h·Cl$_2$). To a dry 1,4-dioxane solution (9 mL) of **7h** (85.9 mg, 0.0684 mmol) was added formaldehyde (0.066 mL, 37 wt% solution in H_2O, 0.89 mmol) under an Ar atmosphere. After 5 min, HCl (0.035 mL, 4 M solution in dioxane, 0.14 mmol) was added dropwise. The reaction mixture was stirred at r.t. for 30 min, and concentrated under reduced pressure. The residual material was dissolved in a minimal amount of CH_2Cl_2 (ca. 1 mL) and added dropwise to cold Et_2O (30 mL) with stirring. The resulting suspension was centrifuged and the supernatant was decanted. The precipitate was resuspended in Et_2O with sonication and centrifuged again to afford **1h**·Cl_2 as a yellow solid (71.3 mg, 0.0527 mmol, yield = 78%). ^{1}H NMR (500 MHz, DMSO-d_6, 298 K): δ 8.81 (s, 2H), 8.46 (d, J = 8.6 Hz, 2H), 8.39 (d, J = 9.5 Hz, 2H), 8.19 (d, J = 8.9 Hz, 2H), 7.62 (t, J = 7.8 Hz, 2H), 7.27 (d, J = 8.4 Hz, 2H), 7.15 (t, J = 7.5 Hz, 2H), 6.78–6.71 (m, 10H), 5.71 (s, 4H), 3.88–3.79 (m, 2H), 3.73 (q, J = 6.6 Hz, 2H), 3.68–3.61 (m, 28H), 3.58 (s, 6H), 3.56–3.52 (m, 4H), 2.45 (s, 6H), 1.72 (dt, J = 13.5, 6.8 Hz, 4H), 1.63 (p, J = 7.6 Hz, 4H), 1.56–1.46 (m, 4H), 1.36 (p, J = 7.4 Hz, 4H). ^{13}C NMR (126 MHz, DMSO-d_6, 298 K): δ 149.9, 149.2, 145.9, 145.8, 143.5, 140.6, 135.4, 134.6, 127.6, 126.9, 122.4, 120.9, 116.4, 116.0, 114.1, 113.3, 113.2, 68.3, 67.7, 55.5, 50.0, 29.3, 27.1, 26.2, 25.5, 17.4. FT-IR (ATR, cm^{-1}): 3276, 2934, 2853, 2827, 1604, 1582, 1497, 1465, 1446, 1398, 1328, 1309, 1288, 1268, 1208, 1150, 1100, 1046, 952, 929, 879, 854, 831, 775, 744, 727, 708, 674, 650, 562, 446, 409. HRMS (ESI) calcd for $C_{81}H_{92}N_4O_{10}Cl_2$ $[M - 2Cl]^{2+}$ 640.3401, found 640.3418.

2-(5-Methylpyridin-2-yl)-*N*-(2-((1^5,3^2,3^5,5^2,5^5,7^2,7^5,9^2,9^5-nonamethoxy-1,3,5,7,9(1,4)-pentabenzenacyclodecaphane-1^2-yl)oxy)ethyl)aniline (9). An oven-dried 50-mL Schlenk flask was charged with **6** (175 mg, 0.951 mmol) and dry THF (5 mL) under an Ar atmosphere. The solution was cooled to –78 °C, and *n*-butyllithium (0.59 mL, 1.6 M solution in hexanes, 0.95 mmol) was added dropwise via syringe over a period of 1 min. After 30 min, a solution of **8** (730 mg, 0.865 mmol) in dry THF (6 mL) was added dropwise over a period of 5 min. The reaction mixture was warmed to r.t., stirred for 2 d, quenched by adding water (15 mL), and extracted into CH_2Cl_2 (50 mL × 3). The combined extracts were dried over anhyd Na_2SO_4, filtered, and concentrated under reduced pressure. Flash column chromatography on SiO_2 (hexane:EtOAc = 1:0 to 6:1, v/v) furnished **9** as a pale-brown solid (422 mg, 0.446 mmol, yield = 52%). ^{1}H NMR (400 MHz, CD_2Cl_2, 298 K): δ 8.62 (t, J = 4.9 Hz, 1H), 8.36 (s, 1H), 7.58 (s, 2H), 7.54 (d, J = 7.7 Hz, 1H), 7.25 (t, J = 7.7 Hz, 1H), 6.94–6.82 (m, 11H), 6.74 (t, J = 7.5 Hz, 1H), 4.14 (t, J = 5.6 Hz, 2H), 3.77–3.69 (m, 34H), 3.65 (q, J = 5.4 Hz, 2H), 3.58 (s, 3H), 2.30 (s, 3H). ^{13}C NMR (126 MHz, CD_2Cl_2, 298 K): δ 157.3, 151.1, 150.92, 150.91, 150.89, 150.86, 150.84, 150.79, 149.9, 148.3, 148.2, 138.1, 131.1, 130.4, 129.8, 129.0, 128.81, 128.76, 128.73, 128.72, 128.71, 128.68, 128.6, 122.5, 122.3, 116.5, 114.8, 113.93, 113.86, 113.80, 113.75, 113.7, 113.6, 111.8, 67.5, 56.02, 55.97, 55.90, 55.87, 43.9, 29.7, 29.65, 29.61, 29.59, 29.5, 18.4. FT-IR (ATR, cm^{-1}): 2933, 2852, 2828, 1736, 1605, 1582, 1497, 1464, 1447, 1399, 1309, 1208, 1183, 1152, 1100, 1047, 952, 930, 880, 855, 774, 746, 728, 705, 673, 650, 563, 462, 419. HRMS (ESI) calcd for $C_{58}H_{62}N_2O_{10}$ $[M + H]^+$ 947.4478, found 947.4477.

9-Methyl-5-(2-((1^5,3^2,3^5,5^2,5^5,7^2,7^5,9^2,9^5-nonamethoxy-1,3,5,7,9(1,4)-pentabenzenacyclodecaphane-1^2-yl)oxy)ethyl)-5,6-dihydropyrido[1,2-*c*]quinazolin-7-

ium chloride (2·Cl). To a dry 1,4-dioxane solution (8.5 mL) of **9** (122 mg, 0.129 mmol) was added formaldehyde (0.04 mL, 37 wt% solution in H_2O, 0.6 mmol) under an Ar atmosphere. After 5 min, HCl (0.04 mL, 4 M solution in dioxane, 0.1 mmol) was added dropwise. The reaction mixture was stirred at r.t. for 3 h to furnish a yellow solid material, which was isolated by filtration, and washed with 1,4-dioxane and Et_2O to afford **2**·Cl as a yellow solid (77.2 mg, 0.0775 mmol, yield = 60%). ^{1}H NMR (500 MHz, CD_2Cl_2, 298 K): δ 8.06 (s, 1H), 7.89 (d, J = 8.0 Hz, 1H), 7.66 (t, J = 7.8 Hz, 1H), 7.60 (d, J = 8.4 Hz, 1H), 7.27 (t, J = 7.6 Hz, 1H), 6.92 (d, J = 3.3 Hz, 2H), 6.88 (s, 1H), 6.83 (s, 1H), 6.79 (d, J = 5.2 Hz, 2H), 6.75 (s, 2H), 6.67 (s, 1H), 6.60 (s, 1H), 6.13 (s, 1H), 6.01 (s, 2H), 4.13 (t, J = 4.4 Hz, 2H), 3.90 (s, 2H), 3.80–3.68 (m, 28H), 3.65 (d, J = 5.4 Hz, 5H), 3.58 (s, 5H), 3.29 (s, 2H). ^{13}C NMR (126 MHz, MeOD, 298 K): δ 152.8, 152.55, 152.37, 152.26, 151.8, 149.7, 147.0, 145.7, 143.1, 141.4, 137.1, 135.9, 131.2, 131.0, 130.1, 129.9, 129.6, 129.4, 126.9, 125.4, 122.6, 121.9, 121.4, 116.1, 115.9, 114.0, 71.9, 70.5, 68.1, 57.11, 57.01, 56.8, 56.6, 56.47, 56.22, 56.1, 55.5, 31.0, 27.4, 15.6. FT-IR (ATR, cm^{-1}): 3367, 2934, 2828, 1630, 1608, 1524, 1497, 1464, 1398, 1310, 1262, 1208, 1182, 1098, 1042, 952, 930, 880, 855, 836, 802, 773, 760, 704, 672, 650, 616, 605, 580, 563, 538, 520, 498, 486, 473, 461, 451, 441, 431, 418, 406. HRMS (ESI) calcd for $C_{59}H_{63}N_2O_{10}Cl$ [M – Cl]$^+$ 959.4478, found 959.4478.

2-(5-Methylpyridin-2-yl)-*N*-(4-((1^5,3^2,3^5,5^2,5^5,7^2,7^5,9^2,9^5-nonamethoxy-1,3,5,7,9(1,4)-pentabenzenacyclodecaphane-1^2-yl)oxy)butyl)aniline) (9b). An oven-dried 50-mL Schlenk flask was charged with **6** (73.0 mg, 0.395 mmol) and dry THF (2 mL) under an Ar atmosphere. The solution was cooled to –78 °C, and *n*-butyllithium (0.26 mL, 1.6 M solution in hexanes, 0.41 mmol) was added dropwise. After 20 min, a solution of **8b** (265 mg, 0.304 mmol) in dry THF (2 mL) was added dropwise. The reaction mixture was warmed to r.t., stirred for 2 d, quenched by adding water (10 mL), and extracted into CH_2Cl_2 (50 mL × 3). The combined extracts were dried over anhyd Na_2SO_4, filtered, and concentrated under reduced pressure. Flash column chromatography on SiO_2 (hexane:EtOAc = 6:1 to 5:1, v/v) furnished **9b** as a pale-yellow solid (128 mg, 0.132 mmol, yield = 43%). ^{1}H NMR (500 MHz, CD_2Cl_2, 298 K): δ 8.39–8.34 (m, 2H), 7.62–7.57 (m, 2H), 7.54 (dd, J = 7.8, 1.7 Hz, 1H), 7.24 (ddd, J = 8.5, 7.2, 1.6 Hz, 1H), 6.91–6.86 (m, 9H), 6.84 (s, 1H), 6.75 (d, J = 8.4 Hz, 1H), 6.70 (td, J = 7.5, 1.2 Hz, 1H), 3.96 (t, J = 5.9 Hz, 2H), 3.77–3.71 (m, 34H), 3.63 (s, 3H), 3.31 (q, J = 5.7, 5.3 Hz, 2H), 2.31 (s, 3H), 2.01–1.95 (m, 4H). ^{13}C NMR (126 MHz, CD_2Cl_2, 298 K): δ 157.4, 150.94, 150.90, 150.85, 150.2, 148.4, 148.1, 138.1, 131.0, 130.5, 129.6, 129.0, 128.9, 128.78, 128.74, 128.71, 128.68, 128.64, 122.1, 121.6, 115.9, 114.8, 113.9, 113.84, 113.80, 113.7, 113.7, 111.6, 68.6, 56.04, 55.98, 55.92, 55.86, 43.7, 29.9, 29.73, 29.68, 29.6, 29.4, 28.4, 26.9, 18.3. FT-IR (ATR, cm^{-1}): 2988, 2935, 2827, 1736, 1605, 1582, 1497, 1464, 1446, 1398, 1309, 1268, 1207, 1183, 1151, 1101, 1045, 997, 952, 930, 880, 855, 773, 746, 728, 705, 673, 650, 563, 465, 424,417, 410, 403. HRMS (ESI) calcd for $C_{60}H_{66}N_2O_{10}$ [M + H]$^+$ 975.4790, found 975.4813.

9-Methyl-5-(4-((1^5,3^2,3^5,5^2,5^5,7^2,7^5,9^2,9^5-nonamethoxy-1,3,5,7,9(1,4)-pentabenzenacyclodecaphane-1^2-yl)oxy)butyl)-5,6-dihydropyrido[1,2-*c*]quinazolin-7-ium chloride (2b·Cl). To a dry 1,4-dioxane solution (4 mL) of **9b** (51.4 mg, 0.0527 mmol) was added formaldehyde (0.02 mL, 37 wt% solution in H_2O, 0.3 mmol) under an Ar atmosphere. After 5 min, HCl (0.02 mL, 4 M solution in dioxane, 0.06 mmol) was added dropwise. The reaction mixture was stirred at r.t. for 2 h, and concentrated under reduced pressure. The residual material was dissolved in a minimal amount of CH_2Cl_2 (ca. 1 mL) and added dropwise to cold Et_2O (30 mL) with stirring. The resulting suspension was centrifuged and the supernatant was decanted. The precipitate was resuspended in Et_2O with sonication and centrifuged again to afford **2b**·Cl as a yellow solid (37.7 mg, 0.0368 mmol, yield = 70%). ^{1}H NMR (500 MHz, CD_2Cl_2, 298 K): δ 10.06 (s,

1H), 7.97 (d, J = 8.4 Hz, 1H), 7.88 (t, J = 7.9 Hz, 2H), 7.55 (t, J = 5.4 Hz, 1H), 7.15–7.10 (m, 2H), 6.88–6.87 (m, 3H), 6.86–6.84 (m, 4H), 6.83 (s, 1H), 6.78 (s, 1H), 6.71 (s, 1H), 6.33 (s, 2H), 3.91 (t, J = 5.9 Hz, 2H), 3.83 (t, J = 6.2 Hz, 2H), 3.75–3.72 (m, 24H), 3.71–3.70 (m, 8H), 3.62–3.60 (m, 5H), 2.52 (s, 3H), 2.04 (q, J = 6.4, 5.6 Hz, 2H), 1.95–1.90 (m, 2H). ^{13}C NMR (126 MHz, CD_2Cl_2, 298 K): δ 151.0, 150.94, 150.88, 150.8, 149.9, 146.9, 145.6, 144.4, 142.9, 136.6, 135.5, 129.0, 128.9, 128.78, 128.75, 128.65, 128.58, 126.8, 122.0, 121.7, 116.9, 116.3, 114.9, 114.0, 113.91, 113.87, 113.80, 113.76, 113.65, 113.56, 69.0, 68.2, 56.14, 56.09, 56.07, 56.00, 55.96, 55.8, 51.0, 29.82, 29.80, 29.6, 29.5, 29.3, 27.8, 25.3, 18.6. FT-IR (ATR, cm^{-1}): 3377, 2935, 2828, 1631, 1609, 1584, 1560, 1527, 1497, 1465, 1398, 1309, 1276, 1207, 1182, 1101, 1044, 995, 952, 930, 880, 855, 773, 759, 729, 704, 673, 650, 583, 558, 459, 423, 413. HRMS (ESI) calcd for $C_{61}H_{67}N_2O_{10}Cl$ [M – Cl]$^+$ 987.4790, found 987.4809.

2-(5-Methylpyridin-2-yl)-*N*-(6-((1^5,3^2,3^5,5^2,5^5,7^2,7^5,9^2,9^5-nonamethoxy-1,3,5,7,9(1,4)-pentabenzenacyclodecaphane-1^2-yl)oxy)hexyl)aniline) (9h). An oven-dried 50-mL Schlenk flask was charged with **6** (67.1 mg, 0.364 mmol) and dry THF (2 mL) under an Ar atmosphere. The solution was cooled to –78 °C, and *n*-butyllithium (0.24 mL, 1.6 M solution in hexanes, 0.38 mmol) was added dropwise. After 20 min, a solution of **8h** (252 mg, 0.280 mmol) in dry THF (2 mL) was added dropwise. The reaction mixture was warmed to r.t., stirred for 18 h, quenched by adding water (10 mL), and extracted into CH_2Cl_2 (50 mL × 3). The combined extracts were dried over anhyd Na_2SO_4, filtered, and concentrated under reduced pressure. Flash column chromatography on SiO_2 (hexane:EtOAc = 7:1, v/v) furnished **9h** as a pale-yellow solid (92.8 mg, 0.0924 mmol, yield = 33%). ^{1}H NMR (500 MHz, CD_2Cl_2, 298 K): δ 8.41 (s, 1H), 8.28 (s, 1H), 7.62–7.58 (m, 2H), 7.53 (dd, J = 7.8, 1.7 Hz, 1H), 7.23 (ddd, J = 8.5, 7.2, 1.6 Hz, 1H), 6.90–6.86 (m, 10H), 6.74 (d, J = 7.24 Hz, 1H), 6.69 (t, J = 7.41 Hz, 1H), 3.89 (t, J = 6.5 Hz, 2H), 3.76–3.71 (m, 37H), 3.22 (t, J = 7.0 Hz, 2H), 2.33 (s, 3H), 1.89 (q, J = 7.0 Hz, 2H), 1.76 (q, J = 7.0 Hz, 2H), 1.66–1.61 (m, 2H), 1.59–1.55 (m, 2H). ^{13}C NMR (126 MHz, CD_2Cl_2, 298 K): δ 157.5, 151.0, 150.94, 150.92, 150.89, 150.3, 148.5, 148.1, 138.1, 131.0, 130.5, 129.6, 128.9, 128.81, 128.80, 128.75, 128.7, 122.2, 121.6, 115.8, 114.8, 113.90, 113.89, 113.87, 113.8, 113.7, 111.6, 68.8, 56.04, 56.01, 55.99, 55.9, 43.8, 30.5, 29.9, 29.8, 29.73, 29.69, 29.6, 29.5, 27.9, 26.8, 18.4. FT-IR (ATR, cm^{-1}): 2933, 2854, 2827, 1738, 1605, 1582, 1497, 1464, 1447, 1398, 1308, 1207, 1182, 1151, 1101, 1046, 997, 952, 930, 880, 854, 774, 745, 728, 704, 673, 650, 562, 461. HRMS (ESI) calcd for $C_{62}H_{70}N_2O_{10}$ [M + H]$^+$ 1003.5103, found 1003.5115.

9-Methyl-5-(6-((1^5,3^2,3^5,5^2,5^5,7^2,7^5,9^2,9^5-nonamethoxy-1,3,5,7,9(1,4)-pentabenzenacyclodecaphane-1^2-yl)oxy)hexyl)-5,6-dihydropyrido[1,2-*c*]quinazolin-7-ium chloride (2h·Cl). To a dry 1,4-dioxane solution (9.3 mL) of **9h** (70.4 mg, 0.0701 mmol) was added formaldehyde (0.03 mL, 37 wt% solution in H_2O, 0.4 mmol) under an Ar atmosphere. After 5 min, HCl (0.02 mL, 4 M solution in dioxane, 0.08 mmol) was added dropwise. The reaction mixture was stirred at r.t. for 30 min, and concentrated under reduced pressure. The residual material was dissolved in a minimal amount of CH_2Cl_2 (ca. 1 mL) and added dropwise to cold Et_2O (30 mL) with stirring. The resulting suspension was centrifuged and the supernatant was decanted. The precipitate was resuspended in Et_2O with sonication and centrifuged again to afford **2h**·Cl as a yellow solid (55.7 mg, 0.0530 mmol, yield = 76%).^{1}H NMR (500 MHz, DMSO-d_6, 298 K): δ 8.80 (s, 1H), 8.46 (d, J = 8.6 Hz, 1H), 8.37 (d, J = 8.5 Hz, 1H), 8.19 (d, J = 7.6 Hz, 1H), 7.62 (t, J = 7.7 Hz, 1H), 7.27 (d, J = 8.4 Hz, 1H), 7.15 (t, J = 7.6 Hz, 1H), 6.80–6.67 (m, 10H), 5.71 (s, 2H), 3.79 (t, J = 6.2 Hz, 2H), 3.64 (d, J = 8.5 Hz, 34H), 3.59 (s, 3H), 3.56–3.52 (m, 2H), 2.43 (s, 3H), 1.70 (dt, J = 17.8, 9.1 Hz, 2H), 1.63 (q, J = 7.5 Hz, 2H), 1.52 (p, J = 7.6 Hz, 2H), 1.37 (q, J = 7.6 Hz, 2H). ^{13}C NMR (126 MHz, CD_2Cl_2, 298 K): δ 150.0, 149.94, 149.88, 149.8,

149.2, 145.9, 145.8, 143.5, 140.6, 135.4, 134.6, 127.5, 127.4, 126.9, 122.4, 120.9, 116.4, 116.0, 114.1, 113.3, 68.3, 67.6, 64.9, 55.4, 50.0, 29.0, 27.1, 26.2, 25.5, 17.4. FT-IR (ATR, cm^{-1}): 3339, 2932, 2855, 2828, 2199, 2059, 1631, 1608, 1584, 1559, 1526, 1497, 1464, 1398, 1312, 1278, 1208, 1121, 1096, 1040, 984, 928, 880, 854, 775, 760, 729, 713, 650, 582, 559, 497, 478, 462, 445, 436, 428, 406. HRMS (ESI) calcd for $C_{63}H_{71}N_2O_{10}Cl$ [M - Cl]$^+$ 1015.5103, found 1015.5142.

***N,N′*-((1,4-Phenylenebis(oxy))bis(ethane-2,1-diyl))bis(2-(5-methylpyridin-2-yl)aniline) (11).** An oven-dried 50-mL Schlenk flask was charged with **6** (398 mg, 2.16 mmol) and dry THF (8 mL) under an Ar atmosphere. The solution was cooled to –78 ℃, and *n*-butyllithium (0.86 mL, 2.5 M solution in hexanes, 2.2 mmol) was added dropwise via syringe over a period of 1 min. After 30 min, a solution of **10** (350 mg, 1.08 mmol) in dry THF (10 mL) was added dropwise over a period of 5 min. The reaction mixture was warmed to r.t., stirred for 2 d, quenched by adding water (15 mL), and extracted into CH_2Cl_2 (70 mL × 3). The combined extracts were dried over anhyd Na_2SO_4, filtered, and concentrated under reduced pressure. Flash column chromatography on SiO_2 (hexane:EtOAc = 1:0 to 1:1, v/v) furnished **11** as a pale-brown solid (318 mg, 0.599 mmol, yield = 55%). ^1H NMR (400 MHz, CD_2Cl_2, 298 K): δ 8.51 (t, J = 5.0 Hz, 2H), 8.37 (s, 2H), 7.58 (s, 4H), 7.52 (d, J = 7.7 Hz, 2H), 7.24 (t, J = 7.7 Hz, 2H), 6.88 (s, 4H), 6.79 (d, J = 8.2 Hz, 2H), 6.72 (t, J = 7.6 Hz, 2H), 4.16 (t, J = 5.5 Hz, 4H), 3.56 (q, J = 5.6 Hz, 4H), 2.33 (s, 6H). ^{13}C NMR (126 MHz, CD_2Cl_2, 298 K): δ 157.1, 153.7, 148.0, 147.9, 138.0, 130.9, 130.3, 129.6, 122.24, 122.08, 116.35, 116.23, 116.07, 111.7, 67.9, 43.2, 18.3. FT-IR (ATR, cm^{-1}): 3244, 3006, 2924, 2858, 1604, 1581, 1506, 1486, 1445, 1378, 1326, 1284, 1230, 1166, 1147, 1110, 1060, 1046, 1031, 1019, 953, 825, 749, 709, 676, 650, 603, 560, 518, 483, 451, 417. HRMS (ESI) calcd for $C_{34}H_{34}N_4O_2$ [M + H]$^+$ 531.2755, found 531.2759.

5,5′-((1,4-Phenylenebis(oxy))bis(ethane-2,1-diyl))bis(9-methyl-5,6-dihydropyrido[1,2-*c*]quinazolin-7-ium) chloride (3·Cl_2). To a dry 1,4-dioxane solution (11 mL) of **11** (104 mg, 0.197 mmol) was added formaldehyde (0.19 mL, 37 wt% solution in H_2O, 2.6 mmol) under an Ar atmosphere. After 5 min, HCl (0.1 mL, 4 M solution in dioxane, 0.4 mmol) was added dropwise. The reaction mixture was stirred at r.t. for 3 h to furnish an orange solid material, which was isolated by filtration, and washed with 1,4-dioxane and Et_2O to afford **3**·Cl_2 as a orange solid (93.9 mg, 0.149 mmol, yield = 76%). ^1H NMR (500 MHz, CD_2Cl_2, 298 K): δ 9.93 (s, 2H), 8.00 (d, J = 8.2 Hz, 2H), 7.83 (m, 4H), 7.54 (t, J = 7.8 Hz, 2H), 7.21 (d, J = 8.4 Hz, 2H), 7.10 (t, J = 7.6 Hz, 2H), 6.38 (s, 4H), 6.26 (s, 4H), 4.07 (t, J = 4.6 Hz, 4H), 4.00 (d, J = 4.4 Hz, 4H), 2.41 (s, 6H). ^{13}C NMR (126 MHz, DMSO−d_6, 298 K): δ 151.9, 145.6, 145.0, 143.2, 140.9, 135.3, 134.4, 126.8, 122.4, 121.6, 117.5, 117.4, 114.8, 69.3, 66.1, 55.0, 50.8, 17.4. FT-IR (ATR, cm^{-1}): 3370, 2924, 1671, 1631, 1608, 1559, 1526, 1507, 1459, 1356, 1276, 1221, 1044, 954, 835, 762, 711, 620, 605, 580, 563, 538, 520, 498, 486, 473, 461, 450, 441, 431, 418, 406. HRMS (ESI) calcd for $C_{36}H_{36}N_4O_2Cl_2$ [M – 2Cl]$^{2+}$ 278.1414, found 278.1414.

***N*-(2-(4-Methoxyphenoxy)ethyl)-2-(5-methylpyridin-2-yl)aniline (13).** An oven-dried 50-mL Schlenk flask was charged with **6** (180 mg, 0.975 mmol) and dry THF (9 mL) under an Ar atmosphere. The solution was cooled to –78 ℃, and *n*-butyllithium (0.39 mL, 2.5 M solution in hexanes, 0.98 mmol) was added dropwise via syringe over a period of 1 min. After 10 min, a solution of **12** (249 mg, 1.08 mmol) in dry THF (3 mL) was added dropwise over a period of 5 min. The reaction mixture was warmed to r.t., stirred for 2 d, quenched by adding water (15 mL), and extracted into CH_2Cl_2 (30 mL × 3). The combined extracts were dried over anhyd Na_2SO_4, filtered, and concentrated under reduced pressure. Flash column chromatography on SiO_2 (hexane:EtOAc =

1:0 to 17:3, v/v) furnished **13** as a pale-yellow solid (160 mg, 0.477 mmol, yield = 50%). ^{1}H NMR (500 MHz, CD_2Cl_2, 298 K): δ 8.54 (s, 1H), 8.37 (s, 1H), 7.58 (s, 2H), 7.55–7.50 (m, 1H), 7.24 (t, J = 7.6 Hz, 1H), 6.90–6.81 (m, 4H), 6.79 (d, J = 8.2 Hz, 1H), 6.72 (t, J = 7.0 Hz, 1H), 4.16 (t, J = 5.5 Hz, 2H), 3.75 (s, 3H), 3.56 (q, J = 5.2 Hz, 2H), 2.34 (s, 3H). ^{13}C NMR (126 MHz, CD_2Cl_2, 298 K): δ 157.2, 154.6, 153.5, 148.18, 148.06, 138.1, 131.0, 130.4, 129.7, 122.32, 122.19, 116.45, 116.24, 115.1, 111.8, 68.1, 56.1, 43.3, 18.4. FT-IR (ATR, cm^{-1}): 3268, 3000, 2925, 2832, 1603, 1579, 1505, 1486, 1460, 1443, 1378, 1327, 1287, 1228, 1179, 1165, 1149, 1106, 1032, 926, 825, 743, 655, 562, 522, 447, 406. HRMS (ESI) calcd for $C_{21}H_{22}N_2O_2$ [M + H]$^+$ 335.1755, found 335.1756.

5-(2-(4-Methoxyphenoxy)ethyl)-9-methyl-5,6-dihydropyrido[1,2-*c*]quinazolin-7-ium chloride (4·Cl). To a dry 1,4-dioxane solution (6 mL) of **13** (84.9 mg, 0.254 mmol) was added formaldehyde (0.09 mL, 37 wt% solution in H_2O, 1.0 mmol) under an Ar atmosphere. After 5 min, HCl (0.07 mL, 4 M solution in dioxane, 0.3 mmol) was added dropwise. The precipitate was isolated immediately by filtration, and washed with 1,4-dioxane and Et_2O to afford **4**·Cl as an orange solid (68.3 mg, 0.178 mmol, yield = 76%). ^{1}H NMR (500 MHz, CD_2Cl_2, 298 K): δ 10.04 (s, 1H), 7.96 (d, J = 8.2 Hz, 1H), 7.88 (d, J = 8.0 Hz, 2H), 7.62 (t, J = 7.3 Hz, 1H), 7.33 (d, J = 8.2 Hz, 1H), 7.18 (t, J = 7.3 Hz, 1H), 6.69 (d, J = 8.7 Hz, 2H), 6.59 (t, J = 8.8 Hz, 2H), 6.36 (s, 2H), 4.10 (s, 2H), 3.70 (s, 3H), 2.50 (s, 3H). ^{13}C NMR (126 MHz, CD_2Cl_2, 298 K): δ 154.5, 152.5, 146.9, 145.3, 144.1, 142.7, 136.5, 135.1, 126.5, 122.49, 122.29, 118.6, 118.0, 115.5, 114.8, 69.1, 67.2, 56.1, 52.1, 18.5. FT-IR (ATR, cm^{-1}): 3376, 2934, 1673, 1631, 1607, 1584, 1558, 1526, 1506, 1459, 1357, 1316, 1275, 1225, 1182, 1109, 1037, 955, 904, 829, 760, 737, 707, 672, 620, 605, 580, 562, 537, 521, 498, 486, 473, 461, 451, 441, 431, 419, 406. HRMS (ESI) calcd for $C_{22}H_{23}N_2O_2Cl$ [M – Cl]$^+$ 347.1755, found 347.1752.

3. SC-XRD Experiments

X-ray Crystallographic Studies on 1·Cl_2. Single crystals of **1**·Cl_2 were obtained by slow diffusion of cyclopentane into a CH_2Cl_2 solution of this material. A yellow crystal (approximate dimensions 0.11 × 0.1 × 0.05 mm^3) was coated with Immersion Oil Type NVH to mount on the micro-loop under cold nitrogen stream at T= 100 K. The diffraction data measured using synchrotron radiation (λ = 0.70000 Å) employing a PLSII-2D SMC on a Rayonix SX165 CCD area detector with high precision one-axis goniostat at Pohang Accelerator Laboratory, Korea. The PAL BL2D-SMDC program[23] was used for data collection, and HKL3000sm (Ver.717)[24] was used for cell refinement, reduction, and absorption correction. A total of 23498 reflections were measured (2.832° ≤ 2θ ≤ 49.996°). The structure was solved with the SHELXT[25] structure solution program using intrinsic phasing, and refined with the SHELXL[26] refinement package of OLEX2.[27] The final refinements of **1**·Cl_2 were performed using the SQUEEZE routine of PLATON package.[28] A total of 12005 unique reflections were used in all calculations. The final $R1$ was 0.1698 ($I \geq 2\sigma(I)$), and $wR2$ was 0.2895 (all data). $C_{73}H_{76}Cl_2N_4O_{10}$, M = 1240.33 g/mol, triclinic, $P\bar{1}$ (no. 2), a = 10.638(2) Å, b = 12.743(3) Å, c = 29.159(6) Å, α = 102.21(3)°, β = 93.03(3)°, γ = 103.43(3)°, V = 3736.1(14) $Å^3$, Z = 2, D_c = 1.253 g/cm^3. CCDC 2432639 contains the supplementary crystallographic data for this structure.

X-ray Crystallographic Studies on 2·Cl. Single crystals of **2**·Cl were obtained by slow diffusion of hexane into a CH_2Cl_2 solution of this material. A yellow crystal (approximate dimensions 0.080 × 0.060 × 0.040 mm^3) was placed onto a nylon loop with Paratone-N oil, and mounted on a Bruker single Crystal X-ray diffractometer. The data collection was carried out using Mo Kα radiation, and the crystal was kept at T = 100.0 K. A total of 214027 reflections were measured (4.486° ≤ 2θ ≤ 52.8°). The structure was solved with the SHELXT[25] structure solution program using intrinsic phasing, and refined with the SHELXL[26] refinement package of OLEX2.[27] The final refinements of **2**·Cl were performed using the SQUEEZE routine of PLATON package.[28] A total of 12426 unique reflections were used in all calculations. The final $R1$ was 0.1279 ($I \geq 2\sigma(I)$), and $wR2$ was 0.2797 (all data). $C_{59}H_{63}N_2O_{10}Cl$, M = 995.61 g/mol, trigonal, $R\bar{3}$ (no. 148), a = 19.1322(11) Å, c = 86.425(7) Å, V = 27397(4) $Å^3$, Z = 18, D_c = 1.179 g/cm^3. CCDC 2432640 contains the supplementary crystallographic data for this structure.

[23] J. W. Shin, K. Eom and D. Moon, *J. Synchrotron Radiat.*, 2016, **23**, 369–373.
[24] Z. Otwinowski and W. Minor, *Methods Enzymol.*, 1997, **276**, 307–326.
[25] G. Sheldrick, *Acta Crystallogr., Sect. A: Found. Adv.*, 2015, **71**, 3–8.
[26] G. Sheldrick, *Acta Crystallogr., Sect. C: Struct. Chem.*, 2015, **71**, 3–8.
[27] O. V. Dolomanov, L. J. Bourhis, R. J. Gildea, J. A. K. Howard and H. Puschmann, *J. Appl. Crystallogr.*, 2009, **42**, 339–341.
[28] A. Spek, *Acta Crystallogr., Sect. D: Struct. Biol.*, 2009, **65**, 148–155.

X-ray Crystallographic Studies on [$\mathbf{1} \supset$ DCB]$\cdot$Cl$_2$ complex. Single crystals of [$\mathbf{1} \supset$ DCB]$\cdot$Cl$_2$ complex were obtained by slow evaporation of CH_2Cl_2 solution containing host ($\mathbf{1}\cdot$Cl$_2$) and guest (1,4-DCB) in a 1:150 molar ratio. An orange crystal (approximate dimensions 0.3 $\times$ 0.12 $\times$ 0.09 mm^3) was placed onto a nylon loop with Paratone-N oil, and mounted on a Bruker single Crystal X-ray diffractometer. The data collection was carried out using Mo Kα radiation, and the crystal was kept at T = 100.0 K. A total of 189378 reflections were measured ($3.766° \leq 2\theta \leq 52.808°$). The structure was solved with the SHELXT[25] structure solution program using intrinsic phasing, and refined with the SHELXL[26] refinement package of OLEX2.[27] The final refinements of [$\mathbf{1} \supset$ DCB]$\cdot$Cl$_2$ were performed using the SQUEEZE routine of PLATON package.[28] A total of 15912 unique reflections were used in all calculations. The final $R1$ was 0.1760 ($I \geq 2\sigma(I)$), and $wR2$ was 0.4193 (all data). $C_{79}H_{84}Cl_2N_6O_{10}$, M = 1348.42 g/mol, triclinic, $P\bar{1}$ (no. 2), a = 10.9904(10) Å, b = 12.9193(12) Å, c = 28.626(3) Å, α = 79.392(3)°, β = 80.451(3)°, γ = 79.546(3)°, V = 3891.7(6) Å^3, Z = 2, D_c = 1.151 g/cm^3. CCDC 2552442 contains the supplementary crystallographic data for this structure.

4. Validation Reply Forms

Validation Reply Forms: Detailing any alerts in the [**1** ⊃ DCB]·Cl_2 crystal structure.
_vrf_PLAT084_ALERT_3_B
PROBLEM: High wR_2 Value (i.e. > 0.25) 0.42 Report
RESPONSE: The high wR_2 value is caused by weak diffraction from the small crystal and severe disorder of the encapsulated 1,4-dicyanobutane guest molecule. Despite these limitations, the molecular connectivity of the host–guest complex and the overall inclusion geometry are unambiguously established.

_vrf_PLAT340_ALERT_3_B
PROBLEM: Low Bond Precision on C–C Bonds 0.01213 Ang.
RESPONSE: The low bond precision is a consequence of weak diffraction and guest disorder. Nevertheless, the precision is sufficient to establish the molecular connectivity and host–guest inclusion geometry.

5. Supplementary Tables

Table S1. Summary of X-Ray Crystallographic Data.

	**1**·Cl_2	**2**·Cl	[**1** ⊃ DCB]·Cl_2
Chemical formula	$C_{73}H_{76}Cl_2N_4O_{10}$	$C_{59}H_{63}ClN_2O_{10}$	$C_{79}H_{84}Cl_2N_6O_{10}$
Formula weight	1240.33	995.61	1348.42
Crystal system	triclinic	trigonal	triclinic
Space group	$P\bar{1}$	$R\bar{3}$	$P\bar{1}$
Color of crystal	Yellow	Yellow	Orange
a (Å)	10.638(2)	19.1322(11)	10.9904(10)
b (Å)	12.743(3)	19.1322(11)	12.9193(12)
c (Å)	29.159(6)	86.425(7)	28.626(3)
α (°)	102.21(3)		79.392(3)
β (°)	93.03(3)		80.451(3)
γ (°)	103.43(3)		79.546(3)
Volume (Å^3)	3736.1(14)	27397(4)	3891.7(6)
Z	2	18	2
R_{int}	0.0683	0.0823	0.0960
Final R indices [$I > 2\sigma(I)$]	$R1 = 0.0896$, $wR2 = 0.2458$	$R1 = 0.1135$, $wR2 = 0.2689$	$R1 = 0.1395$, $wR2 = 0.3859$
Final R indices [all data]	$R1 = 0.1698$, $wR2 = 0.2895$	$R1 = 0.1279$, $wR2 = 0.2797$	$R1 = 0.1760$, $wR2 = 0.4193$
Goodness-of-fit on F^2	0.986	1.102	1.012

Table S2. Photophysical properties in MeOH (T = 293 K).

	$\lambda_{abs,max}{}^{a}$ (nm)	$\lambda_{em,max}$ (nm)	Φ_F (%)
**1**·Cl_2	390	495; 580	4.4
**2**·Cl	383	515	4.5
**3**·Cl_2	394	595	4.6
**4**·Cl	397	595	7.8
**1b**·Cl_2	410	575	3.6
**2b**·Cl	413	580	10.0
**1h**·Cl_2	407	563	3.5
**2h**·Cl	413	575	4.3

[a] The longest wavelength absorption maximum.

Table S3. Summary of emission lifetime (τ) and pre-exponential factor (B) for **1**·Cl_2 monitored at 485 nm and 620 nm upon excitation at 375 nm in MeOH (T = 293 K).[a]

λ_{em} (nm)	τ_{av} (ns)	τ_1 (ns)	τ_2 (ns)	B_1 (Rel %)	B_2 (Rel %)
485	0.24	0.16	7.6	63	37
620	0.54	0.74	4.4	92	8

[a] $R(t) = B_1 e^{-t/\tau_1} + B_2 e^{-t/\tau_2}$

Table S4. Voigt peak function parameters for **2**·Cl, **4**·Cl, and **1**·Cl_2 used in spectral fitting with the program Fityk.[29]

**Compound**	**2**	**4**	**1**
Center (cm^{-1})	19135	16769	20275; 17171
FWHM (cm^{-1})	3758	3417	2946; 3881
R-squared	0.9792	0.9986	0.9997

[29] M. Wojdyr, *J. Appl. Crystallogr.*, 2010, **43**, 1126–1128.

Table S5. Excitation energies, wavelengths, oscillator strengths, frontier molecular orbital (FMO) transitions, configuration interaction (CI) expansion coefficients, and contributions of the FMO transition for the S_0-to-S_1 transitions of **ANIPY-Me**, calculated at the TD-B3LYP-D3/6-311++G** level with implicit solvation in MeOH.

	**ANIPY-Me**
Energy (eV)	2.90
λ (nm)	427
f	0.1729
FMO transition	H $\rightarrow$ L
CI expansion coefficient	0.70387
Contribution (%)	99

Table S6. Emission energies, wavelengths, oscillator strengths, frontier molecular orbital (FMO) transitions, configuration interaction (CI) expansion coefficients, and contributions of the FMO transition for the S_0-to-S_1 transitions of **ANIPY-Me**, calculated at the TD-B3LYP-D3/6-311++G** level with implicit solvation in MeOH.

	**ANIPY-Me**
Energy (eV)	2.03
λ (nm)	613
f	0.0927
FMO transition	H $\rightarrow$ L
CI expansion coefficient	0.70663
Contribution (%)	100

Table S7. Binding constants K_{HG} for 1:1 complexation of dicyanoalkane guest with host **1** or **2**.

	1,2-DCE	1,4-DCB
**1**	$(1.8 \pm 0.5) \times 10^2$ M^{-1}	$(2.0 \pm 0.2) \times 10^2$ M^{-1}
**2**	$(4.6 \pm 0.3) \times 10^2$ M^{-1}	$(2.9 \pm 0.4) \times 10^2$ M^{-1}

6. Supplementary Note

Supplementary Note 1. Determining the Host–Guest Complexation Constant (K_{HG}). Fluorescence titration studies were carried out in MeOH at T = 293 K. The concentration of the host molecule was kept at 0.1 mM. Aliquots of the guest 1,2-dicyanoethane were delivered from the stock solution (300 mM).

The host–guest complexation constant expression between host H and guest G is described below. Let the system be the 1:1 complex formation equilibrium according to eqn (4).

$$\mathsf{H} + \mathsf{G} \rightleftharpoons \mathsf{HG} \tag{4}$$

The host–guest complexation constant (K_{HG}) is expressed as eqn (5).

$$K_{\mathsf{HG}} = \frac{[\mathsf{HG}]}{[\mathsf{H}][\mathsf{G}]} \tag{5}$$

The mass balance is described by eqn (6) and (7).

$$[\mathsf{H}]_0 = [\mathsf{H}] + [\mathsf{HG}] \tag{6}$$

$$[\mathsf{G}]_0 = [\mathsf{G}] + [\mathsf{HG}] \tag{7}$$

The fluorescence intensity is proportional to the concentration of the fluorescent molecule in the low concentration range. It is written as eqn (8).[30]

$$\mathsf{F} = I_0 \Phi \varepsilon \mathsf{b}\,[\mathsf{X}] = k_{\mathsf{i}}\,[\mathsf{X}] \tag{8}$$

Here, F is the fluorescence intensity, I_0 is the intensity of the excitation radiation, ε is the molar absorptivity at the excitation wavelength, b is the path length, $[\mathsf{X}]$ is the molar concentration, Φ is the fluorescence quantum yield, and k_i represents a proportionality constant. The total intensity of the fluorescence F is written as eqn (9).[31]

$$\mathsf{F} = k_{\mathsf{H}}\,[\mathsf{H}] + k_{\mathsf{G}}\,[\mathsf{G}] + k_{\mathsf{HG}}\,[\mathsf{HG}] \tag{9}$$

Before any guest is added to the solution, the observed fluorescence intensity is described by eqn (10).

$$\mathsf{F}_0 = k_{\mathsf{H}}^0\,[\mathsf{H}]_0 \tag{10}$$

[30] P. Thordarson, *Chem. Soc. Rev.*, 2011, **40**, 1305–1323.

[31] K. Kimura, H. Sakamoto, Y. Liu, H. Tsukube, H. Furuta, A. Odani, Y. Takeda, Y. Kudo and Y. Inoue, in *Comprehensive Supramolecular Chemistry*, ed. J. E. D. Davies and J. A. Ripmeester, Pergamon, Oxford, 1996, vol. 8, pp. 442–443.

After the addition of a guest, the resulting fluorescence is given by eqn (11).

$$\mathsf{F} = k_{\mathsf{H}}\,[\mathsf{H}] + k_{\mathsf{HG}}\,[\mathsf{HG}] \tag{11}$$

The fluorescence spectra show that emission persists upon guest addition without abrupt quenching, suggesting that the fluorescence quantum yield of the free host remains largely unchanged. Similar fluorescence behavior is ascribed to static complex formation in other pillar[5]arene-based host–guest systems.[32] [33]

Based on eqn (10), this supports the assumption given in eqn (12).

$$k_{\mathsf{H}}^{0} = k_{\mathsf{H}} \tag{12}$$

From eqs (5)–(12), the following relationship is established as eqn (13).

$$\mathsf{F} = k_{\mathsf{H}}\,[\mathsf{H}]_0 + \frac{(k_{\mathsf{HG}} - k_{\mathsf{H}})}{2}\left\{[\mathsf{H}]_0 + [\mathsf{G}]_0 + \frac{1}{K_{\mathsf{HG}}} - \sqrt{\left([\mathsf{H}]_0 + [\mathsf{G}]_0 + \frac{1}{K_{\mathsf{HG}}}\right)^2 - 4\,[\mathsf{H}]_0\,[\mathsf{G}]_0}\right\} \tag{13}$$

Using eq (13), fitting was carried out by non-linear least-squares regression analysis to obtain K_{HG}.

[32] Z. Qi, K. Achazi, R. Haag, S. Dong and C. A. Schalley, *Chem. Commun.*, 2015, **51**, 10326–10329.

[33] I. Pisagatti, D. Crisafulli, A. Pappalardo, G. Trusso Sfrazzetto, A. Notti, F. Nastasi, M. F. Parisi, N. Micali, G. Gattuso and V. Villari, *Mater. Today Chem.*, 2022, **24**, 100841.

7. Supplementary Figures

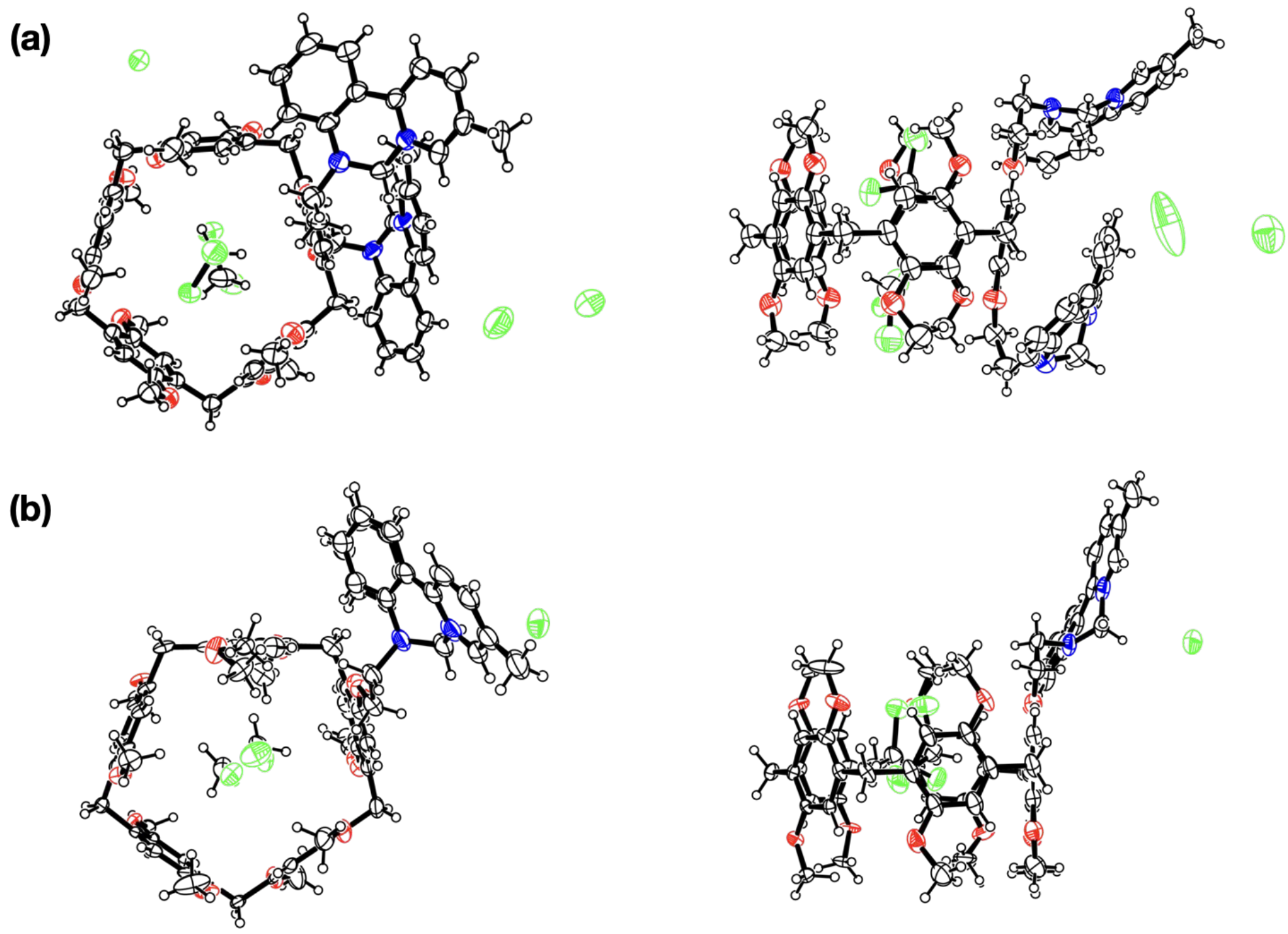

Fig. S1 ORTEP diagrams showing (a) face-on (left) and edge-on (right) views of **1**·Cl_2, (b) face-on (left) and edge-on (right) views of **2**·Cl, with thermal ellipsoids at the 50% probability level.

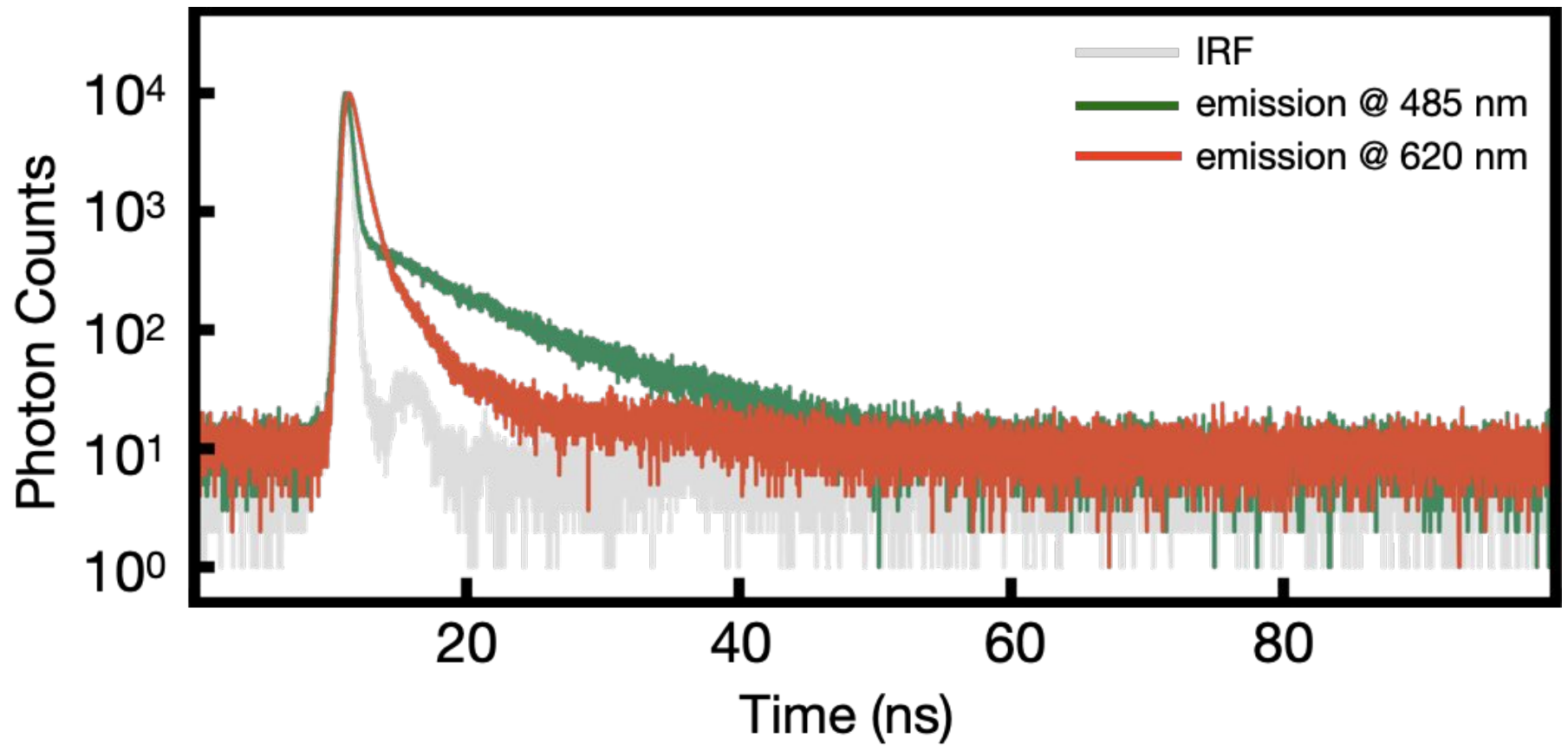

Fig. S2 Fluorescence decay curves of **1**·Cl_2 in MeOH (T = 293 K).

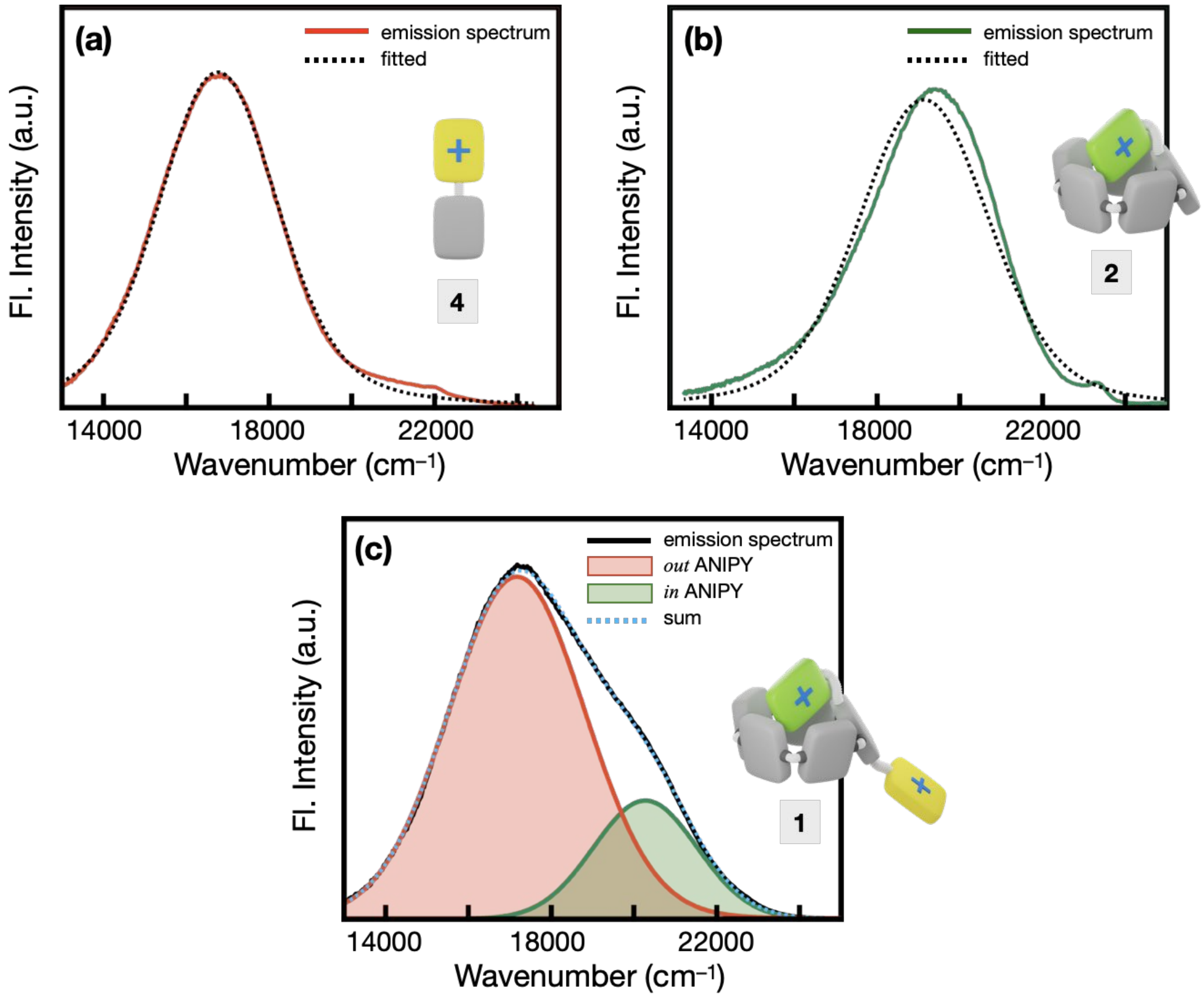

Fig. S3 Experimentally determined emission spectra (solid line) overlaid with fitted curves (dotted lines) for (a) **2** and (b) **4**. (c) Deconvolution analysis performed using two Voigt curves. The solid line is the experimental spectrum of **1**. The blue dotted lines are the sum of two spectral components (green and red).

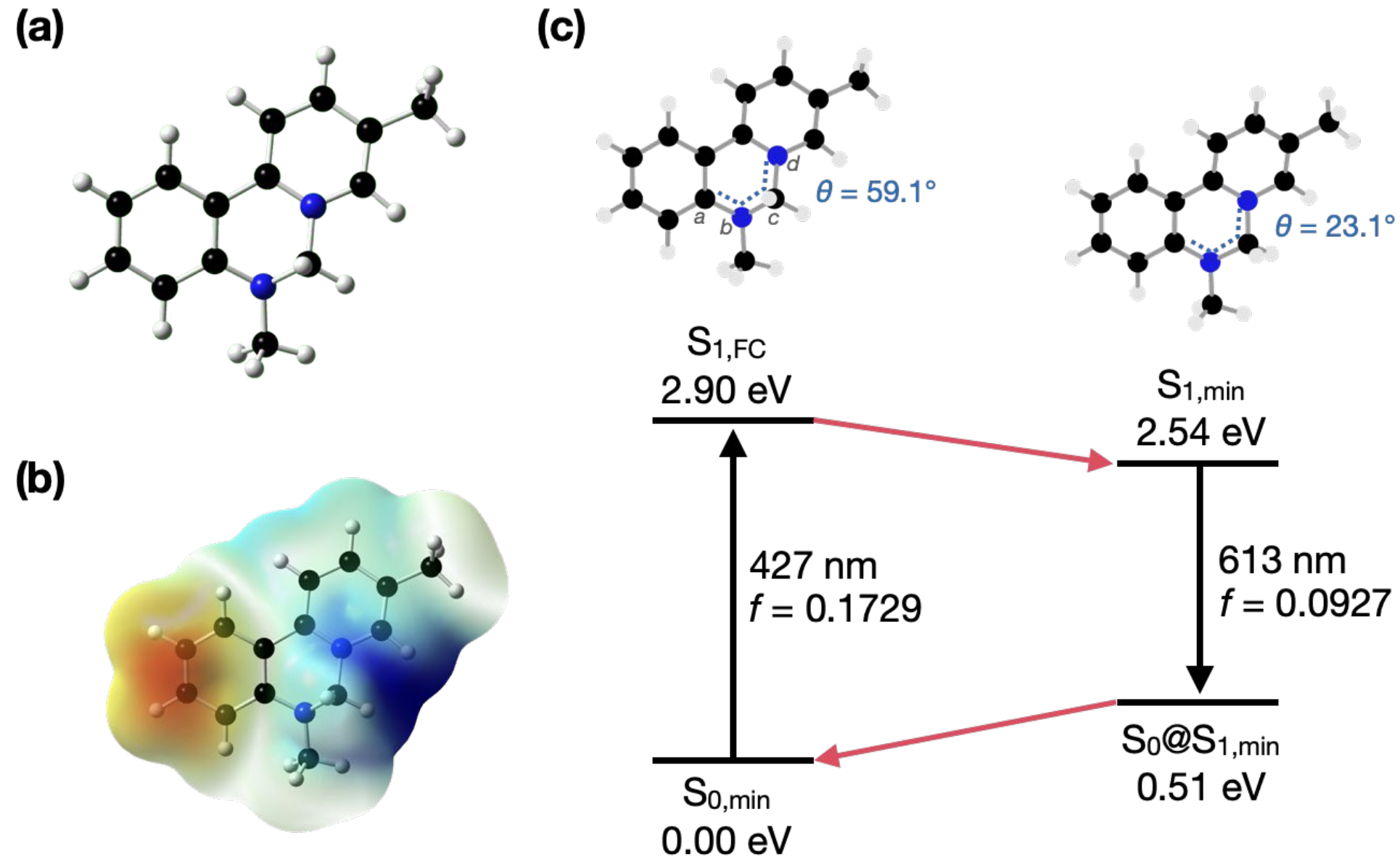

Fig. S4 (a) Energy-minimized geometry of **ANIPY-Me** at $S_{0,\mathrm{min}}$. The dihedral angle θ is defined by the four atoms a, b, c and d. (b) Molecular electrostatic potential (MEP) map of **ANIPY-Me**. (c) Energy level diagram of **ANIPY-Me** calculated by TD-DFT (TD–B3LYP–D3/6-311++G** level of theory) with implicit solvation in MeOH.

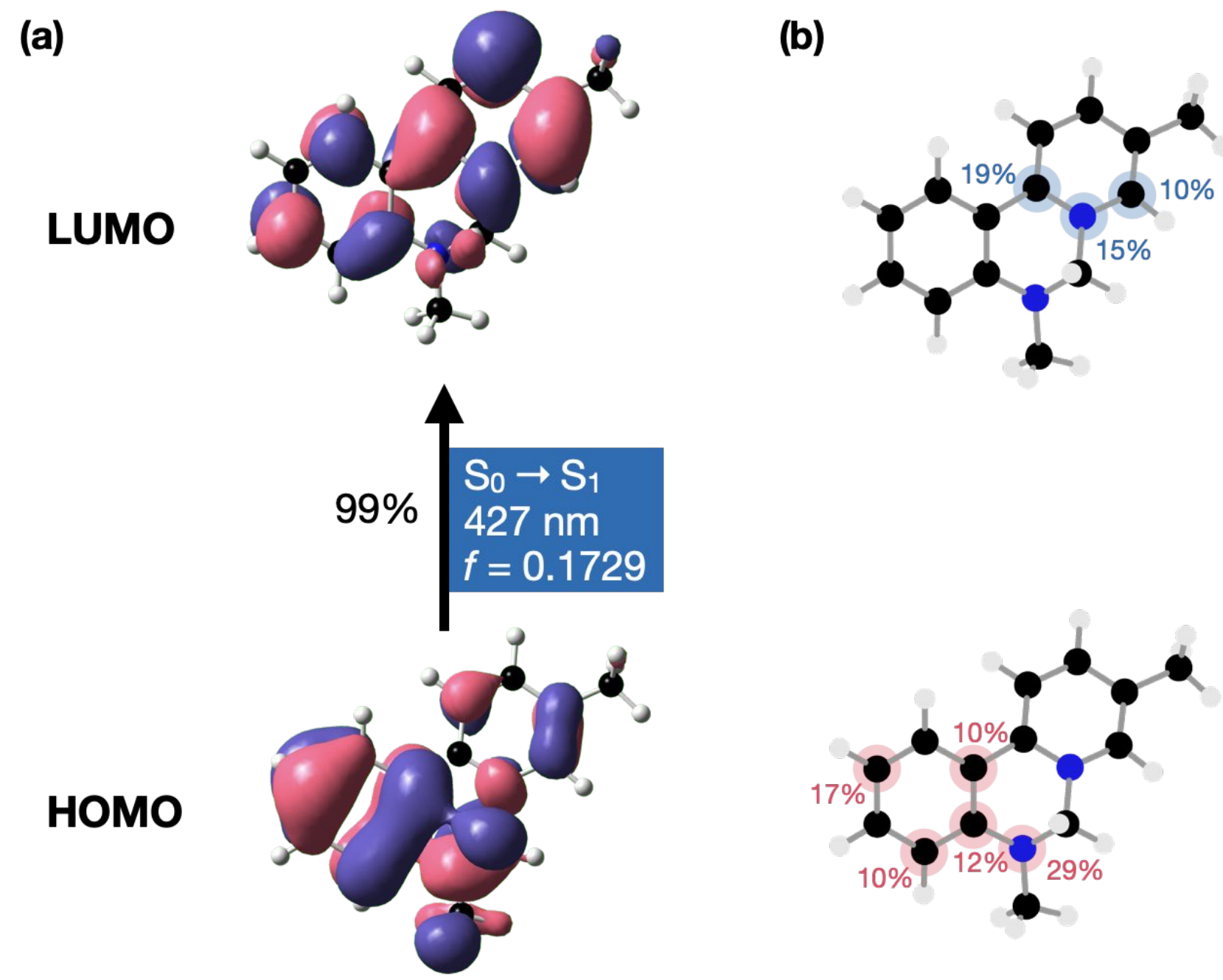

Fig. S5 (a) Frontier molecular orbitals (FMOs) involved in the electronic transition to the S_1 excited state of **ANIPY-Me**. (b) The percent atomic contribution to the FMOs of **ANIPY-Me**; only contributions of $> 10\%$ are shown. The calculation was performed by TD-DFT (TD–B3LYP–D3/6-311++G** level of theory) with implicit solvation in MeOH.

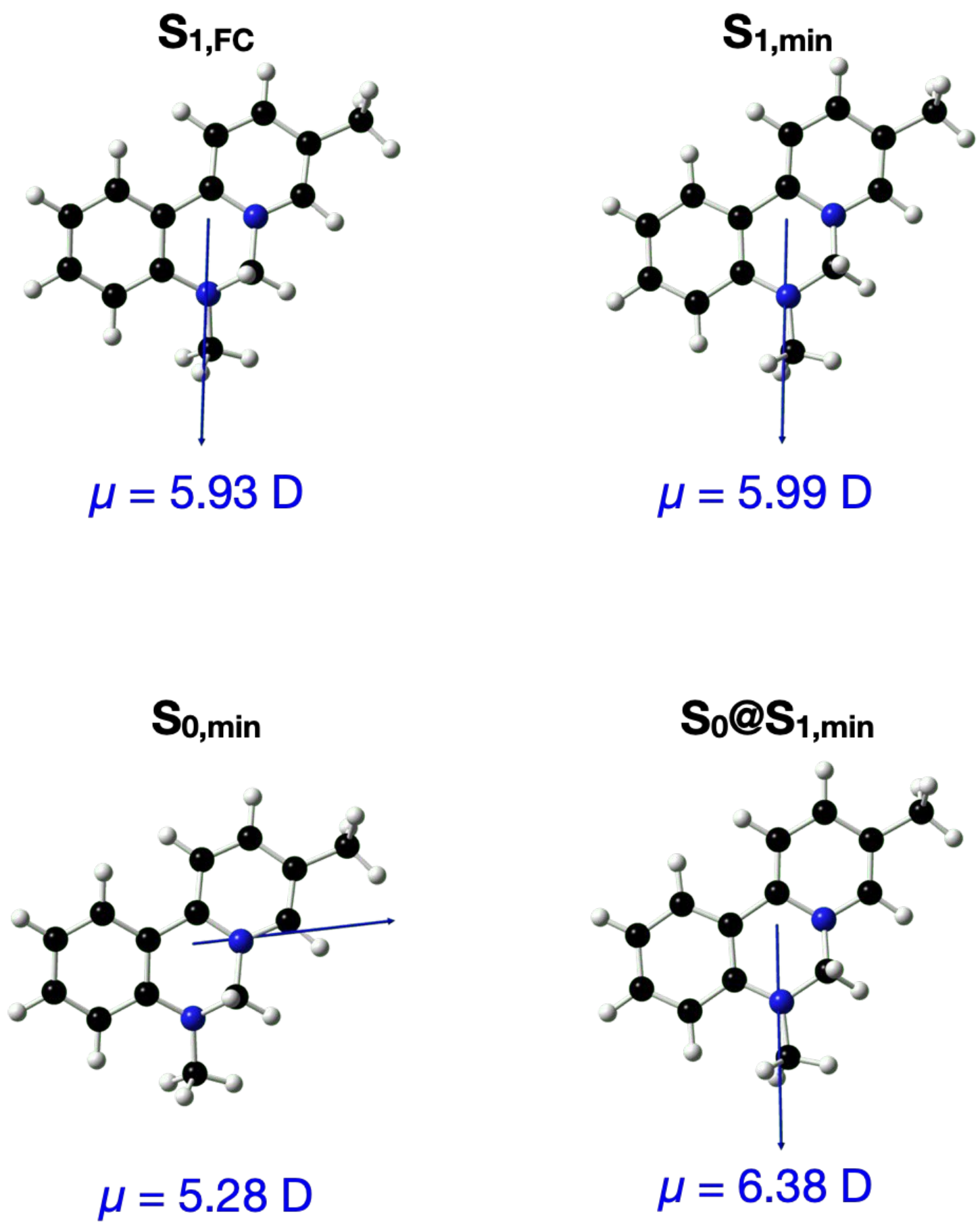

Fig. S6 Ground-state (bottom) and excited-state (top) dipole moments of **ANIPY-Me**. The calculation was performed by TD-DFT (TD–B3LYP–D3/6-311++G** level of theory) with implicit solvation in MeOH.

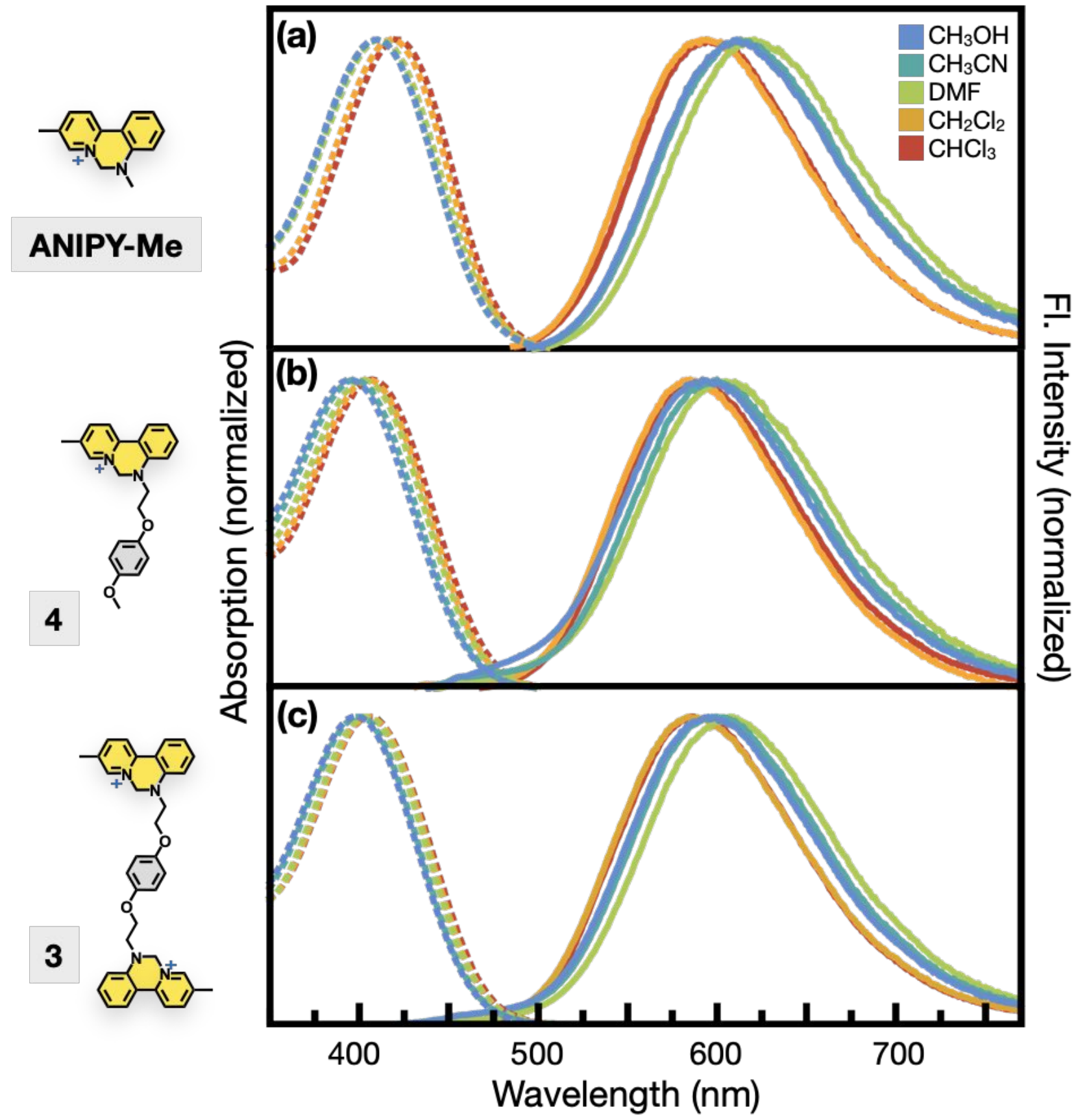

Fig. S7 Normalized absorption and emission spectra of **ANIPY-Me**·Cl, **4**·Cl, and **3**·Cl_2 in different solvents (sample concentration = 0.1 mM; T = 293 K).

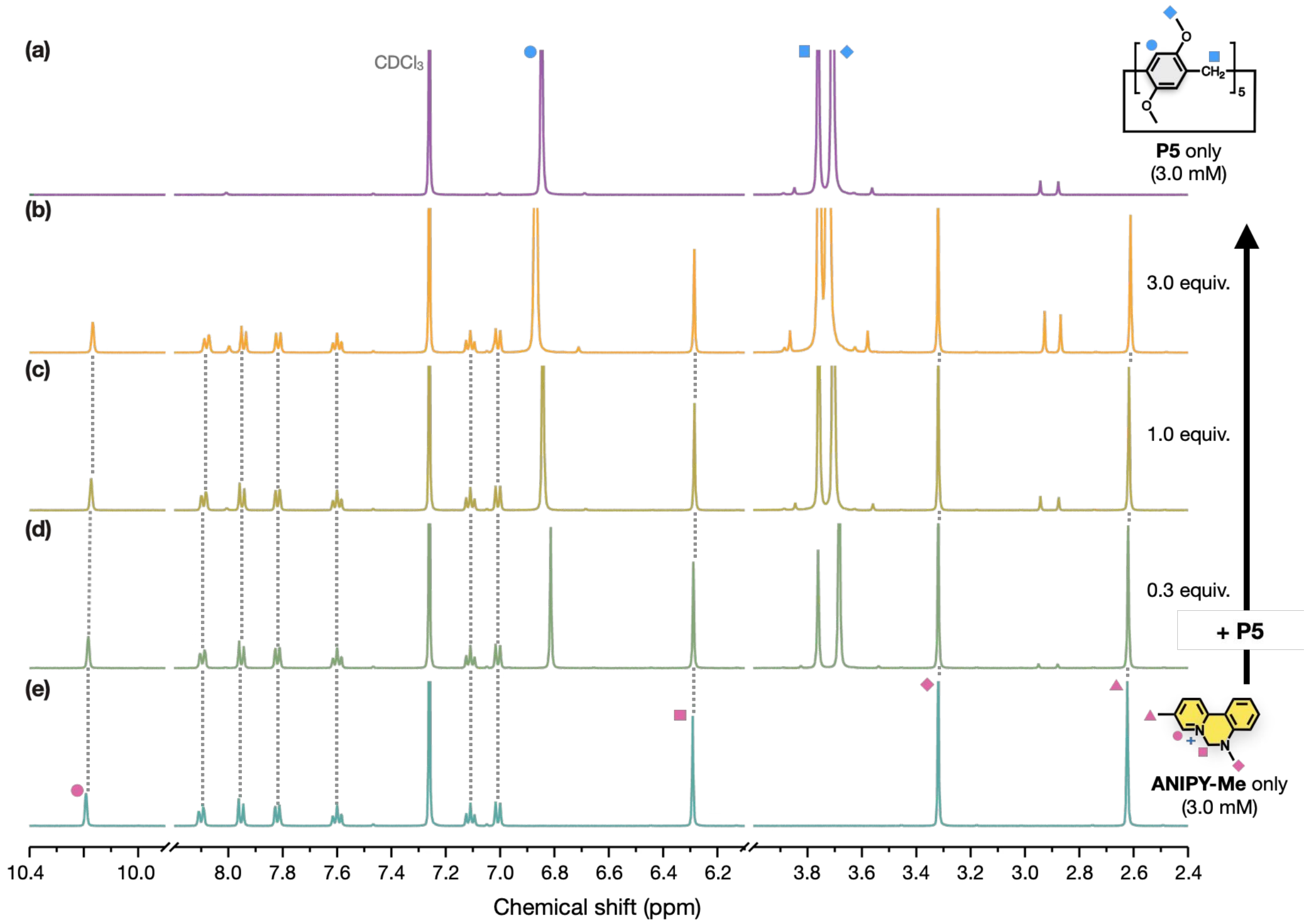

Fig. S8 ^{1}H NMR spectra (500 MHz, $CDCl_3$, T = 293 K) of **ANIPY-Me** (3.0 mM) obtained with varying equivalents of **P5**: (b) 3.0, (c) 1.0, and (d) 0.3 equiv. The spectra of **P5** (3.0 mM) and **ANIPY-Me** (3.0 mM) are shown above (a) and below (e), respectively. Pink and blue symbols denote proton resonances of **ANIPY-Me** and **P5**, respectively.

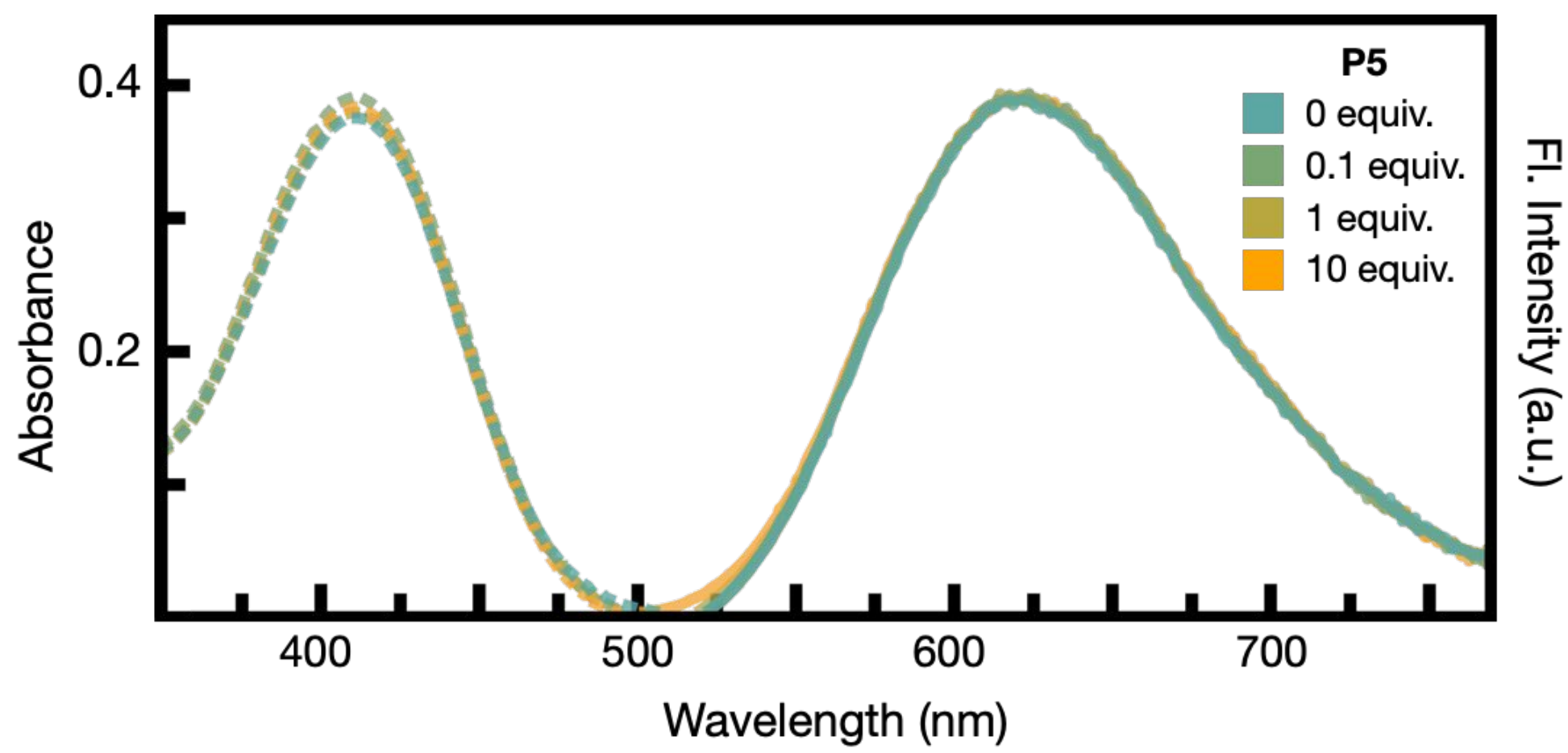

Fig. S9 Absorption and emission spectra ($\lambda_{\mathrm{exc}} = 420$ nm, in DMF, $T = 293$ K) of **ANIPY-Me** (0.1 mM) with 0, 0.1, 1, and 10 equiv of **P5**.

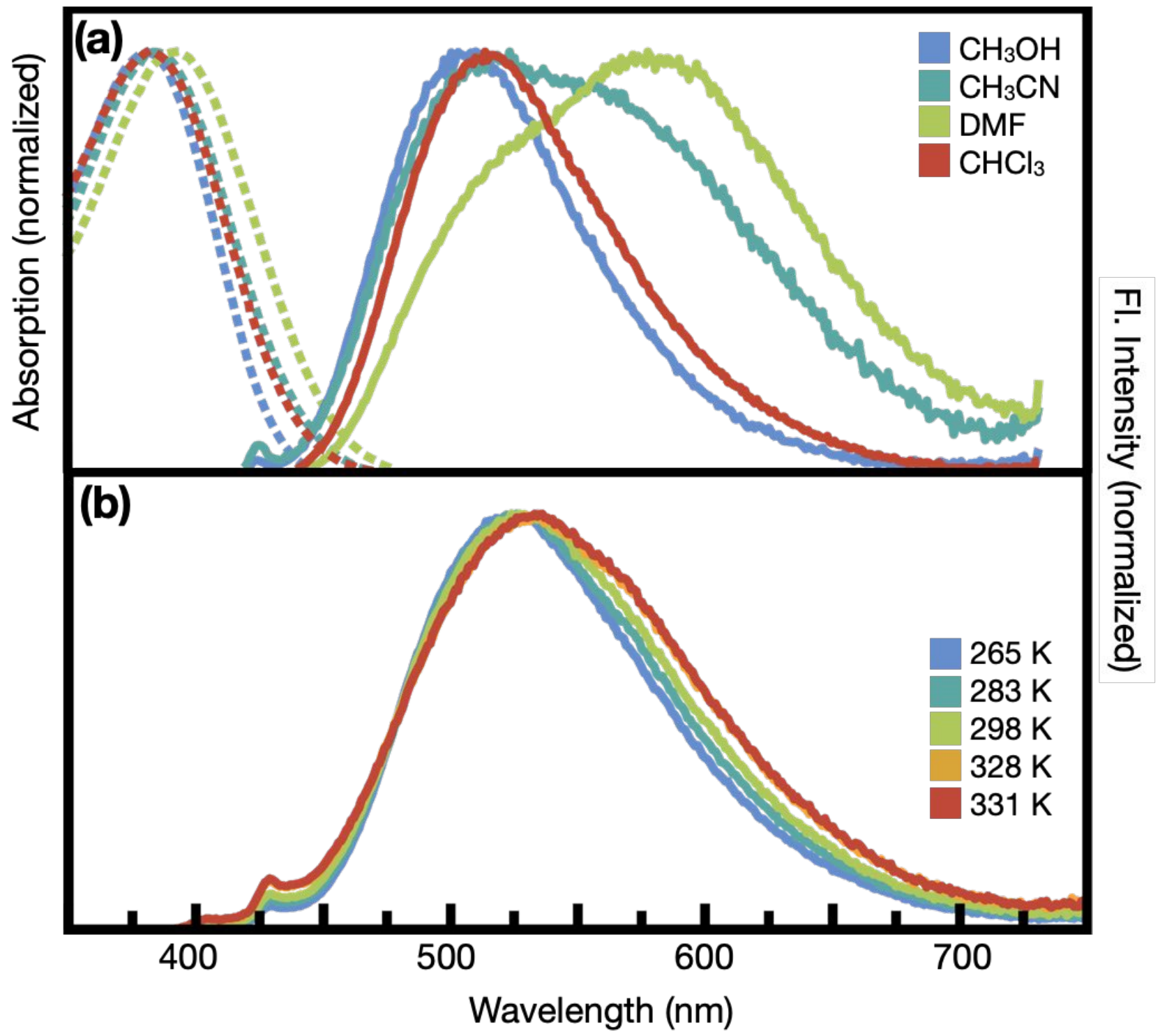

Fig. S10 (a) Solvent-dependent (T = 293 K) and (b) temperature-dependent (in MeOH) absorption (dashed lines) and emission (solid lines) spectra of **2**·Cl (0.1 mM) with λ_{exc} = 380 nm.

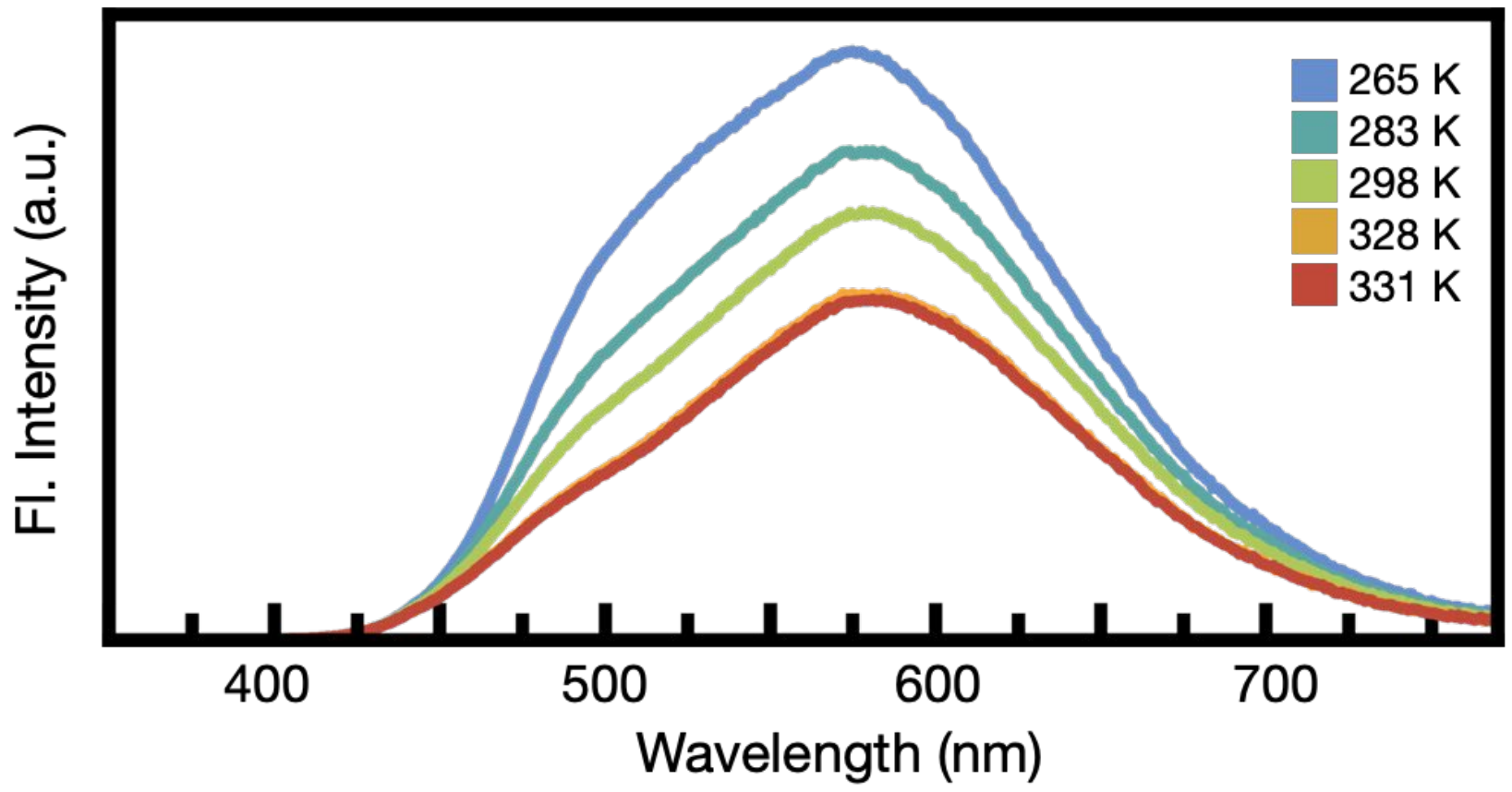

Fig. S11 Temperature-dependent emission spectra (λ_{exc} = 390 nm) of **1**·Cl_2 (0.1 mM) in MeOH from T = 265 to 331 K.

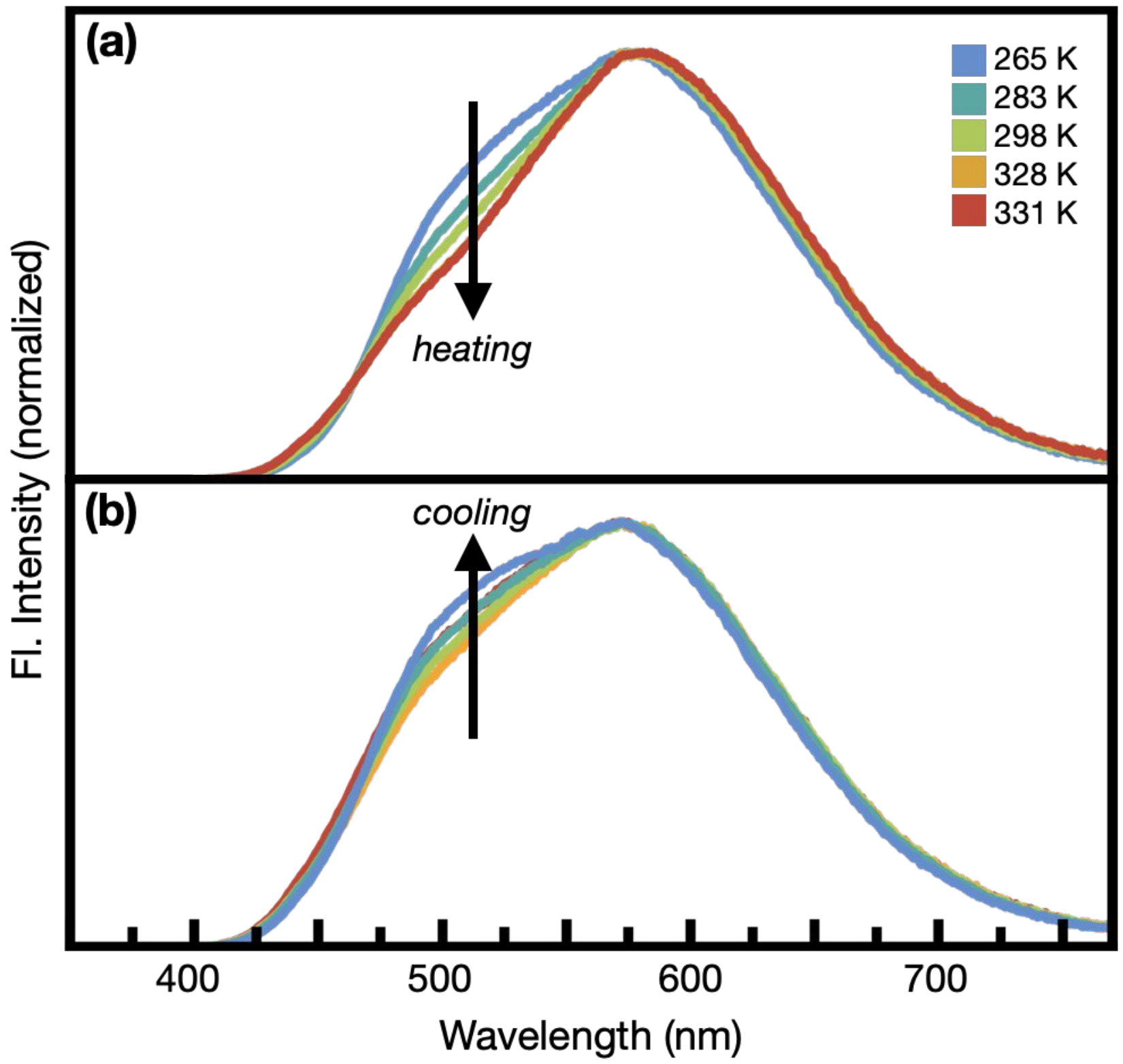

Fig. S12 Temperature-dependent emission spectra of **1**·Cl_2 in MeOH obtained by (a) heating the sample from T = 265 K to 331 K, and (b) cooling the sample from T = 331 K to 265 K (λ_{exc} = 390 nm; sample concentration = 0.1 mM).

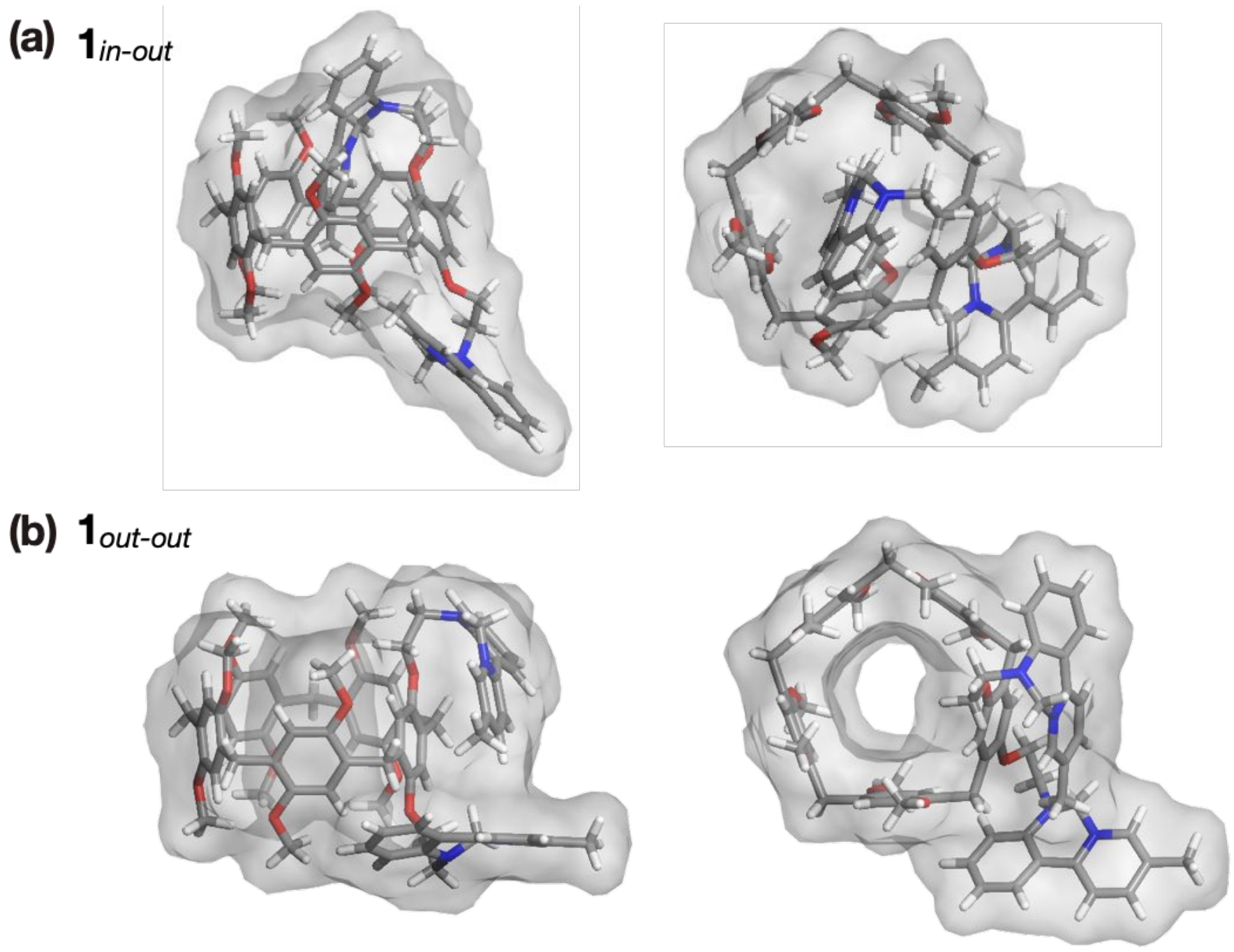

(c)

	Surface area (Å^2)	
Probe Radius (Å)	$\mathbf{1}_{out\text{-}out}$	$\mathbf{1}_{in\text{-}out}$
2.5	894.06	754.77
1.4	915.94	784.43
0.8	954.33	830.36

Fig. S13 Connolly surfaces of the DFT models of (a) $\mathbf{1}_{in\text{-}out}$ and (b) $\mathbf{1}_{out\text{-}out}$ calculated at the B3LYP-D3/6-31G** level of theory with implicit solvation in MeOH, and analyzed with a probe radius of 1.4 Å. (c) A summary of surface areas analyzed by three different probe radii.

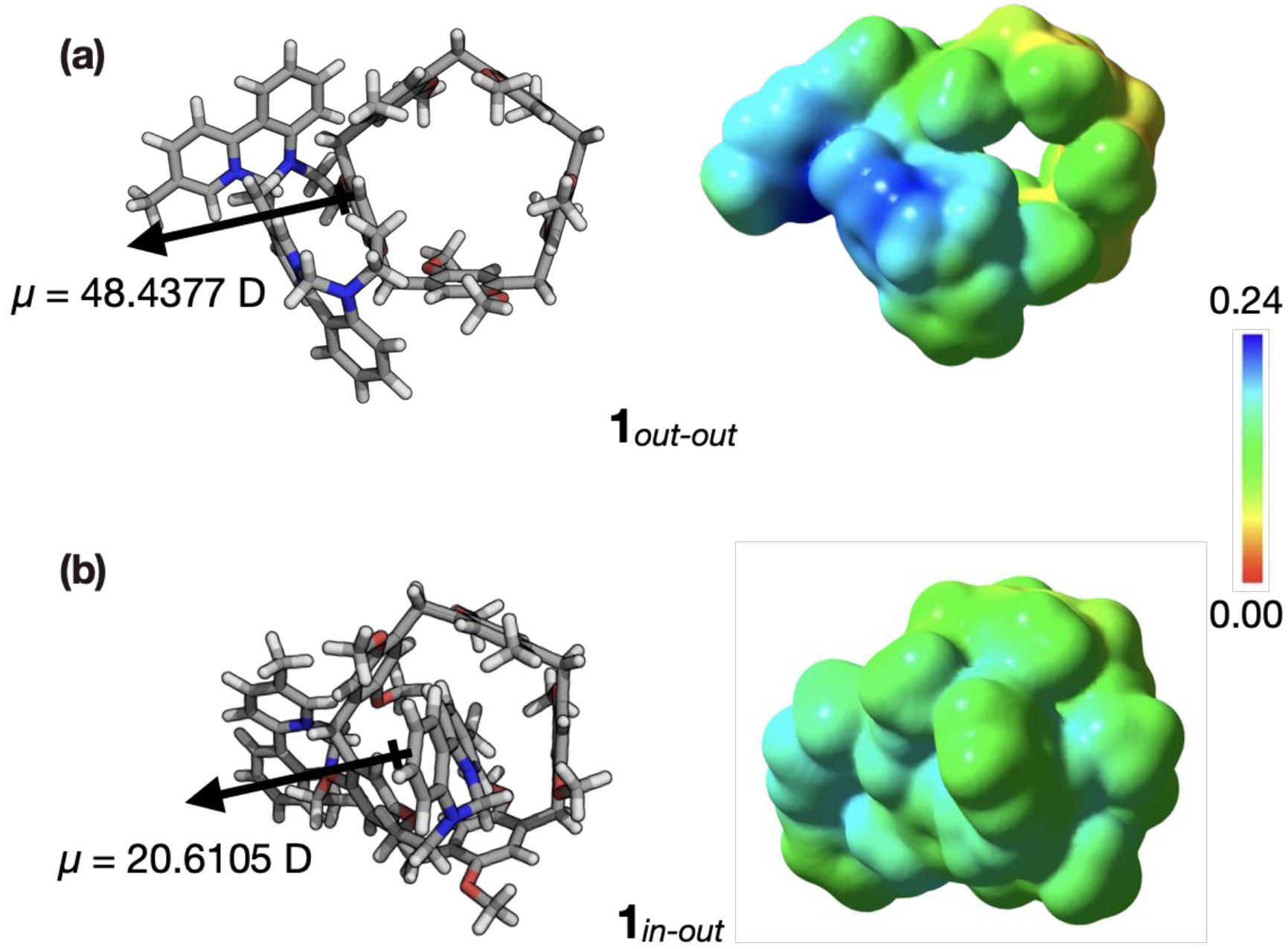

Fig. S14 Molecular electrostatic potential (MEP) maps and dipole moments of (a) $\mathbf{1}_{out\text{-}out}$, and (b) $\mathbf{1}_{in\text{-}out}$ calculated at the B3LYP-D3/6-31G** level of theory with implicit solvation in MeOH.

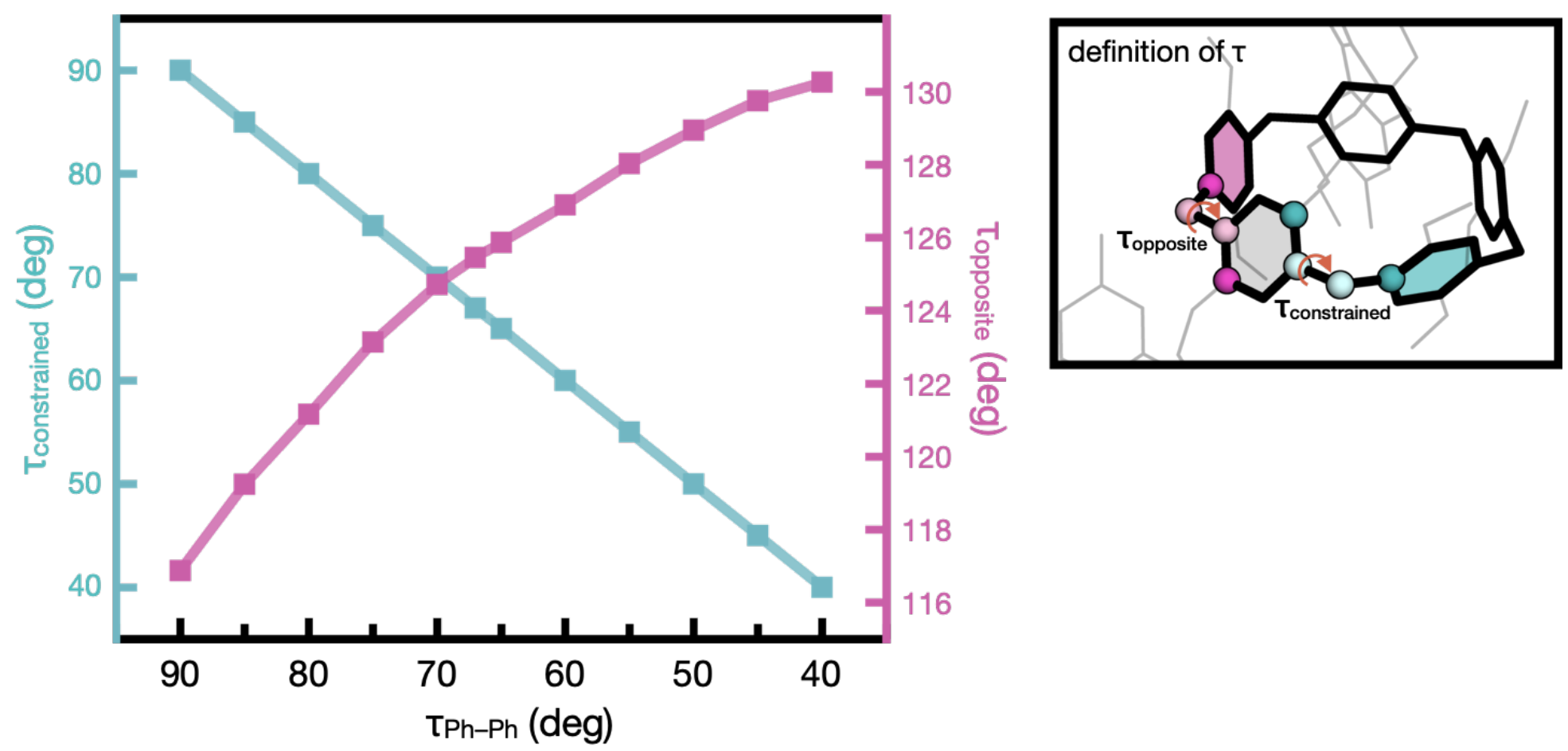

Fig. S15 Correlated movement of the constrained dihedral angle ($\tau_{\text{constrained}}$, cyan) and the dihedral angle on the opposite phenylene panel (τ_{opposite}, pink) as a function of the scan coordinate, computed for the DFT model of **1** (B3LYP-D3/6-31G** level; implicit solvation in MeOH). The definitions of $\tau_{\text{constrained}}$ and τ_{opposite} are provided in the right panel.

(a)

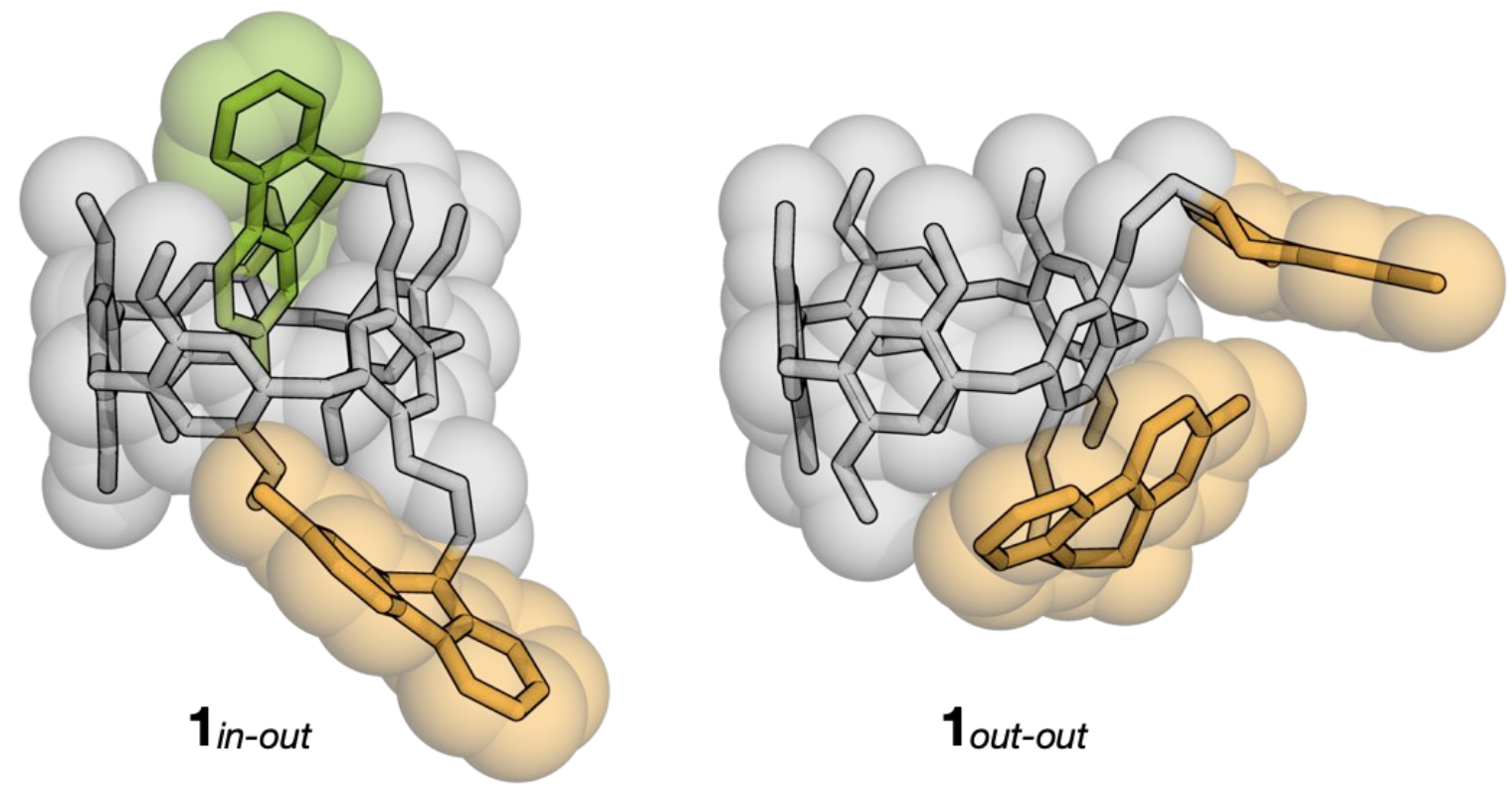

(b)

	$\mathbf{1}_{in\text{-}out}$	$\mathbf{1}_{out\text{-}out}$
CCS Exp. (Å^2)	360	470
CCS Calc. (Å^2)	375	417

Fig. S16 Energy-minimized DFT computational models of (a) $\mathbf{1}_{in\text{-}out}$ and $\mathbf{1}_{out\text{-}out}$. (b) Comparison of the experimental and calculated collision cross section (CCS) values (Å^2) for the corresponding structures shown in (a).

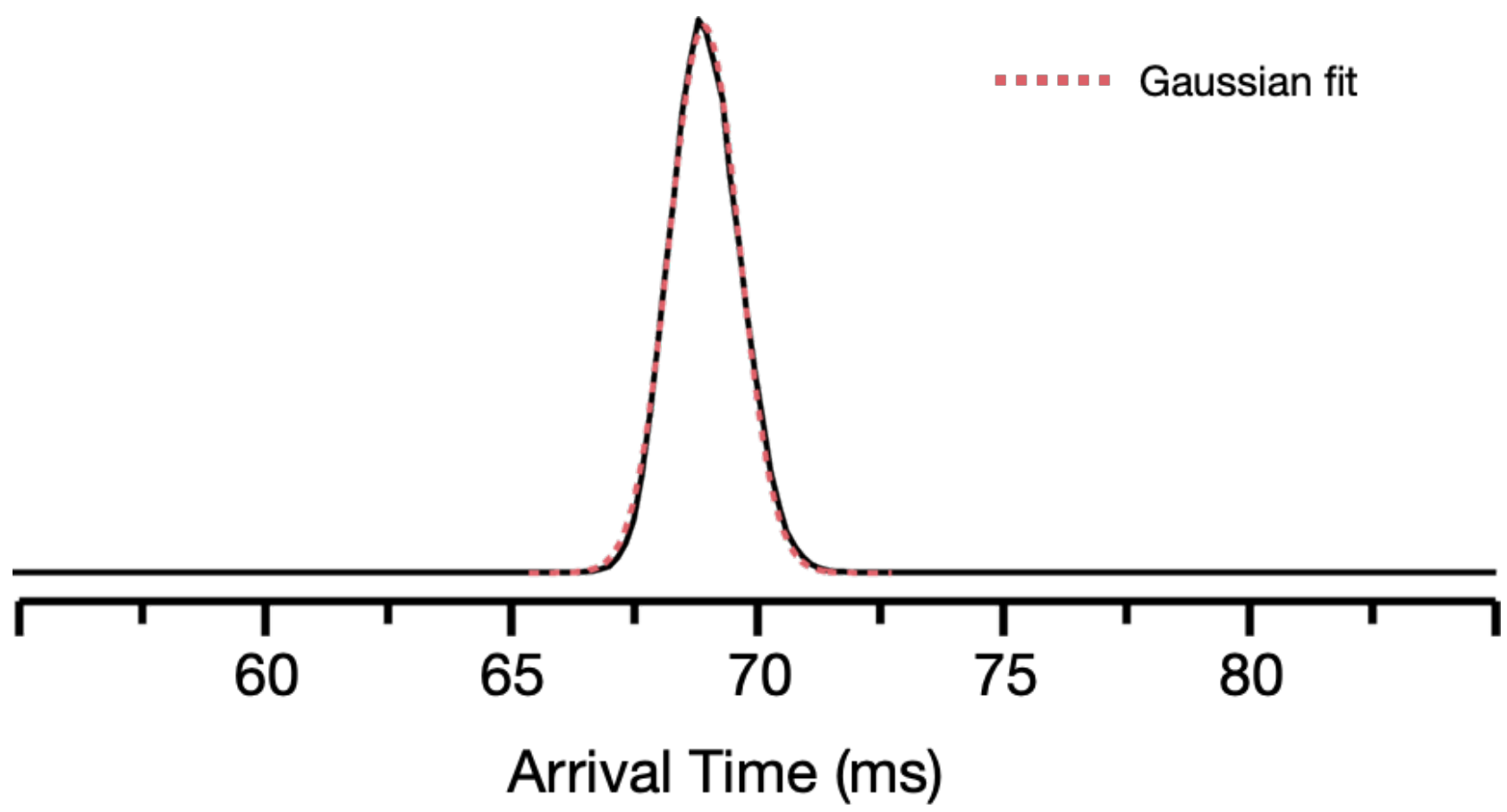

Fig. S17 Arrival time distribution of **2** (solid line), overlaid with a fitted Gaussian curve (red dashed line).

(a)

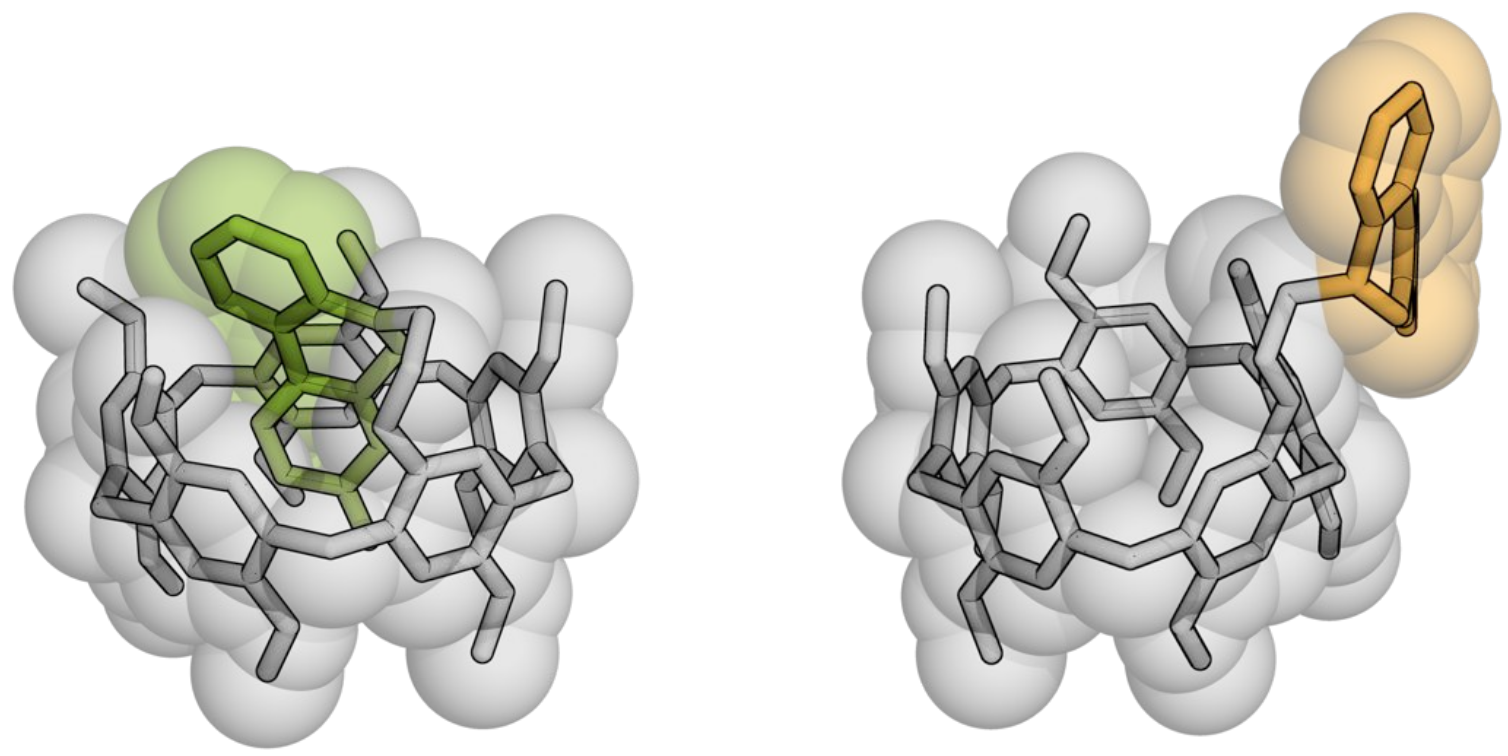

$\mathbf{2}_{in}$ $\mathbf{2}_{out}$

(b)

	$\mathbf{2}_{in}$	$\mathbf{2}_{out}$
CCS Exp. (Å^2)	278	
CCS Calc. (Å^2)	285	302

Fig. S18 Energy-minimized DFT computational models of (a) $\mathbf{2}_{in}$ and $\mathbf{2}_{out}$. (b) Comparison of the experimental and calculated collision cross section (CCS) values (Å^2) for the corresponding structures shown in (a).

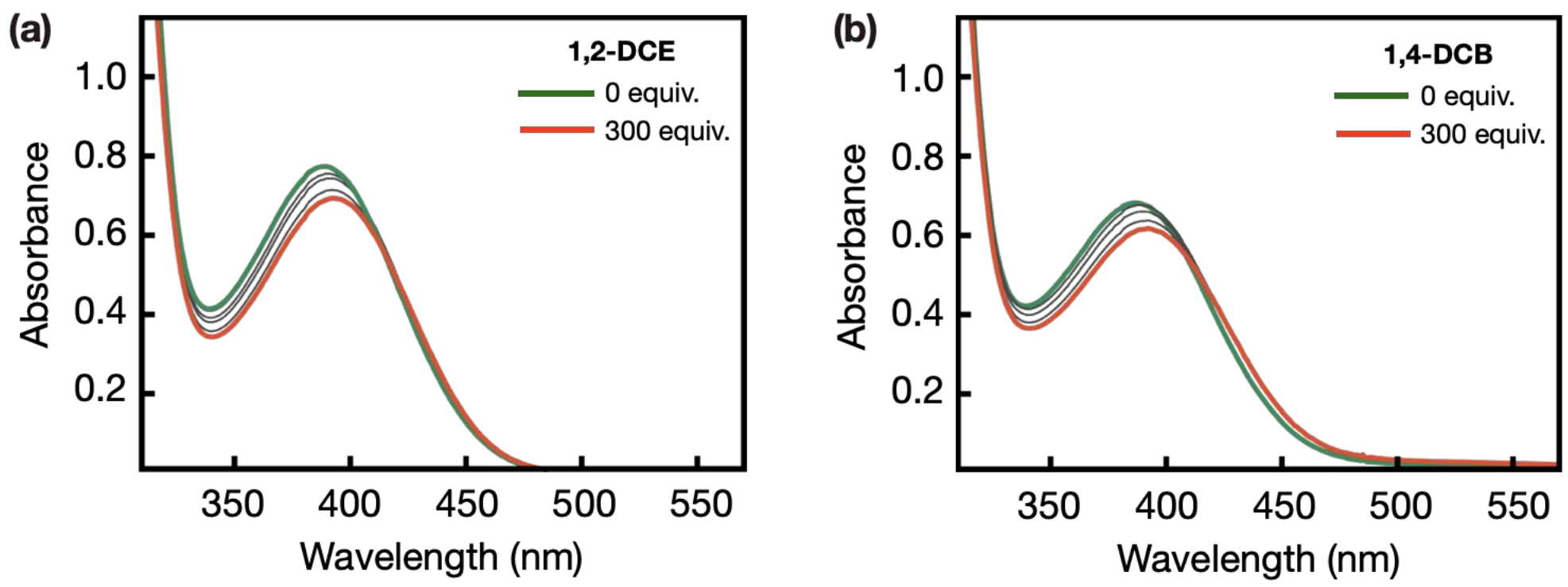

Fig. S19 UV–vis absorption spectral changes of $\mathbf{1}\cdot Cl_2$ (0.1 mM) upon addition of (a) 1,2-DCE, and (b) 1,4-DCB in MeOH at $T = 293$ K.

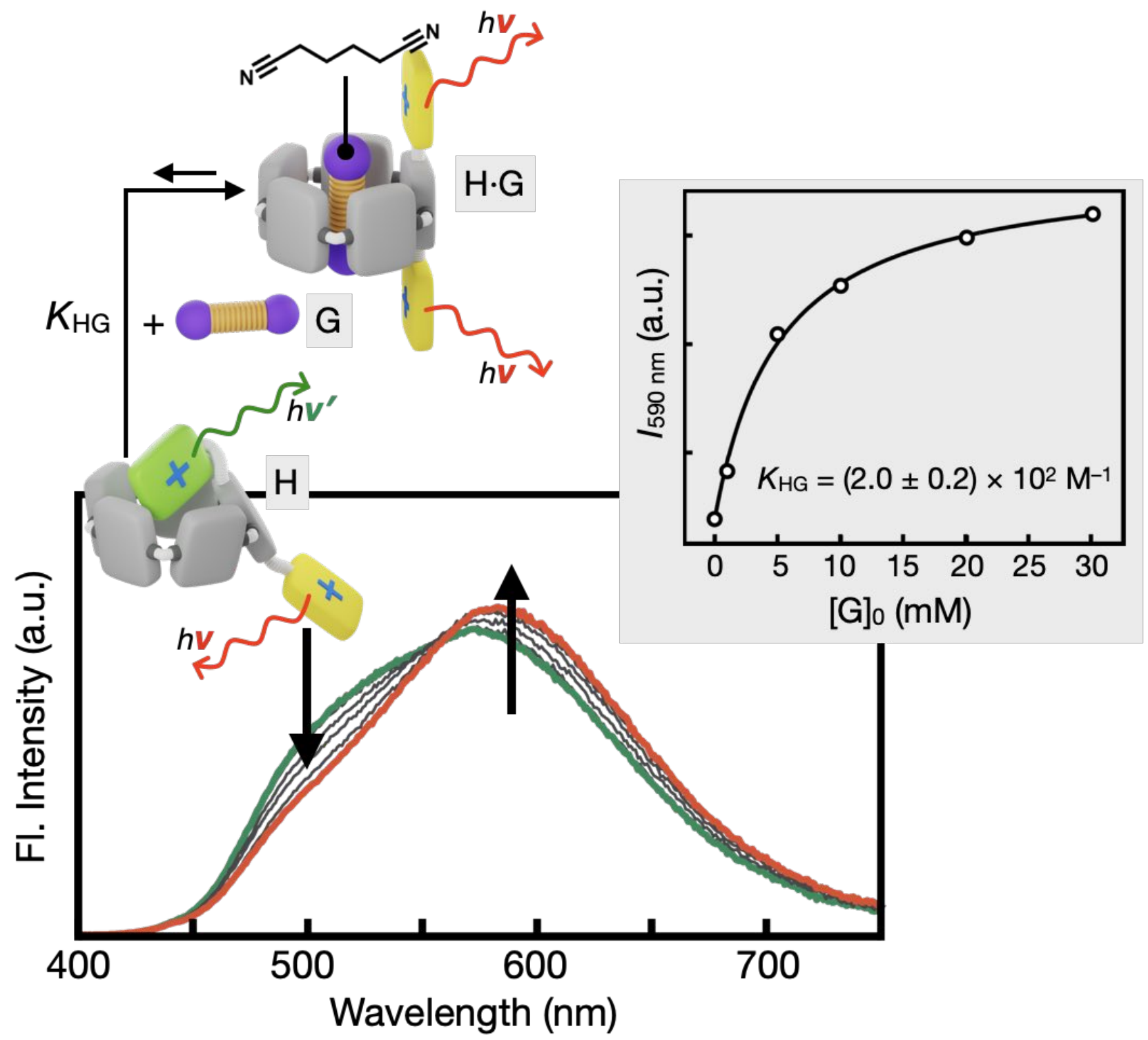

Fig. S20 Fluorescence titration ($\lambda_{\text{exc}} = 390$ nm) of host (**H**) **1** (0.1 mM in MeOH; $T =$ 293 K) with the guest (**G**) 1,4-dicyanobutane (1,4-DCB), fitted to a 1:1 binding isotherm with $K_{\text{HG}} = (2.0\ \pm 0.2) \times 10^2\ \text{M}^{-1}$, using changes in the fluorescence intensity at $\lambda =$ 590 nm ($I_{590\,nm}$).

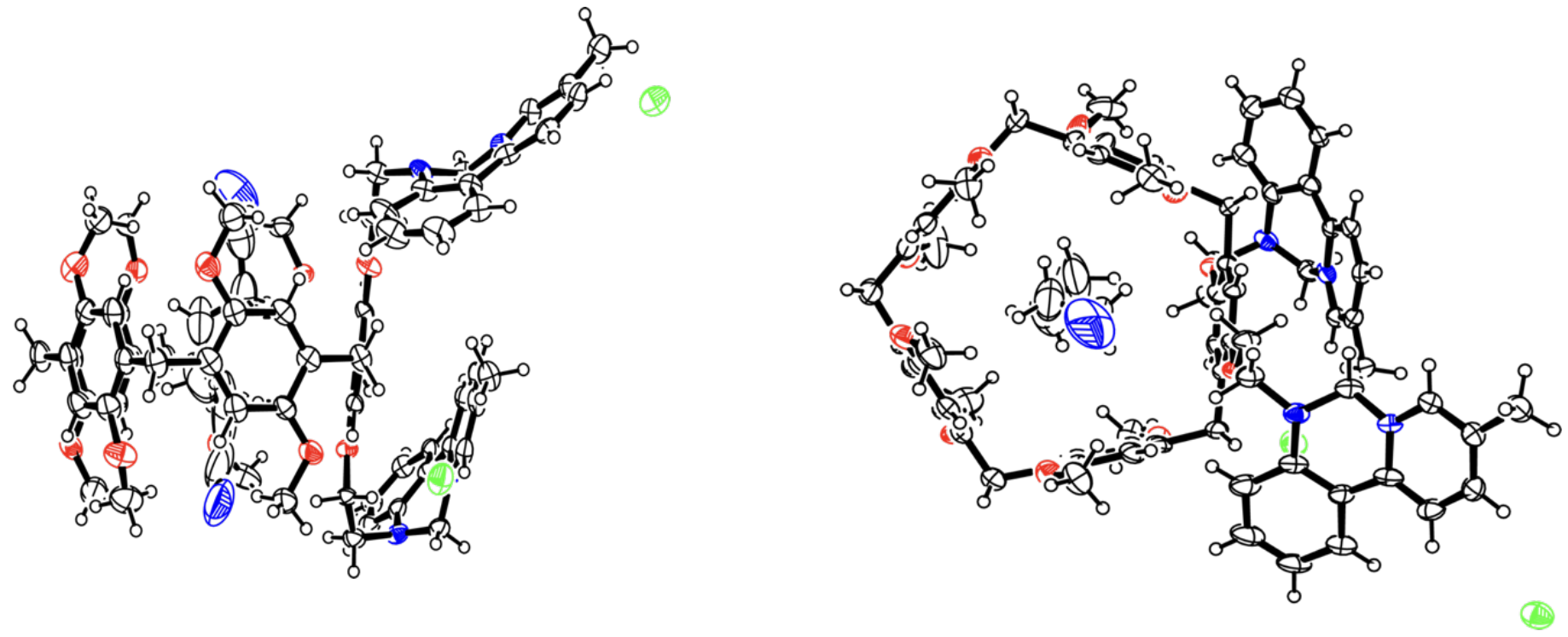

edge-on view face-on view

Fig. S21 ORTEP diagrams showing edge-on (left) and face-on (right) views of [**1** $\supset$ 1,4-DCB]·Cl_2 with thermal ellipsoids at the 50% probability level.

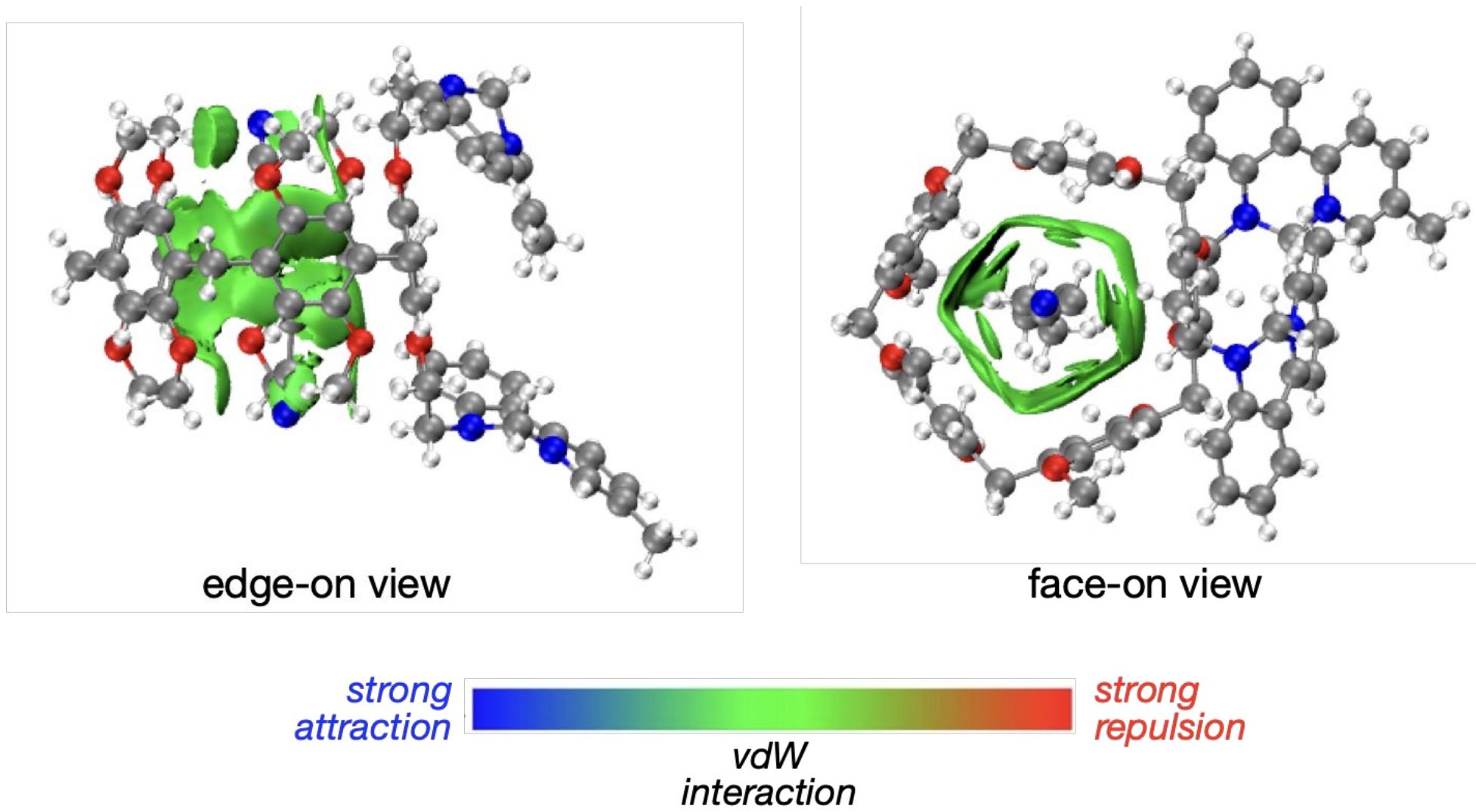

Fig. S22 IGMH analysis of [**1** ⊃ 1,4-DCB]$^{2+}$ with an isosurface for $\delta_g{}^{\text{inter}} = 0.002$ a.u. The green-colored regions indicate dispersive intermolecular vdW contacts without significant attractive (blue) or repulsive (red) interactions.

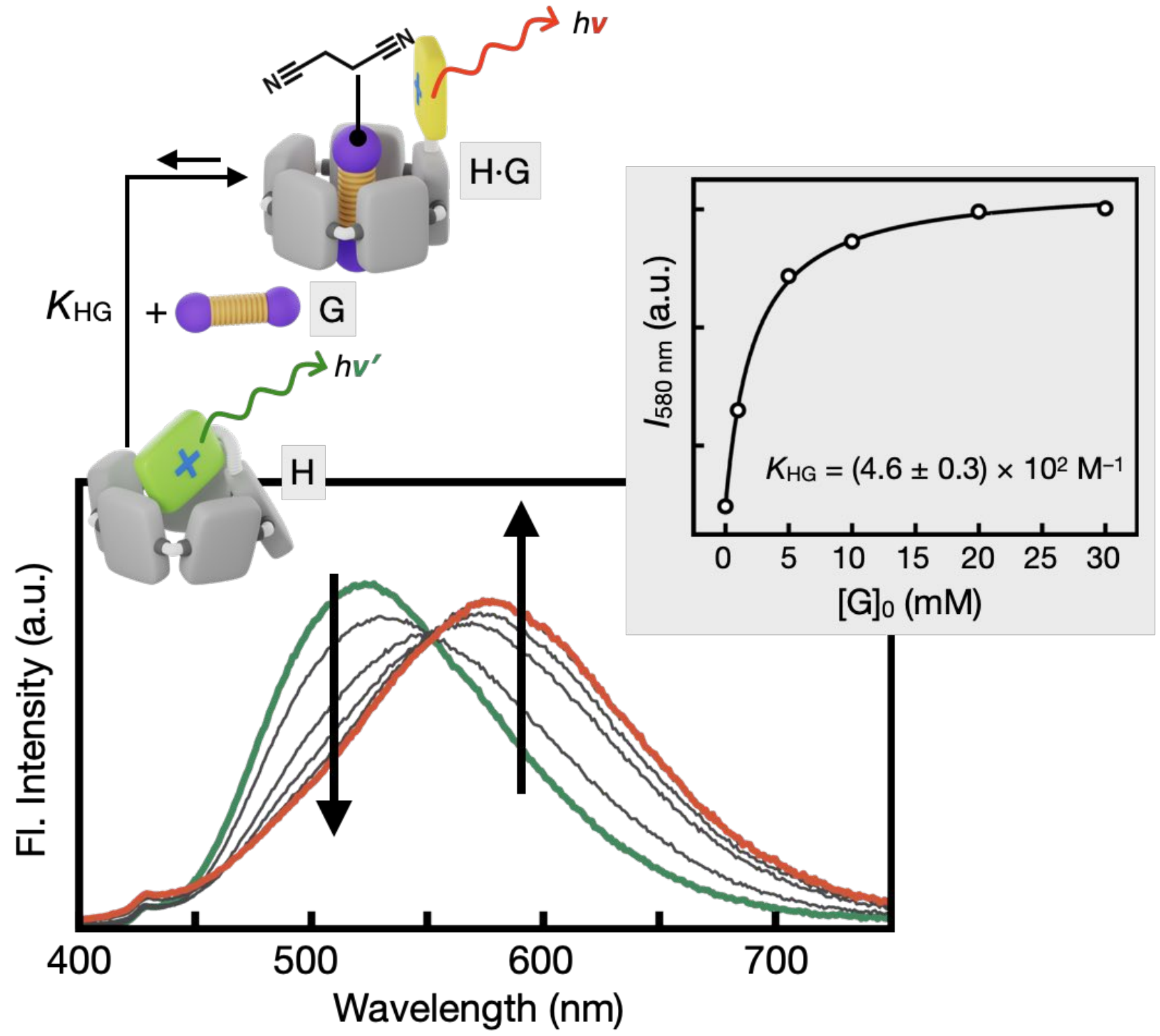

Fig. S23 Fluorescence titration (λ_{exc} = 380 nm) of host (**H**) **2** (0.1 mM in MeOH; T = 293 K) with the guest (**G**) 1,2-dicyanoethane (1,2-DCE), fitted to a 1:1 binding isotherm with $K_{HG} = (4.6 \pm 0.3) \times 10^2$ M^{-1}, using changes in the fluorescence intensity at λ = 580 nm ($I_{580\,nm}$).

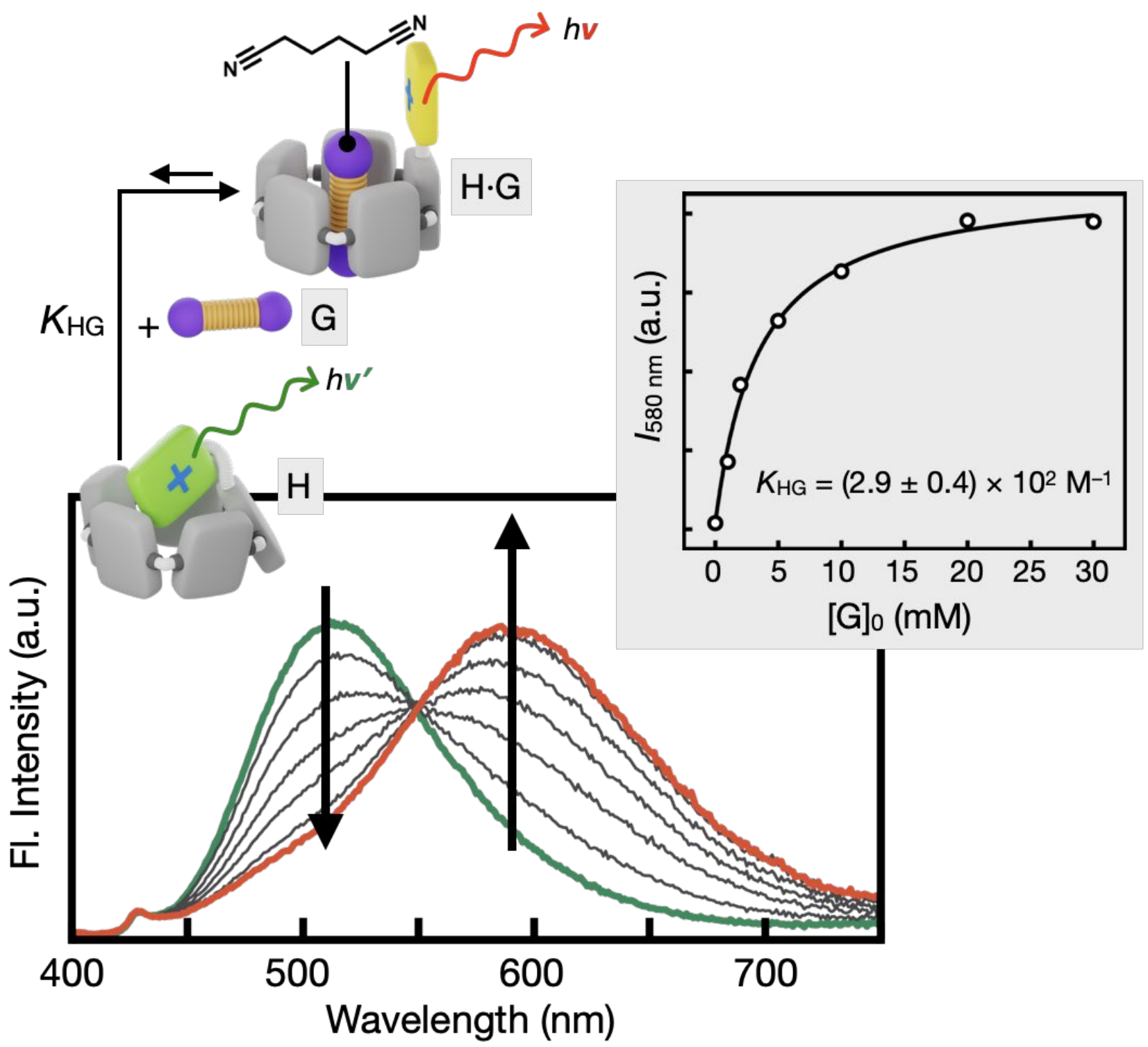

Fig. S24 Fluorescence titration (λ_{exc} = 380 nm) of host (**H**) **2** (0.1 mM in MeOH; T = 293 K) with the guest (**G**) 1,4-dicyanobutane (1,4-DCB), fitted to a 1:1 binding isotherm with $K_{HG} = (2.9\ \pm 0.4) \times 10^2\ M^{-1}$, using changes in the fluorescence intensity at λ = 580 nm ($I_{580\,nm}$).

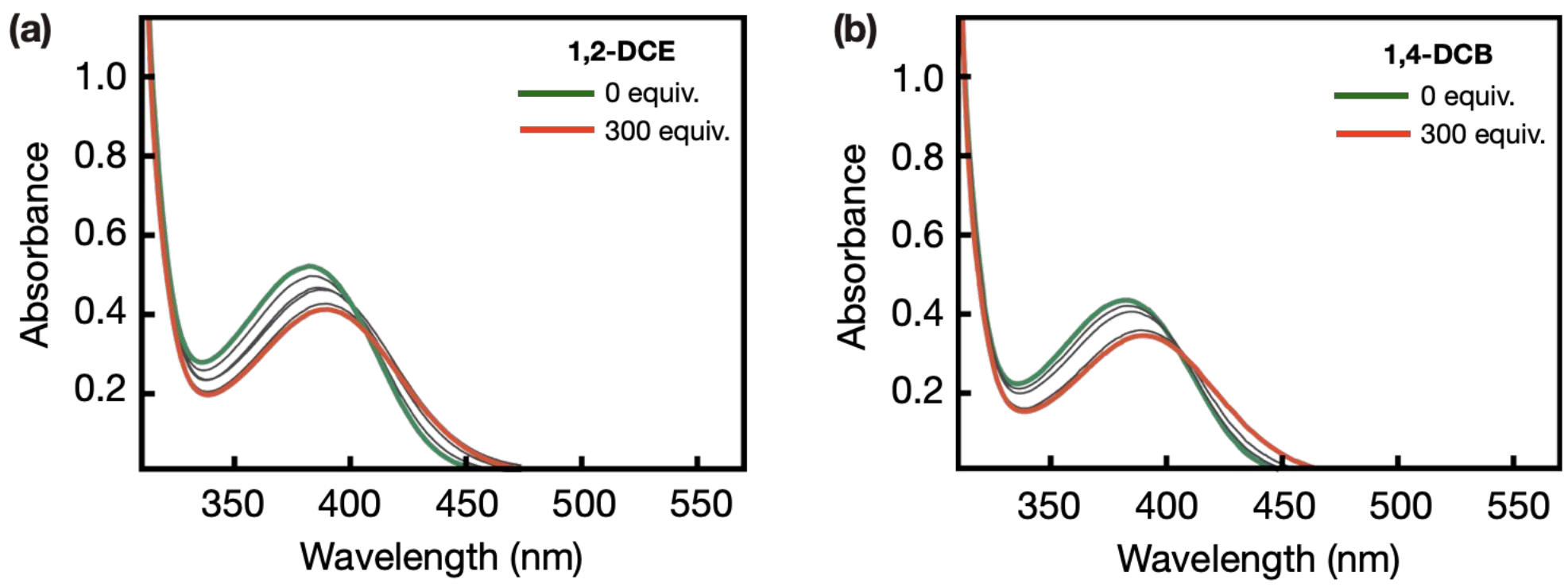

Fig. S25 UV–vis absorption spectral changes of **2**·Cl (0.1 mM) upon addition of (a) 1,2-DCE, and (b) 1,4-DCB in MeOH at $T = 293$ K.

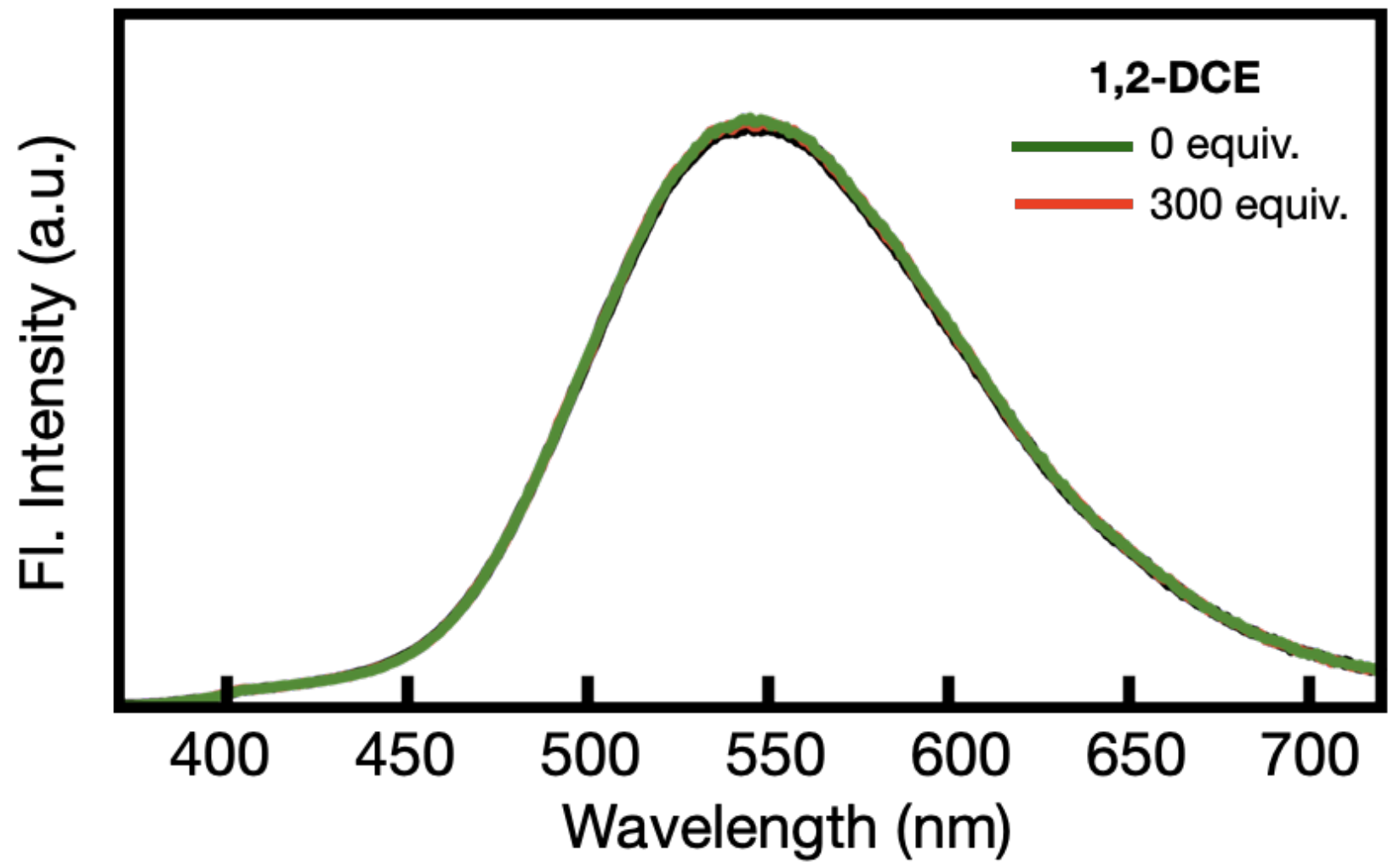

Fig. S26 Fluorescence spectral change (λ_{exc} = 400 nm) of **3**·Cl_2 (0.1 mM) in MeOH upon addition of 1,2-DCE at T = 293 K.

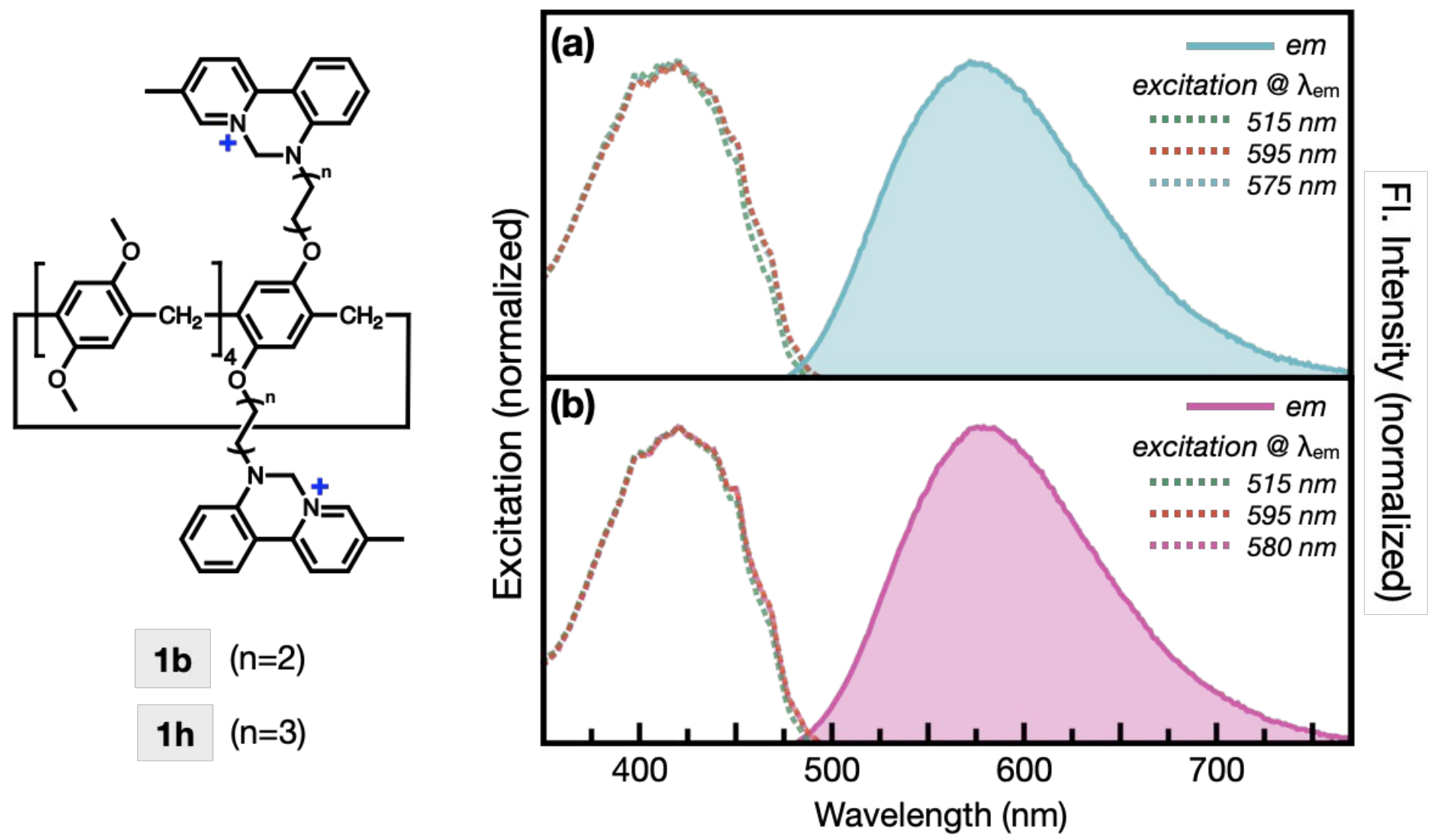

Fig. S27 Normalized excitation and emission spectra of (a) **1b**·Cl_2 and (b) **1h**·Cl_2 in MeOH (sample concentration = 0.1 mM) at T = 293 K.

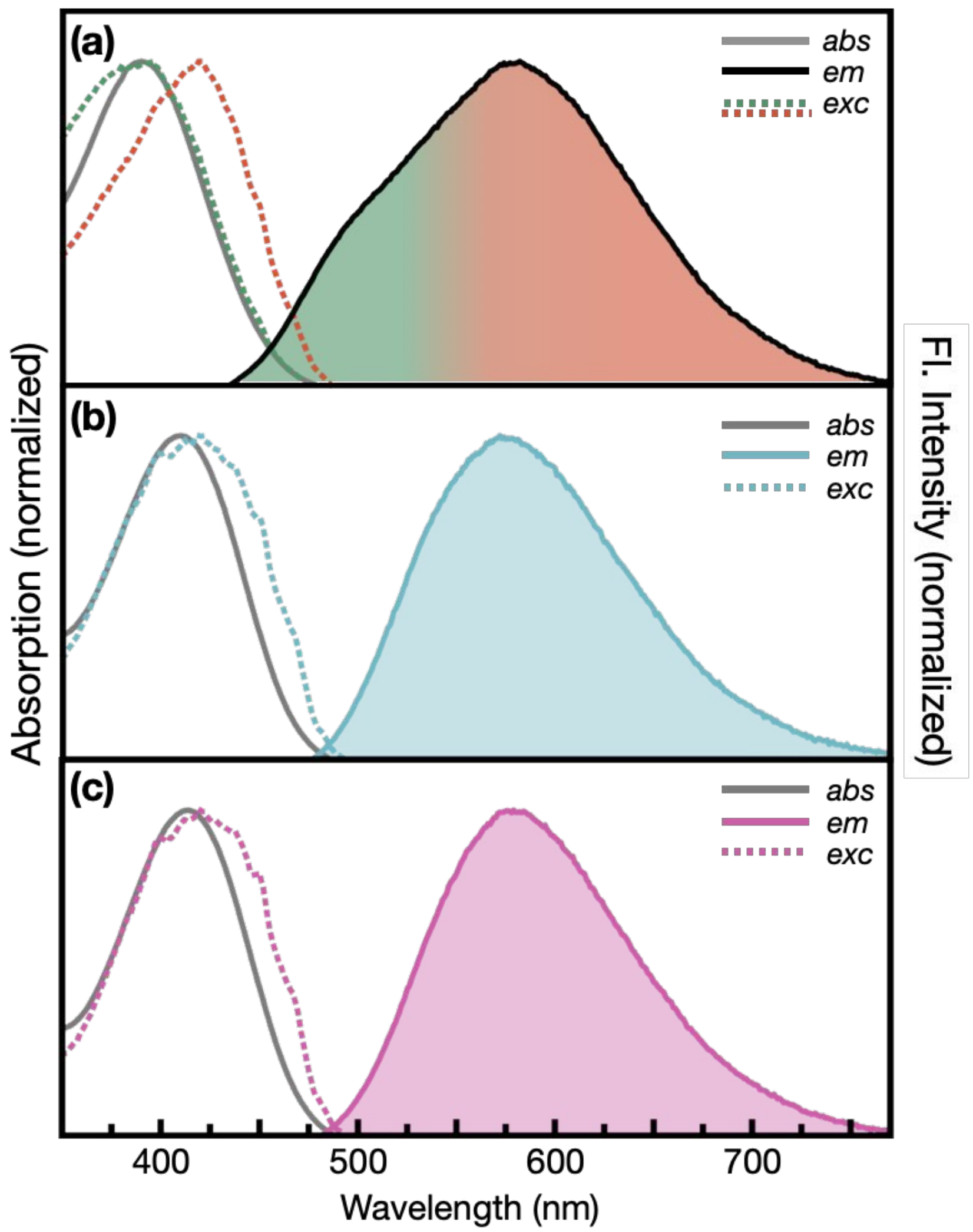

Fig. S28 Stacked spectra (absoption, excitation, and emission) of (a) **1**·Cl_2 (b) **1b**·Cl_2 and (c) **1h**·Cl_2 in MeOH (sample concentration = 0.1 mM) at T = 293 K.

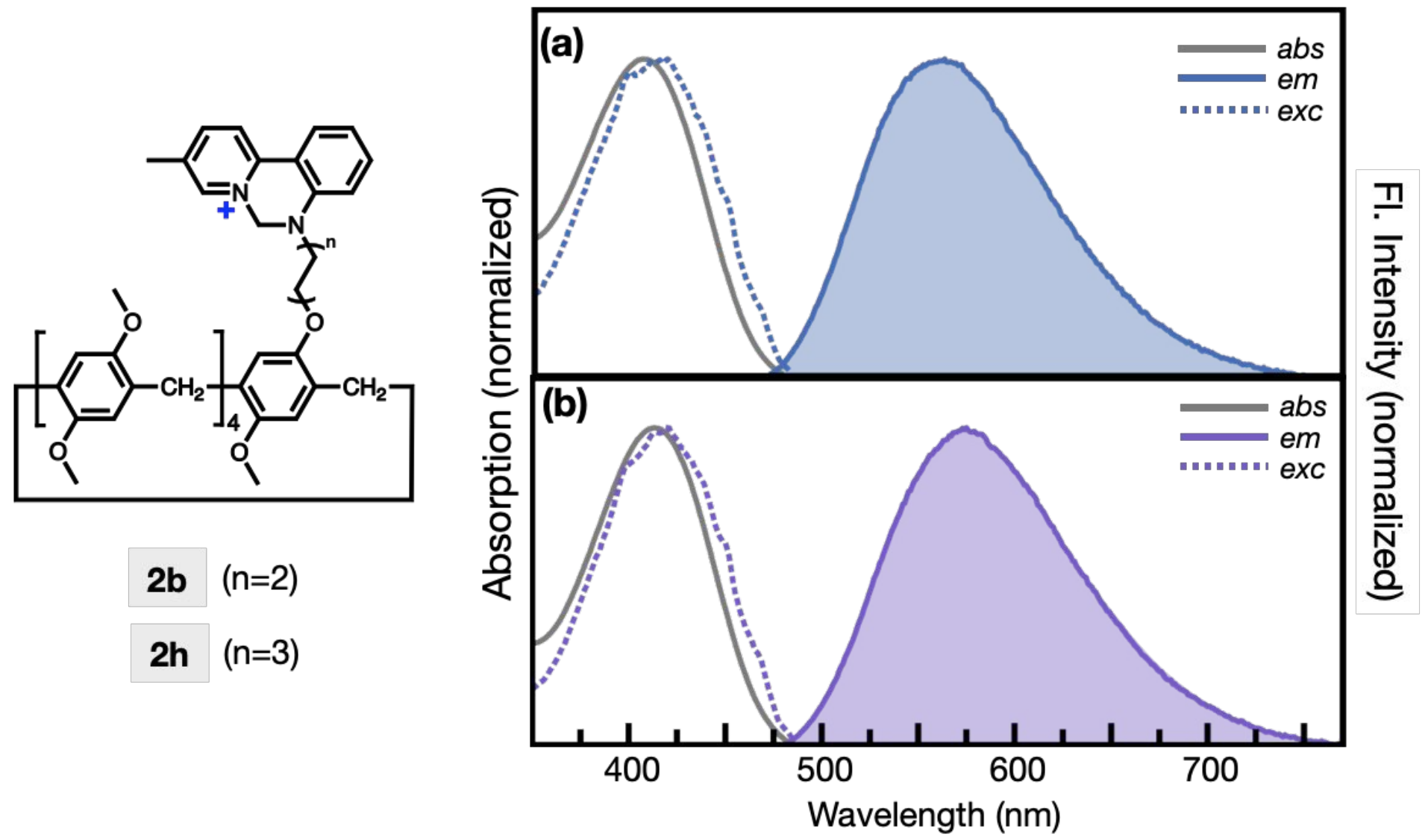

Fig. S29 Normalized absorption, excitation, and emission spectra of (a) **2b**·Cl and (b) **2h**·Cl in MeOH (sample concentration = 0.1 mM) at T = 293 K.

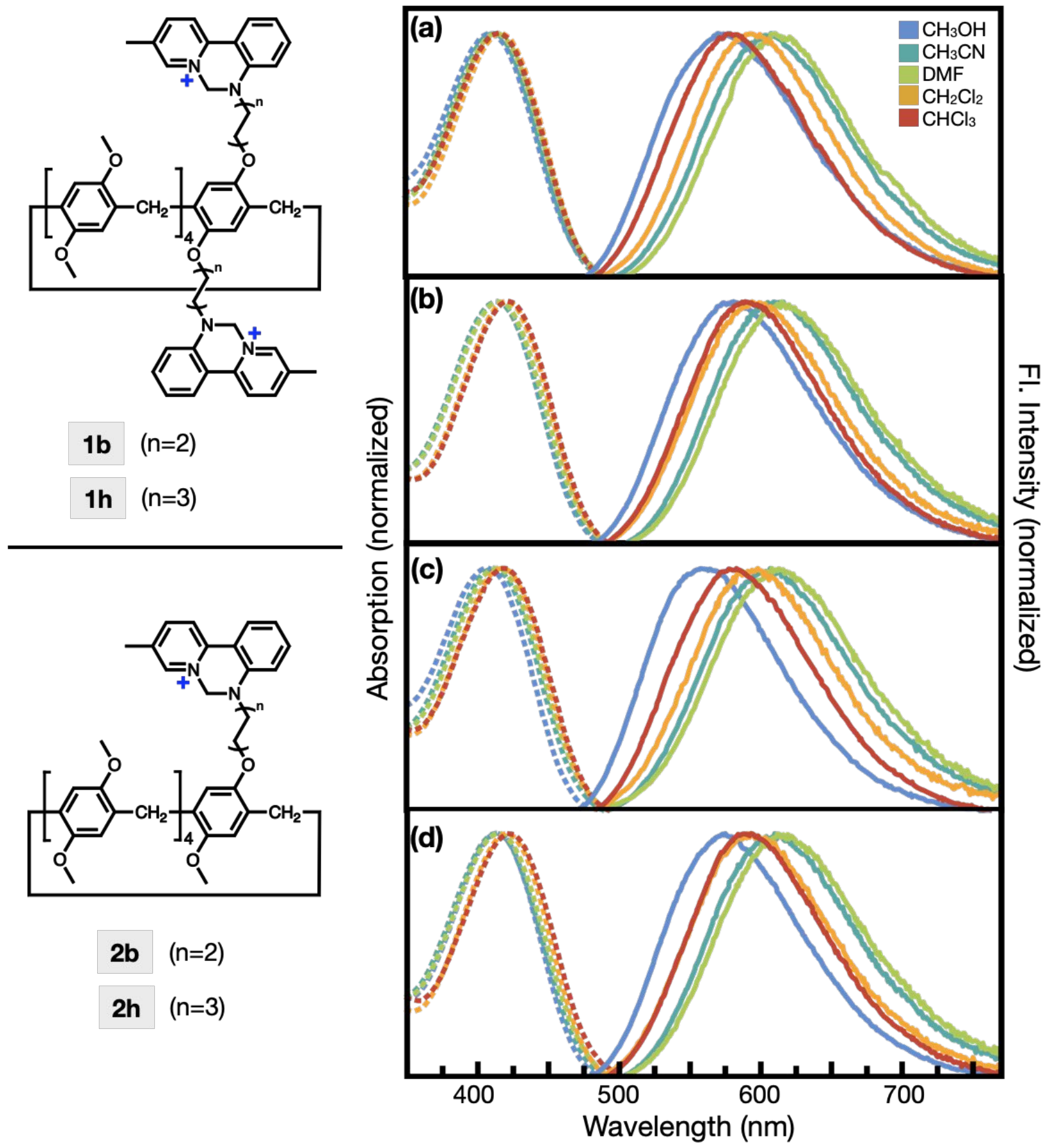

Fig. S30 Normalized absorption and emission spectra of (a) **1b**·Cl_2, (b) **1h**·Cl_2, (c) **2b**·Cl, and (d) **2h**·Cl in different solvents (sample concentration = 0.1 mM; T = 293 K).

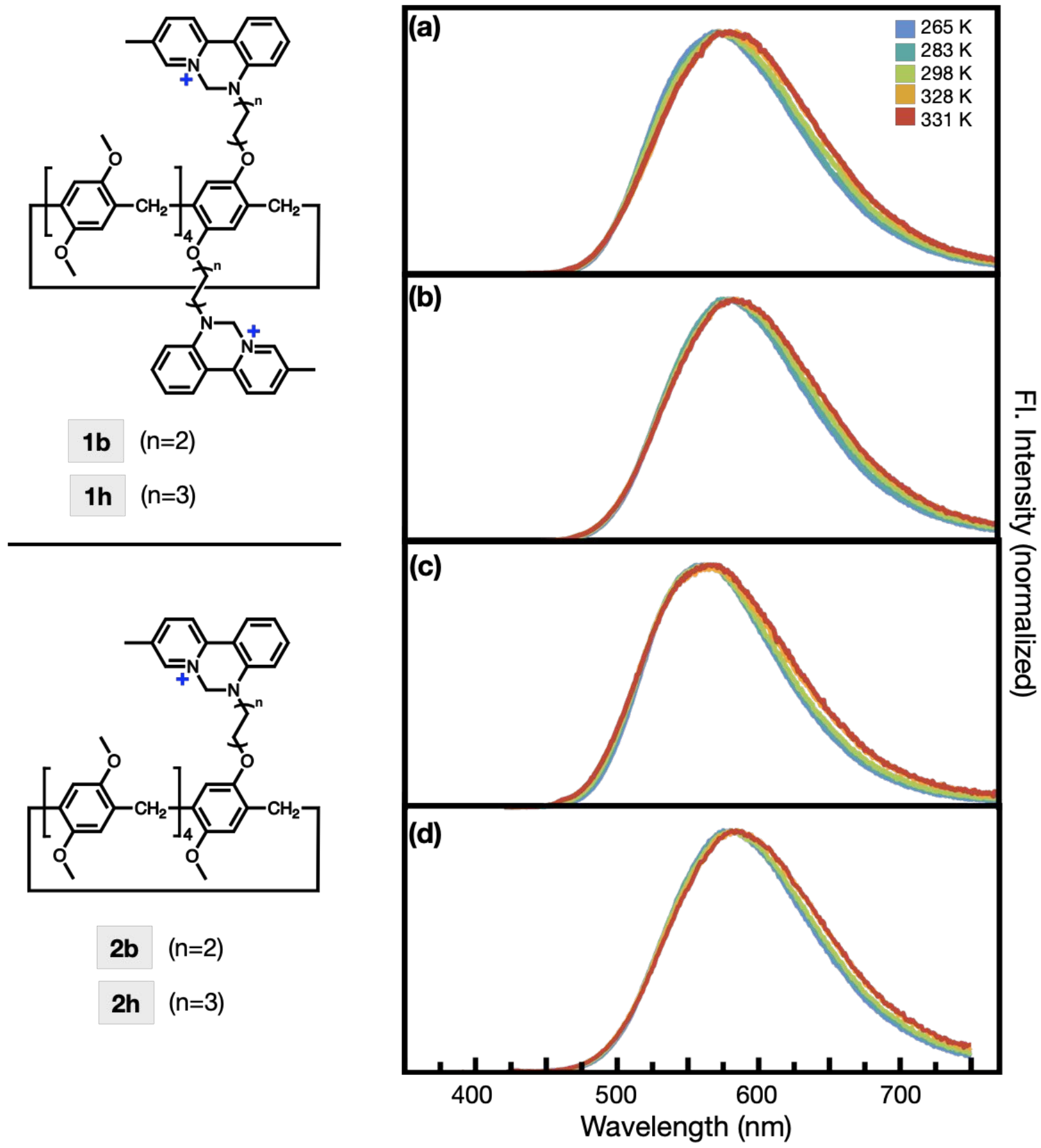

Fig. S31 Temperature-dependent emission spectra of (a) **1b**·Cl_2, (b) **1h**·Cl_2, (c) **2b**·Cl, and (d) **2h**·Cl in MeOH (sample concentration = 0.1 mM) from T = 265 to 331 K.

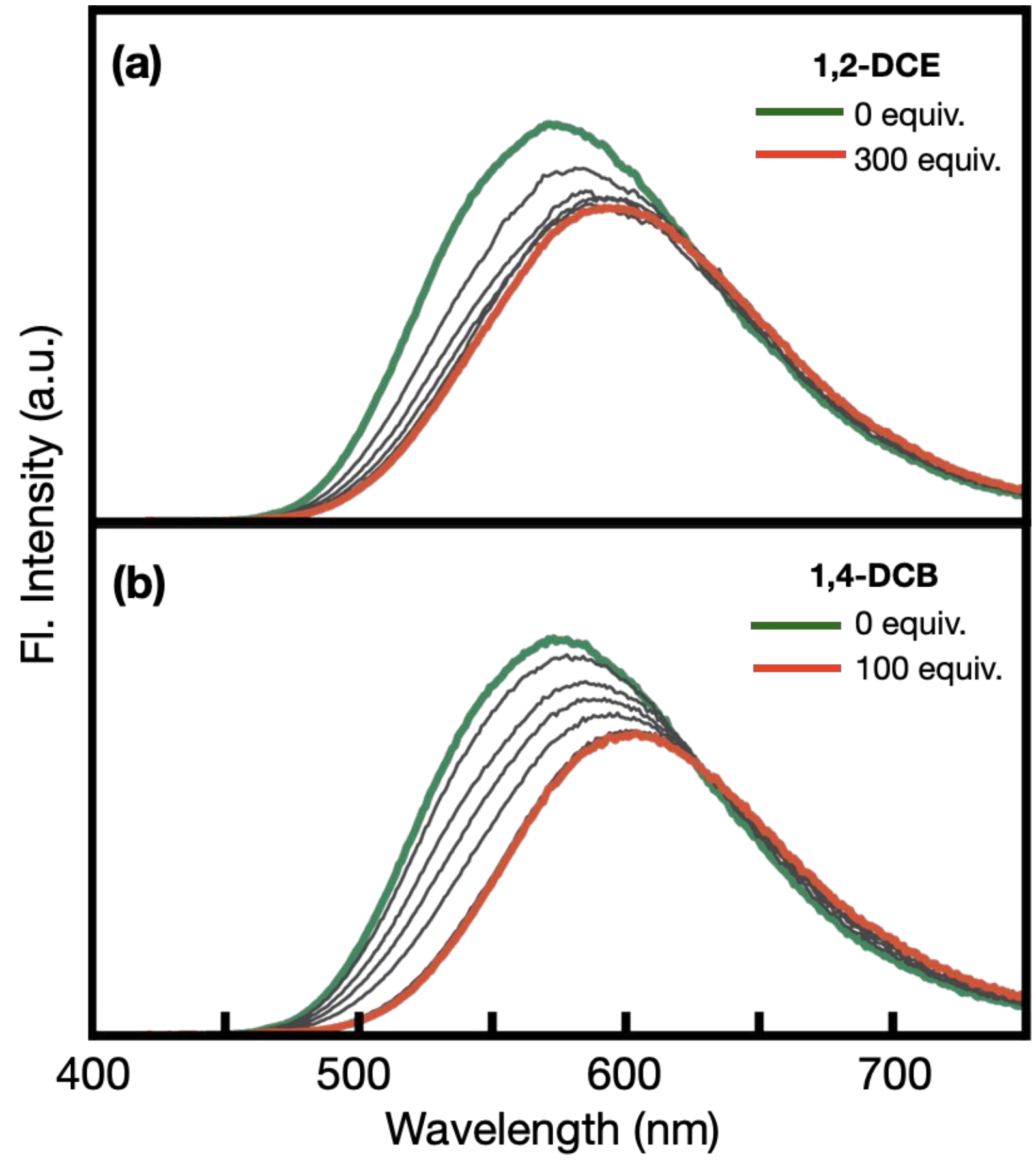

Fig. S32 Fluorescence spectral change (λ_{exc} = 410 nm) of **1b**·Cl_2 (0.1 mM) in MeOH upon addition of (a) 1,2-DCE, and (b) 1,4-DCB at T = 293 K.

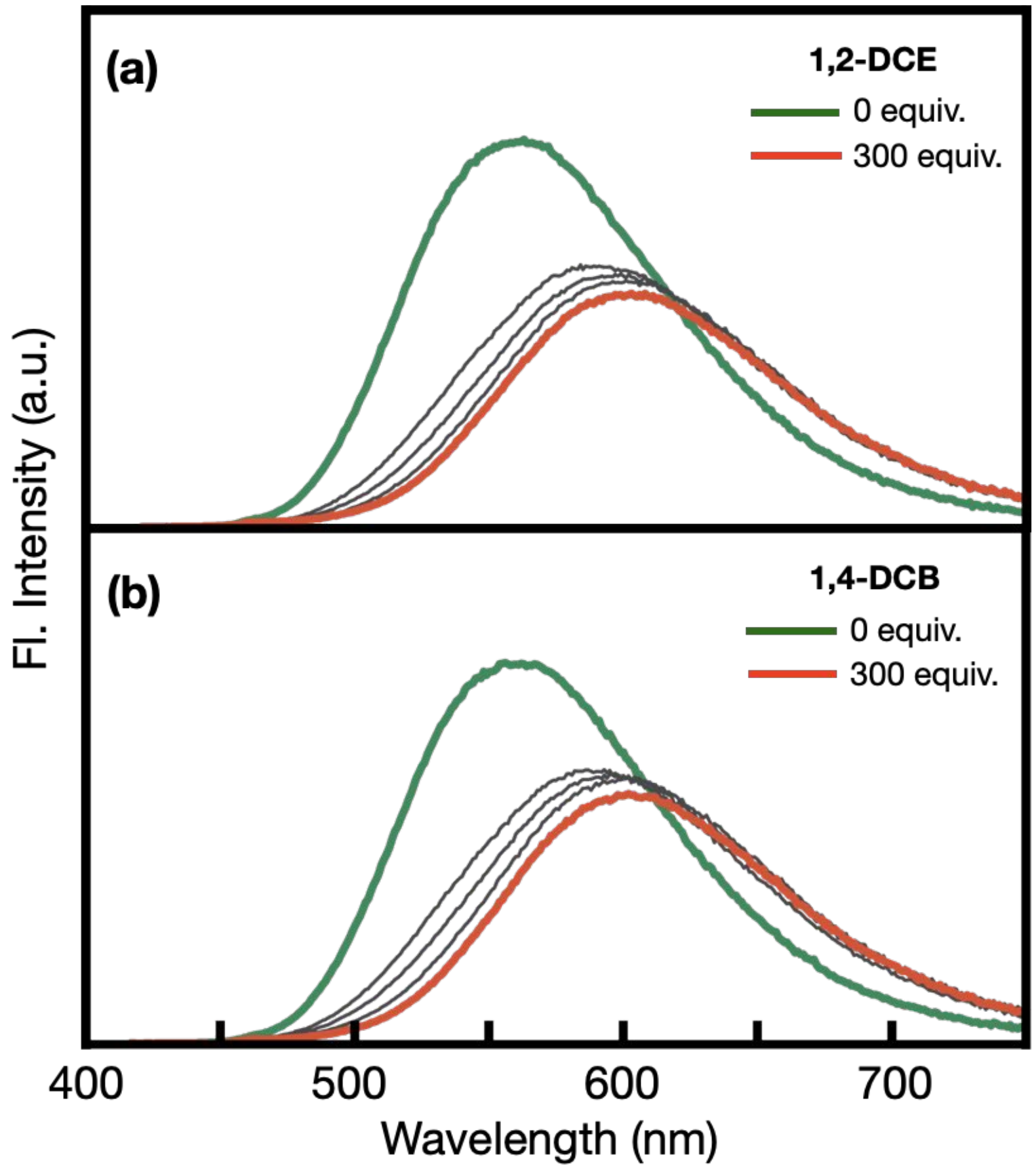

Fig. S33 Fluorescence spectral change (λ_{exc} = 410 nm) of **2b**·Cl (0.1 mM) in MeOH upon addition of (a) 1,2-DCE, and (b) 1,4-DCB at T = 293 K.

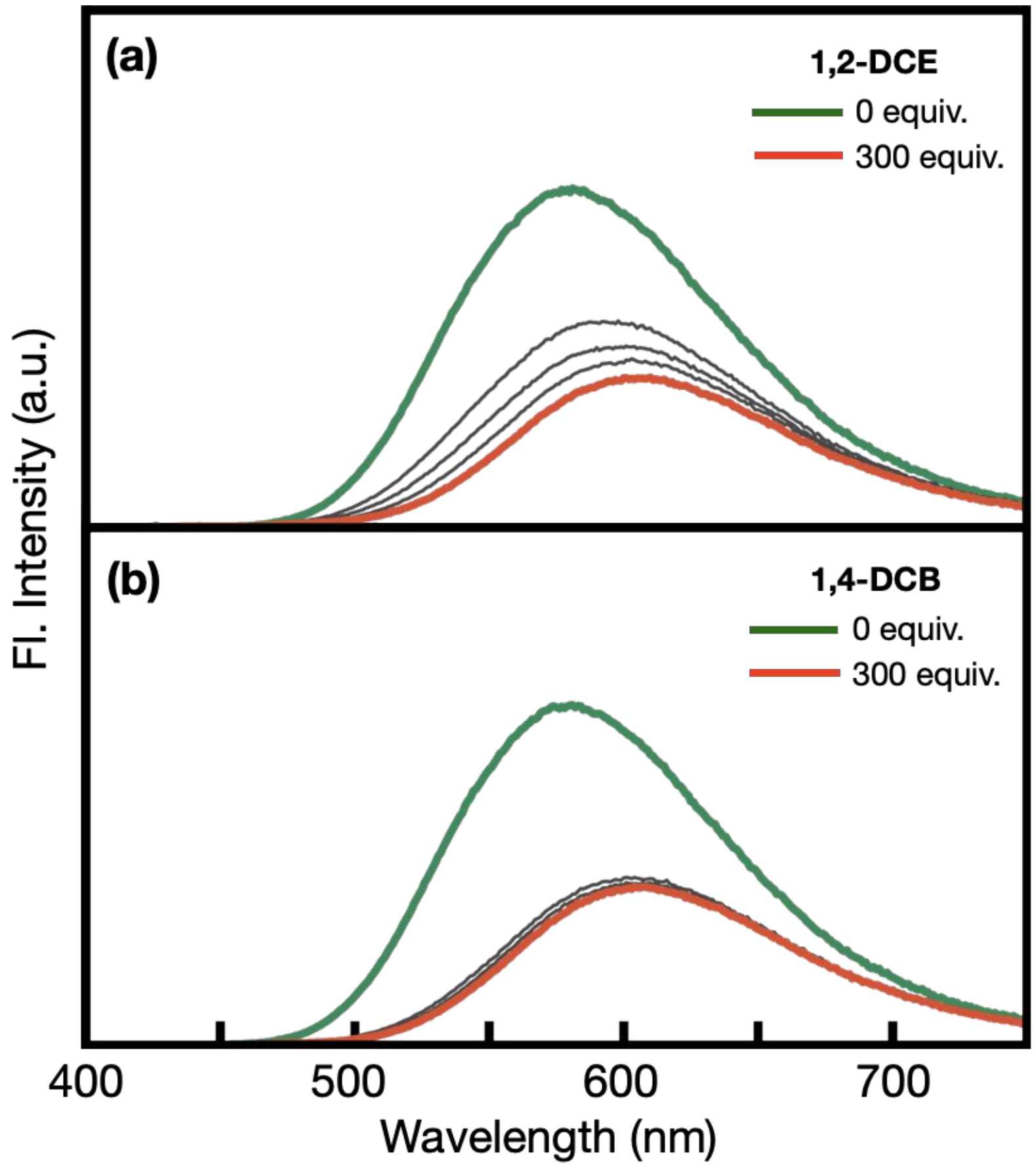

Fig. S34 Fluorescence spectral change (λ_{exc} = 415 nm) of **1h**·Cl_2 (0.1 mM) in MeOH upon addition of (a) 1,2-DCE, and (b) 1,4-DCB at T = 293 K.

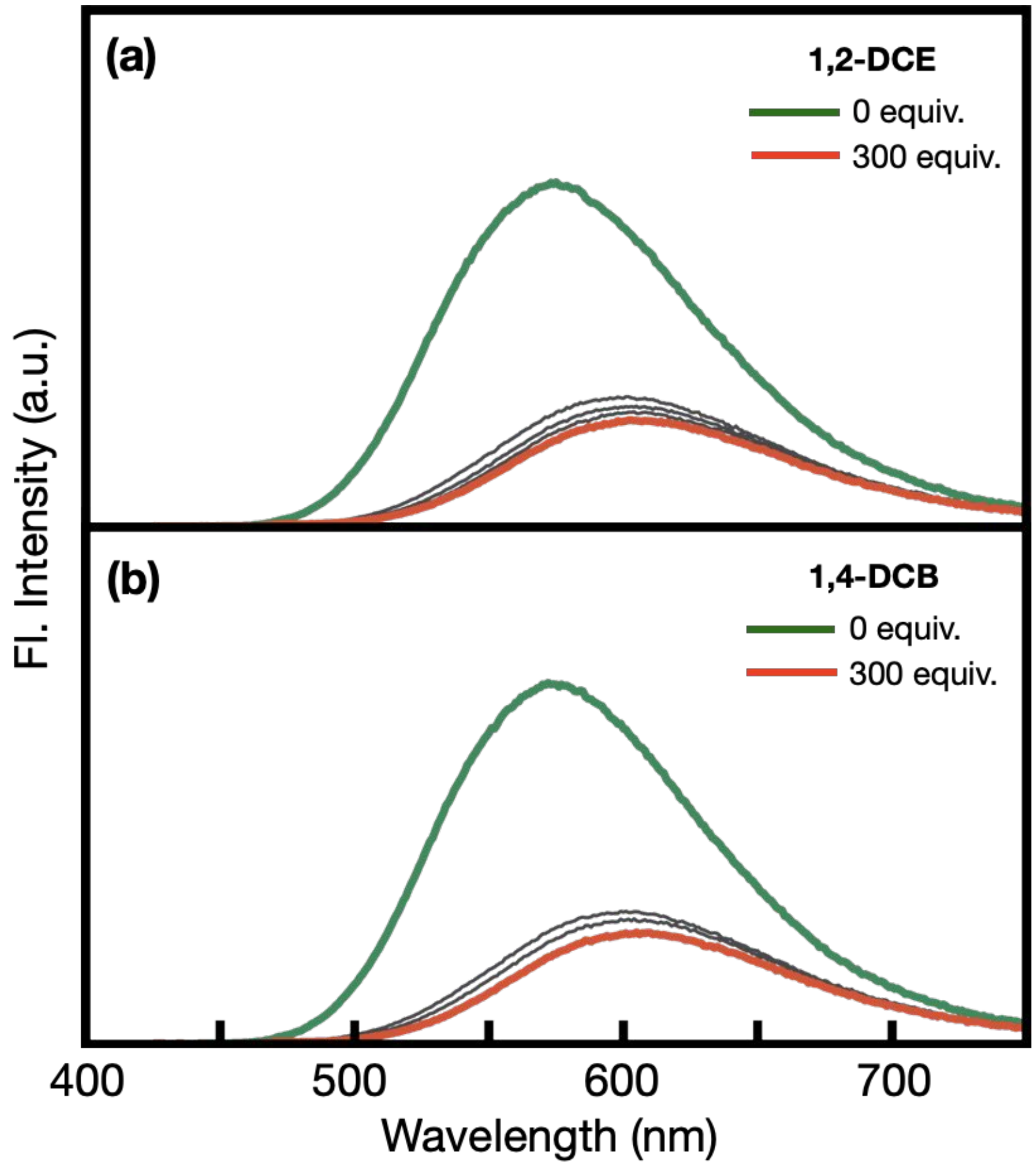

Fig. S35 Fluorescence spectral change (λ_{exc} = 415 nm) of **2h**·Cl (0.1 mM) in MeOH upon addition of (a) 1,2-DCE, and (b) 1,4-DCB at T = 293 K.

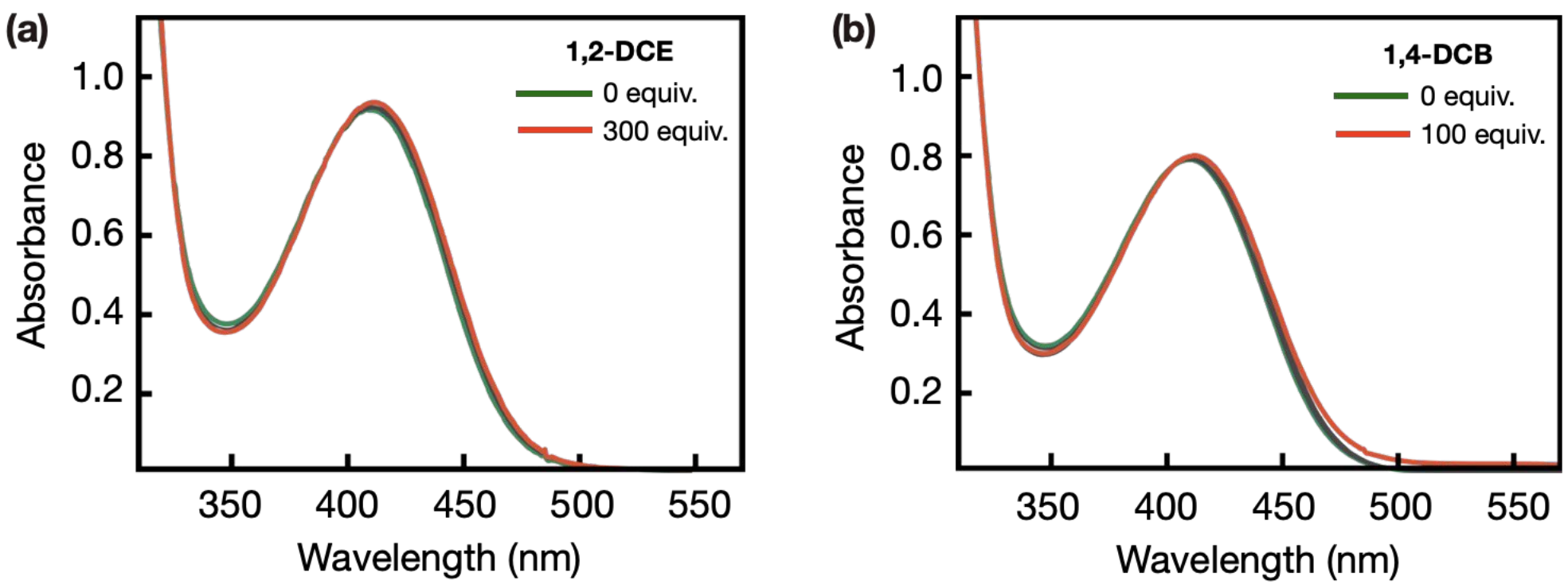

Fig. S36 UV–vis absorption spectral changes of **1b**·Cl_2 (0.1 mM) upon addition of (a) 1,2-DCE, and (b) 1,4-DCB in MeOH at $T = 293$ K.

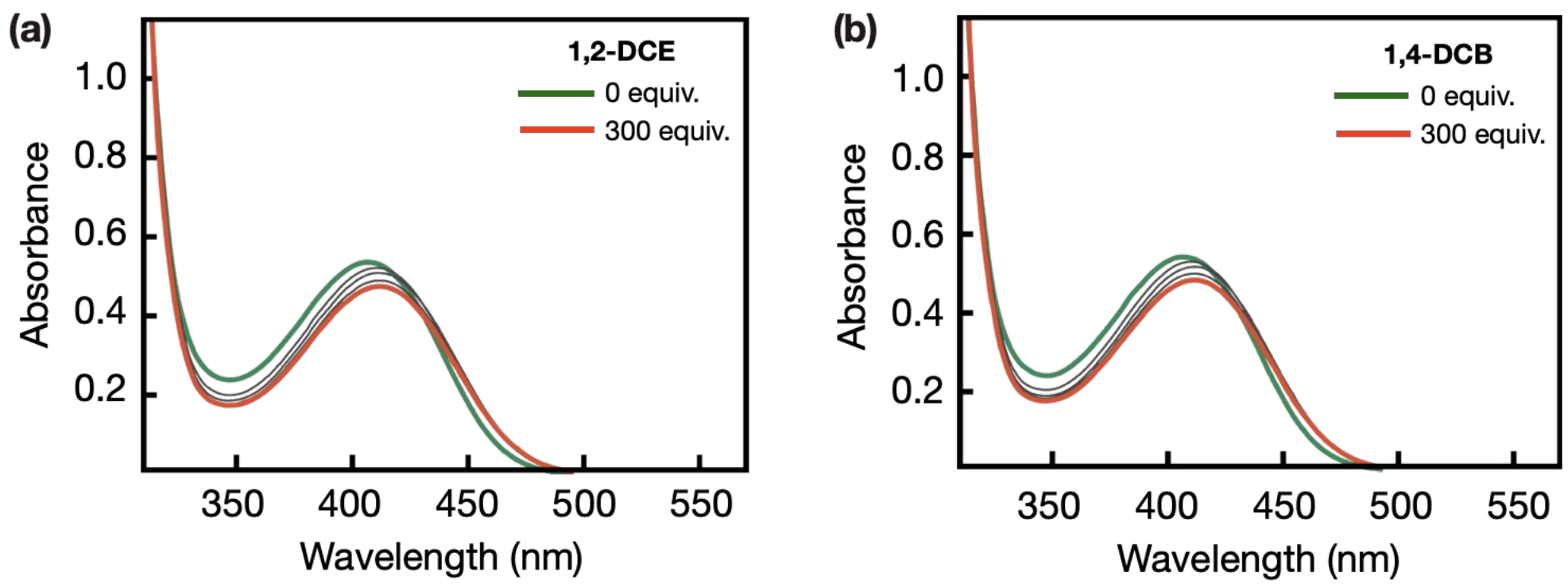

Fig. S37 UV–vis absorption spectral changes of **2b**·Cl (0.1 mM) upon addition of (a) 1,2-DCE, and (b) 1,4-DCB in MeOH at $T = 293$ K.

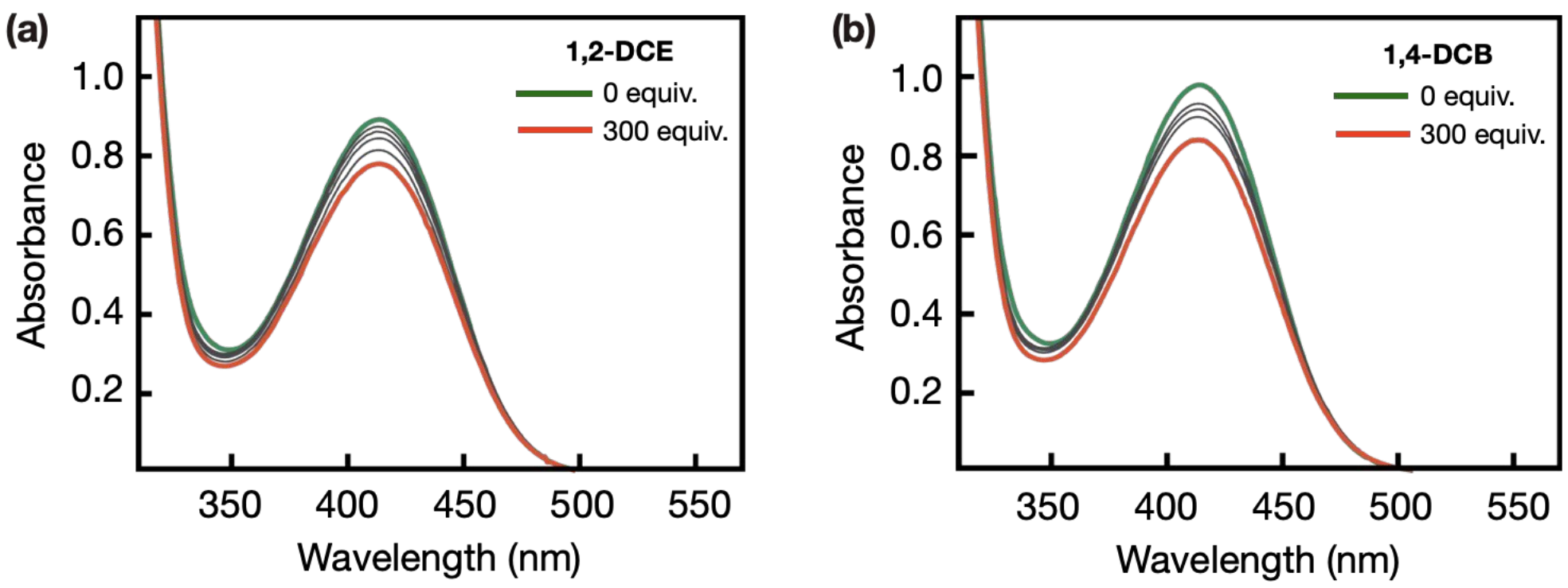

Fig. S38 UV–vis absorption spectral changes of **1h**·Cl_2 (0.1 mM) upon addition of (a) 1,2-DCE, and (b) 1,4-DCB in MeOH at T = 293 K.

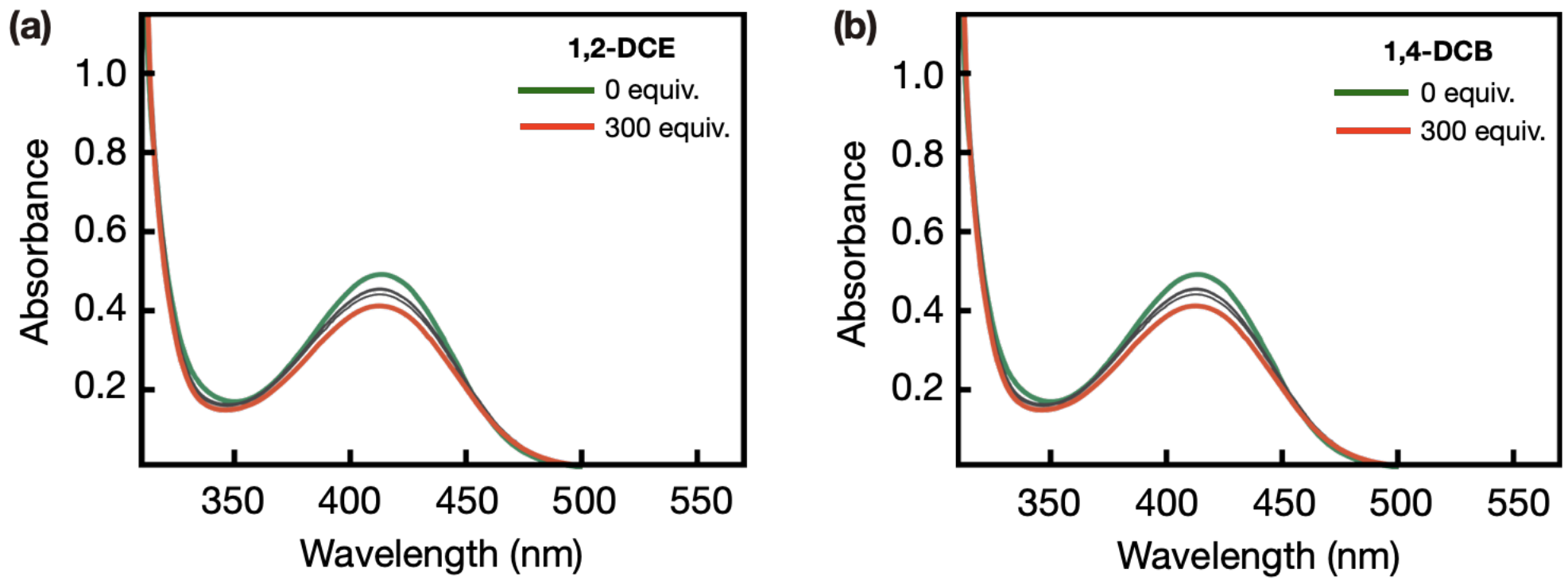

Fig. S39 UV–vis absorption spectral changes of **2h**·Cl (0.1 mM) upon addition of (a) 1,2-DCE, and (b) 1,4-DCB in MeOH at $T = 293$ K.

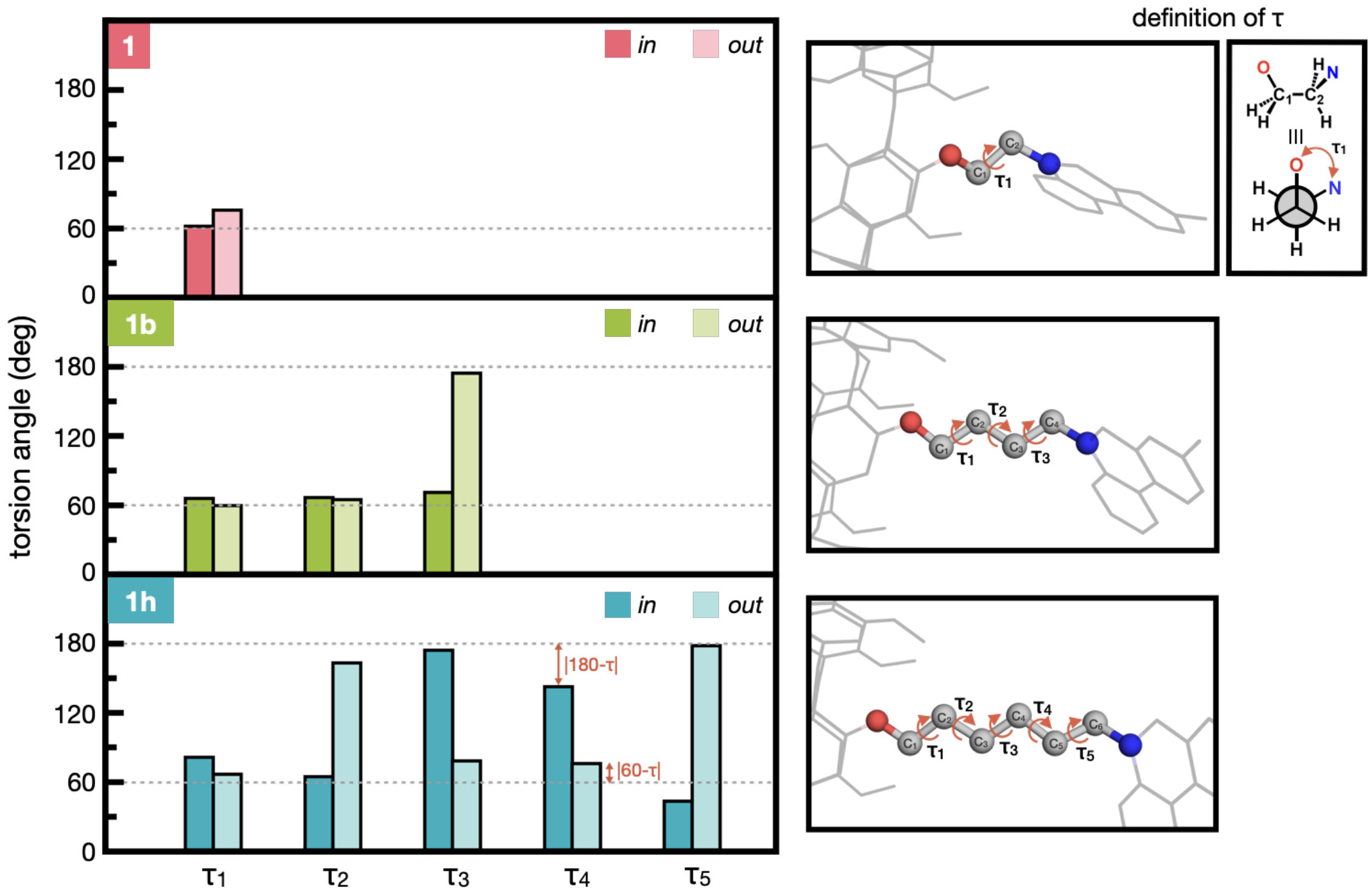

Fig. S40 Alkyl tether torsion angles (τ_1–τ_5) of the *in* and *out* arms in the DFT-optimized structures of **1** (top), **1b** (middle), and **1h** (bottom). Torsion-angle definitions are shown in the right panel. Dashed lines mark the ideal staggered values (60° and 180°); $|180-\tau|$ and $|60-\tau|$ represent the deviation of a given torsion angle from the nearer staggered minimum.

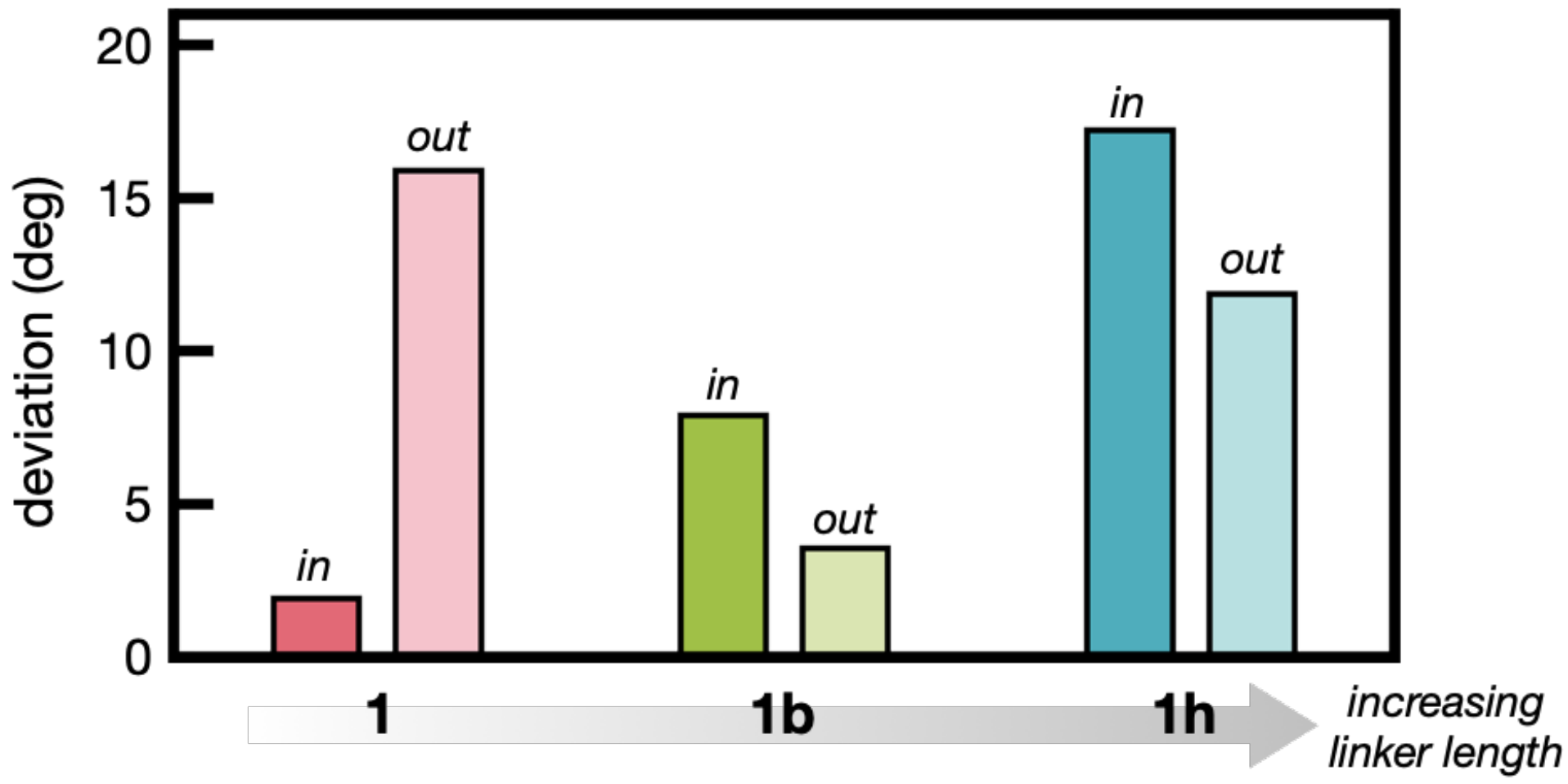

Fig. S41 Average deviation of the alkyl backbone torsion angles from the nearer ideal staggered value (60° or 180°) for the *in* and *out* arms of **1**, **1b**, and **1h**.

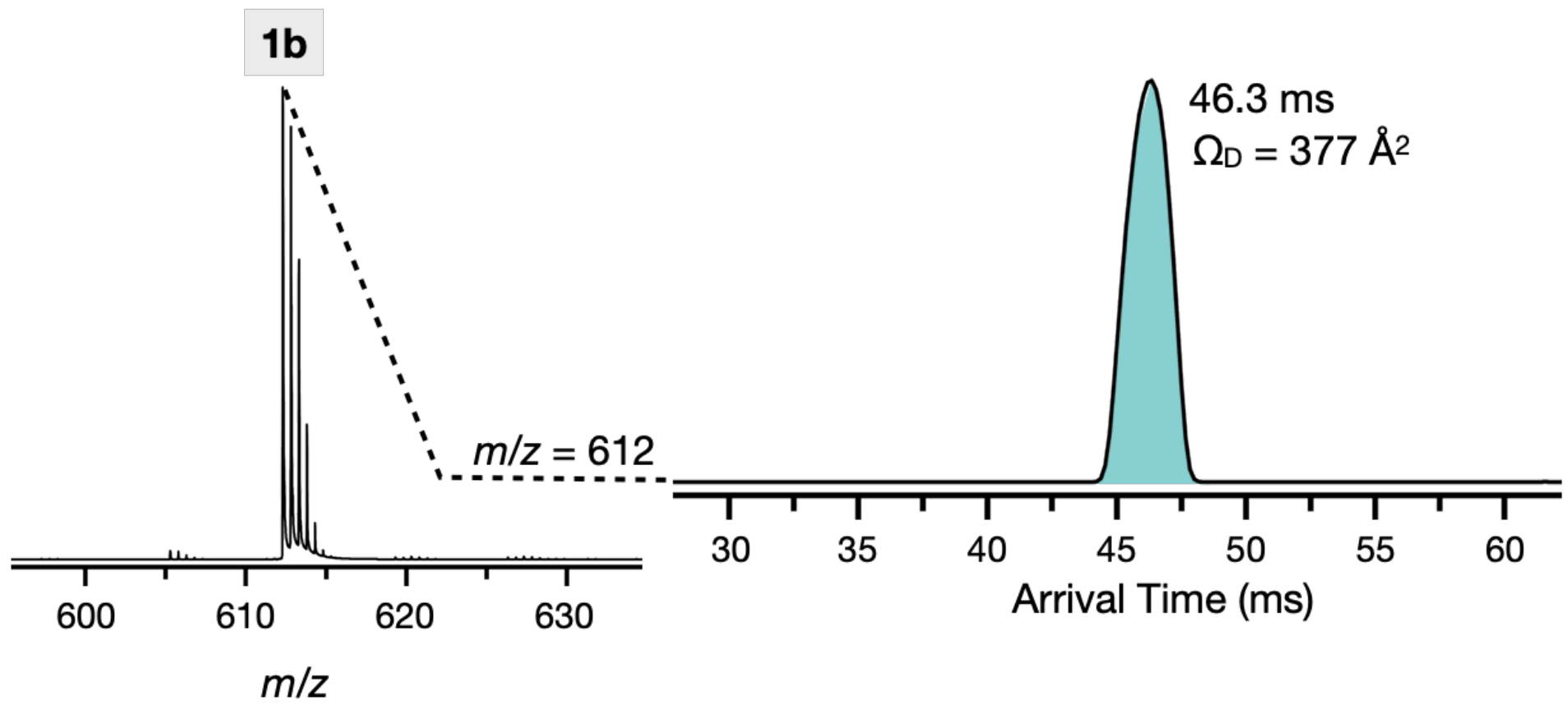

Fig. S42 ESI-IMS-MS spectra of **1b**, showing mass-to-charge analysis (left) and ion mobility separation by arrival time distributions (right). Experimental collision cross-section values ($\Omega_{D,exp}$) are provided for mobility peaks.

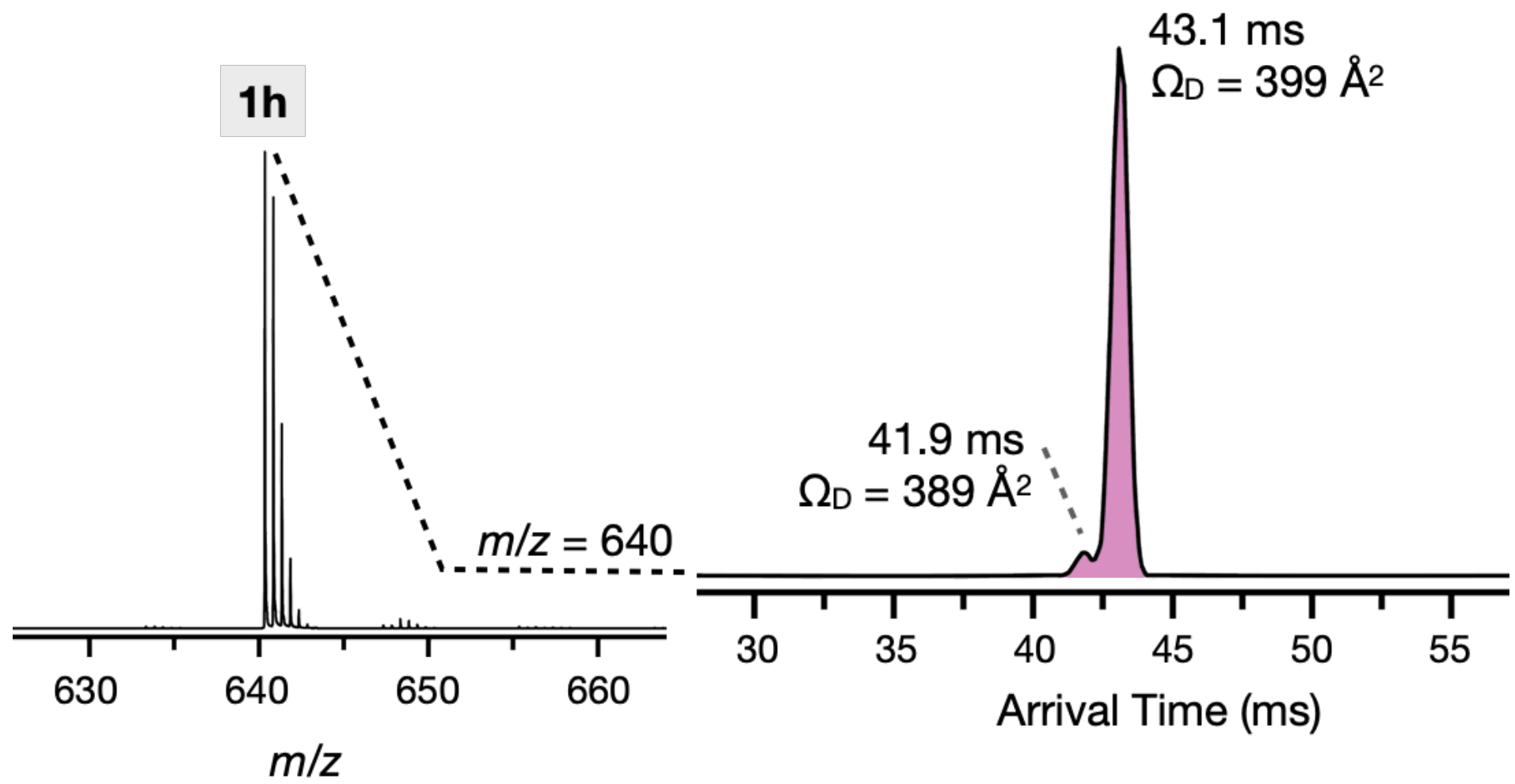

Fig. S43 ESI-IMS-MS spectra of **1h**, showing mass-to-charge analysis (left) and ion mobility separation by arrival time distributions (right). Experimental collision cross-section values ($\Omega_{D,exp}$) are provided for mobility peak.

(a)

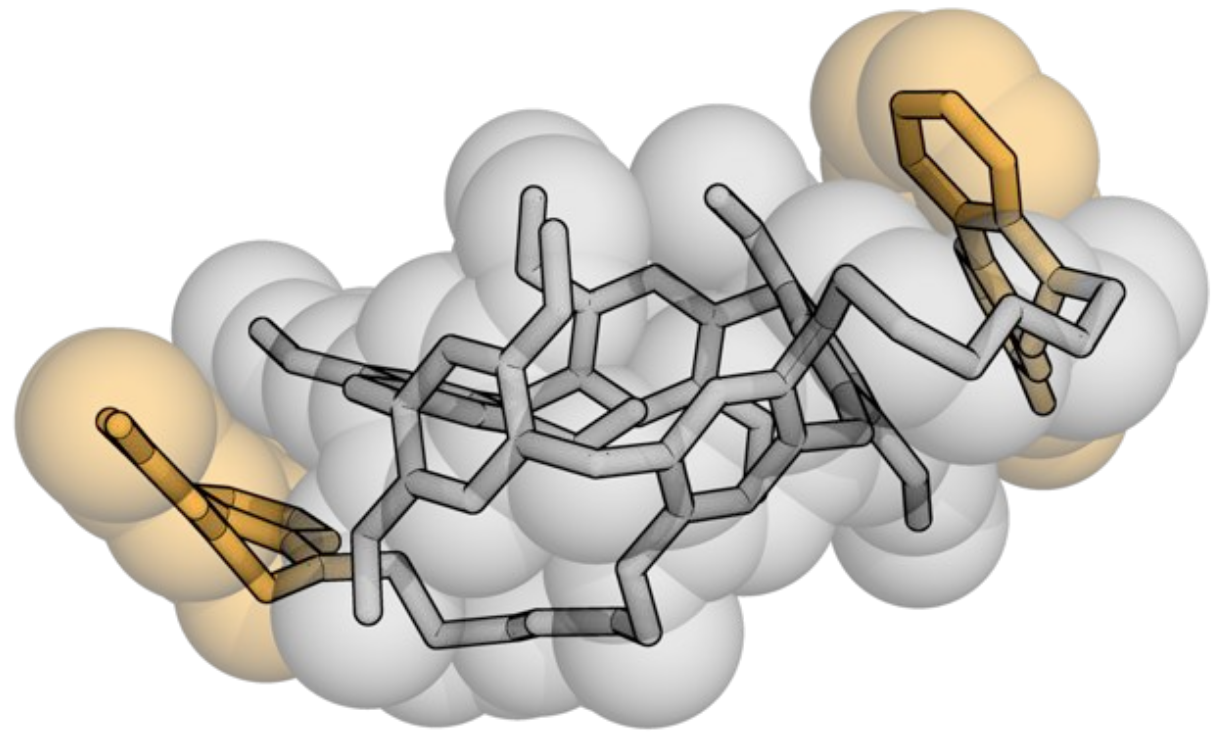

(b)

BisC6	
CCS Exp. (Å^2)	399
CCS Calc. (Å^2)	394

Fig. S44 Energy-minimized DFT computational model of (a) **1h**. (b) Comparison of the experimental and calculated collision cross section (CCS) values (Å^2) for the corresponding structures shown in (a).

(a)

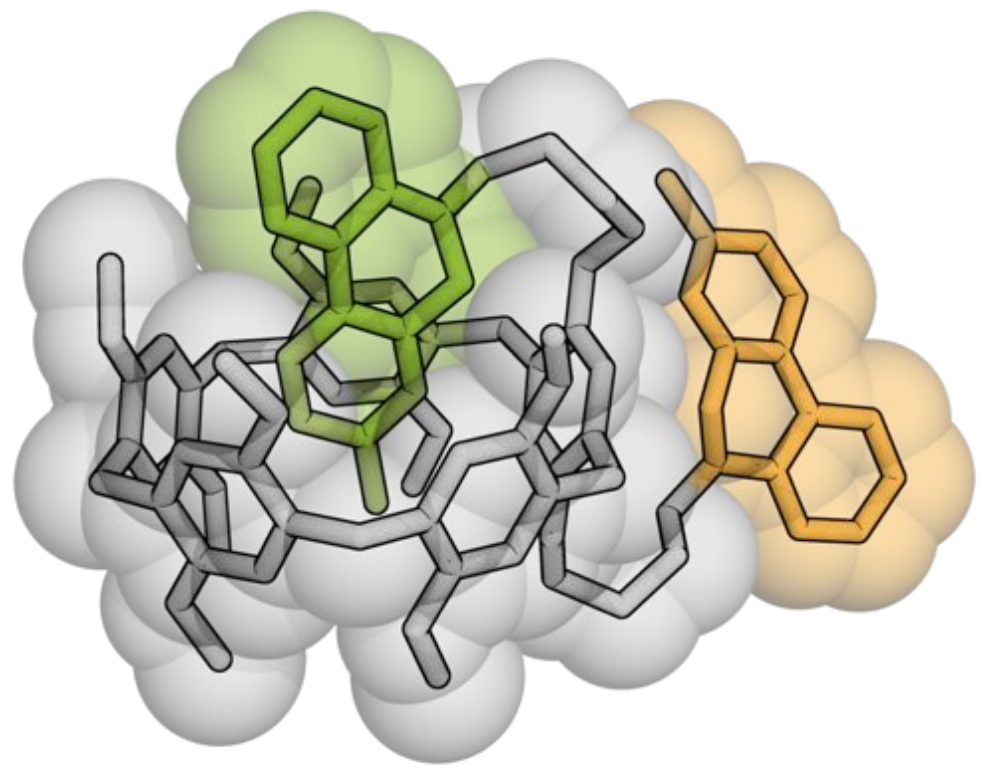

(b)

BisC4	
CCS Exp. (Å^2)	377
CCS Calc. (Å^2)	364

Fig. S45 Energy-minimized DFT computational model of (a) **1b**. (b) Comparison of the experimental and calculated collision cross section (CCS) values (Å^2) for the corresponding structures shown in (a).

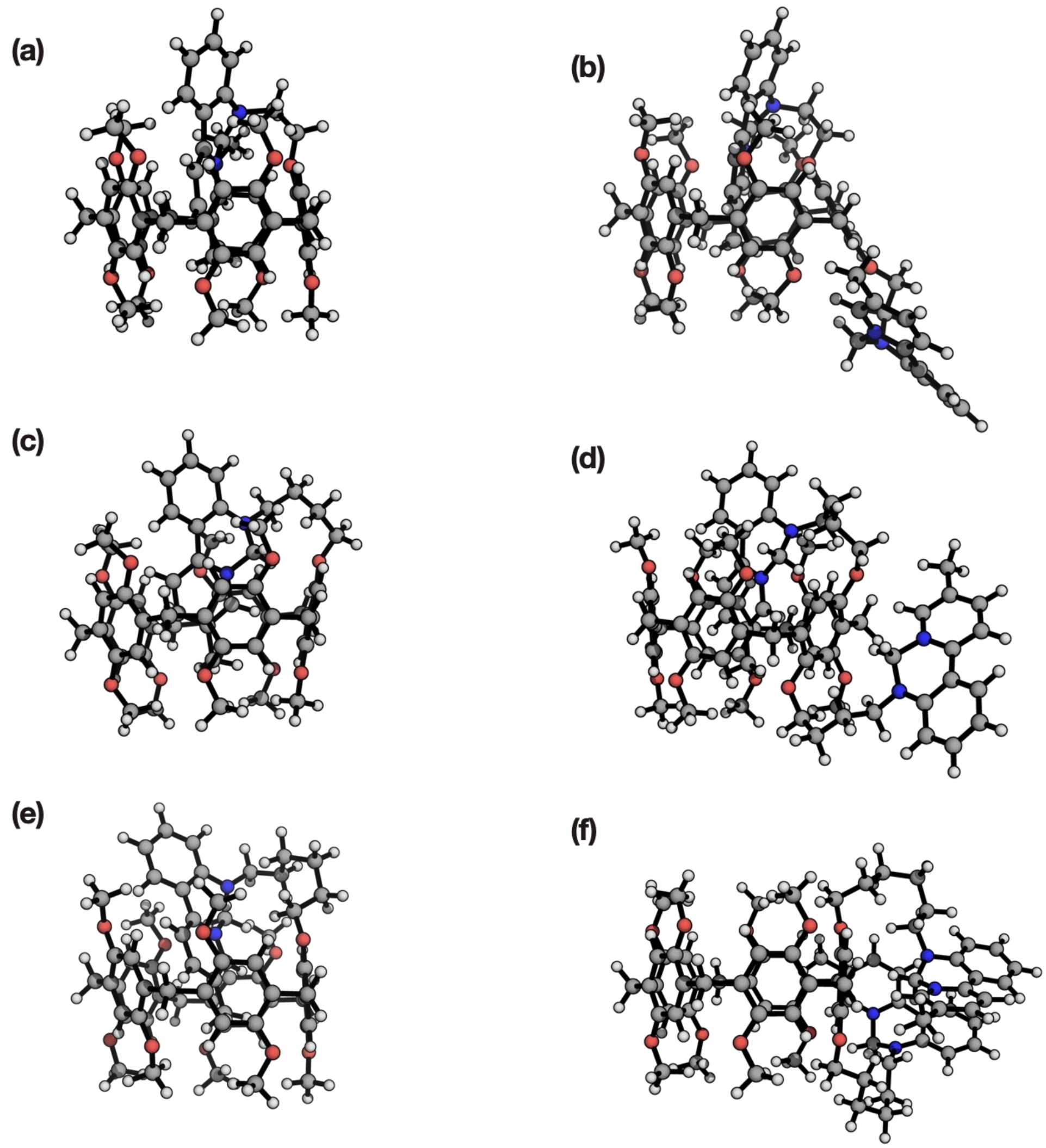

Fig. S46 Geometry optimized DFT models (B3LYP-D3/6-31G** level; implicit solvation in MeOH) of (a) **2**, (b) **1**, (c) **2b**, (d) **1b**, (e) **2h**, and (f) **1h**.

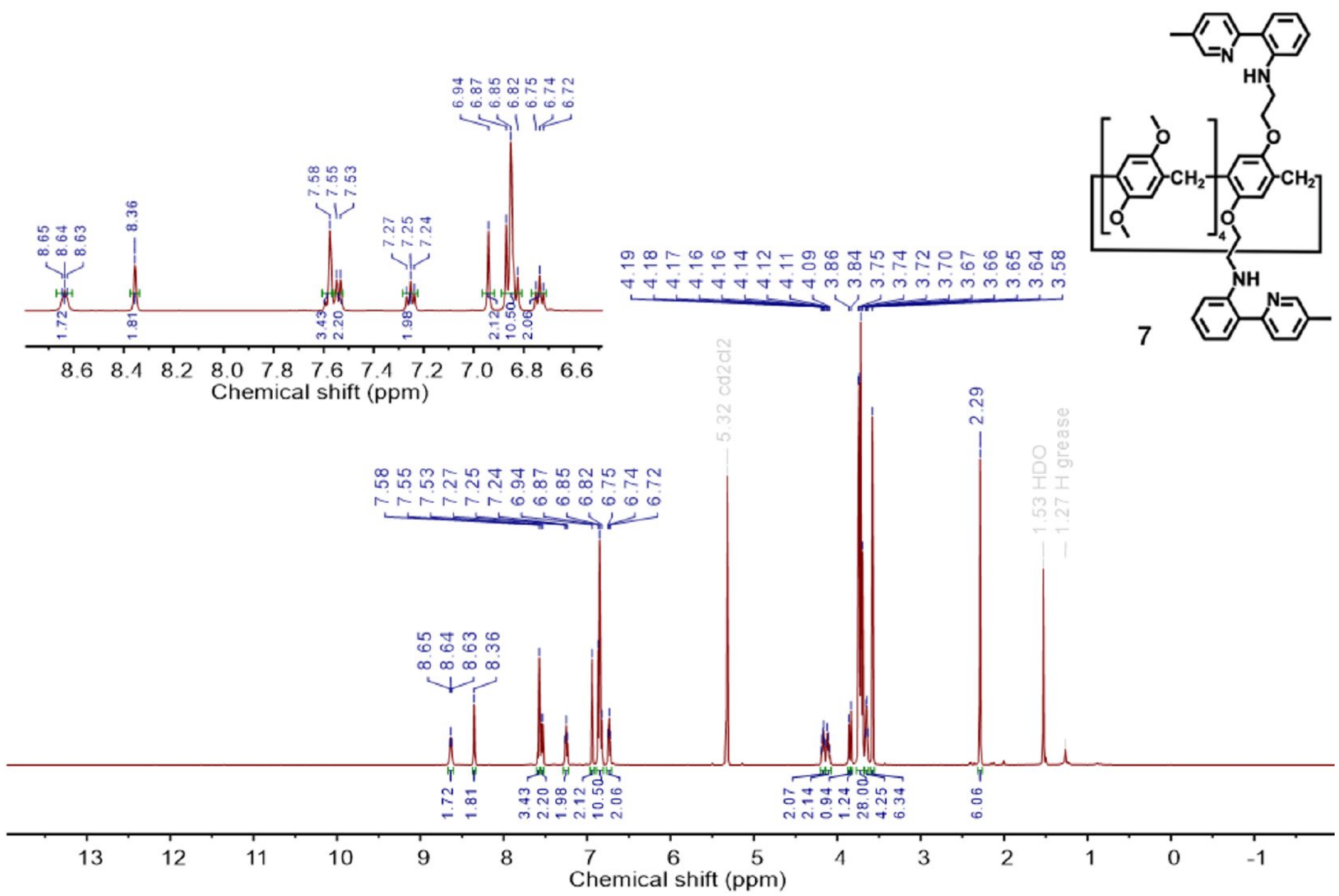

Fig. S47 ^{1}H NMR (500 MHz) spectrum of **7** in CD_2Cl_2 (T = 298 K).

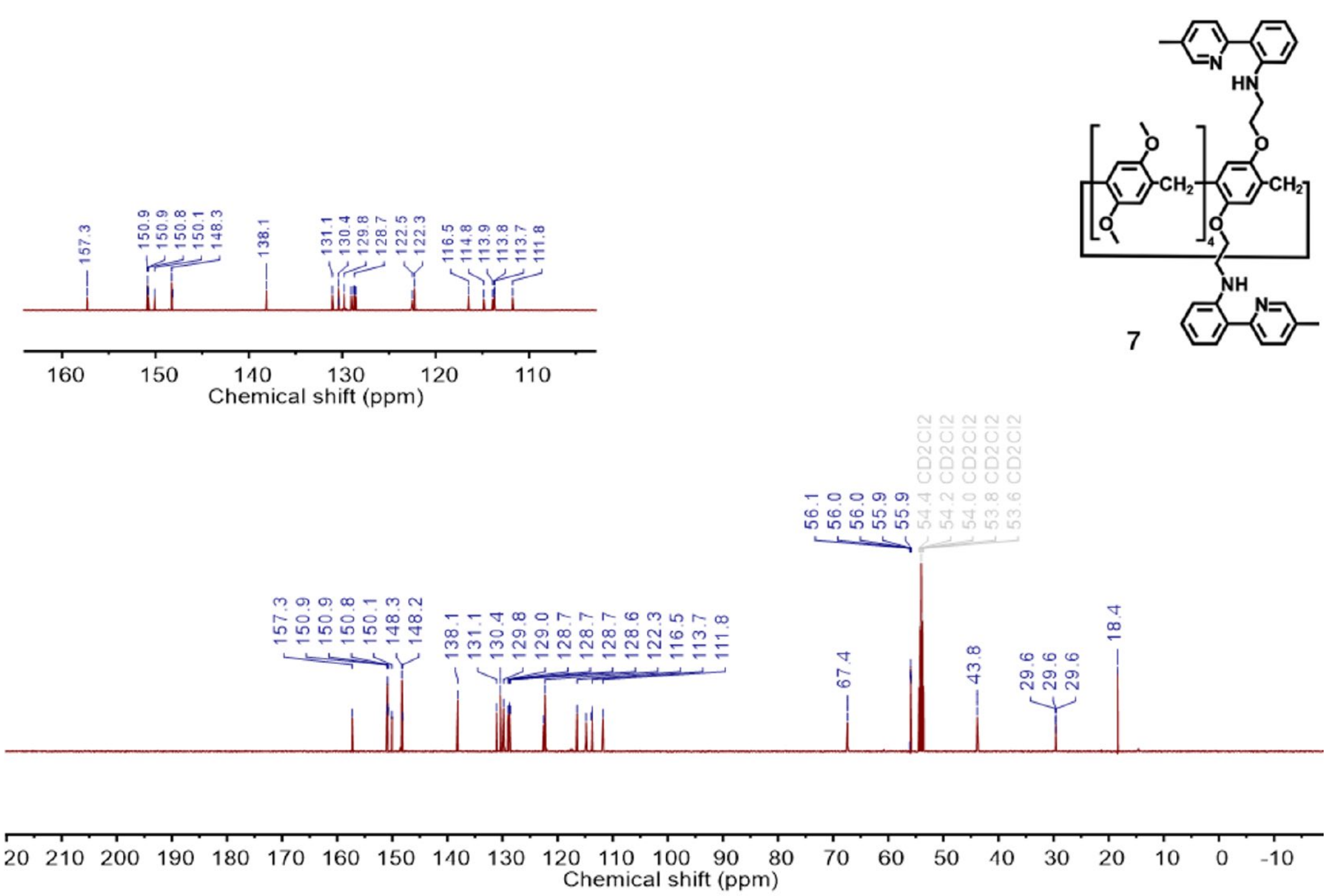

Fig. S48 ^{13}C NMR (126 MHz) spectrum of **7** in CD_2Cl_2 (T = 298 K).

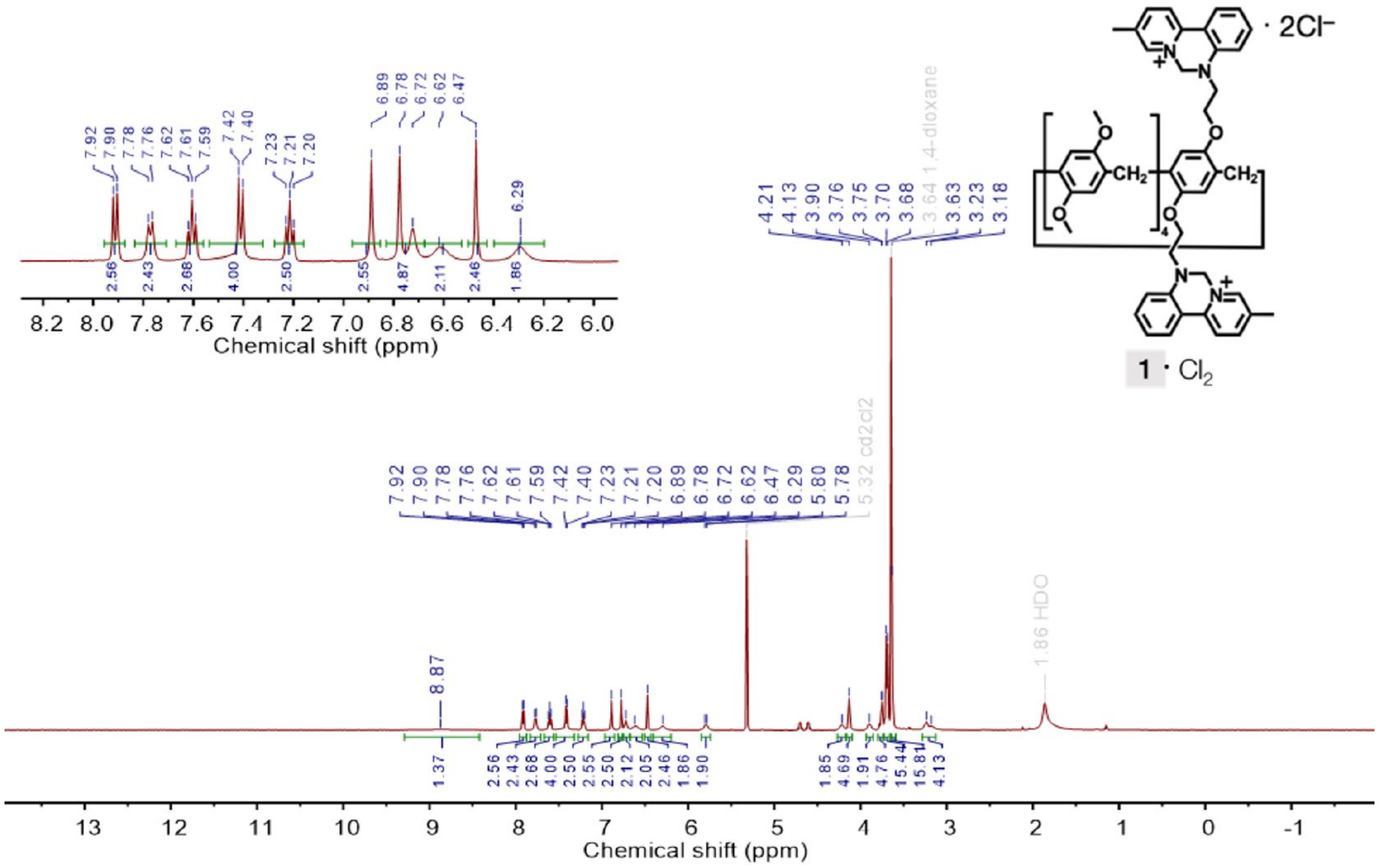

Fig. S49 ^{1}H NMR (500 MHz) spectrum of **1**·Cl_2 in CD_2Cl_2 (T = 298 K).

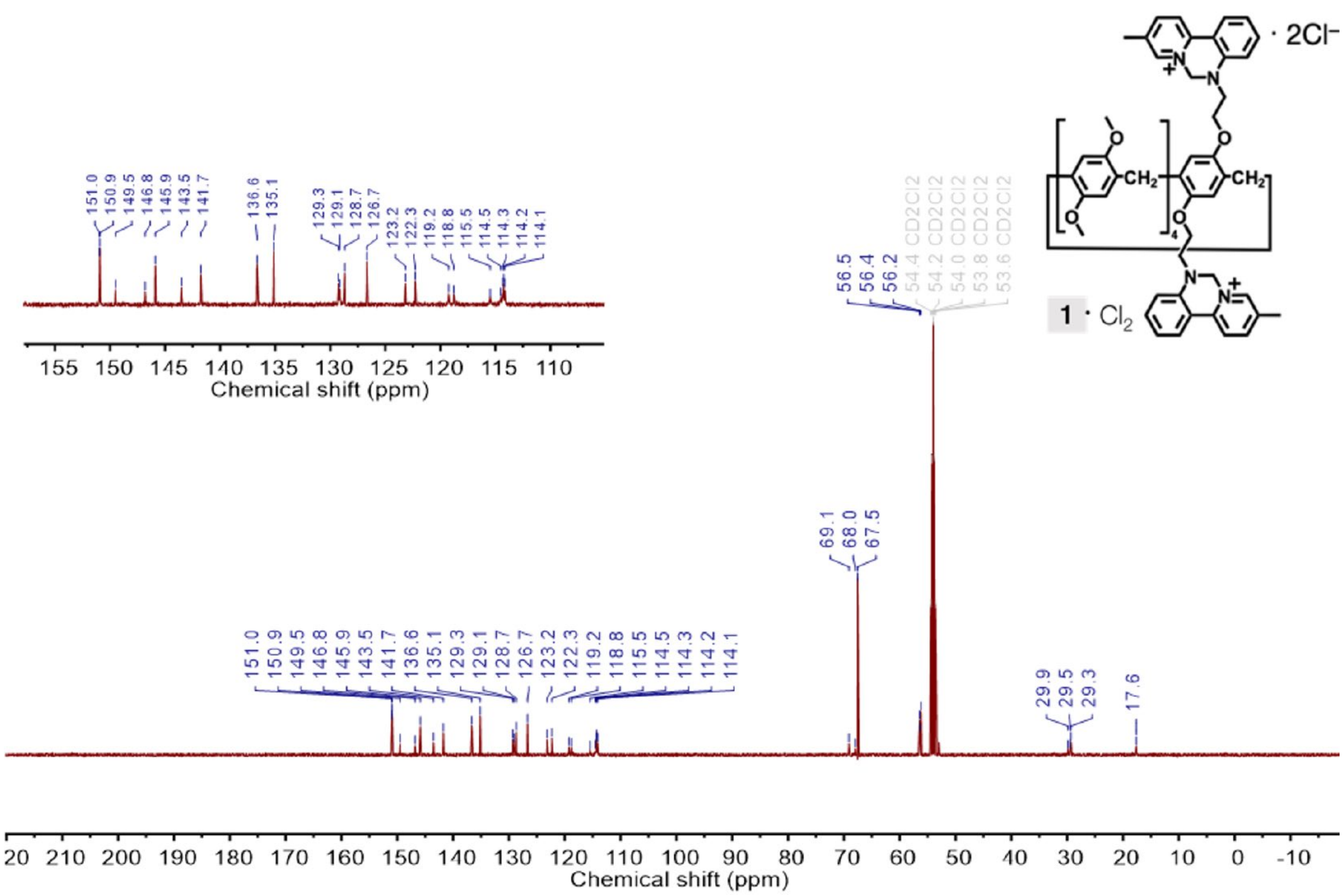

Fig. S50 ^{13}C NMR (126 MHz) spectrum of **1**·Cl_2 in CD_2Cl_2 (T = 298 K).

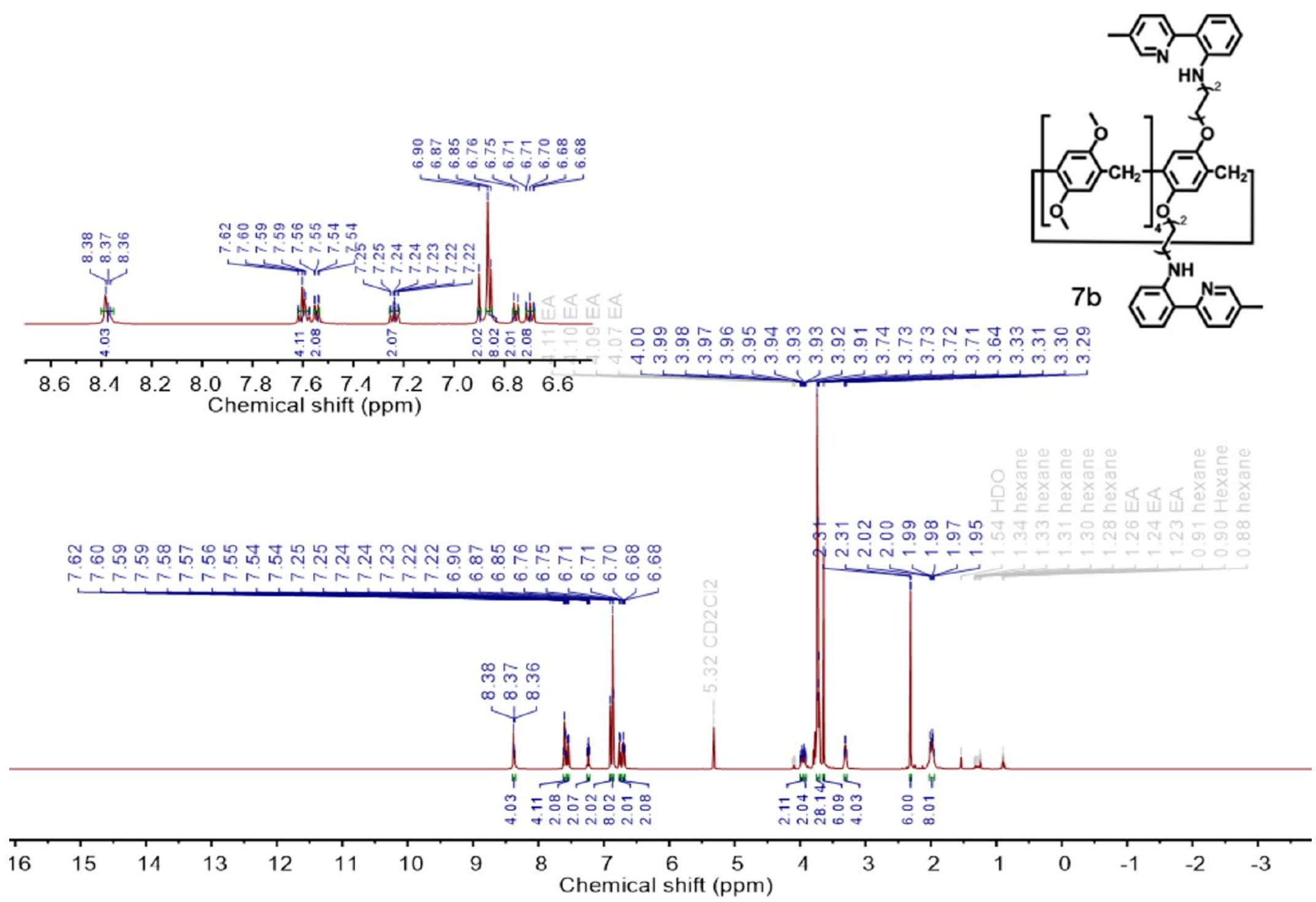

Fig. S51 ^{1}H NMR (500 MHz) spectrum of **7b** in CD_2Cl_2 (T = 298 K).

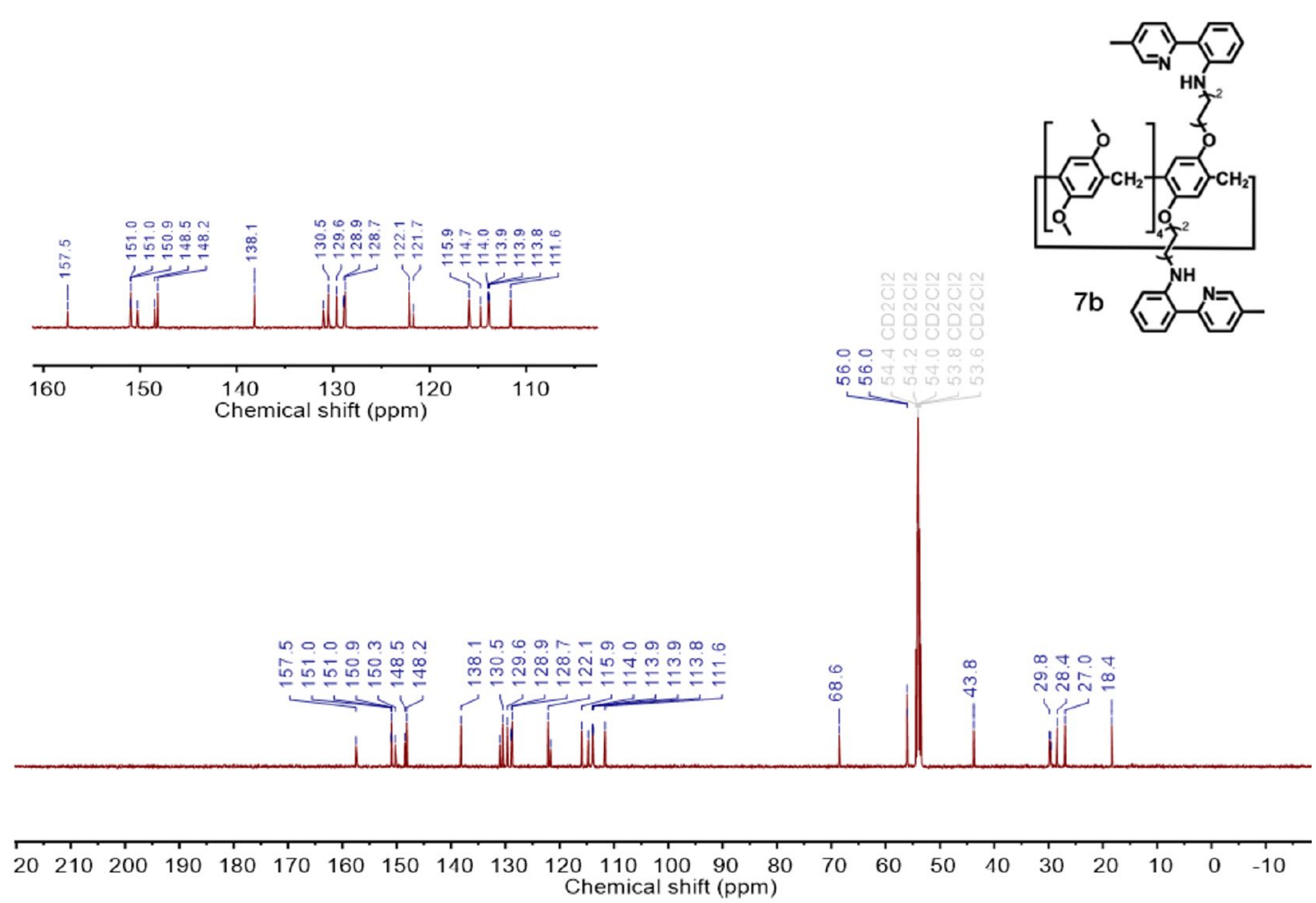

Fig. S52 ^{13}C NMR (126 MHz) spectrum of **7b** in CD_2Cl_2 (T = 298 K).

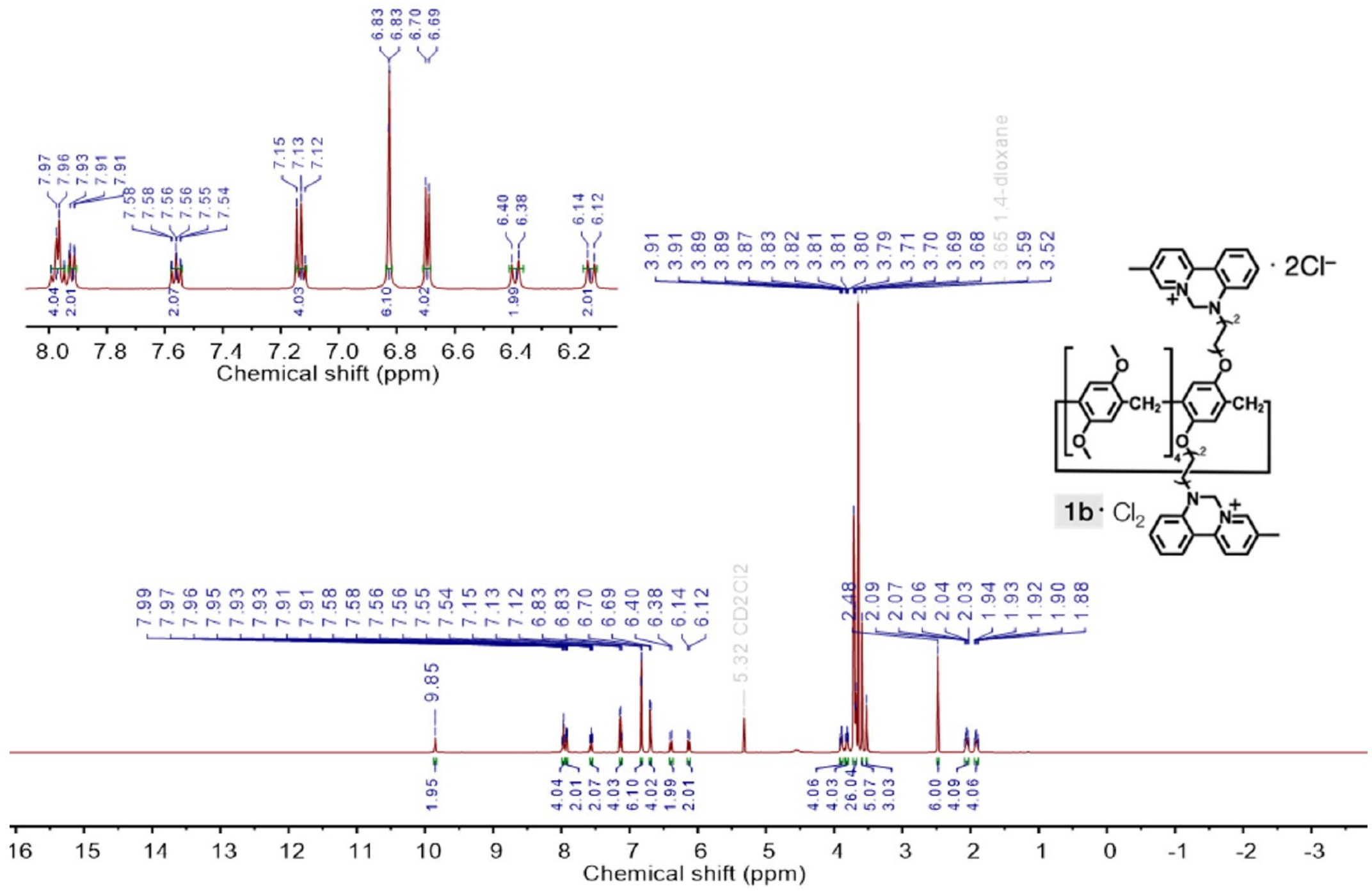

Fig. S53 ^{1}H NMR (500 MHz) spectrum of **1b**·Cl_2 in CD_2Cl_2 (T = 298 K).

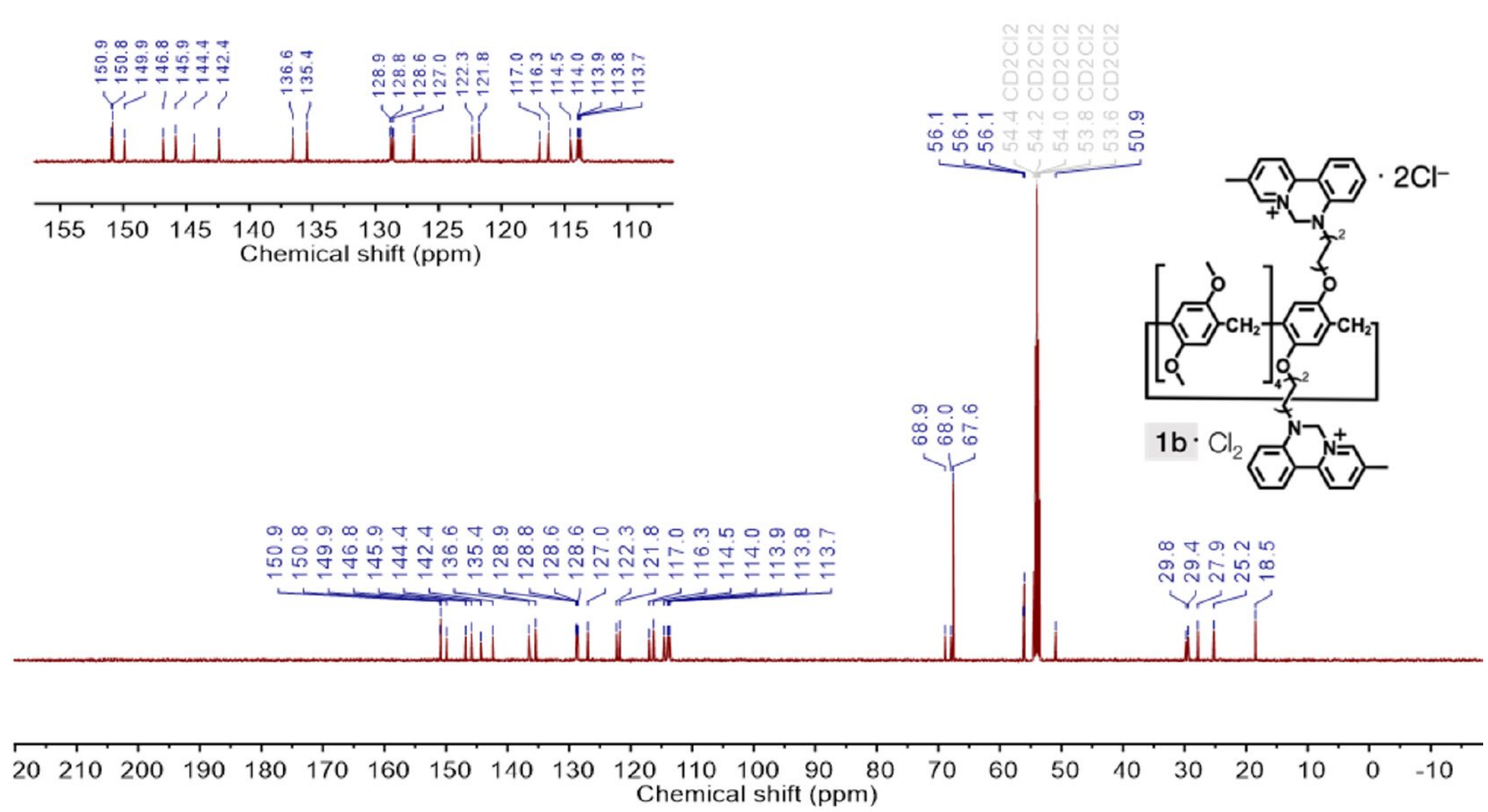

Fig. S54 ^{13}C NMR (126 MHz) spectrum of **1b**·Cl_2 in CD_2Cl_2 (T = 298 K).

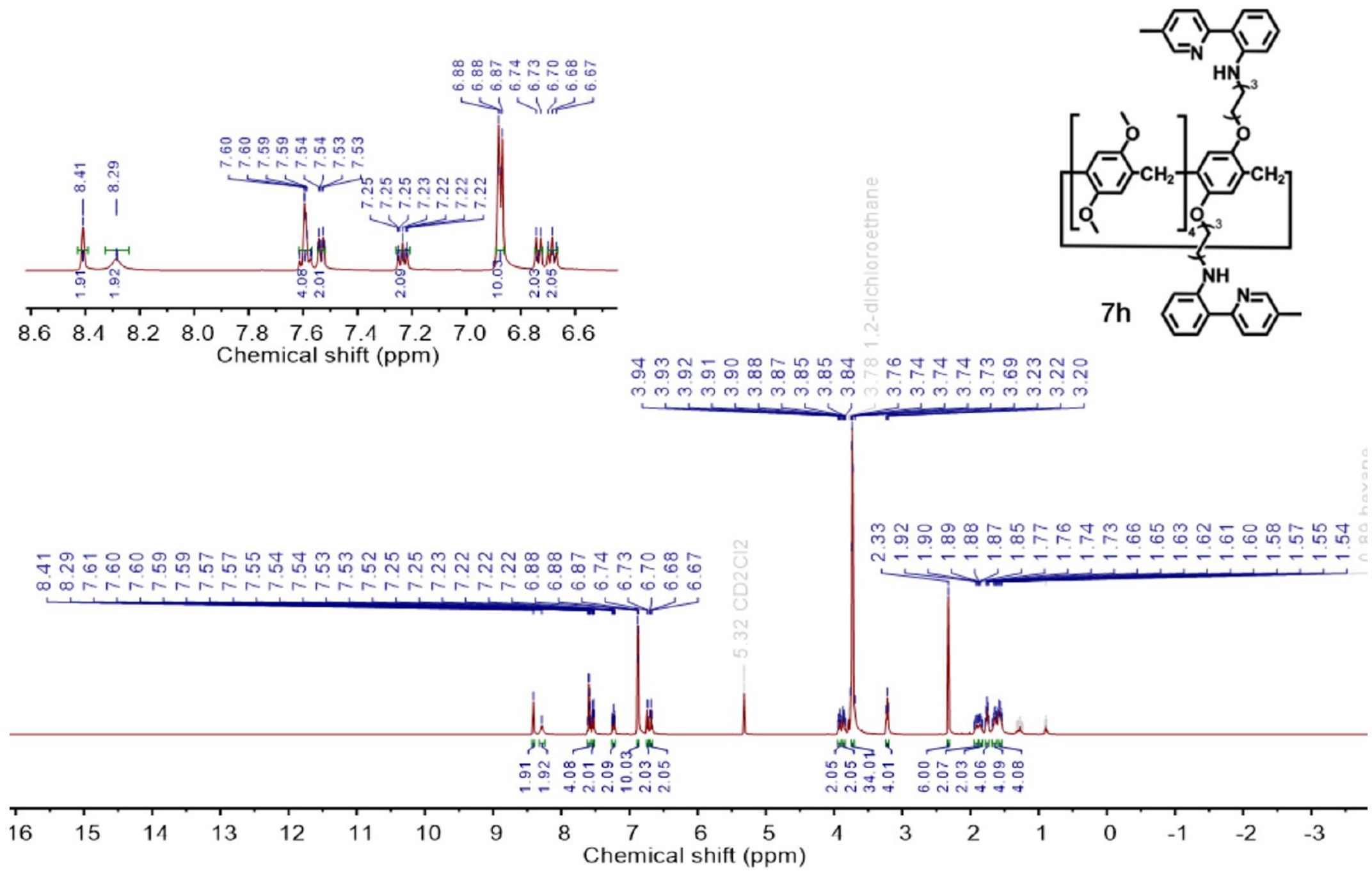

Fig. S55 ^{1}H NMR (500 MHz) spectrum of **7h** in CD_2Cl_2 (T = 298 K).

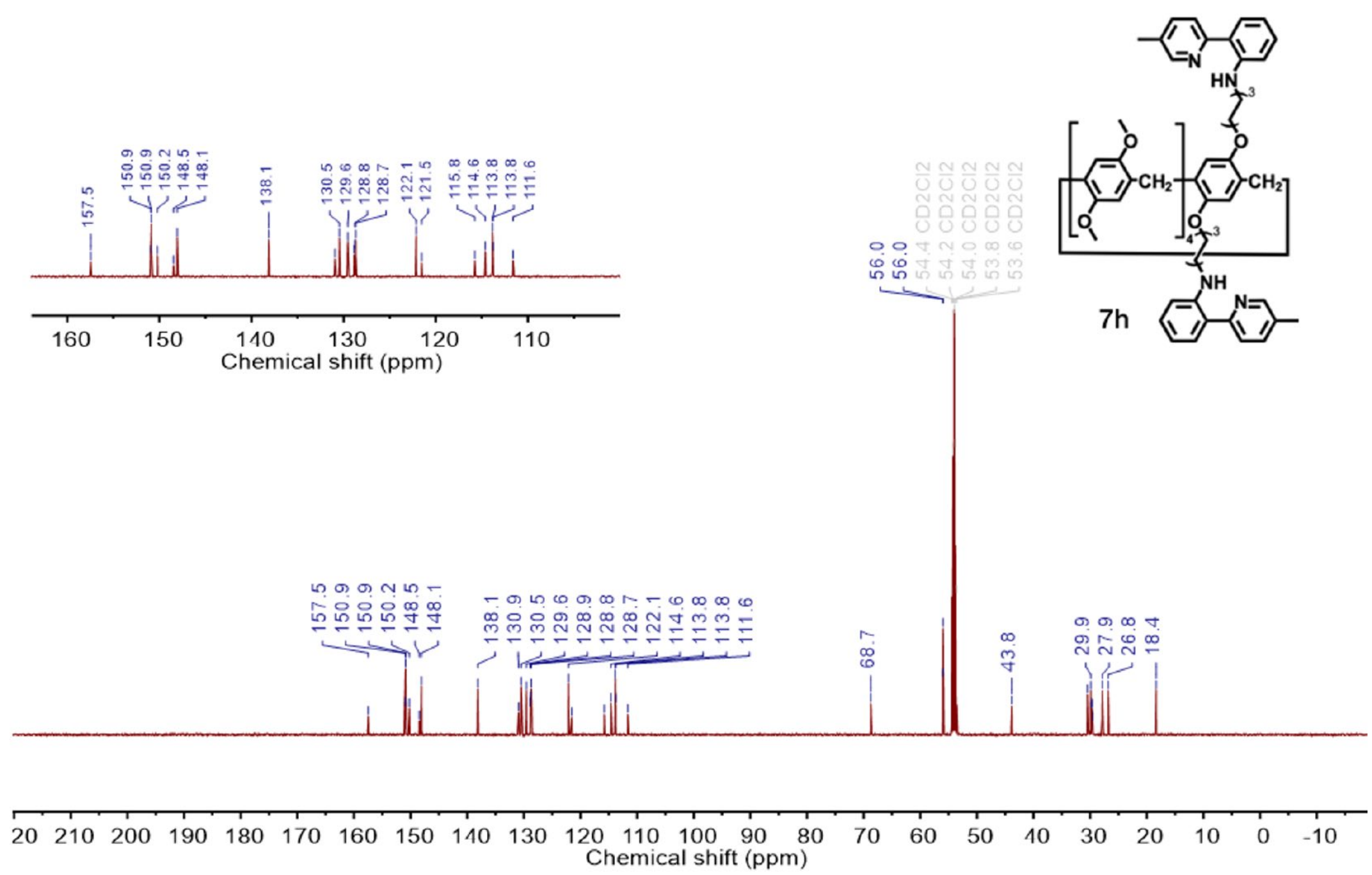

Fig. S56 ^{13}C NMR (126 MHz) spectrum of **7h** in CD_2Cl_2 (T = 298 K).

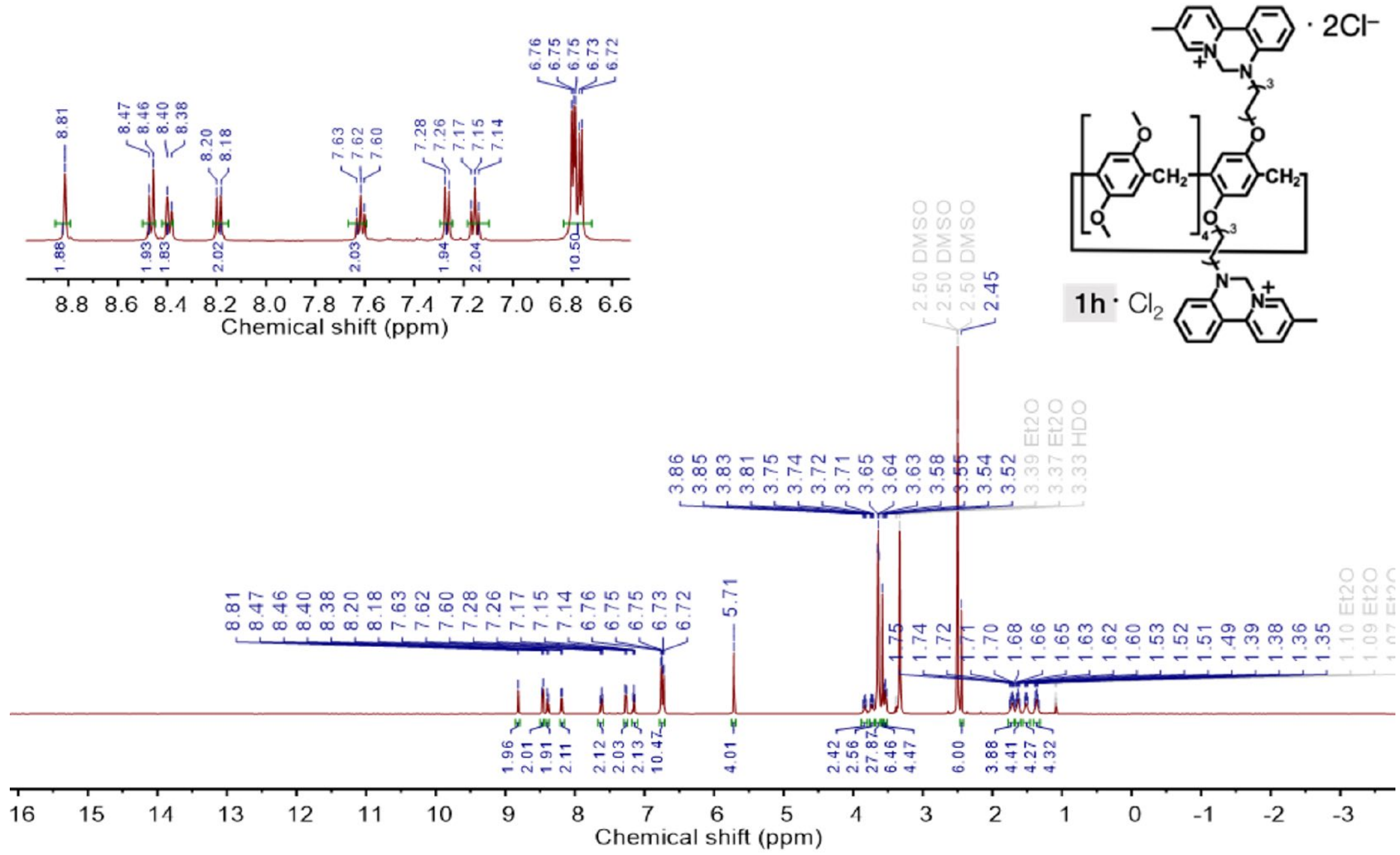

Fig. S57 ^{1}H NMR (500 MHz) spectrum of **1h**·Cl_2 in DMSO−d_6 (T = 298 K).

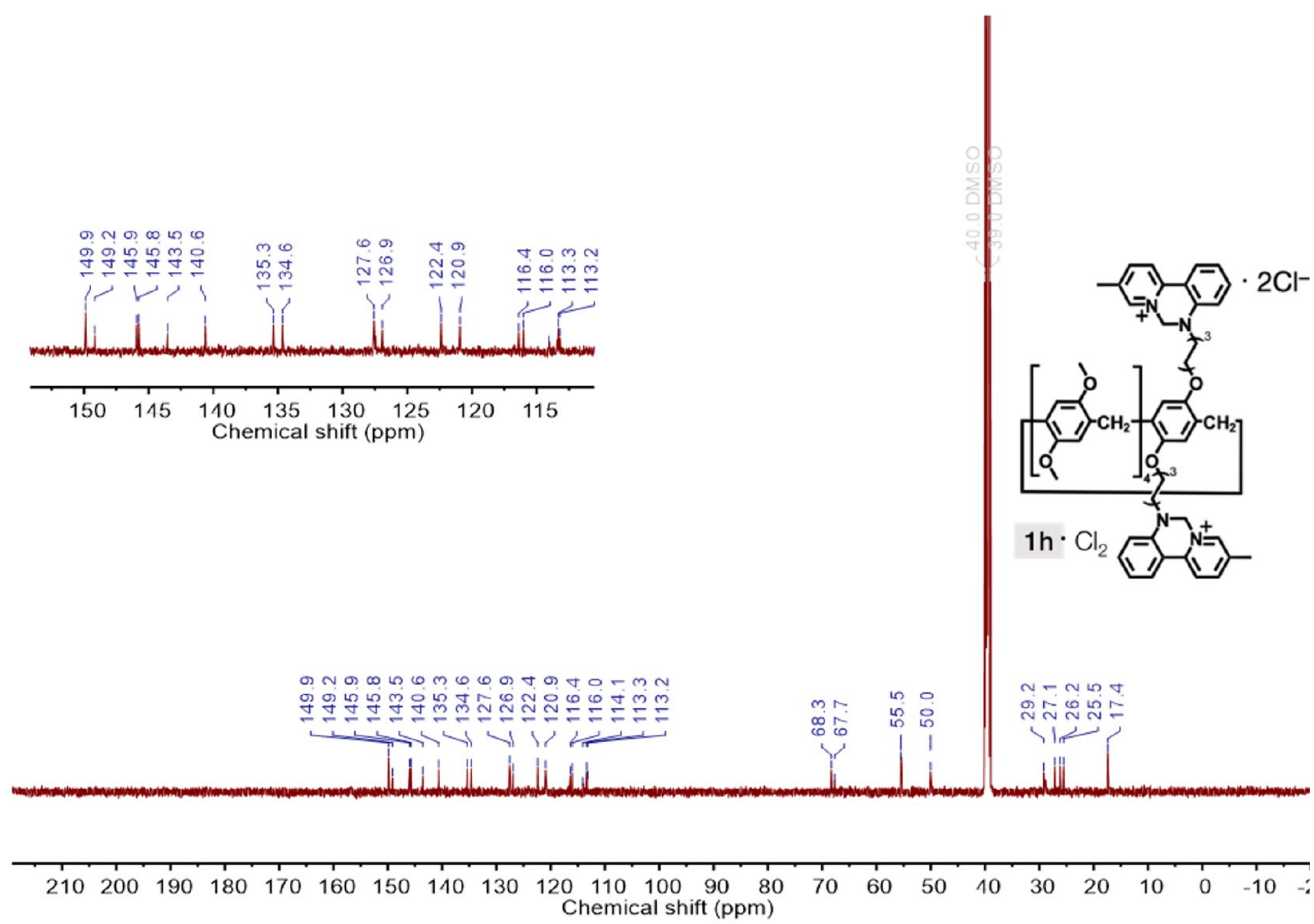

Fig. S58 ^{13}C NMR (126 MHz) spectrum of **1h**·Cl_2 in DMSO−d_6 (T = 298 K).

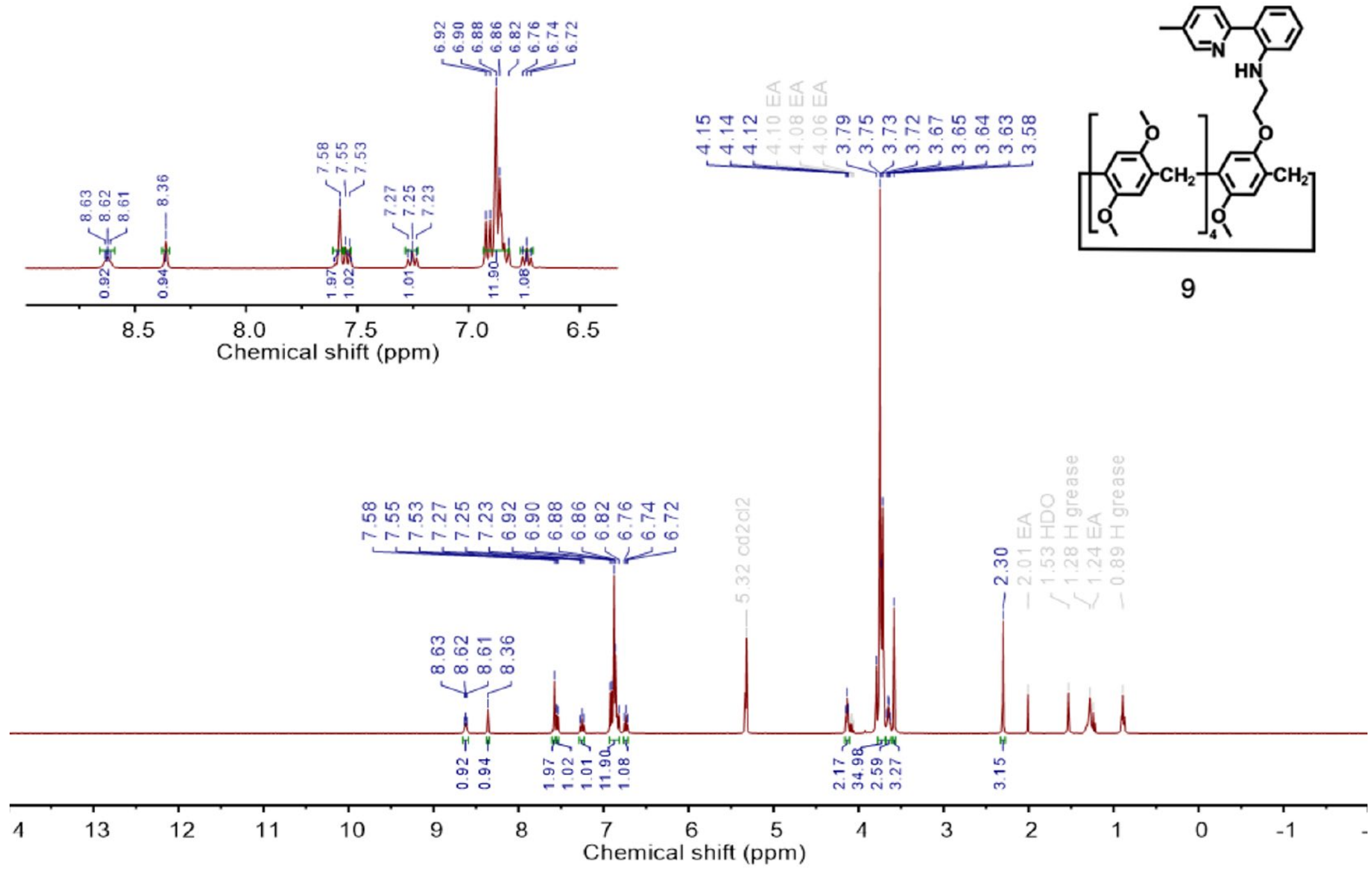

Fig. S59 ^{1}H NMR (500 MHz) spectrum of **9** in CD_2Cl_2 (T = 298 K).

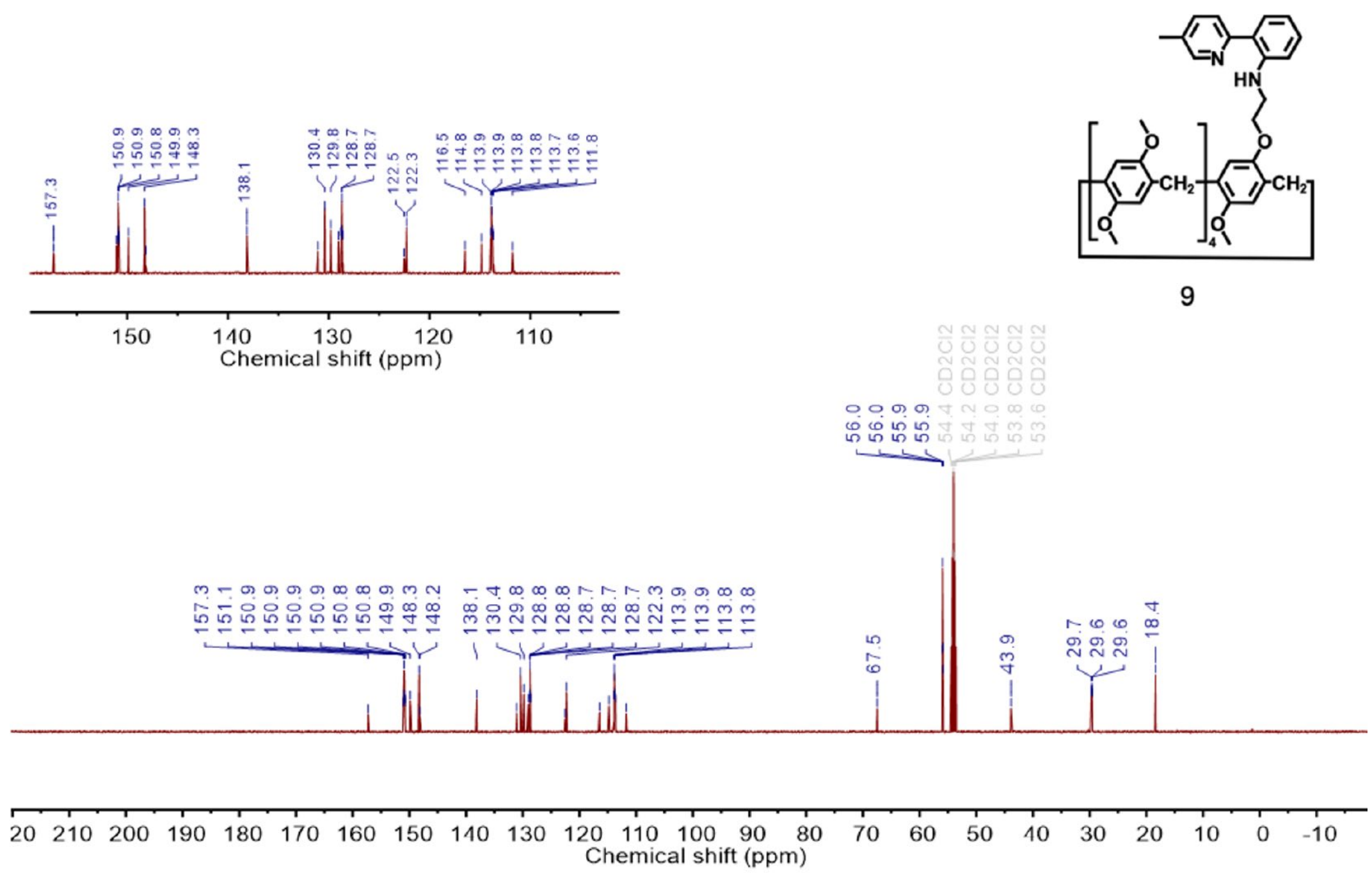

Fig. S60 ^{13}C NMR (126 MHz) spectrum of **9** in CD_2Cl_2 (T = 298 K).

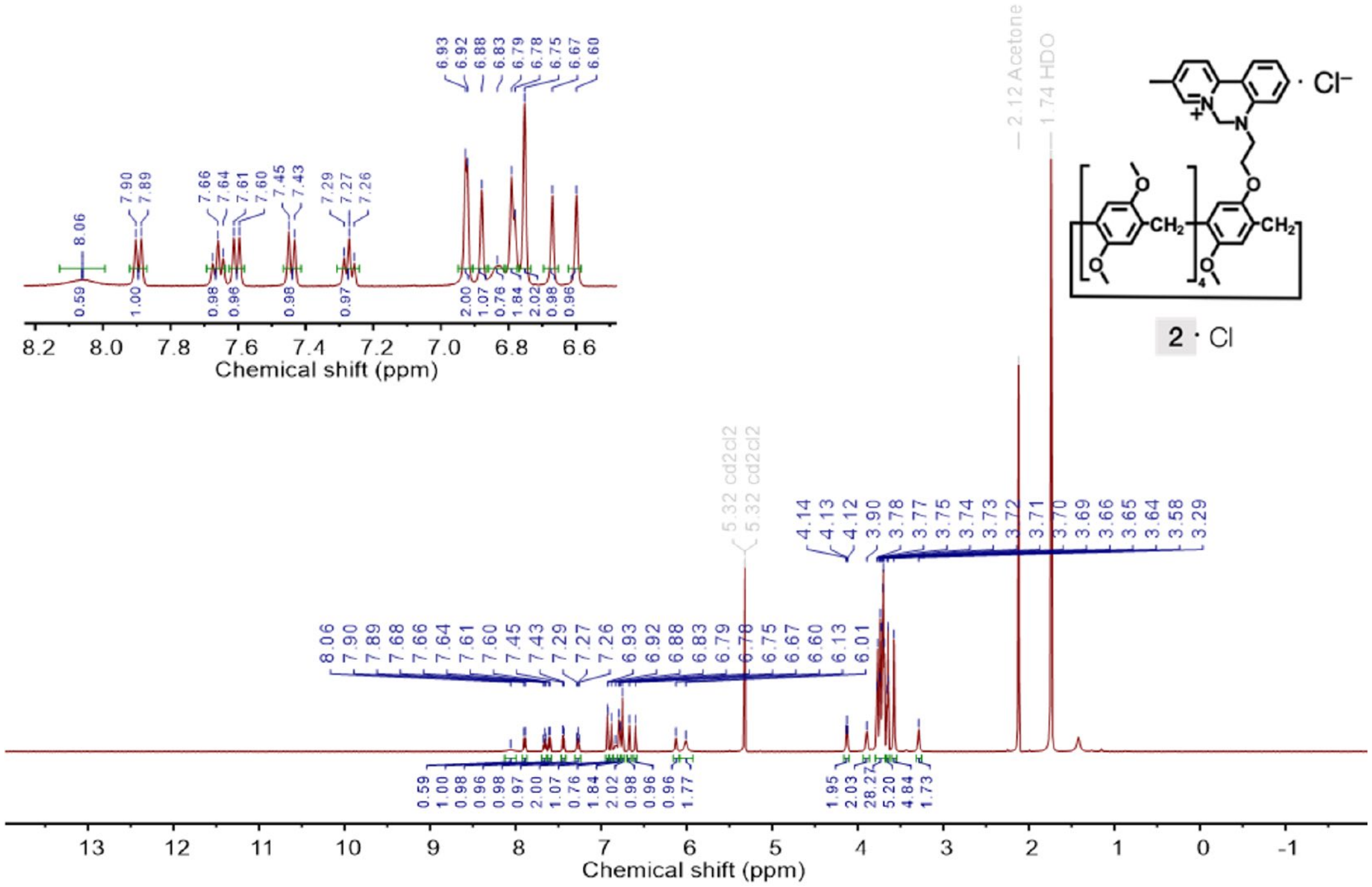

Fig. S61 ^{1}H NMR (500 MHz) spectrum of **2**·Cl in CD_2Cl_2 (T = 298 K).

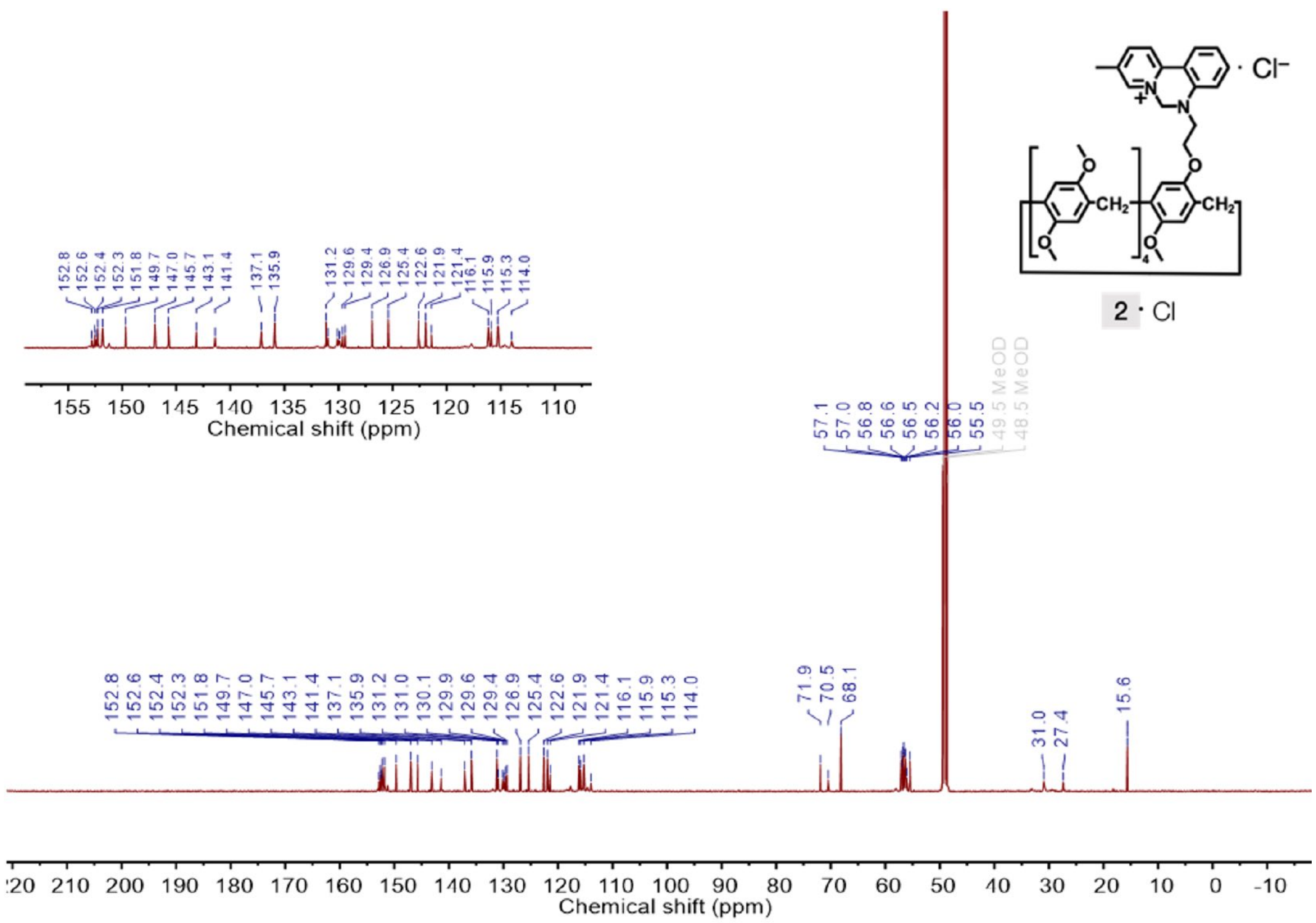

Fig. S62 ^{13}C NMR (126 MHz) spectrum of **2**·Cl in MeOD (T = 298 K).

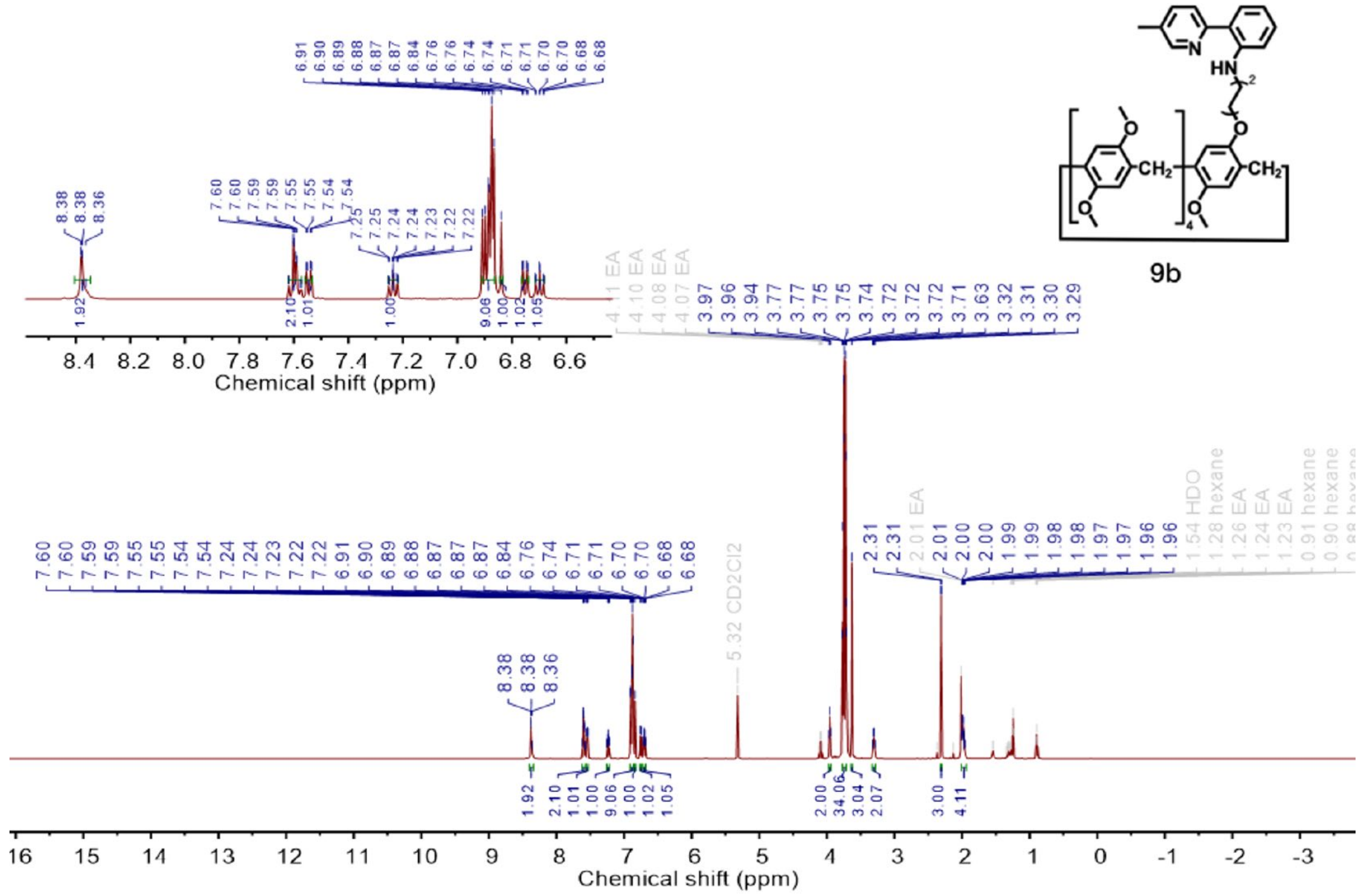

Fig. S63 ^{1}H NMR (500 MHz) spectrum of **9b** in CD_2Cl_2 (T = 298 K).

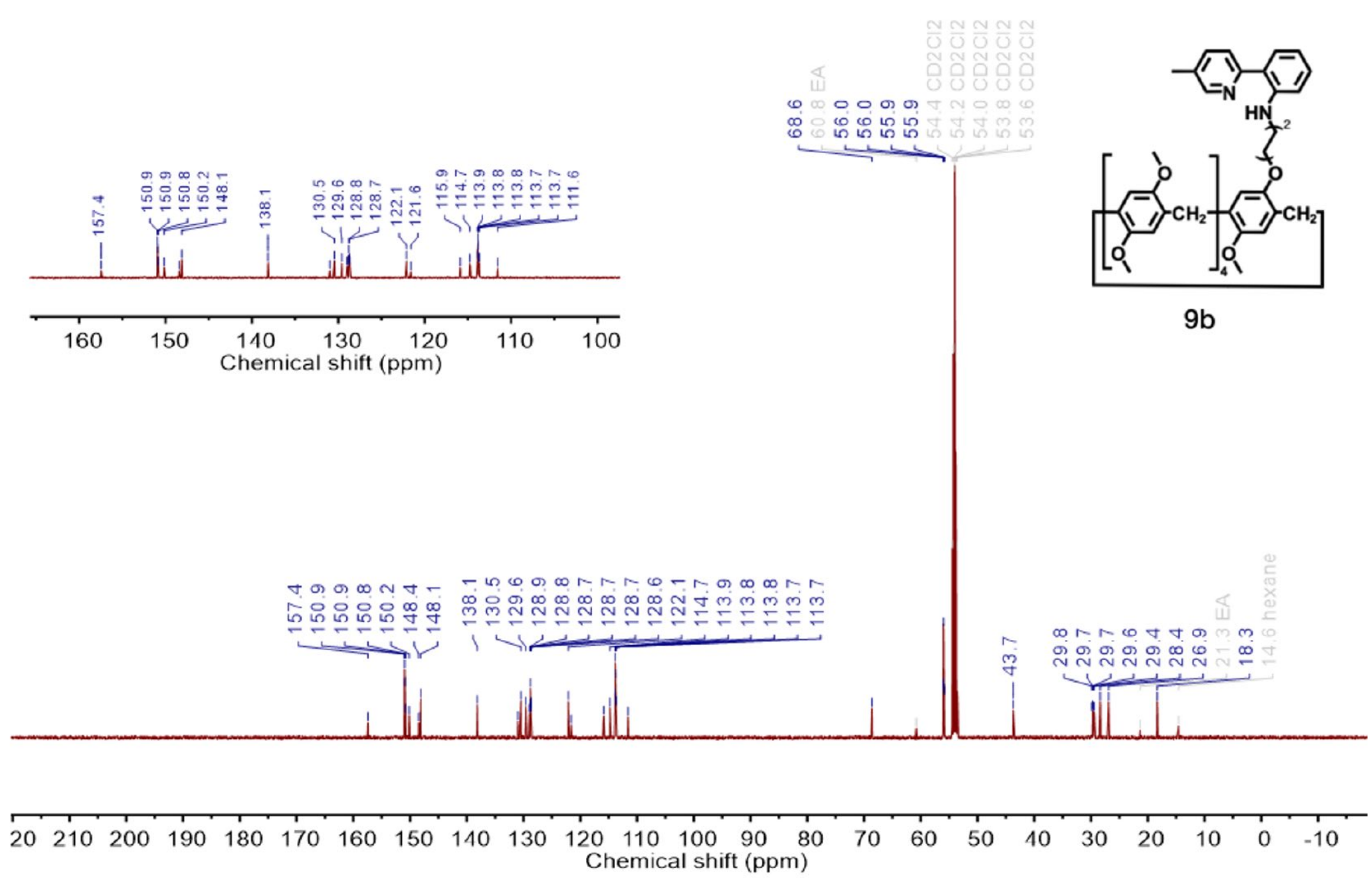

Fig. S64 ^{13}C NMR (126 MHz) spectrum of **9b** in CD_2Cl_2 (T = 298 K).

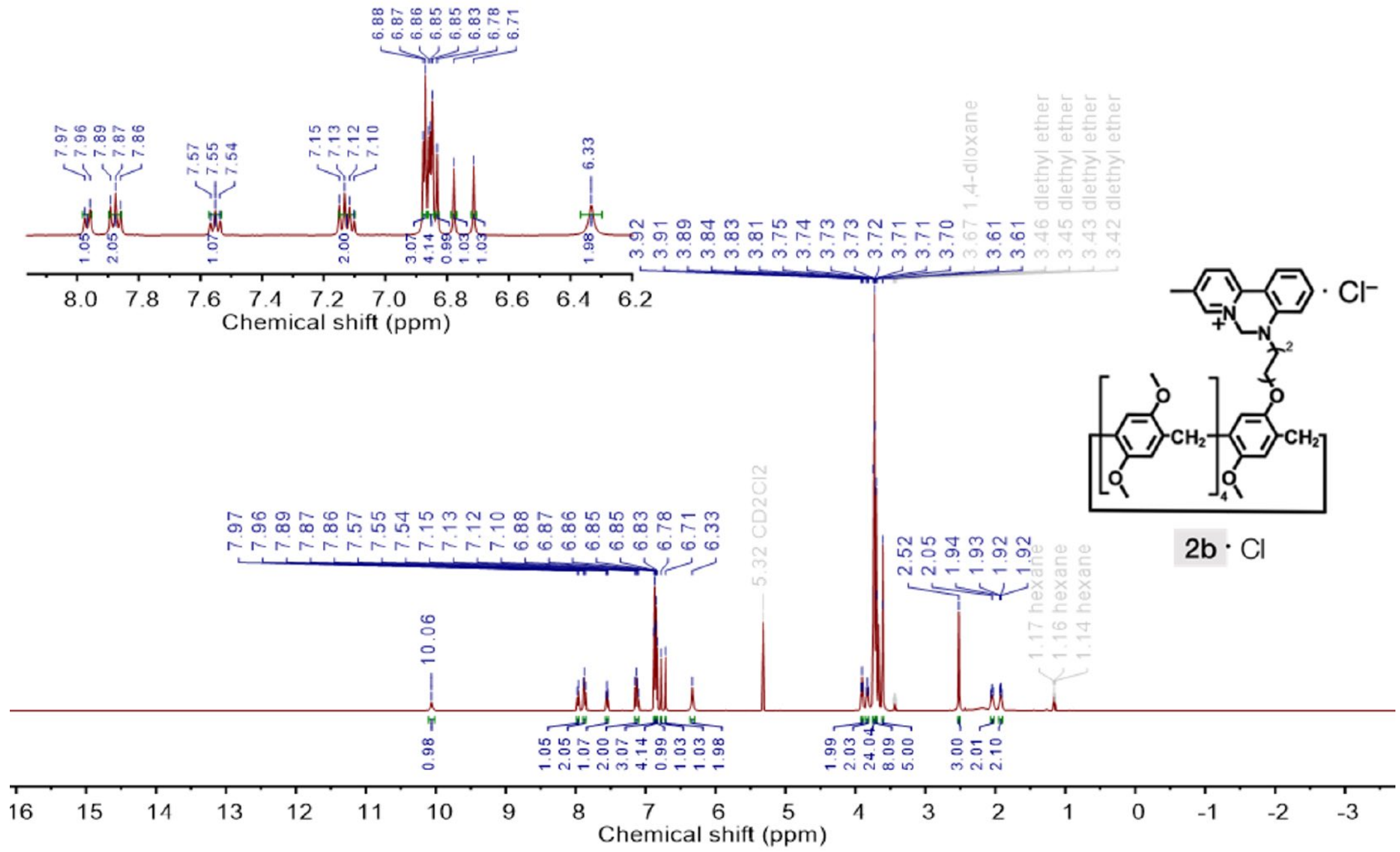

Fig. S65 ^{1}H NMR (500 MHz) spectrum of **2b**·Cl in CD_2Cl_2 (T = 298 K).

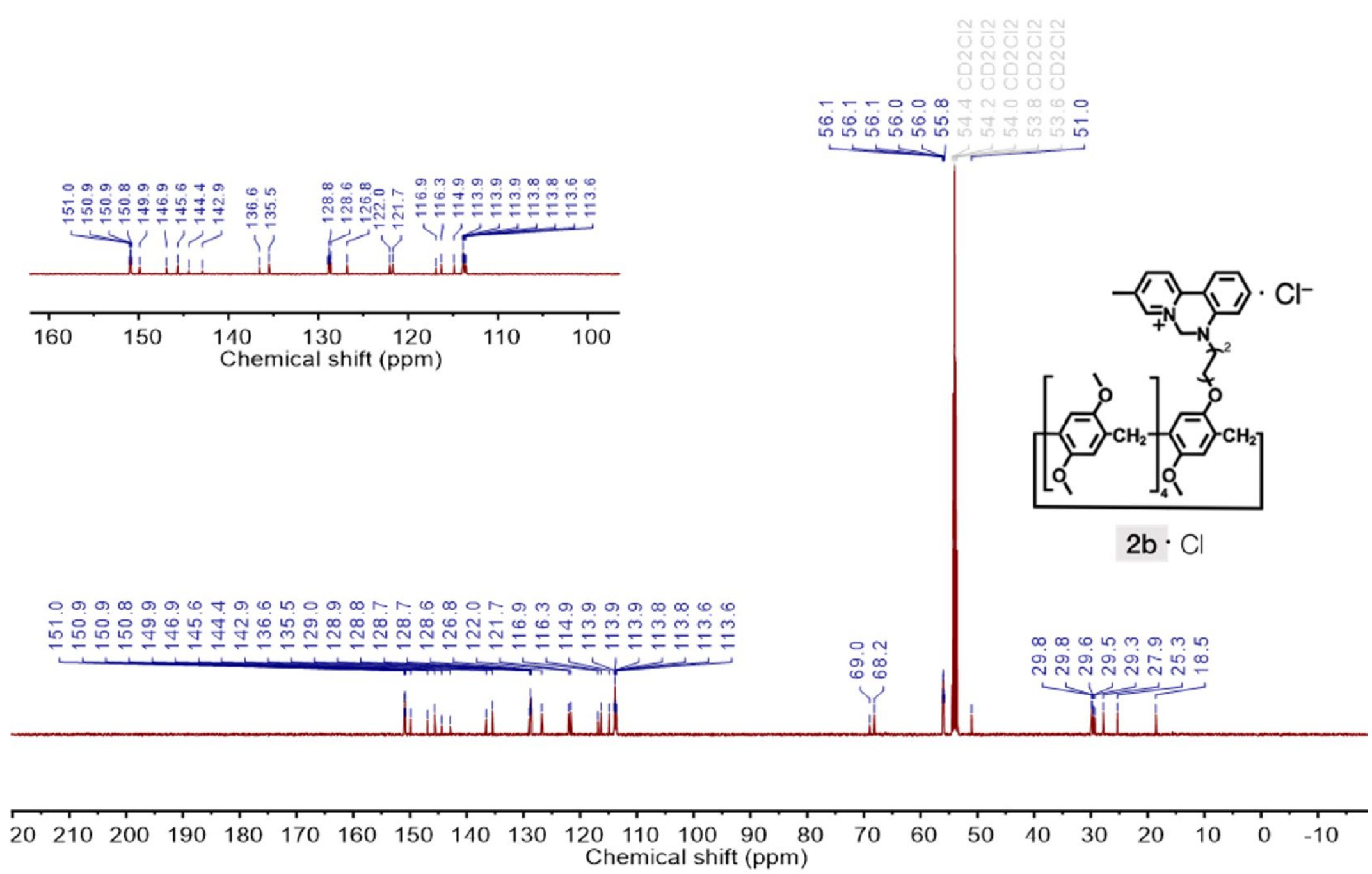

Fig. S66 ^{13}C NMR (126 MHz) spectrum of **2b**·Cl in CD_2Cl_2 (T = 298 K).

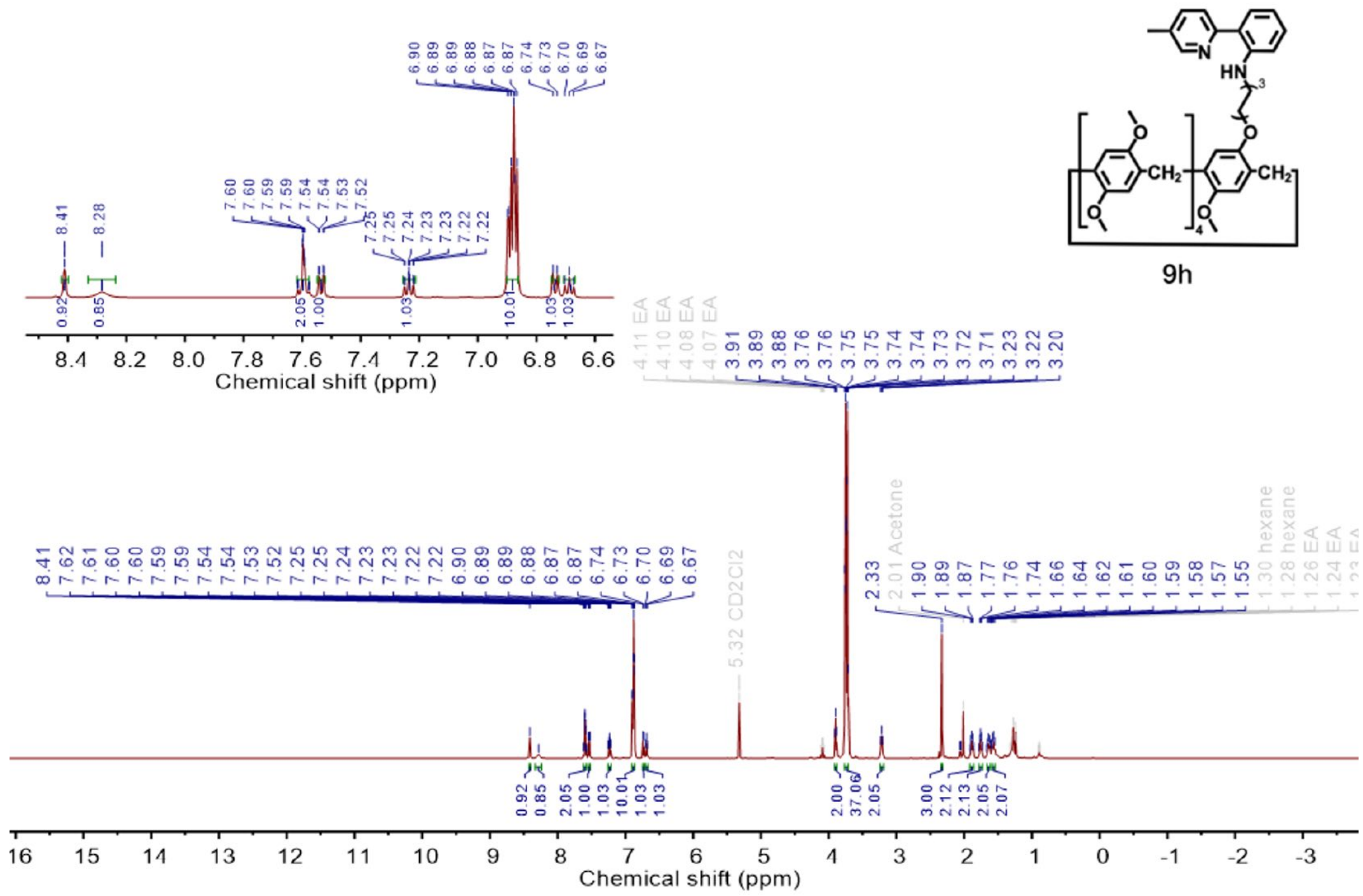

Fig. S67 ^{1}H NMR (500 MHz) spectrum of **9h** in CD_2Cl_2 (T = 298 K).

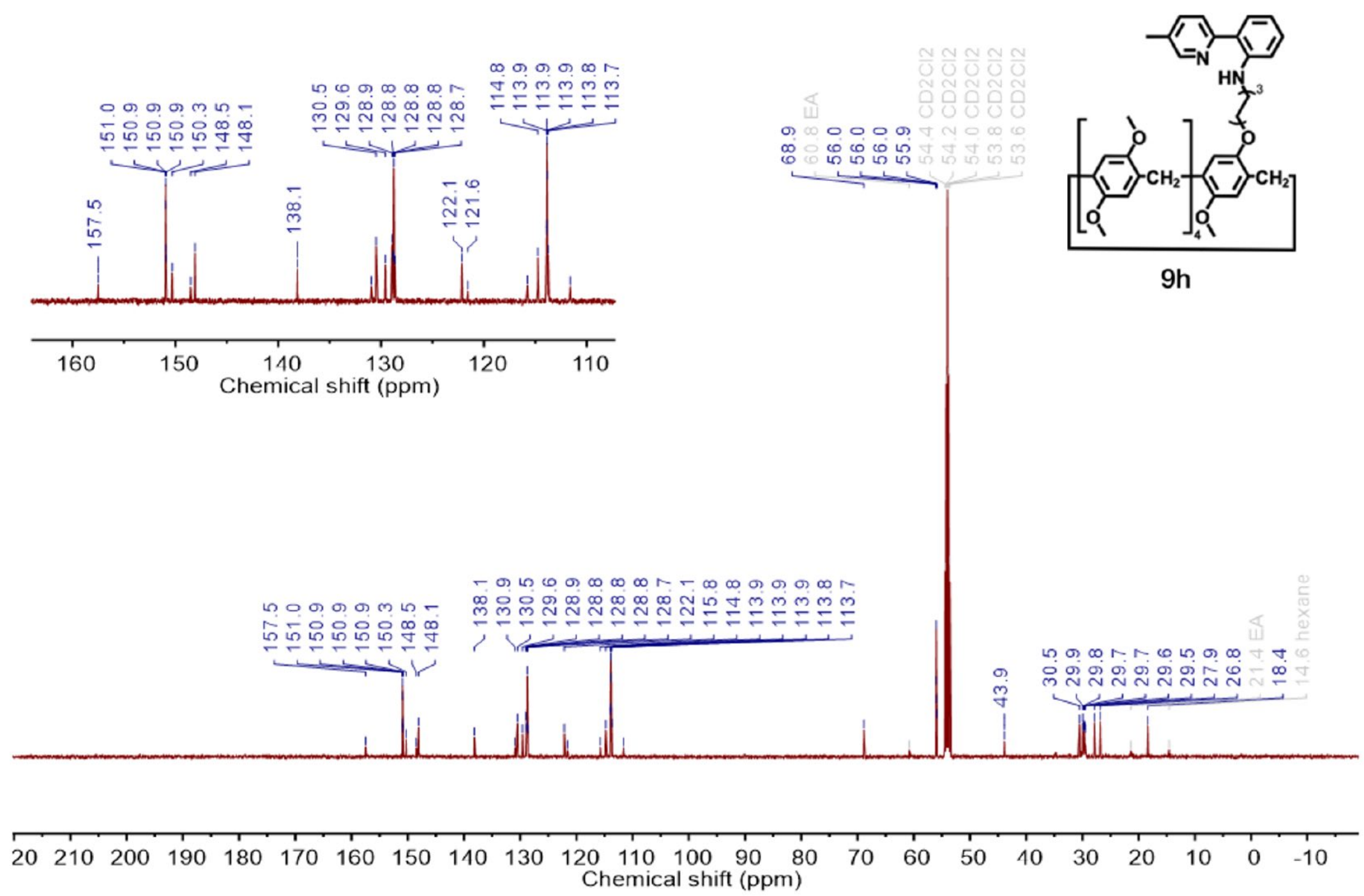

Fig. S68 ^{13}C NMR (126 MHz) spectrum of **9h** in CD_2Cl_2 (T = 298 K).

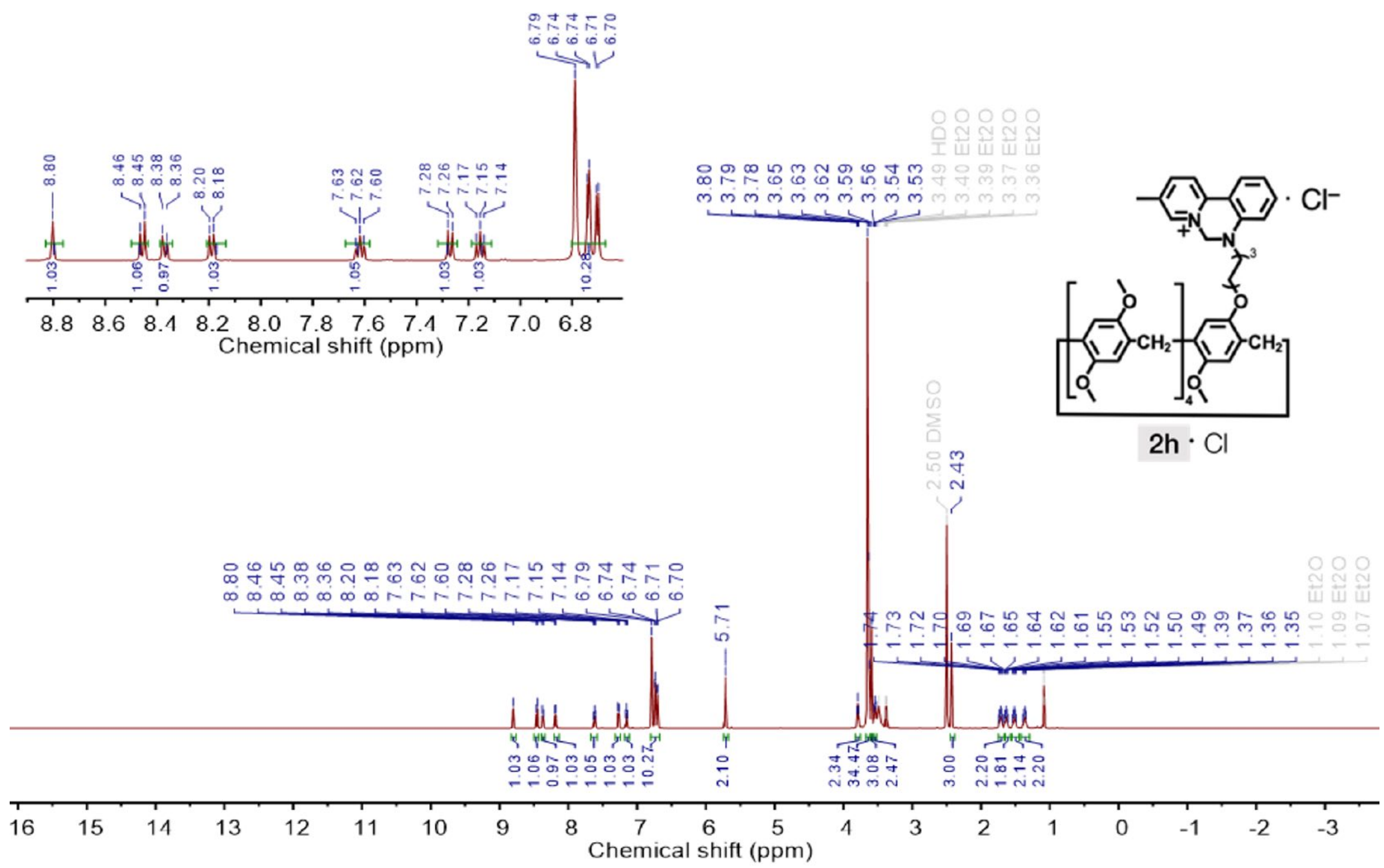

Fig. S69 ^{1}H NMR (500 MHz) spectrum of **2h**·Cl in DMSO−d_6 (T = 298 K).

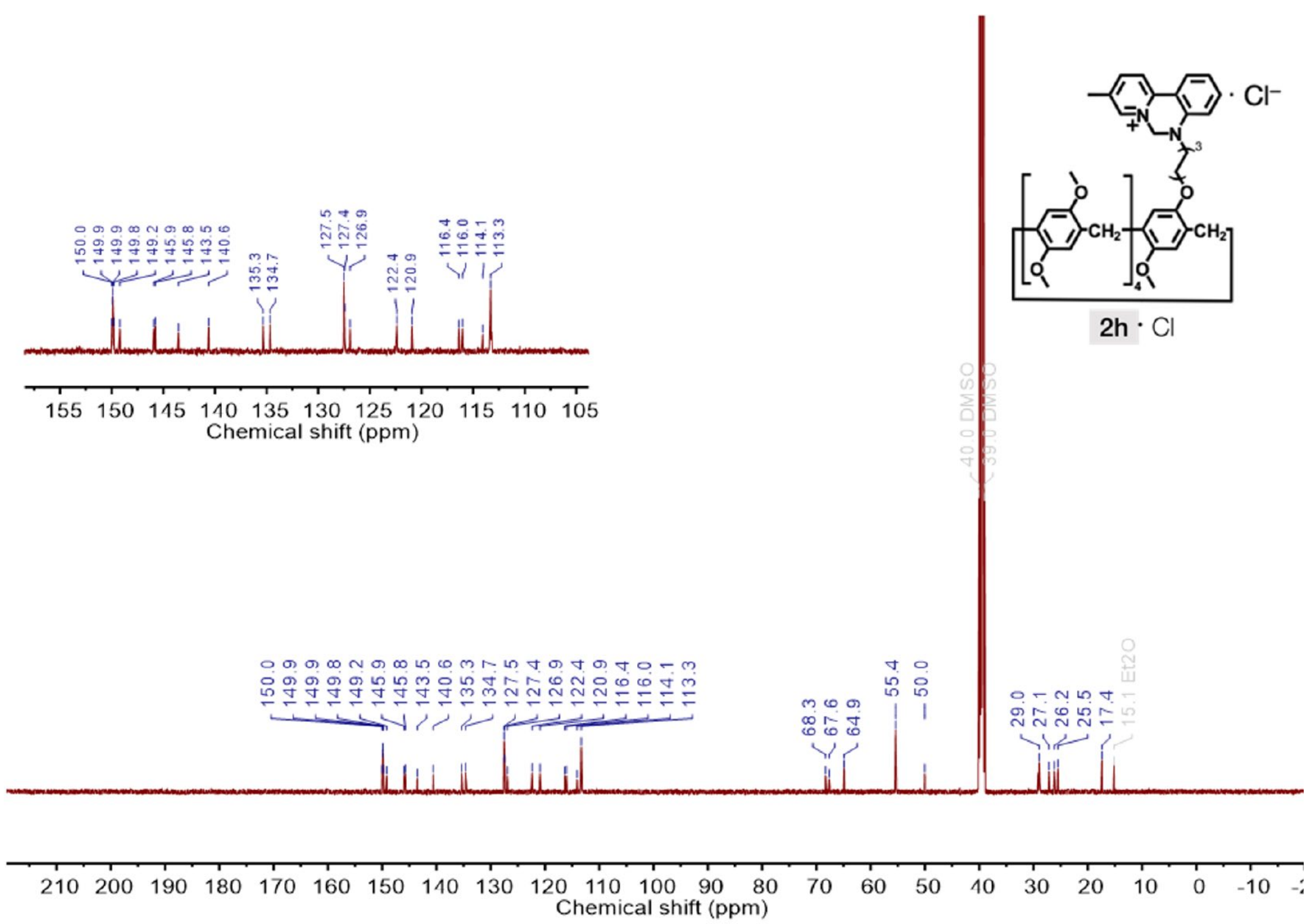

Fig. S70 ^{13}C NMR (126 MHz) spectrum of **2h**·Cl in DMSO−d_6 (T = 298 K).

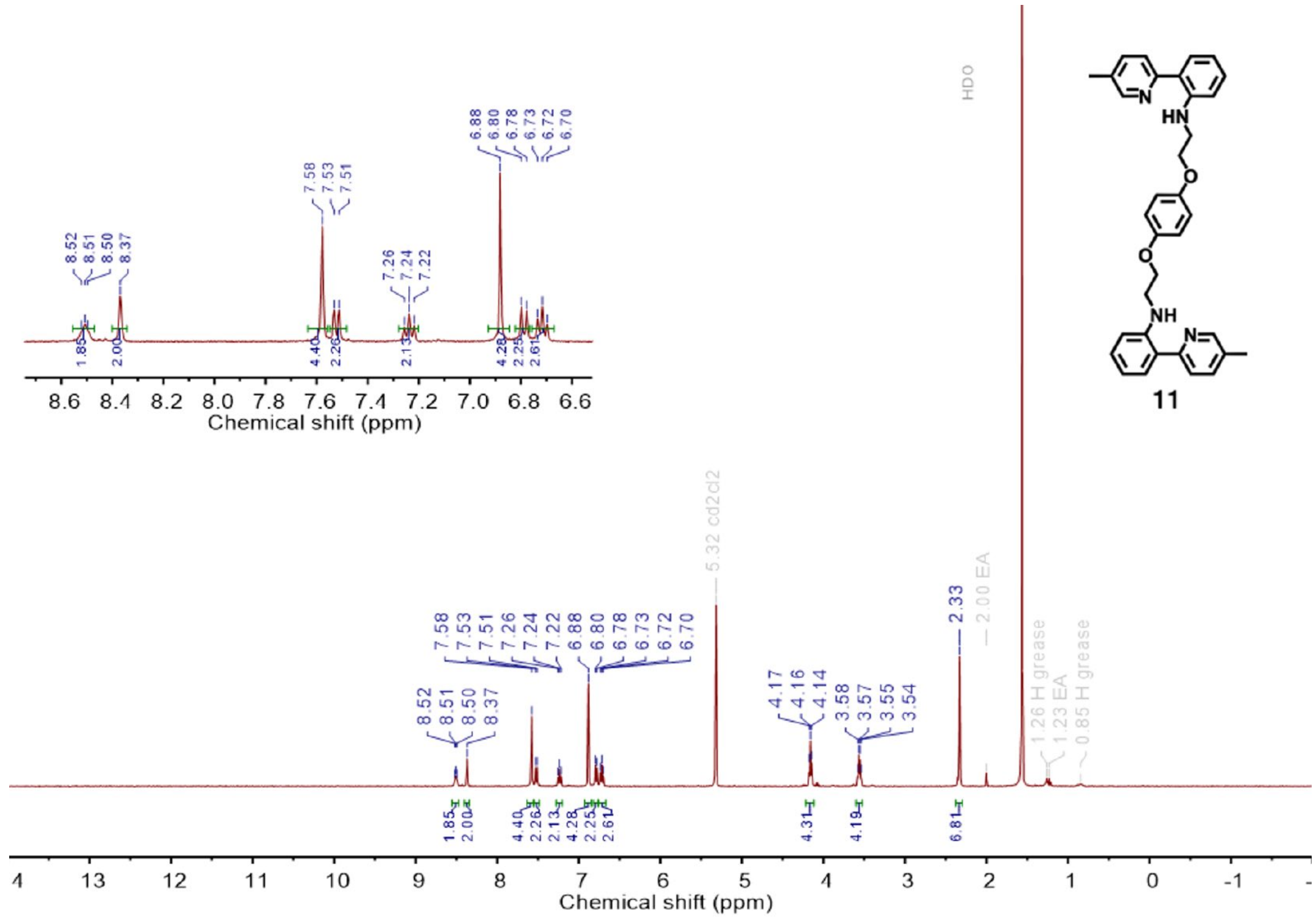

Fig. S71 ^{1}H NMR (500 MHz) spectrum of **11** in CD_2Cl_2 (T = 298 K).

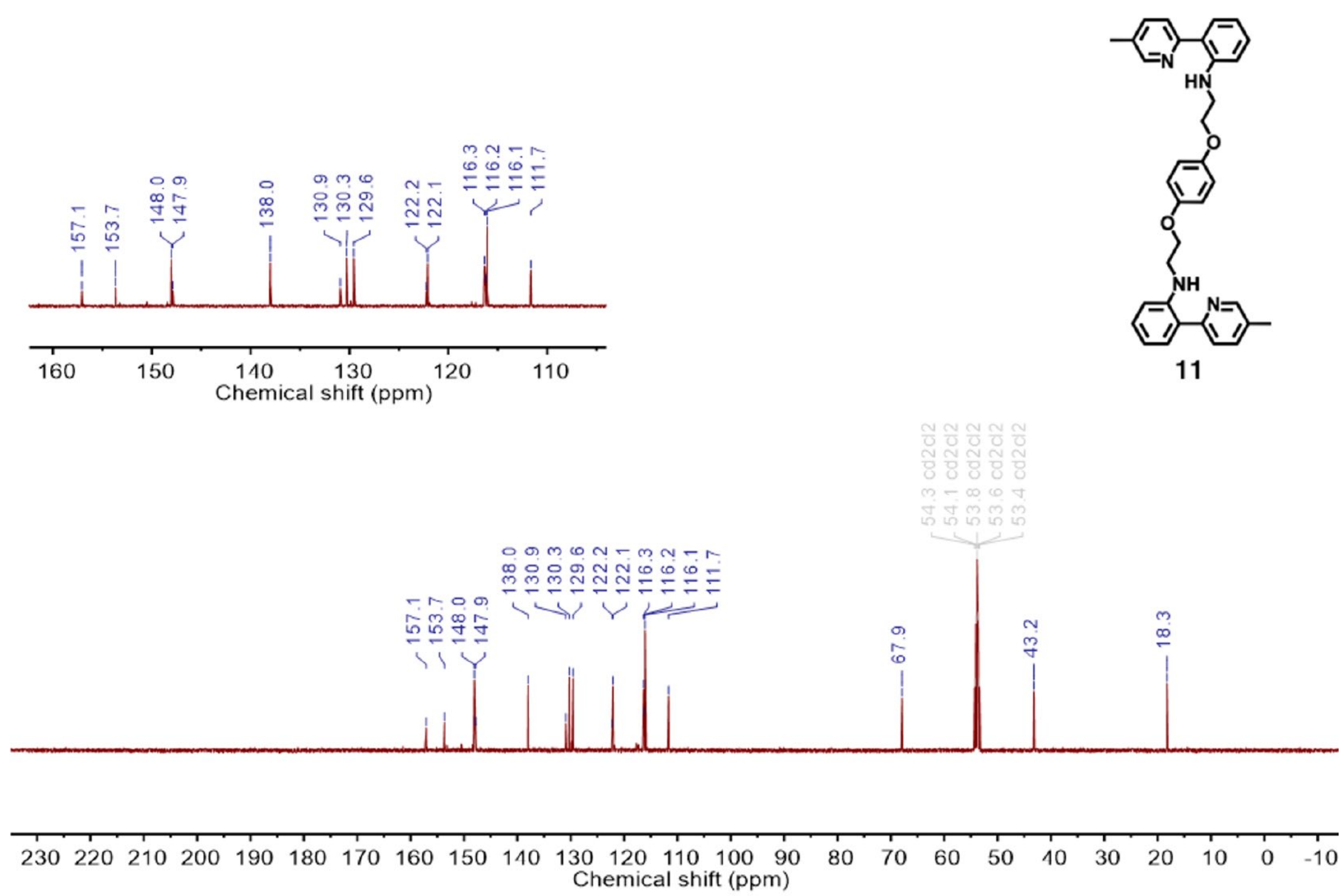

Fig. S72 ^{13}C NMR (126 MHz) spectrum of **11** in CD_2Cl_2 (T = 298 K).

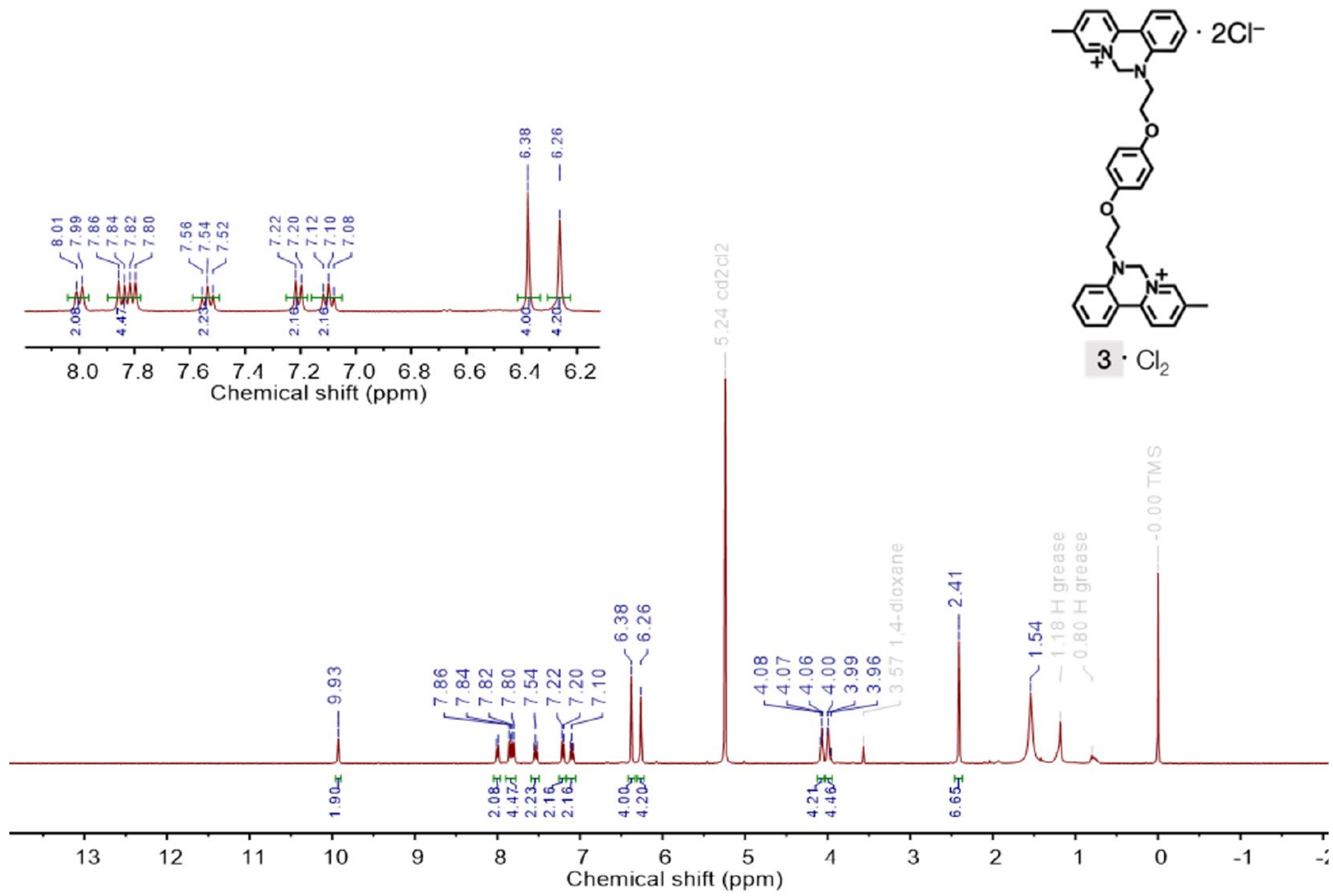

Fig. S73 ^{1}H NMR (500 MHz) spectrum of **3**·Cl_2 in CD_2Cl_2 (T = 298 K).

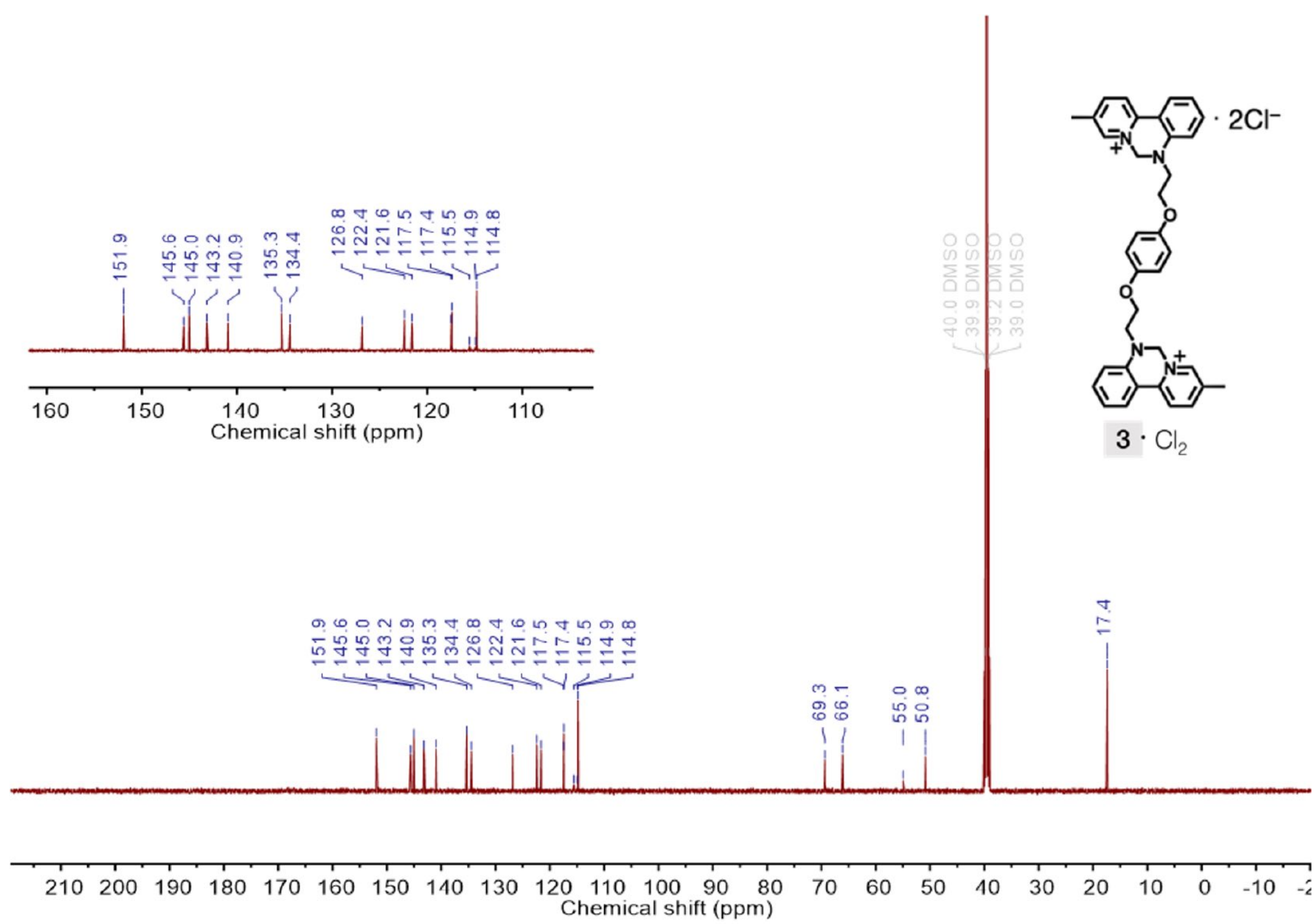

Fig. S74 ^{13}C NMR (126 MHz) spectrum of **3**·Cl_2 in DMSO−d_6 (T = 298 K).

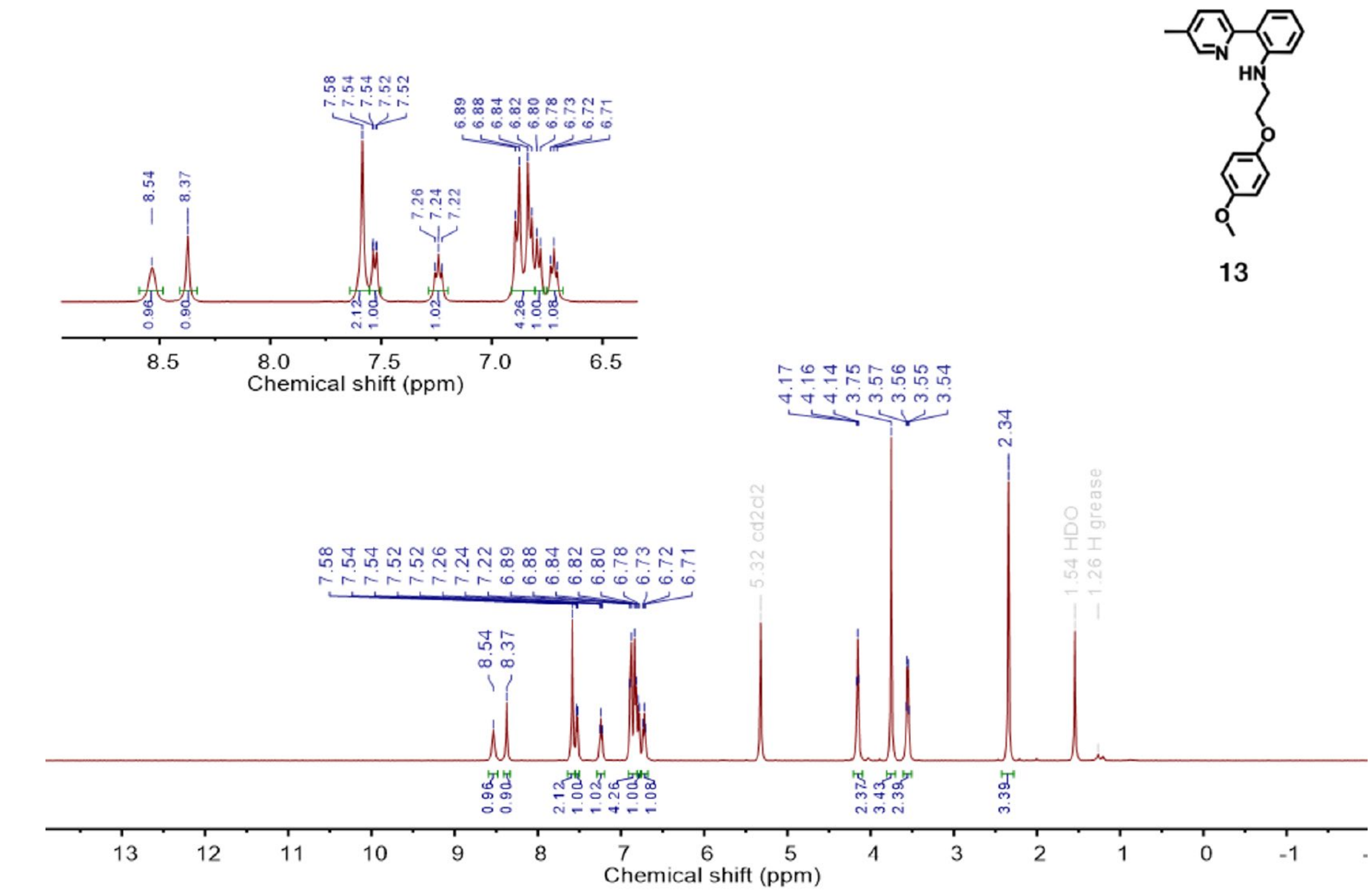

Fig. S75 ^{1}H NMR (500 MHz) spectrum of **13** in CD_2Cl_2 (T = 298 K).

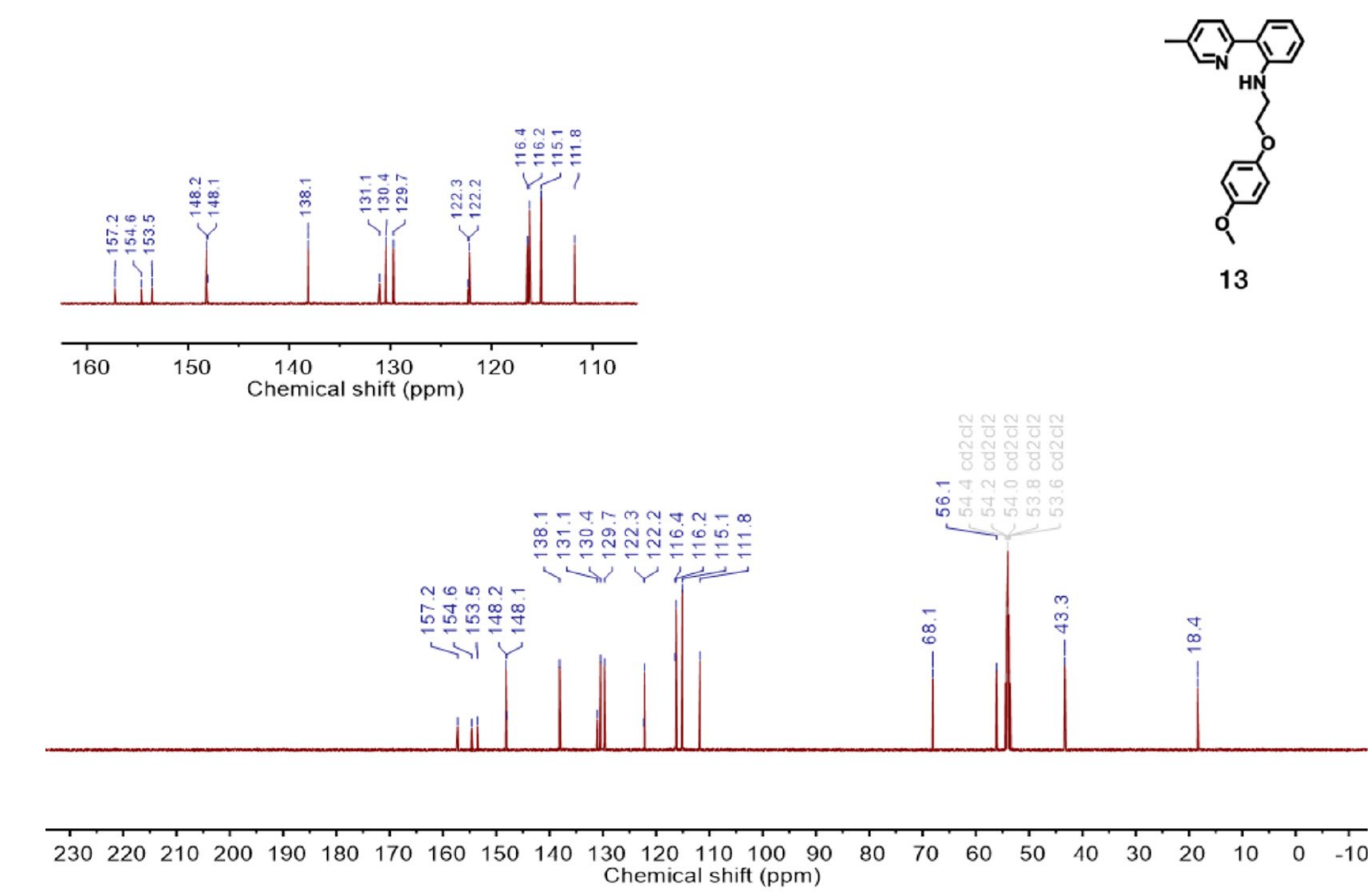

Fig. S76 ^{13}C NMR (126 MHz) spectrum of **13** in CD_2Cl_2 (T = 298 K).

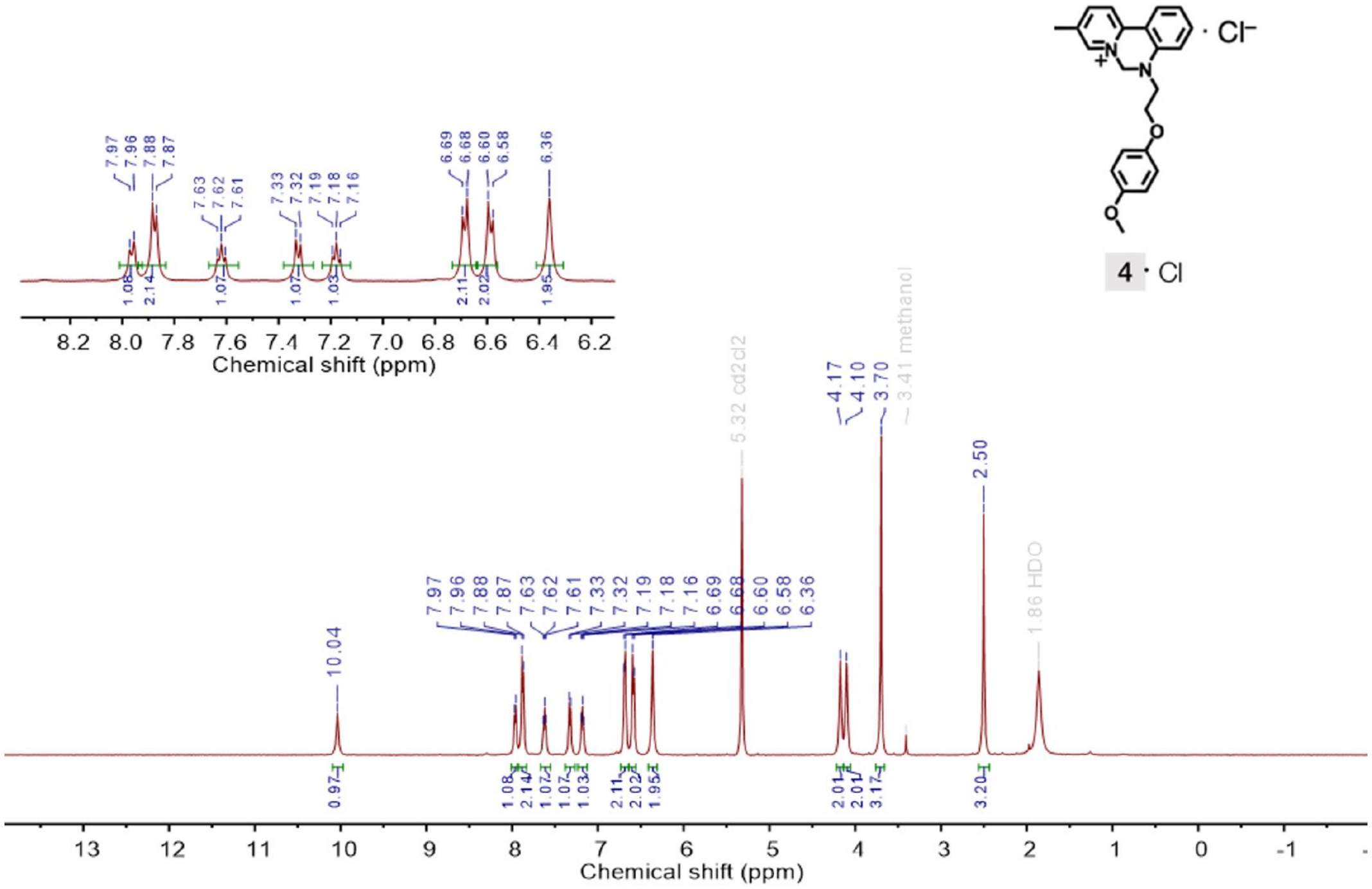

Fig. S77 ^{1}H NMR (500 MHz) spectrum of **4**·Cl in CD_2Cl_2 (T = 298 K).

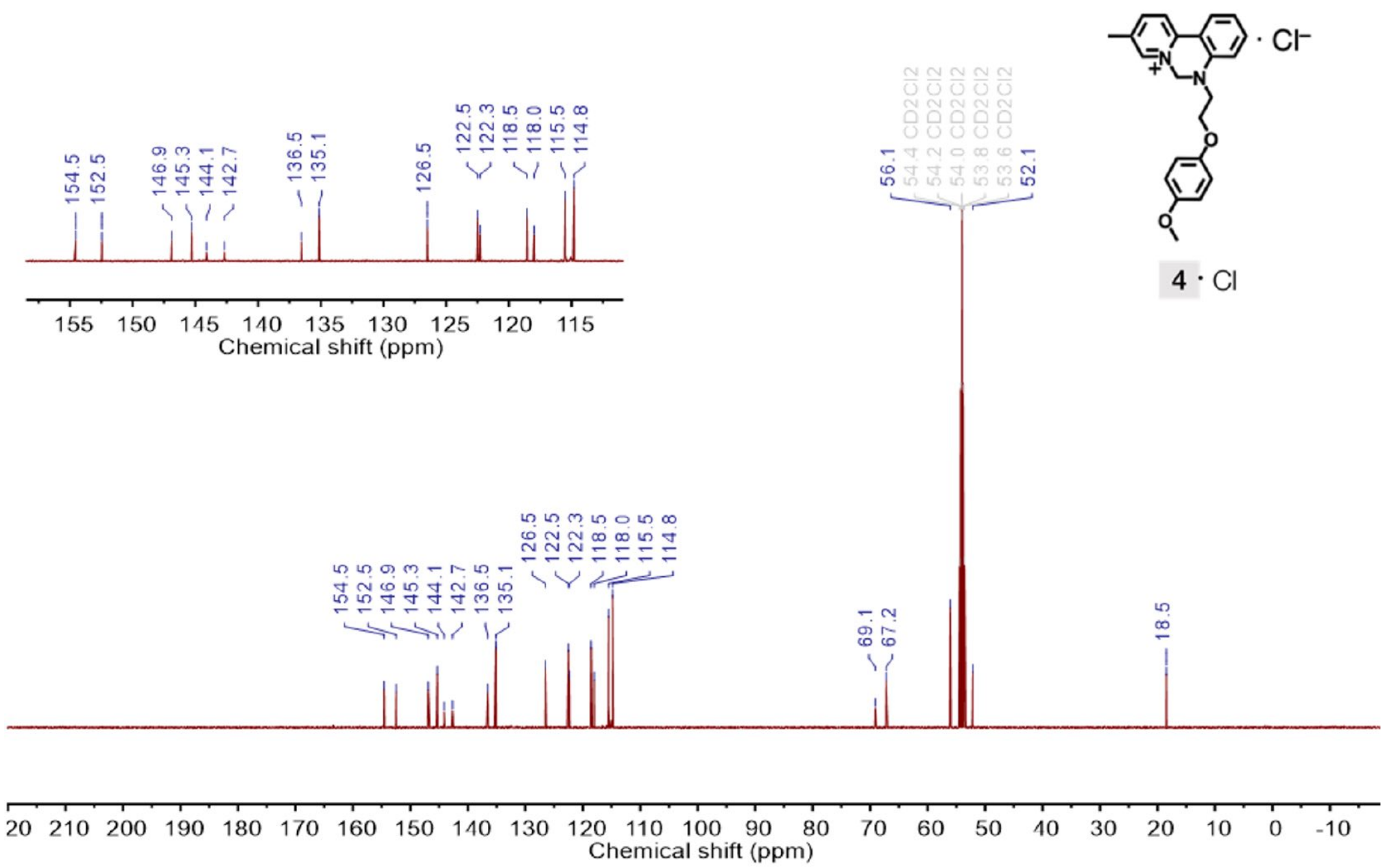

Fig. S78 ^{13}C NMR (126 MHz) spectrum of **4**·Cl in CD_2Cl_2 (T = 298 K).

8. Cartesian Coordinates of DFT Computational Models

Table S8. Cartesian coordinates of the DFT energy-minimized structure of **ANIPY-Me** at $S_{0,min}$. CPCM model with MeOH.

Atom	x	y	z	Atom	x	y	z
C	2.977506	–2.094858	0.135217	H	3.728804	1.187004	–0.405938
C	3.731183	–0.939754	–0.122910	H	1.013017	–2.887958	0.452157
C	3.122094	0.307661	–0.230180	H	–2.690830	1.860374	0.364741
C	1.728458	0.424717	–0.081280	H	–3.411884	–2.287560	–0.520276
C	0.952279	–0.750566	0.133141	H	–0.981877	–2.673170	–0.412419
C	1.597374	–1.995851	0.254750	H	0.106887	1.549493	1.737942
C	–0.500644	–0.638068	0.087806	H	–0.637194	2.642085	0.533104
N	–1.047148	0.598121	0.277384	H	2.390426	2.798403	–1.276356
C	–2.381480	0.836833	0.194243	H	1.040238	3.681190	–0.557188
C	–3.281688	–0.174603	–0.084092	H	2.398127	3.157031	0.471655
C	–2.745898	–1.462631	–0.286934	H	4.810050	–1.009588	–0.222570
C	–1.382104	–1.687891	–0.214214	C	–4.759714	0.092297	–0.169848
N	1.046499	1.632162	–0.167881	H	–5.139486	–0.178269	–1.160333
C	–0.123138	1.693474	0.670863	H	–4.990136	1.144233	0.012044
C	1.766186	2.884950	–0.384897	H	–5.299655	–0.511988	0.566295
H	3.465574	–3.057522	0.240639				

Table S9. Cartesian coordinates of the DFT energy-minimized structure of **ANIPY-Me** at $S_{1,min}$. CPCM model with MeOH.

Atom	x	y	z	Atom	x	y	z
C	2.940267	–2.152747	–0.115775	H	3.798122	1.120618	–0.178358
C	3.752474	–1.018316	–0.178896	H	0.942062	–2.927993	0.001867
C	3.168064	0.242918	–0.129482	H	–2.695893	1.891685	–0.467599
C	1.762888	0.381111	–0.010811	H	–3.530379	–2.263577	0.374283
C	0.921109	–0.784982	0.045721	H	–1.082026	–2.667045	0.464055
C	1.539170	–2.024784	–0.017554	H	–0.355081	2.018707	1.346377
C	–0.543772	–0.636933	0.127916	H	–0.558061	2.675146	–0.289814
N	–1.035126	0.661006	–0.089756	H	2.537241	2.955464	–0.875813
C	–2.404186	0.875751	–0.230543	H	1.331715	3.704278	0.191898
C	–3.314906	–0.141331	–0.079900	H	2.705767	2.831572	0.899574
C	–2.837338	–1.444210	0.228626	H	4.828772	–1.111509	–0.266524
C	–1.450454	–1.662275	0.296393	C	–4.787749	0.115176	–0.257363
N	1.194125	1.625146	0.050429	H	–5.186210	–0.485723	–1.082803
C	–0.223593	1.801155	0.274195	H	–4.997297	1.167425	–0.465171
C	1.995453	2.850547	0.068759	H	–5.335651	–0.174900	0.646199
H	3.379359	–3.143695	–0.150595				

Table S10. Cartesian coordinates of the DFT energy-minimized structure of $\mathbf{1}_{in\text{-}out}$. CPCM model with MeOH.

Atom	x	y	z	Atom	x	y	z
O	–4.187156	–0.996469	1.124795	H	–9.262270	1.579060	1.400049
O	1.024029	4.467957	2.614936	C	–2.027559	–0.424930	1.925846
O	5.182678	–2.296018	–1.276157	C	–7.732689	0.758003	0.134618
O	1.037091	–2.460079	2.252306	C	4.713428	2.254178	–3.240904
O	5.188708	3.000452	–0.590395	H	5.718692	2.409066	–2.844270
O	2.156969	–5.311295	1.241338	H	4.705742	2.608522	–4.275065
O	–2.553604	1.582319	–0.470868	C	4.609005	0.248243	5.946003
O	0.806874	–2.948768	–3.589672	H	4.929915	0.222334	6.982425
O	2.962824	1.080078	–5.071483	C	1.154904	4.197663	4.009819
O	0.359935	4.608662	–2.792946	H	1.429001	3.151578	4.195596
N	–6.411269	0.857331	–0.184773	H	0.231414	4.430260	4.552504
N	–6.403165	–1.404546	–1.052441	H	1.957891	4.844251	4.364532
N	3.113426	–0.837114	1.242713	C	–8.518969	–2.618929	–1.304442
N	3.735569	–2.113282	3.215807	H	–7.982798	–3.491996	–1.660230
C	2.450821	–3.849146	–2.160537	C	2.774065	–3.099151	3.730083
C	–1.665057	2.079959	1.671012	H	3.021786	–3.322531	4.770023
C	–0.765642	2.808316	2.455023	H	2.890618	–4.017158	3.153571
H	–0.682268	2.551776	3.504212	C	4.349594	–0.943951	5.281734
C	–1.088347	–3.038844	1.331991	H	4.476217	–1.893435	5.789883
C	–2.398336	–2.658973	1.025709	C	–7.396620	2.604217	1.661385
H	–3.041674	–3.382004	0.538187	H	–7.798076	3.290413	2.400230
C	0.517319	–4.287830	–0.135700	C	–6.036281	2.679209	1.310808
C	1.102246	–3.508737	–2.372913	C	4.488144	1.477767	5.281379
C	–0.115648	4.187078	0.563884	H	4.716159	2.403782	5.797652
C	–2.880612	–1.381417	1.347179	C	3.916051	–1.972924	1.803736
C	4.679248	–1.442819	–2.236181	H	3.623747	–2.876882	1.277921
C	3.061105	4.196457	–0.382302	H	4.958798	–1.731279	1.585477
H	3.248655	4.465982	0.650448	C	1.712457	1.272336	0.256102
C	3.825031	–2.007583	–3.196698	H	1.161665	2.116971	–0.131821
C	1.845533	–4.704769	0.047231	C	1.324258	–2.648906	3.643285
C	0.037236	3.820484	1.910503	H	0.667428	–3.419191	4.068572
C	–1.746082	2.390478	0.302033	H	1.167791	–1.713981	4.197489
C	–0.530857	–4.399976	0.961165	C	6.439403	–1.953238	–0.690912
H	–1.349692	–5.045615	0.625686	H	6.363332	–1.081982	–0.029355
H	–0.082504	–4.870681	1.837847	H	7.189745	–1.744477	–1.461979
C	2.510357	3.474336	–3.002863	H	6.745799	–2.819203	–0.102348
H	2.315385	3.176991	–4.024451	C	2.400595	1.399867	1.450135
C	3.119556	0.319316	1.965519	H	2.357256	2.331508	1.996229
C	–2.500976	0.973468	2.283527	C	–10.607522	–1.471568	–0.844843
H	–3.544748	1.080832	1.990388	H	–11.691929	–1.468948	–0.836946
H	–2.472896	1.085372	3.373087	C	5.560264	3.417125	0.717656
C	0.164384	–3.707106	–1.358183	H	5.598356	4.510092	0.799893
H	–0.862923	–3.388766	–1.490906	H	6.553739	3.004280	0.895786
C	3.253939	–1.163168	–4.154918	H	4.872233	3.024984	1.474379

Atom	x	y	z	Atom	x	y	z
H	2.579273	–1.602126	–4.877255	C	1.693788	0.061542	–0.446174
C	4.965228	–0.073374	–2.264628	C	–9.907134	–2.618361	–1.249249
H	5.620158	0.368596	–1.522753	H	–10.452612	–3.506807	–1.550781
C	–0.262513	–2.095956	1.955097	C	4.070455	1.506672	3.959564
C	4.399650	0.767875	–3.224784	H	3.963759	2.461613	3.457611
C	–1.024991	3.464624	–0.218467	C	–9.909224	–0.339761	–0.453851
H	–1.107576	3.734098	–1.261976	H	–10.452492	0.544182	–0.138347
C	–8.500465	–0.329637	–0.469132	C	3.389375	–6.026129	1.328609
C	1.836119	4.572734	–0.943839	H	4.255653	–5.352970	1.322408
C	2.795815	–4.464449	–0.954697	H	3.490979	–6.747069	0.509370
H	3.835562	–4.722786	–0.798067	H	3.361540	–6.555299	2.281850
C	1.570302	4.208884	–2.276130	C	1.946512	0.592895	–5.942551
C	–0.716263	–0.807577	2.217568	H	1.593009	1.458345	–6.504232
H	–0.036556	–0.088797	2.665255	H	1.111574	0.159261	–5.378442
C	–5.581904	1.769470	0.372907	H	2.336008	–0.156966	–6.641638
H	–4.552059	1.739940	0.037681	C	2.421233	–0.986430	0.090558
C	0.768456	5.240338	–0.090371	H	2.478771	–1.960269	–0.374530
H	1.239080	5.856109	0.677516	C	–5.103590	3.685795	1.927941
H	0.151776	5.892320	–0.714381	H	–4.989457	3.491750	2.999547
C	3.994020	3.441615	–1.104687	H	–5.501972	4.698792	1.818179
C	–5.203772	–2.008344	1.082117	H	–4.112892	3.651078	1.469870
H	–6.077521	–1.582143	1.584482	C	0.005272	4.164301	–4.099524
H	–4.888678	–2.891427	1.646711	H	0.688131	4.560119	–4.860691
C	3.777109	0.311853	3.273195	H	–0.999589	4.546142	–4.282899
C	3.517182	0.208342	–4.166499	H	–0.004993	3.068906	–4.162155
C	3.723814	3.077451	–2.434323	C	–2.508778	1.765298	–1.888774
C	3.508605	–3.494496	–3.189014	H	–2.946432	2.725106	–2.185050
H	3.164494	–3.786786	–4.185280	H	–3.093659	0.952658	–2.319436
H	4.419714	–4.057135	–2.968983	H	–1.481468	1.702455	–2.261783
C	–5.903175	–0.080209	–1.243681	C	–0.552191	–2.634333	–3.882770
H	–6.252476	0.331480	–2.195932	H	–0.555034	–2.246452	–4.901849
H	–4.817945	–0.070586	–1.195600	H	–0.944303	–1.866822	–3.205451
C	–7.802599	–1.468749	–0.944084	H	–1.190041	–3.524426	–3.827616
C	3.959579	–0.928086	3.934272	C	0.882497	–0.082695	–1.701603
C	–5.563404	–2.390838	–0.357541	H	0.921280	0.842773	–2.282246
H	–4.650211	–2.542830	–0.939878	H	–0.163895	–0.277906	–1.439888
H	–6.105796	–3.337801	–0.346901	H	1.242190	–0.894350	–2.330080
C	–8.228358	1.654309	1.091075				

Table S11. Cartesian coordinates of the DFT energy-minimized structure of $\mathbf{1}_{out\text{-}out}$. CPCM model with MeOH.

Atom	x	y	z	Atom	x	y	z
O	–2.518209	–1.248014	1.731173	H	–8.322640	–3.485192	–0.954717
O	1.017608	–5.060636	0.454774	C	–1.834986	–0.399121	–0.374652
O	5.732798	0.477964	3.714085	C	–7.047264	–2.394364	0.388413
O	–0.209153	2.826038	–1.240015	C	7.157549	–2.228660	–0.211028
O	5.781567	–3.990820	1.461730	H	7.815326	–2.044450	–1.063688
O	0.823493	1.846172	3.660727	H	7.713091	–2.824620	0.517140
O	–0.938635	–1.711047	–3.511535	C	0.037083	7.306836	–0.036532
O	4.333285	4.221654	0.082419	H	0.857983	7.977653	0.195190
O	6.884118	0.117850	–1.701113	C	0.438272	–5.033053	1.753603
O	3.741339	–3.346356	–3.655394	H	–0.603623	–5.378073	1.740175
N	–7.034760	–1.367241	1.285550	H	0.476257	–4.028178	2.192249
N	–5.061069	–2.232640	2.317475	H	1.032155	–5.714979	2.363403
N	–3.084924	3.922518	–2.081987	C	–3.826866	–4.101036	1.271812
N	–1.066755	5.059162	–2.777804	H	–3.086685	–4.117398	2.059603
C	3.981018	2.984245	2.065393	C	0.034752	4.169503	–3.181585
C	–1.113410	–2.592934	–1.326745	H	0.966015	4.599023	–2.811142
C	–0.626049	–3.419125	–0.309208	H	0.093283	4.144488	–4.273643
H	–1.131337	–3.388632	0.647255	C	0.054080	6.597720	–1.231408
C	–0.860728	1.959128	0.856881	H	0.867025	6.728439	–1.937191
C	–1.446396	0.942537	1.612383	C	–9.372736	–1.868229	–0.023617
H	–1.484272	1.069028	2.686972	H	–10.300641	–2.080598	–0.544930
C	1.181432	3.146835	1.728487	C	–9.327936	–0.811489	0.908021
C	3.435432	3.669775	0.967675	C	–1.045909	7.194558	0.849309
C	1.133312	–4.251589	–1.759698	H	–1.067457	7.780558	1.761343
C	–1.940248	–0.221106	1.012515	C	–2.398510	4.711042	–3.162663
C	6.063566	0.361440	2.383298	H	–2.402224	4.118590	–4.075431
C	4.128733	–4.564170	–0.247307	H	–3.002530	5.612054	–3.303520
H	3.590587	–5.230856	0.413952	C	–4.344597	2.589681	–0.062516
C	5.963558	1.530420	1.611173	H	–4.835039	2.066672	0.751280
C	1.727128	2.438878	2.812354	C	–0.127881	2.746134	–2.658472
C	0.487799	–4.238502	–0.510578	H	–1.037383	2.300798	–3.077433
C	–0.448842	–2.580275	–2.562789	H	0.719962	2.121851	–2.968658
C	–0.319190	3.209467	1.524084	C	5.732790	–0.701527	4.510756
H	–0.818579	3.340498	2.487779	H	5.398611	–0.394694	5.502685
H	–0.551536	4.080612	0.911254	H	6.735563	–1.139980	4.587067
C	5.437875	–2.826966	–1.971340	H	5.042932	–1.456408	4.112669
H	5.954112	–2.130012	–2.619351	C	–3.613597	3.733042	0.210154
C	–2.964767	4.425422	–0.820830	H	–3.504145	4.078827	1.228875
C	–2.289804	–1.673178	–1.060288	C	–4.588906	–4.942442	–0.878140
H	–3.020430	–2.188717	–0.437769	H	–4.451743	–5.602142	–1.727189
H	–2.774257	–1.420749	–2.006184	C	5.064674	–4.770895	2.411882
C	2.047730	3.758883	0.819786	H	4.041438	–4.398552	2.548182
H	1.608697	4.287301	–0.015905	H	5.610747	–4.677412	3.351513
C	6.243118	1.458013	0.242891	H	5.023723	–5.828188	2.121712

Atom	x	y	z	Atom	x	y	z
H	6.126354	2.362282	–0.341835	C	–4.431398	2.080684	–1.370981
C	6.477619	–0.834594	1.789187	C	–3.692215	–4.971689	0.199057
H	6.557150	–1.746294	2.367101	H	–2.853548	–5.659878	0.184831
C	–0.760060	1.780222	–0.534728	C	–2.085770	6.325517	0.558498
C	6.765136	–0.906121	0.423179	H	–2.914244	6.225815	1.251230
C	0.653504	–3.415483	–2.771137	C	–5.656410	–4.061781	–0.841659
H	1.184432	–3.411769	–3.714017	H	–6.358966	–4.038238	–1.667054
C	–5.833379	–3.184272	0.246555	C	1.325929	1.029081	4.712244
C	3.630131	–4.390304	–1.541471	H	0.451372	0.616479	5.217193
C	3.113303	2.373874	2.977157	H	1.917296	1.610283	5.430670
H	3.554304	1.824410	3.799605	H	1.942218	0.208204	4.324618
C	4.289914	–3.498027	–2.403645	C	6.668472	1.244267	–2.543615
C	–1.226101	0.608296	–1.130425	H	5.629653	1.593973	–2.490505
H	–1.117732	0.434927	–2.193836	H	6.883925	0.907064	–3.558343
C	–8.123946	–0.599155	1.548880	H	7.337773	2.075644	–2.289464
H	–7.984297	0.187859	2.278987	C	–3.775042	2.789853	–2.360696
C	2.367140	–5.106558	–1.985667	H	–3.793083	2.487691	–3.400333
H	2.441323	–5.354661	–3.047426	C	–10.531278	0.042583	1.196002
H	2.271085	–6.042309	–1.429342	H	–10.873519	0.539884	0.283071
C	5.262491	–3.874498	0.193223	H	–10.313295	0.807265	1.943996
C	–3.028968	–0.959016	3.029619	H	–11.357458	–0.574129	1.563210
H	–3.489807	0.037003	3.034977	C	4.306645	–2.370016	–4.522793
H	–2.232677	–0.960557	3.784856	H	3.688671	–2.372055	–5.421722
C	–2.066050	5.560210	–0.624490	H	5.340393	–2.617491	–4.794589
C	6.625361	0.254311	–0.357237	H	4.286547	–1.370211	–4.070981
C	5.939933	–3.005697	–0.678704	C	–0.237237	–1.599488	–4.745538
C	5.483054	2.833092	2.225176	H	–0.760023	–0.833853	–5.320172
H	5.738350	2.854093	3.287028	H	–0.249242	–2.542754	–5.305365
H	5.990324	3.670875	1.741980	H	0.803694	–1.289407	–4.589927
C	–5.731531	–1.027327	1.896461	C	3.820902	4.840901	–1.091199
H	–5.156341	–0.474635	1.139512	H	3.193799	5.709193	–0.852723
H	–5.912925	–0.383804	2.755266	H	3.238554	4.133290	–1.695722
C	–4.872830	–3.161984	1.298907	H	4.688971	5.171637	–1.662581
C	–1.008737	5.741792	–1.552364	C	–5.180405	0.815168	–1.683445
C	–4.072085	–2.027100	3.378880	H	–5.254314	0.644131	–2.759137
H	–3.598798	–2.980211	3.606944	H	–6.191770	0.849900	–1.267943
H	–4.597478	–1.711098	4.286036	H	–4.665368	–0.043608	–1.241645
C	–8.260272	–2.651058	–0.269279				

Table S12. Cartesian coordinates of the DFT energy-minimized structure of $\mathbf{1}_{in\text{-}out}$.

Atom	x	y	z	Atom	x	y	z
O	–4.216402	–1.049861	0.746333	H	–9.440241	1.641602	1.190796
O	0.714097	4.615958	2.225166	C	–2.144081	–0.404620	1.737008
O	5.300679	–2.187615	–0.547662	C	–7.944111	0.709223	–0.037953
O	0.937820	–2.355591	2.312490	C	5.011122	2.197979	–2.814840
O	5.090405	3.268057	–0.221075	H	5.925292	2.419672	–2.262144
O	2.153150	–5.185495	1.603265	H	5.163197	2.515659	–3.849499
O	–2.742258	1.453570	–0.729236	C	4.123145	0.750633	6.074090
O	1.433052	–3.123641	–3.491239	H	4.370615	0.803803	7.129377
O	3.748655	0.850640	–4.910304	C	0.770523	4.509335	3.644706
O	0.445211	4.141164	–3.119427	H	1.080725	3.504800	3.963516
N	–6.624849	0.770085	–0.384495	H	–0.193502	4.754579	4.105559
N	–6.661205	–1.545232	–1.079671	H	1.518031	5.234028	3.969032
N	3.031472	–0.690230	1.355153	C	–8.797029	–2.749896	–1.212798
N	3.551536	–1.810715	3.444033	H	–8.281780	–3.653345	–1.520991
C	2.877891	–3.924472	–1.812379	C	2.601566	–2.813755	3.940231
C	–1.879092	2.091291	1.385990	H	2.780697	–2.973006	5.006551
C	–1.018433	2.895547	2.140209	H	2.798516	–3.753835	3.423328
H	–0.949948	2.710885	3.205943	C	3.957190	–0.490290	5.472595
C	–1.086140	–2.995848	1.218012	H	4.088229	–1.399589	6.049036
C	–2.375048	–2.644116	0.807951	C	–7.557663	2.659036	1.344760
H	–2.970347	–3.376821	0.271816	H	–7.938647	3.404531	2.036703
C	0.696645	–4.269888	–0.032850	C	–6.199060	2.689074	0.971917
C	1.566910	–3.613880	–2.216588	C	4.010228	1.929728	5.322123
C	–0.357507	4.161040	0.172344	H	4.175269	2.893242	5.792021
C	–2.915517	–1.382754	1.094679	C	3.841163	–1.747467	2.045001
C	4.949116	–1.456815	–1.678444	H	3.636868	–2.693706	1.553626
C	2.889501	4.308332	–0.422558	H	4.884256	–1.466981	1.877945
H	2.940577	4.732084	0.573346	C	1.652958	1.299652	0.117756
C	4.336824	–2.109799	–2.753066	H	1.120279	2.102643	–0.371464
C	1.991128	–4.659791	0.343415	C	1.140803	–2.443309	3.727796
C	–0.234289	3.892742	1.546419	H	0.490011	–3.215931	4.158650
C	–1.941066	2.315497	0.001724	H	0.908447	–1.484776	4.211445
C	–0.481908	–4.356221	0.923833	C	6.702878	–2.476490	–0.451601
H	–1.253556	–5.006820	0.497584	H	7.297485	–1.556361	–0.414529
H	–0.148356	–4.821301	1.853618	H	7.038988	–3.081433	–1.302070
C	2.695920	3.206931	–2.959089	H	6.845739	–3.038872	0.473890
H	2.649617	2.776199	–3.950978	C	2.232670	1.527544	1.354959
C	2.941006	0.511017	1.997064	H	2.122574	2.494279	1.825522
C	–2.676624	0.983047	2.045271	C	–10.863375	–1.541647	–0.805301
H	–3.724387	1.033049	1.747911	H	–11.947247	–1.523840	–0.784273
H	–2.652171	1.137394	3.129730	C	5.381949	4.056243	0.924220
C	0.504863	–3.763114	–1.322617	H	5.359097	5.127860	0.692698
H	–0.498686	–3.471089	–1.610582	H	6.386479	3.773093	1.240988
C	3.937708	–1.335243	–3.850041	H	4.681991	3.855673	1.746479

Atom	x	y	z	Atom	x	y	z
H	3.443958	–1.834833	–4.672763	C	1.733850	0.048050	–0.501121
C	5.159612	–0.076237	–1.701845	C	–10.184125	–2.724484	–1.135500
H	5.626789	0.404896	–0.847164	H	–10.747075	–3.623162	–1.365751
C	–0.338770	–2.026658	1.903710	C	3.693925	1.857914	3.974494
C	4.770158	0.699515	–2.791363	H	3.600501	2.770847	3.396595
C	–1.225073	3.361713	–0.579788	C	–10.144708	–0.395177	–0.510690
H	–1.277134	3.552161	–1.643536	H	–10.672977	0.518797	–0.260770
C	–8.734835	–0.405450	–0.548456	C	3.301331	–5.999727	1.842600
C	1.715181	4.503804	–1.154789	H	4.218544	–5.400620	1.917433
C	3.061979	–4.466372	–0.539166	H	3.427508	–6.751935	1.056114
H	4.071617	–4.709651	–0.232691	H	3.125368	–6.495957	2.797987
C	1.626663	3.940135	–2.439859	C	3.239165	0.240606	–6.090388
C	–0.845714	–0.750313	2.121872	H	3.063228	1.053094	–6.796172
H	–0.219279	–0.009392	2.609769	H	2.293788	–0.284017	–5.897121
C	–5.770342	1.706259	0.097678	H	3.959419	–0.464316	–6.521902
H	–4.742879	1.632481	–0.243814	C	2.441636	–0.938487	0.165613
C	0.517477	5.192824	–0.522298	H	2.572084	–1.935745	–0.227927
H	0.854329	5.939136	0.198819	C	–5.250348	3.733022	1.497114
H	–0.064984	5.703215	–1.292872	H	–5.177171	3.670779	2.587945
C	3.949406	3.548085	–0.931212	H	–5.607868	4.738124	1.251836
C	–5.240696	–2.034377	0.948375	H	–4.246540	3.614297	1.084414
H	–6.034603	–1.562968	1.539774	C	0.393161	3.776542	–4.493038
H	–4.851141	–2.882472	1.520404	H	1.182923	4.270716	–5.070844
C	3.493511	0.611529	3.348853	H	–0.580382	4.108421	–4.856847
C	4.141275	0.046858	–3.872512	H	0.479981	2.689586	–4.629366
C	3.865136	2.998818	–2.220985	C	–2.573904	1.446108	–2.144542
C	4.058608	–3.604859	–2.709662	H	–2.961509	2.360999	–2.609011
H	3.858946	–3.961255	–3.723777	H	–3.138689	0.588211	–2.515128
H	4.939550	–4.136798	–2.340895	H	–1.519195	1.326840	–2.416358
C	–6.155154	–0.247345	–1.386492	C	0.125847	–2.983762	–4.028935
H	–6.537854	0.095200	–2.355261	H	0.258686	–2.701092	–5.074139
H	–5.068313	–0.243310	–1.370282	H	–0.442071	–2.197037	–3.514500
C	–8.059733	–1.588443	–0.947896	H	–0.433050	–3.925957	–3.975598
C	3.670094	–0.575702	4.102341	C	1.067707	–0.205635	–1.824238
C	–5.803131	–2.518098	–0.390750	H	1.017864	0.724003	–2.397502
H	–4.973924	–2.794554	–1.050656	H	0.047342	–0.573071	–1.661366
H	–6.400626	–3.415851	–0.219954	H	1.608360	–0.943052	–2.416648
C	–8.410548	1.682040	0.860149				

Table S13. Cartesian coordinates of the DFT energy-minimized structure of $\mathbf{1}_{out\text{-}out}$.

Atom	x	y	z	Atom	x	y	z
O	3.690080	–0.531302	0.715167	H	10.736172	–1.478383	–1.017031
O	2.821664	4.293540	–1.056600	C	2.186942	–1.222867	–0.987832
O	–2.733110	3.113147	4.224369	C	8.903849	–1.750020	0.074385
O	–1.092454	–2.963926	–0.749904	C	–3.532822	6.003256	0.252955
O	–0.930086	6.686691	0.997289	H	–4.395537	6.260893	–0.364492
O	–0.362423	–1.198397	4.315585	H	–3.315798	6.856554	0.898805
O	1.300358	–0.047386	–4.155081	C	–5.523858	–6.412684	–4.297286
O	–4.874615	–0.359559	1.211867	H	–5.517174	–6.756314	–5.326500
O	–5.468423	4.131914	–0.491249	C	3.646719	4.098908	0.074707
O	–1.503163	4.101026	–3.876638	H	4.634470	3.701512	–0.202399
N	8.375911	–2.346849	1.185334	H	3.182619	3.422743	0.806597
N	6.563890	–0.802740	1.189910	H	3.778214	5.082264	0.529042
N	–4.355024	–4.741737	0.314758	C	6.219386	0.739694	–0.696495
N	–3.200299	–4.871753	–1.826812	H	5.351984	1.173340	–0.224291
C	–3.601295	0.178716	3.123288	C	–2.905544	–3.480648	–2.233359
C	2.564661	0.825176	–2.361410	H	–3.524222	–2.762318	–1.676793
C	2.929593	1.905648	–1.550893	H	–3.192588	–3.398413	–3.284157
H	3.538718	1.701183	–0.682241	C	–4.372927	–5.850678	–3.751322
C	0.398794	–2.253313	0.948730	H	–3.463255	–5.758804	–4.335682
C	1.598139	–1.645948	1.329643	C	11.023718	–2.762164	0.672721
H	1.794171	–1.550062	2.391392	H	12.078610	–2.915451	0.465126
C	–1.618400	–1.709010	2.385718	C	10.451021	–3.359139	1.818728
C	–3.719003	–0.558353	1.933363	C	–6.677533	–6.568763	–3.517035
C	1.617718	3.439641	–2.897680	H	–7.560052	–7.036592	–3.939391
C	2.488690	–1.141734	0.378921	C	–3.121350	–5.196384	–0.437109
C	–3.408073	3.417954	3.063802	H	–2.259811	–4.716846	0.019655
C	0.050421	5.809167	–1.064322	H	–3.074346	–6.282013	–0.313103
H	1.054985	6.082426	–0.770656	C	–6.601846	–3.867584	1.598889
C	–4.393521	2.500473	2.667896	H	–7.499243	–3.504384	2.090720
C	–1.491430	–0.964955	3.569232	C	–1.446531	–3.068505	–2.126137
C	2.476055	3.198736	–1.810732	H	–0.797902	–3.792522	–2.636759
C	1.683376	1.058803	–3.425771	H	–1.339887	–2.094260	–2.621247
C	–0.573426	–2.744238	2.001848	C	–1.777134	4.047324	4.701491
H	–0.018919	–3.035048	2.895642	H	–1.389008	3.634375	5.634657
H	–1.068973	–3.647926	1.630679	H	–2.231193	5.025458	4.904008
C	–2.501363	5.065579	–1.859004	H	–0.949965	4.180270	3.991323
H	–3.507263	4.777936	–2.139982	C	–6.701148	–4.472586	0.354713
C	–5.555458	–4.932500	–0.307586	H	–7.662771	–4.568238	–0.132968
C	3.060574	–0.575200	–2.042306	C	7.674813	0.552801	–2.633046
H	4.093101	–0.529199	–1.692213	H	7.940220	0.863593	–3.636984
H	3.043690	–1.180483	–2.951271	C	0.339746	7.179346	1.389510
C	–2.726593	–1.465120	1.563752	H	1.074463	6.368993	1.500112
H	–2.785515	–1.994739	0.623050	H	0.191977	7.663352	2.356361
C	–5.100583	2.742032	1.487390	H	0.729303	7.914974	0.674123

Atom	x	y	z	Atom	x	y	z
H	–5.847204	2.018848	1.187181	C	–5.354327	–3.697517	2.225812
C	–3.159992	4.555262	2.291559	C	6.594943	1.153672	–1.966379
H	–2.397702	5.271490	2.572333	H	6.010719	1.927023	–2.455177
C	0.109143	–2.354577	–0.419697	C	–6.690214	–6.132335	–2.199554
C	–3.863877	4.795237	1.108790	H	–7.582264	–6.268993	–1.597322
C	1.227996	2.356610	–3.686637	C	8.397753	–0.440306	–1.997007
H	0.532122	2.552677	–4.492032	H	9.234589	–0.904066	–2.508288
C	8.045718	–0.871855	–0.699409	C	–0.212481	–0.495756	5.543493
C	–0.114023	5.155020	–2.287331	H	0.724751	–0.846239	5.978289
C	–2.478969	–0.035597	3.923561	H	–1.033816	–0.713195	6.237448
H	–2.377259	0.571615	4.813082	H	–0.156535	0.588481	5.383664
C	–1.406645	4.767927	–2.674608	C	–6.542447	3.296590	–0.881224
C	0.995128	–1.846368	–1.368367	H	–6.209293	2.264940	–1.069395
H	0.777581	–1.886387	–2.428253	H	–6.935087	3.718208	–1.807953
C	9.111004	–3.115464	2.035178	H	–7.340160	3.278214	–0.127183
H	8.575730	–3.531113	2.880656	C	–4.247077	–4.159273	1.533620
C	1.083618	4.834603	–3.163457	H	–3.245887	–4.066204	1.930309
H	0.798105	4.914411	–4.215077	C	11.261159	–4.214157	2.754254
H	1.872969	5.566800	–2.974645	H	11.689928	–5.068777	2.221294
C	–1.040264	6.080791	–0.234911	H	10.659377	–4.596652	3.581455
C	4.184778	–0.718582	2.026817	H	12.093193	–3.641030	3.175700
H	4.170586	–1.787456	2.292155	C	–2.798958	3.807243	–4.370724
H	3.573352	–0.188260	2.771698	H	–2.653594	3.337631	–5.345571
C	–5.543604	–5.537076	–1.640053	H	–3.402316	4.715383	–4.496077
C	–4.834388	3.866230	0.702028	H	–3.338958	3.113054	–3.711698
C	–2.336601	5.727345	–0.639114	C	0.515879	0.163636	–5.320967
C	–4.629662	1.237385	3.475951	H	0.382374	–0.818630	–5.777837
H	–4.565087	1.461591	4.543502	H	1.020753	0.826396	–6.034525
H	–5.636076	0.860718	3.272072	H	–0.467301	0.588242	–5.077886
C	6.910347	–2.175973	1.392970	C	–4.910451	–0.785449	–0.136050
H	6.413612	–2.849933	0.678274	H	–4.971204	–1.880795	–0.215744
H	6.667761	–2.483624	2.409991	H	–4.035627	–0.431531	–0.696429
C	6.905310	–0.303731	–0.060694	H	–5.814614	–0.355539	–0.571111
C	–4.369540	–5.430735	–2.420455	C	–5.214907	–3.024118	3.562002
C	5.609635	–0.169078	2.097185	H	–4.167758	–2.899178	3.842945
H	5.611663	0.903111	1.893978	H	–5.726986	–3.598605	4.340617
H	5.974106	–0.295331	3.124200	H	–5.666188	–2.028877	3.521900
C	10.272366	–1.968915	–0.171348				

Table S14. Cartesian coordinates of the DFT energy-minimized structure of $\mathbf{2}_{in}$.

Atom	x	y	z	Atom	x	y	z
C	–3.660039	–2.283445	0.192793	H	–4.074434	–2.427534	–0.796083
C	–4.301939	–1.363005	1.032038	H	–2.283030	–1.784206	3.754070
C	–3.779793	–1.165214	2.320612	H	–1.044280	–3.856582	3.479609
C	–2.678622	–1.907247	2.754940	H	–0.856974	–4.592788	1.889648
C	–2.025776	–2.807728	1.911128	H	–4.222053	–1.083970	4.971278
C	–2.502907	–2.957161	0.597472	H	–3.008575	0.179444	4.617792
O	–1.735976	–3.760934	–0.217442	H	–4.711986	0.631083	4.897609
C	–0.863244	–3.639641	2.422933	H	–6.063871	–0.153626	1.315385
C	0.513845	–3.011976	2.297619	H	–6.021554	–1.022595	–0.226762
O	–4.386575	–0.195792	3.086489	H	2.699228	–1.011267	3.995841
C	–4.052947	–0.124571	4.468110	H	0.976239	–4.005578	0.472678
C	–5.416325	–0.473555	0.496862	H	4.946976	–1.272807	3.137632
C	–4.774362	0.735874	–0.167048	H	5.251877	–2.196685	1.659060
C	0.995038	–2.134136	3.282891	H	3.495198	3.048376	1.499797
C	2.312940	–1.672116	3.232384	H	5.209378	–1.126022	–0.491130
C	3.173331	–2.030141	2.189991	H	3.989183	4.194797	–0.717776
C	2.673598	–2.859620	1.171690	H	4.067363	3.172853	–2.154936
C	1.363666	–3.349423	1.242274	H	–2.420377	4.768978	–1.454443
C	4.586550	–1.466211	2.124388	H	–2.323883	3.827061	–2.943140
C	4.588477	–0.182328	1.314718	H	–3.323708	1.692689	–3.106921
C	4.138933	1.022623	1.886057	H	–4.591695	1.817181	1.659557
C	3.899416	2.135682	1.082793	H	3.030317	–5.130151	–0.205007
C	4.096635	2.093427	–0.301377	H	2.286311	–3.959239	–1.337096
C	4.631963	0.924762	–0.859688	H	4.026218	–4.301402	–1.435852
C	4.876465	–0.193827	–0.051848	H	0.910767	–0.009918	5.017335
C	3.634789	3.258462	–1.156756	H	1.409065	–1.487087	5.888394
C	2.117097	3.311928	–1.271204	H	–0.262724	–0.883956	6.039735
C	1.454001	2.615801	–2.285120	H	5.888583	0.266089	–3.809167
C	0.065619	2.700272	–2.445020	H	6.713678	0.067137	–2.234882
C	–0.696965	3.517951	–1.594458	H	5.404755	–1.048402	–2.708090
C	–0.041684	4.162018	–0.540280	H	4.229656	2.998943	3.732408
C	1.341787	4.057889	–0.365626	H	3.386491	1.977318	4.928030
C	–2.171972	3.785179	–1.861284	H	2.515695	2.565445	3.483760
C	–4.437719	0.720355	–1.529048	H	–3.846152	–0.273593	–4.097548
C	–3.624940	1.724286	–2.067075	H	–5.398484	–1.147795	–4.044566
C	–3.123345	2.759860	–1.278121	H	–5.379092	0.638000	–4.003098
C	–3.512771	2.813197	0.072832	H	–3.345776	3.326393	2.806951
C	–4.334391	1.817457	0.608489	H	–3.145637	5.055978	2.425802
O	3.528776	–3.131161	0.131167	H	–4.672244	4.225080	2.015923
O	0.108366	–1.795051	4.279798	H	–3.215478	–4.717558	–1.333791
C	3.183392	–4.191949	–0.752529	H	–1.513898	–5.063884	–1.699138
C	0.580886	–1.001647	5.357635	H	–2.184480	–3.783568	–3.555038
O	4.799730	0.903247	–2.229965	H	–3.227601	–2.727511	–2.640778
O	3.881514	0.998083	3.236936	H	–0.106842	–4.068875	–4.174310

Atom	x	y	z	Atom	x	y	z
C	5.755583	–0.009642	–2.761892	H	2.246621	–4.037099	–4.883767
C	3.482246	2.207475	3.865849	H	3.804349	–2.291979	–4.039215
O	–4.848384	–0.381438	–2.257467	H	3.025130	–0.674475	–2.331254
O	–3.007134	3.856318	0.808620	H	2.474452	–0.388558	–0.161126
C	–4.861387	–0.271541	–3.678464	H	1.548511	0.773635	1.788341
C	–3.583303	4.116963	2.084106	H	–2.370012	0.257924	0.109837
C	–2.219837	–4.267743	–1.450006	H	–1.062476	2.461062	2.102956
C	–2.263389	–3.236837	–2.608646	H	–2.143461	1.102132	2.439321
N	–1.244511	–2.202021	–2.534725	H	–0.544372	1.164971	3.206290
C	0.090636	–2.357191	–2.864920	H	–1.440569	–0.132830	–2.884306
C	0.568875	–3.329686	–3.759710	H	–2.639755	–0.863885	–1.786070
C	1.899113	–3.303311	–4.163073	H	2.053545	2.019203	–2.963305
C	2.781732	–2.326715	–3.680705	H	–0.641510	4.771354	0.123425
C	2.345263	–1.419072	–2.726207	O	–0.624758	2.031724	–3.437140
C	1.013153	–1.443556	–2.276941	O	2.025946	4.683369	0.649775
C	0.579901	–0.663630	–1.118127	C	0.123214	1.522956	–4.540255
C	1.415074	–0.202326	–0.101043	H	–0.612472	1.180500	–5.270308
C	0.882064	0.446862	1.001507	H	0.747531	2.302876	–4.991271
C	–0.499354	0.647119	1.124322	H	0.756027	0.676522	–4.246069
C	–1.296204	0.163678	0.104562	C	1.308718	5.551264	1.514934
N	–0.757287	–0.460588	–0.966003	H	0.537586	5.011736	2.082303
C	–1.099538	1.381885	2.289488	H	0.835362	6.372987	0.963517
C	–1.606336	–0.884324	–2.103545	H	2.044457	5.960729	2.208475

Table S15. Cartesian coordinates of the DFT energy-minimized structure of $\mathbf{2}_{out}$.

Atom	x	y	z	Atom	x	y	z
O	1.929420732	–3.295676	3.691396433	H	–1.314927	4.883880	–0.032659
O	5.863262354	–2.768166	0.726320288	C	–6.430937	–1.822465	–1.479823
O	1.910227988	4.478121	1.939421805	C	0.448832	–3.797813	1.931801
O	–2.715142876	–2.141102	0.878891063	C	4.432647	3.408356	–2.214920
O	5.942644703	2.237391	–0.179321337	H	4.361961	3.731139	–3.256012
O	–0.834277418	1.453574	4.570535394	H	5.262202	3.947772	–1.752312
O	0.977972896	–4.470886	–1.279217272	C	–7.885639	–2.541775	–3.265395
O	–1.957143749	2.568837	–0.740343955	H	–8.216358	–3.315125	–3.951018
O	1.966789877	3.508695	–3.520547163	C	6.165405	–2.954486	2.098255
O	3.849514932	–1.203601	–3.995279355	H	6.179835	–4.017619	2.372082
N	–5.721347928	0.138730	0.417558538	H	5.449194	–2.430966	2.746935
N	–5.386274031	–2.071161	–0.54686719	H	7.160907	–2.533417	2.249002
C	–1.016203796	3.235816	1.317285825	C	–4.024749	–2.080525	–1.126304
C	2.468404922	–4.264296	0.54443389	H	–3.661719	–1.056842	–1.299325
C	3.692745808	–3.840426	1.067322499	H	–4.108498	–2.559071	–2.104863
H	3.861385314	–3.979295	2.126987386	C	–6.842664	–2.805183	–2.382504
C	–1.273867963	–1.697997	2.715656935	H	–6.359416	–3.776434	–2.359834
C	–0.103579322	–1.969299	3.431812057	C	–8.533540	–1.298811	–3.249938
H	0.121517829	–1.340524	4.284968814	H	–9.367058	–1.112000	–3.918211
C	–1.762869339	0.775972	2.511302741	C	–5.592067	–1.349339	0.669298
C	–1.666833645	2.242834	0.565803759	H	–4.757850	–1.501402	1.349031
C	4.410916064	–3.051331	–1.11256996	H	–6.534541	–1.659623	1.129398
C	0.760301609	–2.995912	3.044925678	C	–5.941540	2.809351	–0.109192
C	1.969411214	4.252485	0.582658339	H	–6.018941	3.867750	–0.339162
C	5.712525644	–0.017947	–1.100075383	C	–2.986797	–2.847785	–0.324500
H	6.298017573	–0.468990	–0.308968413	H	–3.337352	–3.866062	–0.108181
C	0.744926566	4.293973	–0.10130612	H	–2.079335	–2.917606	–0.939171
C	–1.104333418	1.762836	3.261925413	C	3.121661	4.422024	2.676024
C	4.653666376	–3.229076	0.258714138	H	2.852701	4.613102	3.716840
C	2.215129841	–4.061168	–0.820620095	H	3.835328	5.187294	2.344943
C	–2.162711896	–0.547385	3.141154135	H	3.597479	3.435465	2.598677
H	–2.128844465	–0.449538	4.227142217	C	–6.564387	1.882714	–0.933243
H	–3.200149509	–0.786090	2.880768495	H	–7.108084	2.207587	–1.811031
C	4.169142781	1.065163	–3.134289458	C	6.770293	1.723761	0.850571
H	3.558085047	1.511907	–3.908446932	H	6.240838	0.985243	1.468255
C	–6.466925911	0.512165	–0.664262497	H	7.048621	2.578154	1.470338
C	1.410337404	–4.867431	1.450953935	H	7.680418	1.259975	0.448376
H	1.892749249	–5.342097	2.308531899	C	–5.191115	2.399449	1.006137
H	0.854728577	–5.636638	0.908558679	C	–8.114564	–0.307683	–2.373727
C	–2.012159186	1.026741	1.155238889	H	–8.628103	0.647999	–2.358002
H	–2.46566788	0.234661	0.574202542	C	–0.128902	2.404506	5.357303
C	0.737259588	4.057460	–1.478253928	H	–0.010051	1.950174	6.341770
H	–0.217037627	4.072402	–1.988276525	H	–0.689113	3.342849	5.458697
C	3.152022218	3.991234	–0.11337074	H	0.860330	2.622263	4.934731
H	4.104772375	3.935878	0.397624034	C	0.759765	3.554839	–4.256473

Atom	x	y	z	Atom	x	y	z
C	–1.566740157	–2.482430	1.595765913	H	0.036923	2.805624	–3.901230
C	3.145395041	3.752111	–1.489404814	H	1.025663	3.332020	–5.291350
C	3.184651819	–3.470194	–1.634090244	H	0.290329	4.546829	–4.212238
H	3.005140173	–3.292615	–2.68714693	C	–5.105663	1.033484	1.225907
C	5.183917968	–0.867992	–2.074804655	H	–4.539469	0.620951	2.048900
C	–0.744112819	2.976363	2.660415517	C	3.121642	–0.677263	–5.090025
H	–0.204775445	3.731933	3.215430739	H	2.827256	–1.533967	–5.699402
C	4.384965383	–0.314341	–3.086889156	H	3.733201	0.002711	–5.697649
C	–0.725132465	–3.532605	1.22713381	H	2.220258	–0.140563	–4.761454
H	–0.933022578	–4.148954	0.362880527	C	0.729596	–4.385213	–2.671528
C	5.429360433	–2.364011	–2.002424786	H	–0.267552	–4.802721	–2.829149
H	5.377137522	–2.789919	–3.006863396	H	1.456270	–4.967594	–3.252559
H	6.433531072	–2.546348	–1.613134769	H	0.747932	–3.345650	–3.027469
C	5.471109763	1.357671	–1.129540568	C	–2.415514	1.546297	–1.601316
C	2.323118023	–2.483978	4.786924601	H	–3.427507	1.208827	–1.330054
H	1.600512644	–2.535576	5.61172146	H	–1.739582	0.681232	–1.604256
H	2.451059536	–1.435707	4.486434996	H	–2.449130	1.979838	–2.602588
C	–7.042526301	–0.547691	–1.493290657	C	–4.494827	3.377851	1.909689
C	1.917852997	3.777910	–2.170356459	H	–3.821480	2.873700	2.605102
C	4.710813033	1.916923	–2.168278623	H	–5.225685	3.960731	2.480440
C	–0.549193527	4.520560	0.658757514	H	–3.894989	4.073520	1.317370
H	–0.399737922	5.284391	1.426319646	H	3.280559	–2.881335	5.127136

Table S16. Cartesian coordinates of the DFT energy-minimized structure of $\mathbf{2}_{in}$. CPCM model with MeOH.

Atom	x	y	z	Atom	x	y	z
C	–0.527188	–1.323122	3.411535	H	0.019282	–2.233699	3.201902
C	0.158631	–0.309898	4.081212	H	–2.377973	1.968797	4.159766
C	–0.502318	0.908980	4.309698	H	–4.551706	–0.740871	3.164747
C	–1.847548	1.048447	3.957325	H	–4.443670	0.960411	3.632706
C	–2.548762	0.020017	3.315817	H	–0.685820	3.590179	4.084279
C	–1.853533	–1.160880	3.002266	H	–1.208508	3.160870	5.738426
O	–2.532439	–2.095584	2.244119	H	0.420934	3.831400	5.464300
C	–4.022885	0.196590	2.972298	H	1.705543	–1.552523	4.875196
C	–4.235796	0.593965	1.524725	H	1.811114	0.138153	5.367535
O	0.240459	1.902275	4.898952	H	–3.549682	3.126836	–0.658593
C	–0.355322	3.187339	5.049707	H	–4.982364	–1.314150	0.941497
C	1.593989	–0.523440	4.526081	H	–4.531323	0.713619	–3.313397
C	2.596990	–0.259569	3.419127	H	–4.484627	2.444454	–2.973187
C	–3.883876	1.879951	1.072949	H	0.077343	3.676436	–3.373012
C	–3.881579	2.164301	–0.293557	H	–2.438050	–0.573228	–3.498103
C	–4.236449	1.198635	–1.243393	H	1.780770	0.934856	–5.061456
C	–4.687397	–0.050502	–0.789212	H	1.817884	2.656487	–4.672658
C	–4.688697	–0.334488	0.584182	H	5.975659	–0.297227	–0.053867
C	–4.031612	1.481782	–2.721513	H	5.977468	1.423325	0.360322
C	–2.553472	1.518377	–3.082605	H	4.367860	–1.819561	0.950780
C	–1.831040	2.722753	–3.024419	H	2.653429	1.832836	3.803402
C	–0.482344	2.751166	–3.389384	H	–6.083726	–2.613523	–2.274703
C	0.187665	1.594369	–3.794008	H	–5.174908	–2.825236	–0.763198
C	–0.514936	0.376776	–3.796241	H	–6.659655	–1.835096	–0.771699
C	–1.874639	0.352563	–3.459085	H	–2.371879	4.182119	0.968170
C	1.632722	1.661335	–4.258669	H	–4.105185	4.574397	1.150660
C	2.638174	1.385726	–3.157677	H	–3.010677	4.663423	2.560323
C	3.213416	0.124661	–3.006772	H	–1.267257	–1.816672	–5.165177
C	4.105456	–0.150237	–1.963663	H	0.275701	–2.642391	–4.801428
C	4.465346	0.857954	–1.058300	H	–0.906407	–2.374513	–3.506916
C	3.901598	2.130923	–1.220268	H	–1.000258	4.959906	–1.740435
C	2.987026	2.394790	–2.240469	H	–2.543635	5.783808	–2.091174
C	5.336511	0.563609	0.151572	H	–1.409906	5.417880	–3.418834
C	3.068805	–1.300891	2.603049	H	2.457963	–4.518226	2.423188
C	4.003730	–1.030393	1.597608	H	2.733883	–3.498164	0.992889
C	4.451641	0.271589	1.352264	H	4.068109	–3.801280	2.142002
C	3.959577	1.316440	2.155221	H	4.283165	4.576201	2.117928
C	3.048133	1.040998	3.178528	H	2.743706	3.714389	2.388587
O	–5.021724	–0.977289	–1.749424	H	4.033827	3.652712	3.623221
O	–3.502730	2.777832	2.036059	H	–2.496746	–3.963825	3.149764
C	–5.775081	–2.120852	–1.352247	H	–1.019833	–3.556344	2.248964
C	–3.235074	4.122178	1.640805	H	–2.555228	–5.198997	1.081621
O	0.212411	–0.730240	–4.160931	H	–3.721736	–3.899246	0.891163
O	–2.529564	3.837481	–2.622114	H	–1.438137	–6.328595	–0.464208

Atom	x	y	z	Atom	x	y	z
C	–0.474211	–1.948659	–4.419599	H	0.670121	–7.295875	–1.300193
C	–1.817612	5.061336	–2.465401	H	2.539124	–5.814509	–2.024317
O	2.547587	–2.548296	2.850007	H	2.315737	–3.366717	–1.804964
O	4.420765	2.578551	1.867279	H	2.351170	–1.593137	–0.588086
C	2.987174	–3.640911	2.049583	H	2.063008	0.732391	0.125978
C	3.829232	3.681845	2.546176	H	–2.183127	0.096833	0.155011
C	–2.109415	–3.463416	2.253378	H	0.610752	2.455635	1.108011
C	–2.654535	–4.116567	0.990012	H	–0.546024	2.687757	–0.214641
N	–1.969890	–3.691353	–0.237818	H	–1.111860	2.130346	1.372710
C	–0.768776	–4.286250	–0.645698	H	–2.263629	–2.353252	–1.810647
C	–0.614459	–5.678337	–0.736751	H	–3.026926	–1.905795	–0.264265
C	0.572396	–6.218252	–1.216576	H	2.911911	–0.658927	–3.691426
C	1.626826	–5.386850	–1.623453	H	4.168750	2.894314	–0.502294
C	1.498308	–4.011695	–1.506993	O	4.602689	–1.416734	–1.723832
C	0.317613	–3.442434	–0.992614	O	2.384203	3.612895	–2.424128
C	0.248739	–2.022277	–0.650864	C	4.768038	–2.283771	–2.847611
C	1.356058	–1.198489	–0.447040	H	5.312103	–1.781205	–3.655262
C	1.188967	0.114223	–0.032635	H	3.808645	–2.642473	–3.238193
C	–0.087302	0.638227	0.206886	H	5.343659	–3.138185	–2.489070
C	–1.162157	–0.210194	–0.001773	C	2.524011	4.592729	–1.399590
N	–0.983276	–1.482952	–0.419772	H	2.191292	4.203678	–0.429231
C	–0.298095	2.059972	0.648602	H	1.886792	5.427828	–1.693208
C	–2.158428	–2.360346	–0.721250	H	3.559251	4.943208	–1.309625

Table S17. Cartesian coordinates of the DFT energy-minimized structure of **2b**. CPCM model with MeOH.

Atom	x	y	z	Atom	x	y	z
C	–2.507634	0.676539	3.380051	H	–3.779968	0.147317	–3.215796
C	–2.253381	2.016301	3.075289	H	–3.031189	–1.972571	–4.389893
C	–3.071801	2.653327	2.127275	H	–2.455119	–3.309940	–3.395487
C	–4.157332	1.974654	1.566930	H	4.810041	2.279890	1.636025
C	–4.416074	0.633879	1.874182	H	4.400408	3.822012	0.879840
C	–3.554598	–0.023720	2.769776	H	2.876420	3.703631	–1.203743
C	–5.511766	–0.123926	1.136251	H	4.575651	–0.864933	–1.914456
C	–4.924459	–0.739006	–0.123054	H	–0.246426	–3.268412	–2.431448
C	–1.131342	2.775850	3.761156	H	0.634285	1.015229	–4.719168
C	0.195149	2.753842	3.021697	H	3.612596	–0.969554	–4.059221
C	1.777849	3.803425	1.492753	H	2.961457	0.482098	–4.830254
C	2.721088	2.786664	1.728463	H	–1.324497	2.243006	–4.422532
C	1.125021	1.724614	3.249107	H	–1.146371	1.543646	–6.057351
C	–4.416196	–2.038421	–0.111864	H	–2.768690	1.929138	–5.423412
C	–3.657275	–2.539033	–1.177455	H	–5.313194	1.842949	–3.284966
C	–3.404069	–1.737148	–2.299616	H	–3.851114	2.340915	–2.388381
C	–3.972092	–0.456670	–2.340343	H	–5.412949	3.145171	–2.070559
C	–4.724291	0.043289	–1.277589	H	–1.878709	–2.046473	3.483816
C	–2.534479	–2.222565	–3.447179	H	–3.263783	–3.118064	3.815370
C	–1.130958	–1.632051	–3.476860	H	–2.964530	–1.701291	4.859924
C	4.070265	2.788190	1.015490	H	–3.062992	5.613402	0.717311
C	3.988072	2.101905	–0.341546	H	–3.498058	4.107858	–0.134503
C	3.259360	2.708098	–1.379576	H	–4.556610	4.741460	1.158642
C	2.969657	2.031666	–2.563690	H	–3.257421	–5.744513	–0.651884
C	3.416895	0.707506	–2.743623	H	–4.799730	–4.838231	–0.602042
C	4.230556	0.148812	–1.762147	H	–3.599656	–4.646285	0.704145
C	4.523296	0.824069	–0.566800	H	1.789544	–4.471648	–2.958721
C	–0.046924	–2.327340	–2.930877	H	3.206497	–4.229601	–1.895410
C	1.264171	–1.844865	–3.039140	H	1.565101	–3.973306	–1.256408
C	1.519579	–0.638405	–3.713921	H	–1.405677	–3.964233	0.788373
C	0.430799	0.077771	–4.220080	H	–0.825940	–6.114248	1.879841
C	–0.877479	–0.398731	–4.104160	H	1.400628	–6.370959	2.971033
C	2.937780	–0.121575	–3.918867	H	3.052979	–4.558366	2.896417
O	–1.966756	0.262193	–4.618001	H	1.695432	1.005619	–0.279104
C	–1.774938	1.565938	–5.159026	H	–2.529186	0.343331	0.056066
O	–5.273088	1.301386	–1.261561	H	–2.059225	–1.869731	0.988302
C	–4.936302	2.195821	–2.317491	H	0.198410	2.728809	–1.097959
O	–3.789473	–1.364966	2.964759	H	–1.366943	2.149304	–1.700755
C	–2.918037	–2.083986	3.833443	H	–1.244911	2.863001	–0.081893
O	2.228721	2.562614	–3.585674	H	2.658547	–1.802587	–0.403164
C	1.817949	3.924130	–3.492647	H	3.092433	–0.428869	0.639199
O	–2.730419	3.946092	1.804489	C	6.123085	–0.849826	0.207467
C	–3.516264	4.628162	0.831072	H	6.890960	–0.755151	0.981488
O	–3.074001	–3.789404	–1.143890	H	6.600817	–0.675195	–0.763213

Atom	x	y	z	Atom	x	y	z
C	–3.728486	–4.800477	–0.374094	C	5.525370	–2.262649	0.251319
O	2.362372	–2.506317	–2.528877	H	4.699118	–2.353809	–0.458514
C	2.205453	–3.872639	–2.140829	H	6.310298	–2.925468	–0.133718
O	5.202423	0.227813	0.479070	C	5.109726	–2.799104	1.632613
N	2.645149	–2.129729	1.701151	H	5.977492	–2.759427	2.303524
C	1.771221	–3.199278	1.806183	H	4.850970	–3.857926	1.522208
C	0.466897	–3.020300	1.262424	C	3.959168	–2.076257	2.353330
C	–0.448261	–4.087000	1.279762	H	4.211097	–1.019415	2.473758
C	–0.118054	–5.292937	1.880693	H	3.841230	–2.489754	3.358711
C	1.138307	–5.435737	2.486254	O	2.155375	4.802792	0.626810
C	2.077669	–4.410197	2.449889	O	0.724680	0.753659	4.130326
C	0.072261	–1.700542	0.783294	C	1.216829	5.833274	0.329917
N	1.059121	–0.820555	0.462401	H	0.299977	5.426722	–0.114064
C	0.823068	0.414947	–0.040341	H	1.708593	6.485480	–0.392408
C	–0.463927	0.872898	–0.235762	H	0.956999	6.411788	1.224409
C	–1.504471	0.013737	0.148761	C	1.637314	–0.286331	4.466738
C	–1.242377	–1.243820	0.664327	H	1.878314	–0.895145	3.590677
C	–0.730977	2.233384	–0.813850	H	1.127928	–0.904903	5.206512
C	2.456135	–1.296330	0.546254	H	2.561588	0.112503	4.902759
H	–1.843483	0.185633	4.079978	H	–0.195398	4.555309	1.962881
H	–4.797935	2.457317	0.842039	C	0.536637	3.777432	2.137022
H	–6.321795	0.563580	0.881506	H	3.105283	0.980153	2.806818
H	–5.920089	–0.909834	1.775299	C	2.373257	1.755027	2.612381
H	–0.984094	2.352165	4.757615	H	1.295621	4.142514	–4.424413
H	–1.439348	3.817022	3.887005	H	2.678116	4.595807	–3.387541
H	–4.555210	–2.631210	0.783980	H	1.133040	4.086666	–2.652386

Table S18. Cartesian coordinates of the DFT energy-minimized structure of **1b**.

Atom	x	y	z	Atom	x	y	z
C	–3.902397	–3.232503	–1.125073	H	3.994513	–2.974712	–0.334850
C	–2.909262	–3.505858	–2.063720	H	3.396929	–3.273434	1.295421
C	–2.832959	–2.687724	–3.206718	H	2.477147	–0.892484	–1.429062
C	–3.741455	–1.640813	–3.382342	H	3.228455	1.135718	2.987969
C	–4.733662	–1.355333	–2.433066	H	–0.636377	5.195241	–1.653564
C	–4.807412	–2.178245	–1.299196	H	–0.046259	2.569629	2.478911
C	–5.670561	–0.172759	–2.627911	H	3.085103	3.553033	0.725657
C	–5.064545	1.133969	–2.126777	H	2.467588	3.169144	2.318330
C	–1.934613	–4.652065	–1.860828	H	–1.985226	2.134484	3.554777
C	–0.605086	–4.201735	–1.282720	H	–1.857417	3.905640	3.762972
C	1.675353	–3.410804	–1.614855	H	–3.459594	3.116726	3.730628
C	1.877333	–3.331373	–0.227872	H	–7.223497	2.545424	1.176727
C	–0.415021	–4.106696	0.107176	H	–8.603772	1.873565	0.270313
C	–3.894740	1.630639	–2.714399	H	–7.717733	3.282231	–0.375098
C	–3.263475	2.783541	–2.250001	H	–7.169954	–2.617197	0.912854
C	–3.753335	3.448048	–1.109313	H	–5.510928	–3.166294	1.259275
C	–4.942617	2.981756	–0.548862	H	–6.416050	–3.865456	–0.114722
C	–5.618996	1.868434	–1.068185	H	1.691642	0.797098	–3.116994
C	–3.014115	4.637758	–0.521710	H	–0.937651	–2.688456	–5.877832
C	–1.629014	4.284829	–0.008805	H	–2.686062	–2.347079	–5.903089
C	3.168028	–2.775888	0.351627	H	–1.560652	–1.194513	–5.125327
C	3.054523	–1.276783	0.587116	H	–2.674805	3.008872	–4.834437
C	2.611000	–0.450847	–0.451406	H	–1.464490	1.865375	–4.186271
C	2.327030	0.897341	–0.256420	H	–1.012593	3.561071	–4.488622
C	2.556862	1.497007	0.997312	H	2.813122	5.728825	–2.457157
C	3.056609	0.685488	2.016891	H	1.240278	5.033750	–2.927823
C	3.297078	–0.683606	1.833454	H	1.305499	6.457681	–1.848685
C	–0.490657	4.643991	–0.734519	H	0.346897	–0.984811	1.427143
C	0.790506	4.285974	–0.314179	H	1.418198	–1.248797	3.600338
C	0.956878	3.496630	0.835763	H	0.116199	–0.991471	5.715384
C	–0.175452	3.171977	1.585863	H	–2.310653	–0.586098	5.603186
C	–1.453062	3.577170	1.188306	H	–4.316801	–1.258002	5.322014
C	2.320150	2.978381	1.249778	H	–6.756608	–1.162383	5.004309
O	–2.595384	3.288453	1.909743	H	–6.073655	0.149018	0.965578
C	–2.449392	3.098839	3.316617	H	–8.586267	0.395237	3.274686
O	–6.808024	1.407426	–0.536668	H	–8.606759	–1.351554	3.041439
C	–7.622145	2.339668	0.174078	H	–8.348658	–0.279798	1.654438
O	–5.794708	–1.888674	–0.379623	H	5.350806	–0.740968	–3.119813
C	–6.234657	–2.952098	0.461987	H	5.608787	–3.194935	–3.201098
O	1.865617	1.714055	–1.255015	H	6.679220	–4.386768	–1.302706
C	1.047348	1.213463	–2.327728	H	7.529102	–3.151658	0.638144
N	–2.317318	–0.531813	1.042578	H	7.175424	3.351472	0.896734
O	–1.842064	–2.996393	–4.102767	H	3.884672	3.402296	–1.868288
C	–1.765819	–2.258195	–5.313043	H	4.321541	1.036681	–2.343432

Atom	x	y	z	Atom	x	y	z
O	–2.144679	3.318011	–2.837052	H	5.761079	5.394792	0.736254
C	–1.818798	2.904371	–4.157114	H	5.733988	5.692604	–1.005509
O	1.934129	4.654709	–0.990451	H	4.230987	5.298833	–0.164588
C	1.798708	5.515457	–2.114895	H	–4.032715	0.178088	0.127168
O	3.691513	–1.519867	2.859855	H	8.688440	0.840343	–0.235271
C	–1.637709	–0.651185	2.235068	H	8.216426	1.440123	1.382430
C	–0.253114	–0.901768	2.322653	H	–3.297173	1.345287	1.259300
C	0.355027	–1.039993	3.562826	H	0.579014	2.122477	–2.714112
C	–0.377226	–0.910805	4.753315	C	–0.030656	0.189982	–1.954834
C	–1.740965	–0.687310	4.685652	H	0.409999	–0.803444	–1.832950
C	–2.396199	–0.580256	3.441723	H	–0.701306	0.103727	–2.820213
C	–3.845675	–0.541005	3.351035	C	–0.838229	0.536084	–0.701013
C	–4.725498	–0.912623	4.381834	H	–1.490180	1.393486	–0.888113
C	–6.095402	–0.870155	4.193615	H	–0.165511	0.836911	0.103580
C	–6.641016	–0.475982	2.957929	C	–1.654136	–0.683865	–0.255277
C	–5.747672	–0.142217	1.956527	H	–1.018054	–1.569600	–0.197224
N	–4.407396	–0.181589	2.159156	H	–2.428415	–0.912443	–0.988857
C	–8.125532	–0.425042	2.713590	C	4.424963	–0.970005	3.964273
N	7.449166	–0.435889	0.915886	H	4.526250	–1.806633	4.660909
C	6.956175	–1.211004	–0.127577	H	3.840861	–0.191729	4.471256
C	6.299506	–0.528390	–1.190187	C	5.796480	–0.447055	3.539325
C	5.823836	–1.261612	–2.294375	H	5.654113	0.312983	2.761953
C	5.968409	–2.637323	–2.345298	H	6.264455	0.053415	4.395976
C	6.572293	–3.307270	–1.272340	C	6.704955	–1.560933	3.006782
C	7.054345	–2.611291	–0.170744	H	6.111986	–2.237746	2.383480
C	6.012920	0.888472	–1.031650	H	7.097234	–2.160026	3.836573
N	6.746531	1.591926	–0.118658	C	7.886135	–1.024578	2.189949
C	6.522202	2.904709	0.156372	H	8.628321	–1.811109	2.005791
C	5.529909	3.620700	–0.483484	H	8.404563	–0.241242	2.750829
C	4.738805	2.914024	–1.413682	O	2.724977	–3.000995	–2.413426
C	4.976888	1.579213	–1.677992	O	–1.500706	–4.417586	0.885346
C	5.302061	5.082041	–0.204514	C	2.550625	–3.058664	–3.821581
C	–3.502280	0.284187	1.069438	H	1.714851	–2.427557	–4.153102
C	7.885288	0.866934	0.518068	H	2.381268	–4.084632	–4.170078
H	–3.942322	–3.851427	–0.237254	H	3.477388	–2.681903	–4.257856
H	–3.710286	–1.041540	–4.284064	C	–1.321588	–4.514567	2.293710
H	–6.612601	–0.357918	–2.113132	H	–1.069353	–3.544475	2.738261
H	–5.893927	–0.072312	–3.695995	H	–0.543433	–5.243995	2.550219
H	–1.755352	–5.140949	–2.821975	H	–2.277246	–4.856692	2.693753
H	–2.386585	–5.387921	–1.192214	H	0.281813	–3.914890	–3.190619
H	–3.474779	1.090780	–3.552567	C	0.451504	–3.851335	–2.124668
H	–5.338608	3.503678	0.314588	H	0.976789	–3.620049	1.686072
H	–3.610664	5.054032	0.294741	C	0.822194	–3.694430	0.616276
H	–2.916904	5.413237	–1.288411				

Table S19. Cartesian coordinates of the DFT energy-minimized structure of **1b**. CPCM model with MeOH.

Atom	x	y	z	Atom	x	y	z
C	4.854824	1.011573	3.040609	H	–2.609335	2.348018	1.917092
C	3.981311	0.192990	3.758650	H	–3.149331	0.883048	2.732902
C	4.126422	–1.200890	3.651283	H	–2.192489	–1.340469	1.599821
C	5.150426	–1.740765	2.868857	H	–2.670753	1.088190	–2.659224
C	6.008553	–0.921218	2.123971	H	2.300037	–0.142872	–4.233639
C	5.842915	0.470790	2.210337	H	–0.614570	–3.692742	–2.425199
C	6.980868	–1.537749	1.128367	H	–2.430623	–0.909166	–3.900749
C	6.248782	–1.814362	–0.175835	H	–2.554917	–2.484262	–3.098137
C	2.876240	0.788605	4.610173	H	0.749730	–4.946962	–1.037513
C	1.537759	0.883593	3.898025	H	0.461612	–5.712170	–2.627064
C	–0.707454	0.021671	3.495634	H	1.923633	–6.122119	–1.690182
C	–1.016978	1.132662	2.690197	H	3.676298	–4.770478	0.600836
C	1.240196	1.985417	3.078073	H	4.936251	–5.625304	1.531051
C	6.195625	–0.850391	–1.185913	H	4.949262	–5.688602	–0.251571
C	5.375500	–1.011810	–2.310830	H	6.849063	2.958714	2.562007
C	4.602281	–2.174345	–2.457150	H	5.617515	3.058864	1.271490
C	4.682120	–3.154850	–1.461011	H	7.346945	3.049336	0.848428
C	5.488303	–2.988234	–0.334206	H	–2.765724	–3.269893	1.314596
C	3.724076	–2.390924	–3.679193	H	3.068397	–3.605347	3.067105
C	2.240262	–2.133823	–3.465019	H	4.133924	–3.835955	4.482710
C	–2.393228	1.287274	2.052576	H	2.365612	–3.754070	4.701342
C	–2.500477	0.571000	0.713400	H	6.321400	1.615931	–2.618652
C	–2.336619	–0.819153	0.663361	H	6.103532	1.425806	–4.371155
C	–2.288580	–1.516996	–0.542881	H	7.282487	0.401312	–3.500111
C	–2.365480	–0.815454	–1.760500	H	–0.412635	2.071194	–5.316334
C	–2.608547	0.559473	–1.717521	H	1.204759	1.798208	–4.627162
C	–2.692868	1.255607	–0.503037	H	0.599910	0.723339	–5.917137
C	1.655771	–0.919901	–3.837895	H	–7.859105	–3.899341	0.331122
C	0.277132	–0.702167	–3.715441	H	–10.285898	–4.099741	0.024456
C	–0.555644	–1.717116	–3.216866	H	–11.700372	–2.076930	–0.311168
C	0.031264	–2.924058	–2.824916	H	–10.667743	0.162449	–0.239055
C	1.406327	–3.135886	–2.935203	H	–9.818551	1.819441	0.911579
C	–2.056506	–1.511643	–3.069892	H	–8.613407	3.906341	1.413751
O	2.017243	–4.311139	–2.574357	H	–4.939709	1.902488	0.446884
C	1.230243	–5.321401	–1.949595	H	–6.180394	5.163322	0.651151
O	5.586133	–3.919542	0.671542	H	–4.805230	4.179784	1.189700
C	4.734718	–5.059571	0.620743	H	–6.069965	4.655518	2.337636
O	6.646130	1.235460	1.392138	H	5.116972	2.765027	–1.261220
C	6.601261	2.653496	1.538706	H	5.689164	5.119772	–1.710453
O	–2.041557	–2.866062	–0.613856	H	3.878677	6.831285	–1.809501
C	–2.613813	–3.768535	0.354022	H	1.523957	6.181425	–1.528528
N	–6.487989	–1.541328	0.357716	H	–0.149649	–0.025825	–0.499227
O	3.214506	–1.957881	4.344594	H	3.821125	–1.089371	0.723184
C	3.208590	–3.365739	4.128522	H	4.585960	1.113288	–0.008624

Atom	x	y	z	Atom	x	y	z
O	5.254422	–0.060759	–3.297204	H	0.265652	–2.328825	0.304015
C	6.303742	0.892197	–3.441008	H	1.942730	–2.899212	0.298904
O	–0.336926	0.484026	–4.063297	H	1.274065	–2.144688	1.755671
C	0.312917	1.306644	–5.035257	H	–6.153035	–0.361970	–1.373459
O	–2.910870	2.615314	–0.422077	H	0.547079	2.150986	–2.518164
C	–7.847859	–1.739141	0.212157	H	–0.593293	2.004252	–1.167353
C	–8.460124	–3.005504	0.218920	H	–4.919493	–0.249002	–0.088442
C	–9.833142	–3.113293	0.041007	H	–1.861312	–4.545963	0.507010
C	–10.633802	–1.975635	–0.145132	C	–3.908625	–4.383499	–0.178830
C	–10.050477	–0.719527	–0.107361	H	–4.272548	–5.099453	0.569726
C	–8.663824	–0.575161	0.092230	H	–3.668764	–4.960046	–1.078586
C	–8.056654	0.723400	0.351131	C	–5.007921	–3.368004	–0.521248
C	–8.745384	1.867662	0.784939	H	–5.841289	–3.886529	–1.006650
C	–8.061218	3.033034	1.081479	H	–4.621953	–2.653742	–1.257359
C	–6.656959	3.094918	0.982023	C	–5.536411	–2.598484	0.698423
C	–6.012666	1.948246	0.563923	H	–4.715158	–2.114761	1.234887
N	–6.698624	0.820385	0.254260	H	–6.015419	–3.275251	1.411006
C	–5.879185	4.340470	1.307194	C	–3.363399	3.352331	–1.581406
N	0.660143	3.647315	–1.030356	H	–3.923600	4.191044	–1.159134
C	1.958431	4.085898	–1.229925	H	–4.065206	2.735080	–2.152882
C	2.992722	3.111069	–1.130753	C	–2.246604	3.886239	–2.483600
C	4.329416	3.506430	–1.316693	H	–1.651080	3.062690	–2.888824
C	4.654301	4.831683	–1.563759	H	–2.751746	4.330747	–3.349725
C	3.633293	5.791263	–1.618772	C	–1.349800	4.967146	–1.851625
C	2.301173	5.430079	–1.456063	H	–1.991259	5.778083	–1.483638
C	2.653637	1.761418	–0.688524	H	–0.712574	5.400246	–2.630910
N	1.349442	1.373116	–0.770039	C	–0.462845	4.519923	–0.677294
C	0.902691	0.162623	–0.357802	H	–1.071774	3.994563	0.062024
C	1.760166	–0.770394	0.189284	H	–0.044296	5.395821	–0.176887
C	3.108776	–0.397189	0.296515	O	–1.713743	–0.897690	3.683235
C	3.546900	0.846444	–0.125069	O	2.244962	2.907407	2.926861
C	1.273918	–2.112584	0.660384	C	–1.422025	–2.082027	4.421138
C	–5.981949	–0.363690	–0.288062	H	–0.604171	–2.649384	3.960260
C	0.409095	2.300915	–1.443504	H	–2.334121	–2.679378	4.398632
H	4.706176	2.081816	3.101378	H	–1.162384	–1.855082	5.461806
H	5.267415	–2.811130	2.770622	C	1.923595	4.171863	2.355188
H	7.383228	–2.467853	1.536692	H	1.609374	4.071247	1.313999
H	7.814150	–0.855896	0.948070	H	2.839434	4.762508	2.393996
H	3.177288	1.791300	4.924952	H	1.133354	4.678110	2.923069
H	2.749617	0.181301	5.510118	H	0.804648	–0.944588	4.703239
H	6.767441	0.059636	–1.049007	C	0.557903	–0.094937	4.080023
H	4.087648	–4.048164	–1.589906	H	–0.265107	2.963118	1.881444
H	3.846876	–3.426846	–4.010533	C	–0.026189	2.101617	2.492400
H	4.077217	–1.744422	–4.484618				

Table S20. Cartesian coordinates of the DFT energy-minimized structure of **2h**. CPCM model with MeOH.

Atom	x	y	z	Atom	x	y	z
C	–3.780589	–2.202332	–2.081750	H	–1.539415	–0.916613	4.807305
C	–4.413022	–0.994602	–1.778537	H	1.933107	–1.968052	4.696495
C	–4.699292	–0.712211	–0.430739	H	0.723419	–0.994222	5.541153
C	–4.324350	–1.610593	0.567200	H	–1.045172	5.211959	–2.681886
C	–3.648633	–2.801075	0.270443	H	–0.006283	4.203367	–3.690925
C	–3.389943	–3.097271	–1.076961	H	–0.675445	5.391737	–0.310066
C	–3.187516	–3.712136	1.400130	H	3.266875	2.439572	–0.702147
C	–2.073217	–3.100001	2.234934	H	3.491461	–0.934822	3.277624
C	–4.674084	0.048476	–2.848422	H	0.885963	3.254568	3.224340
C	–3.608677	1.133571	–2.822487	H	3.116570	4.397410	2.411732
C	–3.826996	2.340421	–2.150381	H	4.134365	3.363304	1.406819
C	–2.840932	3.331408	–2.093181	H	–1.933693	2.122713	5.152187
C	–1.619495	3.148206	–2.762805	H	–0.505316	3.191602	5.086537
C	–1.393149	1.934131	–3.418404	H	–1.318680	2.689259	3.577277
C	–2.359653	0.923908	–3.432645	H	–3.669542	–1.299171	5.538673
C	–0.733231	–3.393014	1.971641	H	–5.118106	–0.968861	4.551358
C	0.304287	–2.815645	2.714447	H	–3.624373	–0.030623	4.279492
C	0.017170	–1.900994	3.738516	H	–2.067502	–4.090233	–3.340626
C	–1.324803	–1.614797	4.010616	H	–3.678509	–4.821067	–3.092312
C	–2.359686	–2.210700	3.288883	H	–2.201671	–5.722402	–2.643140
C	1.121894	–1.249838	4.554675	H	–4.394994	0.986415	1.613815
C	1.683490	0.001431	3.904070	H	–5.826012	1.936780	1.124680
C	–0.559069	4.236208	–2.748891	H	–6.033578	0.295277	1.787039
C	0.412021	4.086786	–1.591815	H	1.679427	–4.637841	1.045373
C	0.201697	4.761903	–0.388104	H	1.465474	–5.170347	2.737874
C	1.068682	4.598852	0.697868	H	3.040962	–4.530597	2.180804
C	2.186726	3.753945	0.585880	H	5.830397	0.364485	3.392154
C	2.398750	3.082895	–0.624005	H	5.392119	–0.840895	2.154050
C	1.523927	3.234338	–1.704902	H	6.519866	0.471276	1.743319
C	2.924773	–0.011381	3.270458	H	2.181077	0.965145	–3.939322
C	3.422207	1.116059	2.599612	H	–0.153228	–4.380850	–1.524032
C	2.677025	2.302501	2.571152	H	1.060517	–6.342841	–2.423953
C	1.448052	2.330301	3.246496	H	3.539037	–6.238184	–2.675032
C	0.948168	1.205000	3.899995	H	4.790828	–4.260872	–1.981309
C	3.115358	3.515326	1.765973	H	1.609412	0.756102	0.888966
O	–0.249706	1.181806	4.560770	H	–2.100998	–0.414573	–0.925459
C	–1.034734	2.371386	4.587352	H	–0.973093	–2.411038	–1.804770
O	–3.688675	–1.993962	3.563150	H	–1.804864	1.919163	0.037479
C	–4.028649	–1.016049	4.542174	H	–1.556006	1.205352	1.639938
O	–2.685100	–4.254720	–1.340592	H	–0.345342	2.310410	0.966905
C	–2.664858	–4.735689	–2.684446	H	3.494611	–0.392778	0.423718
O	0.891102	5.219427	1.912514	H	2.946089	–1.954391	1.097467
C	–0.299797	5.975572	2.113971	H	2.539019	0.865880	–2.214340
O	–5.335541	0.477208	–0.181379	C	4.004653	1.990499	–3.397401

Atom	x	y	z	Atom	x	y	z
C	–5.395372	0.935373	1.165909	H	3.935834	2.827282	–4.101294
O	1.635080	–3.103445	2.483765	H	4.478659	2.387170	–2.493786
C	1.957653	–4.441246	2.085075	C	4.867626	0.875276	–4.013589
O	4.594523	1.083595	1.867533	H	5.900181	1.235197	–4.098720
C	5.633672	0.214495	2.324885	H	4.519802	0.686401	–5.037126
O	1.666333	2.614746	–2.920121	C	4.843519	–0.455269	–3.246568
C	2.580091	1.527211	–3.088376	H	3.826083	–0.864692	–3.257567
N	3.660049	–1.986775	–0.901122	H	5.464628	–1.185208	–3.781044
C	3.043184	–3.144984	–1.350332	C	5.324301	–0.373247	–1.793762
C	1.622301	–3.181560	–1.274054	H	4.819912	0.438701	–1.255793
C	0.923555	–4.343604	–1.650555	H	6.394860	–0.137848	–1.758170
C	1.602686	–5.445855	–2.146425	C	5.091875	–1.691841	–1.047566
C	2.996638	–5.383891	–2.281945	H	5.563026	–2.502410	–1.602105
C	3.712388	–4.257660	–1.891514	H	5.566209	–1.675223	–0.059364
C	0.905809	–1.981532	–0.869939	O	–2.986601	4.516841	–1.412009
N	1.582400	–1.031688	–0.163340	C	–4.143461	4.687413	–0.600591
C	0.981767	0.085209	0.320949	H	–4.225016	3.893030	0.152063
C	–0.361170	0.333772	0.103941	H	–4.020507	5.648273	–0.099272
C	–1.059654	–0.589060	–0.690254	H	–5.062309	4.708308	–1.199491
C	–0.430713	–1.717273	–1.182452	H	–4.777488	2.475726	–1.652346
C	–1.050908	1.518027	0.718290	O	–2.170901	–0.305629	–4.016176
C	2.994933	–1.331067	0.197337	C	–0.888095	–0.604262	–4.557218
H	–3.535240	–2.397946	–3.117536	H	–0.102510	–0.515610	–3.796815
H	–4.528738	–1.396954	1.606240	H	–0.643315	0.046491	–5.405605
H	–2.845517	–4.660222	0.982547	H	–0.940171	–1.638812	–4.899100
H	–4.041406	–3.926453	2.050531	H	–0.435077	1.793892	–3.900320
H	–5.654063	0.506853	–2.691347	H	–1.194909	5.358167	1.968564
H	–4.682337	–0.434389	–3.829042	H	–0.347121	6.841789	1.443165
H	–0.503067	–4.087028	1.170663	H	–0.263201	6.322005	3.147294

Table S21. Cartesian coordinates of the DFT energy-minimized structure of **1h**.

Atom	x	y	z	Atom	x	y	z
C	0.852679	–3.731986	–1.993134	H	1.673998	4.875496	–0.730568
C	1.734551	–4.013685	–0.947483	H	–2.597430	2.660531	–2.758274
C	1.208504	–4.370212	0.304747	H	–2.923876	4.359879	1.856890
C	–0.170997	–4.341936	0.513156	H	–0.277231	5.911360	0.057333
C	–1.049992	–3.992295	–0.511489	H	–0.914550	5.522652	1.660815
C	–0.530587	–3.740099	–1.790723	H	–6.185321	3.198944	2.675501
C	–2.526122	–3.805049	–0.237374	H	–4.444864	3.239371	3.033590
C	–3.036806	–2.407787	–0.551318	H	–5.214788	4.595966	2.157827
C	3.224126	–3.766027	–1.097377	H	0.229429	–1.176460	–0.720618
C	3.536492	–2.421094	–0.465007	H	0.718065	–1.099228	0.984754
C	3.989604	–2.339251	0.850842	H	–0.099428	0.298547	0.249508
C	4.143115	–1.107875	1.493599	H	–0.396598	–3.815511	–4.534239
C	3.794520	0.083575	0.839187	H	–0.389630	–2.123693	–3.955415
C	3.377544	0.005744	–0.495650	H	–1.882615	–2.834799	–4.628027
C	3.260352	–1.221482	–1.147340	H	0.354671	2.492003	4.440887
C	–4.230055	–2.246684	–1.269152	H	–1.317837	3.024190	4.619032
C	–4.740643	–0.980940	–1.559734	H	2.531068	–5.188225	3.189918
C	–4.048350	0.177383	–1.160491	H	1.115741	–4.113043	3.018865
C	–2.859130	0.012797	–0.451455	H	0.987258	–5.844474	2.592967
C	–2.366202	–1.253725	–0.123544	H	–7.347552	–1.459899	–3.476396
C	–4.642285	1.545830	–1.475323	H	–6.892102	–2.623183	–2.204569
C	–3.773269	2.671266	–0.978795	H	–5.801253	–2.339772	–3.593733
C	3.883867	1.422329	1.554303	H	–0.291342	2.709133	–3.234985
C	2.712669	2.349971	1.306763	H	–1.197412	4.076453	–3.949167
C	1.586490	2.303786	2.138299	H	0.545109	4.240388	–3.615785
C	0.513198	3.181173	1.959840	H	3.491840	4.906865	–1.793742
C	0.559048	4.140682	0.927624	H	–5.620123	–3.261915	1.940670
C	1.667160	4.162042	0.083754	H	–6.779153	–5.248727	1.055804
C	2.731687	3.274427	0.255645	H	–8.888591	–5.013666	–0.244818
C	–2.681527	3.071706	–1.759088	H	–9.755131	–2.768601	–0.779932
C	–1.694688	3.917928	–1.254570	H	–9.545485	–0.981329	–2.040326
C	–1.770597	4.360987	0.077771	H	–9.704342	1.301057	–2.951274
C	–2.890776	4.019372	0.831094	H	–7.415378	2.691955	0.415002
C	–3.896948	3.198425	0.313381	H	–9.605943	3.970163	–2.049762
C	–0.602305	5.092750	0.705132	H	–8.113820	3.612609	–2.921951
O	–5.012219	2.825563	1.059244	H	–8.038575	4.309760	–1.297667
C	–5.208548	3.508925	2.296482	H	5.502640	–1.696227	–3.096046
O	–1.274848	–1.388072	0.712239	H	5.621953	0.022385	–4.862351
C	–0.047543	–0.796908	0.268795	H	6.968031	2.085499	–4.478273
O	–1.455272	–3.467404	–2.764244	H	8.075512	2.481224	–2.336845
C	–0.991015	–3.040216	–4.033885	H	7.719730	–1.940978	2.462612
O	–0.613571	3.200128	2.742248	H	6.877843	–5.075283	–0.358905
C	–0.635534	2.464184	3.970514	H	6.537201	–3.347078	–2.082896
N	–6.464870	–0.719802	1.628530	H	8.335405	–5.390594	2.019691

Atom	x	y	z	Atom	x	y	z
O	2.127280	–4.682013	1.280099	H	7.783484	–4.249567	3.253973
C	1.649186	–4.969567	2.584765	H	6.622309	–5.302747	2.428601
O	–5.924437	–0.765709	–2.225160	H	–6.770083	1.288662	2.008830
C	–6.513127	–1.869530	–2.905233	H	6.292276	0.379702	1.018708
O	–0.600421	4.334758	–1.961179	H	7.883628	0.165929	1.793520
C	–0.388834	3.803260	–3.259561	H	–8.176422	0.238804	2.375153
O	3.799042	3.197286	–0.619166	C	–1.133354	1.025732	3.815550
C	4.287653	4.396459	–1.238048	H	–0.944284	0.517429	4.771266
C	–7.034470	–1.860360	1.107314	H	–0.540903	0.500145	3.057768
C	–6.513977	–3.147694	1.339165	C	–2.625405	0.908580	3.472636
C	–7.173424	–4.260140	0.842574	H	–2.794984	1.294934	2.460812
C	–8.361827	–4.131055	0.099897	H	–3.198096	1.552162	4.157109
C	–8.848886	–2.870558	–0.191960	C	–3.138125	–0.534386	3.609206
C	–8.180435	–1.714593	0.269094	H	–2.892073	–0.893553	4.616153
C	–8.455162	–0.398959	–0.281606	H	–2.590568	–1.175383	2.906053
C	–9.122445	–0.148814	–1.494278	C	–4.653924	–0.702777	3.394524
C	–9.190571	1.130192	–2.009854	H	–5.201392	0.041152	3.990043
C	–8.569391	2.215804	–1.354881	H	–4.973935	–1.684970	3.762508
C	–7.941691	1.939402	–0.158288	C	–5.031942	–0.573017	1.918081
N	–7.924002	0.682818	0.359149	H	–4.503068	–1.320462	1.329086
C	–8.580691	3.604902	–1.931791	H	–4.725596	0.399154	1.528610
N	7.991422	0.779854	–0.189921	H	5.008923	4.032154	–1.974319
C	7.434646	0.614852	–1.447951	C	4.954393	5.347968	–0.242504
C	6.757705	–0.613131	–1.709246	H	5.456156	6.129691	–0.827059
C	6.092522	–0.800714	–2.937993	H	4.186436	5.849037	0.356549
C	6.143880	0.167932	–3.923436	C	5.957384	4.657007	0.699240
C	6.895966	1.332324	–3.699822	H	5.401278	4.092621	1.457426
C	7.533394	1.558069	–2.488384	H	6.522337	5.424321	1.241810
C	6.898971	–1.695947	–0.756520	C	6.916447	3.703344	–0.021114
N	7.266689	–1.383547	0.517902	H	6.323016	2.911434	–0.487192
C	7.463619	–2.320846	1.481659	H	7.426536	4.235962	–0.835732
C	7.323591	–3.667535	1.221530	C	7.961559	3.063992	0.897863
C	6.979183	–4.025492	–0.100523	H	7.463418	2.657913	1.789798
C	6.776571	–3.063073	–1.067130	H	8.662499	3.821938	1.267952
C	7.528665	–4.703207	2.293488	C	8.783519	1.947131	0.229236
C	–7.328283	0.411481	1.698300	H	9.322190	2.321584	–0.643576
C	7.326622	0.059270	0.863447	H	9.547256	1.580329	0.925003
H	1.270596	–3.455788	–2.952290	O	4.688393	–0.991672	2.758343
H	–0.583365	–4.535285	1.495884	C	4.572704	–2.109594	3.632540
H	–2.715158	–4.033305	0.818636	H	5.241377	–2.929769	3.338938
H	–3.110542	–4.520895	–0.825903	H	4.861377	–1.755033	4.623392
H	3.495175	–3.765870	–2.156372	H	3.544052	–2.488346	3.661897
H	3.797771	–4.550696	–0.596371	H	4.191966	–3.262768	1.375318
H	–4.754478	–3.135892	–1.596879	O	2.874811	–1.352525	–2.458334
H	–2.320872	0.885228	–0.100741	C	2.461485	–0.182122	–3.153468
H	–5.642369	1.600432	–1.040452	H	2.155290	–0.516105	–4.146089
H	–4.791734	1.624238	–2.558709	H	3.281949	0.539411	–3.249990

Atom	x	y	z	Atom	x	y	z
H	4.798465	1.938844	1.240514	H	1.612259	0.300637	–2.652801
H	3.978636	1.237043	2.626242	H	3.155555	0.929529	–1.011714
H	1.571511	1.568542	2.934130				

Table S22. Cartesian coordinates of the DFT energy-minimized structure of **1h**. CPCM model with MeOH.

Atom	x	y	z	Atom	x	y	z
C	–7.053540278	–1.706204	–1.374894	H	0.722438	–0.187535	–1.776189
C	–6.953116308	–2.393021	–0.161283	H	–1.590553	4.176698	–1.803216
C	–7.181217403	–1.688699	1.032914	H	–0.172388	2.112437	2.455476
C	–7.547403216	–0.339923	0.989515	H	1.997473	1.378416	–0.412714
C	–7.660924268	0.343539	–0.224975	H	1.873336	1.366563	1.341713
C	–7.38589177	–0.348573	–1.416310	H	–2.652470	3.639479	4.587305
C	–8.015461568	1.819840	–0.245153	H	–2.077381	2.145079	3.799613
C	–6.77905479	2.690996	–0.116056	H	–0.924381	3.456046	4.182250
C	–6.546568033	–3.855832	–0.137188	H	–5.527256	2.519340	3.673769
C	–5.037722038	–4.013675	–0.085671	H	–7.221955	2.472444	4.232182
C	–4.37027963	–4.044206	1.142514	H	–6.493145	4.020950	3.730472
C	–2.976565386	–4.133071	1.209047	H	–7.207383	0.503444	–4.576202
C	–2.221730236	–4.220730	0.027205	H	–7.797540	–1.089323	–4.031883
C	–2.88849363	–4.187559	–1.200844	H	–6.088973	–0.635365	–3.777531
C	–4.279945805	–4.070196	–1.267584	H	–0.098606	–0.024643	4.541000
C	–6.05505586	3.064609	–1.252492	H	–0.761224	–1.414676	3.672161
C	–4.882881823	3.819689	–1.150220	H	–6.452683	–0.877077	3.512542
C	–4.425678282	4.240707	0.110194	H	–8.183113	–1.316128	3.566288
C	–5.148328991	3.865053	1.246087	H	–6.957732	–2.437805	4.215825
C	–6.304936301	3.085815	1.146018	H	–4.487209	2.597950	–3.529503
C	–3.130430957	5.022717	0.241554	H	–3.710429	4.057910	–4.200879
C	–1.938569444	4.084872	0.294139	H	–5.455972	4.067457	–3.833795
C	–0.705230862	–4.271595	0.077120	H	–1.103426	2.414389	–3.338221
C	–0.130002561	–2.869479	0.157074	H	–0.038567	3.848754	–3.427661
C	0.106811847	–2.277066	1.399882	H	0.545739	2.267615	–4.002261
C	0.533323836	–0.947863	1.512052	H	–0.505601	–1.015076	–3.318279
C	0.799365762	–0.209782	0.347660	H	7.058299	1.082070	3.056389
C	0.556954995	–0.798723	–0.899194	H	9.472309	0.613598	2.810192
C	0.083105714	–2.109791	–1.009024	H	10.257798	–1.122985	1.207192
C	–1.257785812	3.733628	–0.873959	H	8.622374	–2.439721	–0.077973
C	–0.211964163	2.805929	–0.852450	H	7.170540	–4.051128	–0.204444
C	0.190233329	2.232613	0.361547	H	5.359355	–5.464766	–1.086299
C	–0.487552606	2.583833	1.533241	H	2.595320	–2.283987	–0.227076
C	–1.546688437	3.493053	1.509591	H	1.878491	–4.246161	–1.690312
C	1.309518811	1.216127	0.418154	H	2.210160	–5.411850	–0.404523
O	–2.258219603	3.863738	2.620884	H	3.009637	–5.584224	–1.971338
C	–1.949009983	3.232914	3.859984	H	7.893409	2.782267	0.119761
O	–7.035748917	2.662208	2.231845	H	9.431412	1.158141	–0.918352
C	–6.529858681	2.942467	3.532310	H	8.595050	–0.391458	–2.674820
O	–7.468735785	0.382456	–2.578885	H	6.270010	–0.254839	–3.468230
C	–7.119343358	–0.258461	–3.800623	H	2.119076	4.082886	–0.958747
O	0.70459532	–0.289092	2.706210	H	4.403271	4.316984	2.676812
C	0.163489715	–0.876696	3.906234	H	6.221172	3.099203	1.540838
N	5.082664963	–0.139539	1.720247	H	2.276129	6.059088	1.946248

Atom	x	y	z	Atom	x	y	z
O	–7.025916487	–2.401329	2.199322	H	1.101562	5.163413	0.955874
C	–7.166495693	–1.706817	3.433617	H	1.628250	4.513906	2.510680
O	–4.11655223	4.193556	–2.228833	H	4.632282	0.063571	–0.298864
C	–4.475207173	3.695047	–3.512873	H	2.816712	2.831288	–2.631371
O	0.488934977	2.422176	–1.983602	H	4.409097	3.571417	–2.994039
C	–0.069642742	2.767365	–3.254026	H	3.274638	–0.431751	0.741590
O	–0.185762261	–2.741881	–2.198815	C	1.191864	–1.767137	4.597490
C	–0.052754197	–2.009060	–3.426501	H	1.575528	–2.481585	3.861221
C	6.455071495	–0.419607	1.635539	H	0.682107	–2.363575	5.362937
C	7.396580726	0.300945	2.384700	C	2.346171	–0.956891	5.228493
C	8.752327442	0.035221	2.240296	H	2.146856	–0.819381	6.297088
C	9.196552129	–0.945250	1.341309	H	2.356032	0.051403	4.799390
C	8.274089255	–1.685415	0.618609	C	3.734952	–1.592866	5.055313
C	6.89244376	–1.460545	0.777439	H	3.712506	–2.625090	5.426525
C	5.898866825	–2.367847	0.204655	H	4.455743	–1.054777	5.684598
C	6.15997392	–3.668151	–0.247148	C	4.257882	–1.594690	3.609402
C	5.13327319	–4.466366	–0.726089	H	3.593576	–2.178473	2.960240
C	3.802337519	–4.010767	–0.739689	H	5.235651	–2.090456	3.581494
C	3.579976166	–2.732780	–0.258033	C	4.409026	–0.180843	3.031490
N	4.599608332	–1.955369	0.176667	H	4.978591	0.449260	3.720190
C	2.660907896	–4.857032	–1.234608	H	3.433545	0.294124	2.903846
N	4.417111501	1.509091	–2.527353	H	–0.653731	–2.565447	–4.150559
C	5.739332121	1.368147	–2.141432	C	1.401484	–1.909851	–3.886544
C	6.192455928	2.203578	–1.080887	H	2.012276	–1.582185	–3.042494
C	7.534887804	2.125035	–0.664270	H	1.763623	–2.910202	–4.150242
C	8.400798621	1.214151	–1.246839	C	1.574000	–0.959968	–5.082245
C	7.925395924	0.341396	–2.236410	H	1.054131	–0.012732	–4.881107
C	6.612268559	0.409711	–2.684245	H	1.065296	–1.398519	–5.948705
C	5.200329891	2.975911	–0.343821	C	3.045718	–0.665962	–5.444900
N	4.018274	3.259935	–0.964379	H	3.655918	–1.555348	–5.237541
C	3.002422	3.921503	–0.356284	H	3.122943	–0.490867	–6.523322
C	3.095591	4.338776	0.957138	C	3.667646	0.552505	–4.738395
C	4.293049	4.034520	1.634757	H	3.136220	1.463930	–5.041649
C	5.322834	3.362908	0.999289	H	4.703646	0.671778	–5.075738
C	1.958825	5.062392	1.623866	C	3.657023	0.460028	–3.206980
C	4.340966	–0.531710	0.568493	H	2.633703	0.513717	–2.825434
C	3.876999	2.827542	–2.387872	H	4.065290	–0.500995	–2.881070
H	–6.839454	–2.256618	–2.282133	O	–2.264822	–4.139026	2.385521
H	–7.728360	0.219272	1.898712	C	–2.976295	–3.945720	3.603544
H	–8.701641	2.035616	0.577127	H	–3.524640	–2.995412	3.602425
H	–8.528095	2.058965	–1.179708	H	–3.680420	–4.764465	3.796371
H	–6.935093	–4.351127	–1.030173	H	–2.222447	–3.926515	4.391874
H	–6.989116	–4.343240	0.734641	H	–4.968421	–3.966430	2.041635
H	–6.418083	2.723832	–2.214036	O	–4.984156	–4.002837	–2.446921
H	–4.764864	4.170068	2.210835	C	–4.250142	–3.935432	–3.664504
H	–3.163448	5.627422	1.151055	H	–4.991417	–3.825757	–4.457074
H	–3.019421	5.699824	–0.608825	H	–3.667479	–4.848503	–3.838970

Atom	x	y	z	Atom	x	y	z
H	–0.333067	–4.777901	–0.815532	H	–3.573267	–3.071715	–3.678524
H	–0.379815	–4.844797	0.948694	H	–2.289099	–4.224879	–2.101146
H	–0.093820	–2.872516	2.279782				
